# A Novel Immune-Related Gene Signature Predicts Prognosis of Lung Adenocarcinoma

**DOI:** 10.1155/2022/4995874

**Published:** 2022-04-09

**Authors:** Chao Ma, Feng Li, Ziming Wang, Huan Luo

**Affiliations:** ^1^Department of Thoracic Surgery, First Affiliated Hospital of Zhengzhou University, Zhengzhou, China; ^2^Charité – Universitätsmedizin Berlin, Corporate Member of Freie Universität Berlin, Humboldt-Universität zu Berlin, and the Berlin Institute of Health, Berlin, Germany; ^3^Department of Thoracic Surgery, Klinikum Ernst von Bergmann Potsdam, Academic Hospital of the Charité – Universitätsmedizin Humboldt University Berlin, Potsdam, Germany

## Abstract

**Background:**

Lung adenocarcinoma (LUAD) is the most common form of lung cancer, accounting for 30% of all cases and 40% of all non-small-cell lung cancer cases. Immune-related genes play a significant role in predicting the overall survival and monitoring the status of the cancer immune microenvironment. The present study was aimed at finding an immune-related gene signature for predicting LUAD patient outcomes.

**Methods:**

First, we chose the TCGA-LUAD project in the TCGA database as the training cohort for model training. For model validating, we found the datasets of GSE72094 and GSE68465 in the GEO database and took them as the candidate cohorts. We obtained 1793 immune-related genes from the ImmPort database and put them into a univariate Cox proportional hazard model to initially look for the genes with potential prognostic ability using the data of the training cohort. These identified genes then entered into a random survival forests-variable hunting algorithm for the best combination of genes for prognosis. In addition, the LASSO Cox regression model tested whether the gene combination can be further shrinkage, thereby constructing a gene signature. The Kaplan-Meier, Cox model, and ROC curve were deployed to examine the gene signature's prognosis in both cohorts. We conducted GSEA analysis to study further the mechanisms and pathways that involved the gene signature. Finally, we performed integrating analyses about the 22 TICs, fully interpreted the relationship between our signature and each TIC, and highlighted some TICs playing vital roles in the signature's prognostic ability.

**Results:**

A nine-gene signature was produced from the data of the training cohort. The Kaplan-Meier estimator, Cox proportional hazard model, and ROC curve confirmed the independence and predictive ability of the signature, using the data from the validation cohort. The GSEA analysis results illustrated the gene signature's mechanism and emphasized the importance of immune-related pathways for the gene signature. 22 TICs immune infiltration analysis revealed resting mast cells' key roles in contributing to gene signature's prognostic ability.

**Conclusions:**

This study discovered a novel immune-related nine-gene signature (BTK, CCR6, S100A10, SEMA3C, GPI, SCG2, TNFRSF11A, CCL20, and DKK1) that predicts LUAD prognosis precisely and associates with resting mast cells strongly.

## 1. Introduction

Lung cancer is the leading cause of tumor-related death worldwide and ranks second in incidence among malignancies [1]. Lung adenocarcinoma (LUAD) is the most common histological type of lung cancer, accounting for approximately 40% of lung cancer cases [2, 3]. In the past decade, the treatment of LUAD patients has made great progress, including surgery, radiotherapy, chemotherapy, or targeted therapy [4, 5]. However, the outcomes of patients with LUAD recurrence are still poor [6]. Recent research has confirmed the potential of the targeted therapy, which blocks the upgrowth of lung cancer cells by inhibiting the initiation of vital oncogenic molecules that drive the progression of LUAD [6]. Although targeted therapy has achieved gratifying results in the early treatment of LUAD and shown promising potential, drug resistance's existence and continuous development often directly lead to treatment failure [7, 8]. Therefore, it is still necessary to continue efforts to optimize predicting methods to improve the current situation [9].

Recently, some studies have shown that the prediction model based on RNA sequencing data could precisely predict the survival of patients with cancers [10–14]. The immune microenvironment, including immune cells associated with immune-related genes, has a significant impact on predicting the prognosis of cancers, including lung cancer [11, 15]. In the present study, we first developed an epigenetic-related prognostic signature based on a TCGA dataset and then validated it in two GEO datasets. Moreover, we evaluated the prediction ability of the signature via the Kaplan-Meier estimator, univariate and multivariate Cox analysis, and ROC curve. More importantly, we further studied the functional annotation and the immune microenvironment characteristics of the gene signature.

## 2. Materials and Methods

### 2.1. Public Dataset Selection

We downloaded the level 3 gene expression data and the clinical characteristics of LUAD patients from the GDC Xena Hub (project: TCGA-LUAD, https://gdc.xenahubs.net) and GEO database (datasets: GSE72094 and GSE68465, https://www.ncbi.nlm.nih.gov/geo/). We excluded the samples without prognostic record. In the present work, we listed TCGA-LUAD as a training cohort for model training and used datasets of GSE72094 and GSE68465 for validating the model we built. Since the TCGA and GEO databases are open to researchers, we fully comply with publication guidelines and database access policies.

### 2.2. Immune-Related Genes

The Immunology Database and Analysis Portal (ImmPort, updated: July 2020, https://www.immport.org/home) [16] is developed under the ImmPort Contract by the Northrop Grumman Information Technology Health Solutions team for the National Institutes of Health, National Institute of Allergy and Infectious Diseases, and Division of Allergy, Immunology, and Transplantation. In this study, we found 1793 unique immune-related genes in the ImmPort database, which are displayed in Table S1.

### 2.3. Prognostic Immune-Related Gene Signature Construction and Validation

The immune-related genes of the training cohort were put into a univariate Cox proportional hazard model for the selection of potential prognostic genes (*p* < 0.05). Subsequently, the random survival forests-variable hunting (RSFVH) algorithm was performed on these potential prognostic genes for further filtering. The prognosis of the optimized combination of genes was found. The combination of genes was examined by the LASSO Cox regression (10-fold cross-validation) to identify a shrinkage possibility and discover the best penalty parameter [17–20]. The risk scores were calculated as shown in the following equation:

(1) $$\begin{array}{c} \text{Risk score}=\overset{n}{\underset{i}{\sum }}\text{E}\text{xpi}*\beta i. \end{array}$$

where *n* is the number of hub genes; Expi is the gene expression level; and *β*i is the coefficient.

According to the median risk score, patients were divided into low-risk or high-risk groups. Kaplan-Meier estimator was deployed to compare the survival difference between the high- and low-risk groups. Based on the patient's risk score data, we established univariate and multivariate Cox hazard models and ROC receiver operating characteristics (ROC curves) in the training cohort and validation cohort to evaluate the prognostic ability of the gene signature.

### 2.4. Function Annotated by Gene Set Enrichment Analysis (GSEA)

We conducted GSEA (http://www.broadinstitute.org/gsea/index.jsp) to identify the possible mechanisms between high- and low-risk groups in LUAD patients. We downloaded the HALLMARK gene set collection from the Molecular Signatures Database (v7.1, https://www.gseamsigdb.org/gsea/msigdb/genesets.jsp). For each analysis, the number of permutations was set to 1000 times, and we defined ∣ NES  | >1, NOM *p* value <0.05, and FDR *q* value <0.25 as statistically significant.

### 2.5. 22 Tumor-Infiltrating Immune Cells (TICs) Analysis

The CIBERSORT algorithm was applied to calculate the relative proportion of 22 TICs in the training cohort [21, 22]. The Pearson coefficient tested the correlations between 22 TICs. In order to determine the relationship between 22 TICs and signatures, we conducted a comprehensive analysis including Spearman's coefficient and Wilcoxon rank sum test. Additionally, we used univariate Cox proportional hazard models and Kaplan-Meier estimators to evaluate the prognostic ability of each TIC. In the end, we combined the above analysis results and tried to find out potential candidate TICs that play vital roles in the prognostic ability of the gene signature.

### 2.6. Statistical Analysis

The RSFVH algorithm was implemented with the “randomForestSRC” R package. We used the “glmnet” R package for performing the LASSO regression analysis. Kaplan-Meier estimator was built by applying the “survival” and “survminer” R packages. Also, the “survival” R package construed the Cox proportional hazard models. In addition, the “pROC” R package helped in plotting the ROC curves. In the present study, *p* value <0.05 indicates statistical significance.

## 3. Results

### 3.1. Cohorts' Characteristics

**Figure 1:**
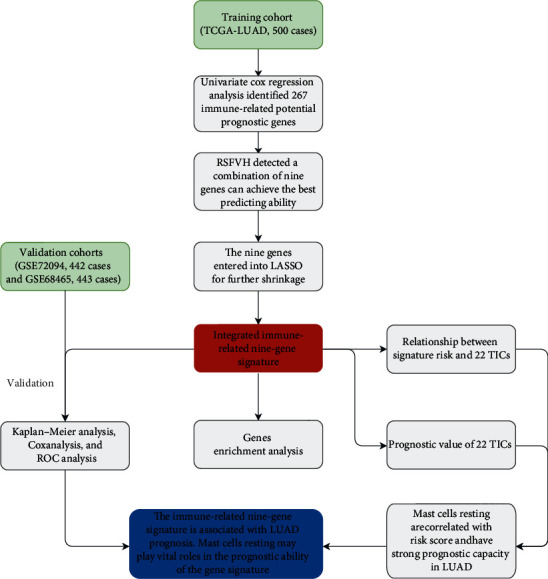
Flowchart of the critical step in the study. LASSO: the least absolute shrinkage and selection operator Cox regression model; RSFVH: random survival forests-variable hunting algorithm; ROC: receiver operating characteristic; LUAD: lung adenocarcinoma; TICs: tumor-infiltrating immune cells.

The present study's flow diagram is displayed in Figure 1. We took 500 LUAD cases from the TCGA-LUAD into the training cohort for model training. The datasets of GSE72094 (*n* = 442) and GSE68465 (*n* = 443) were chosen for model validating. In addition, we collected the clinical characteristics from these cohorts and showed them in Table 1.

### 3.2. Construction of a Prognostic Immune-Related Gene Signature

**Figure 2:**
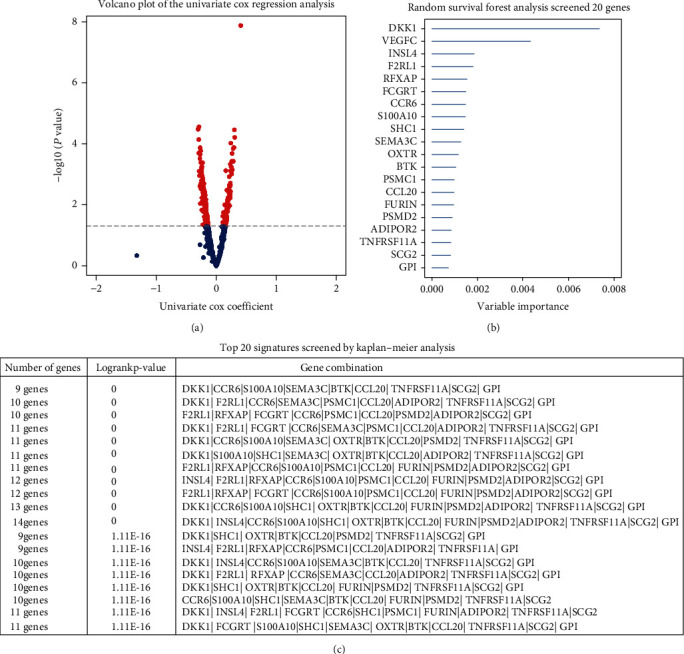
Identification of candidate genes and development of a potential prognostic gene signature. (a) The volcano plot shows the genes of the univariate Cox regression analysis. (b) Random survival forest analysis screened 20 genes. (c) After performing KM analysis on 2^20^ − 1 = 1,048,575 combinations, the log-rank *p* value sorted the top 20 signatures. The selected signature included nine genes. KM: Kaplan-Meier.

**Figure 3:**
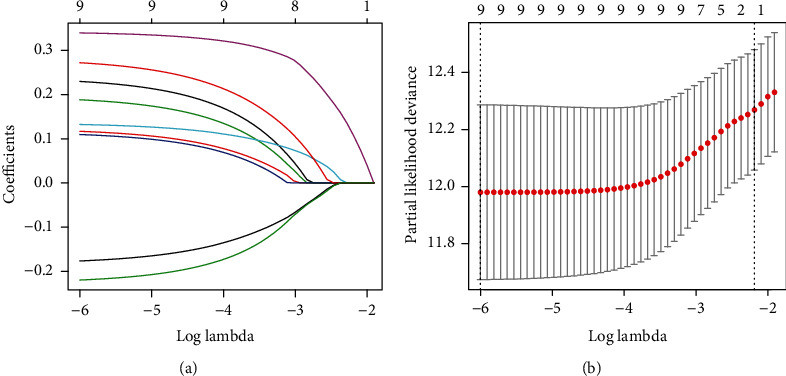
Combination of nine prognostic genes tested in the LASSO regression model. (a) Cross-validation for tuning parameter screening upon LASSO regression analysis. (b) Screening of optimal parameter (lambda) at which the vertical lines were drawn. LASSO: the least absolute shrinkage and selection operator Cox regression model.

We performed overall survival-based univariate Cox analysis on the LUADs in the training cohort to screen 1,793 immune genes and found that 267 of them have potential prognostic significance (Table S2 and Figure 2(a)). Subsequently, from the 267 genes, we screened out top 20 genes (DKK1, VEGFC, INSL4, F2RL1, RFXAP, FCGRT, CCR6, S100A10, SHC1, SEMA3C, OXTR, BTK, PSMC1, CCL20, FURIN, PSMD2, ADIPOR2, TNFRSF11A, SCG2, and GPI) by the random forest-supervised classification algorithm (Figure 2(b)). Since 20 genes can form a total of 2^20^ − 1 = 1,048,575 signatures, we used Kaplan-Meier analysis to further evaluate the above signatures to screen for the best one. By assessing the *p* values in the log-rank test of these 1,048,575 signatures, we discovered a nine-gene signature comprising DKK1, CCR6, S100A10, SEMA3C, BTK, CCL20, TNFRSF11A, SCG2, and GPI ranked top (Figure 2(c)). We listed the top 1000 signatures in Table S3. Furthermore, a LASSO Cox model was built using the above nine genes to check whether further minimizing the number of genes was possible (Figure 3(a)). And we found that when all nine genes were present, the LASSO Cox model could achieve its best state (Figure 3(b)).  Table 2 shows the regression coefficient of each gene.

### 3.3. Validating the Prognostic Capacity of the Nine-Gene Signature

**Figure 4:**
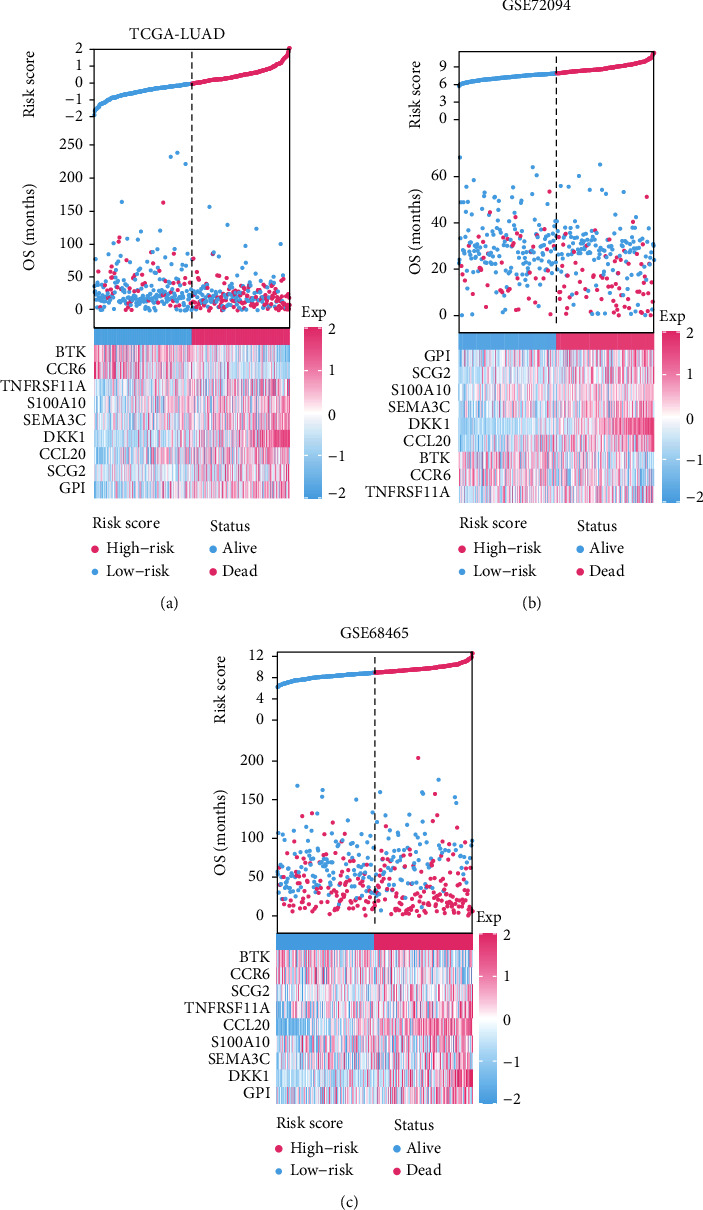
The overall distributions of the risk score (upper), survival status (middle), and gene expression profiles (bottom) of the nine-gene signature in the training (a) and validation (b and c) cohorts.

According to the median risk score, LUADs were assigned to a high-risk group or a low-risk group. In Figure 4, we showed the specific distribution of risk scores, the distribution of patients' survival status and survival time, and the expression distribution of genes in the signature in the high- and low-risk groups. Besides, we checked the nine-gene signature's distribution overall view along with the distributions of survival status, survival times, and signature's gene expression in a five-year period (Figure S1) and found a highly consistent pattern with those shown in Figure 4.

**Figure 5:**
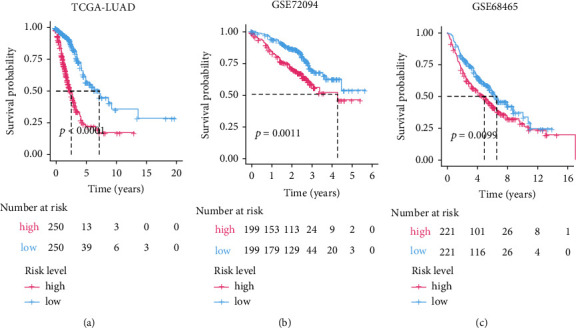
Kaplan-Meier estimator evaluating the prognosis capacity of the nine-gene signature in the training (a) and validation (b and c) cohorts. The bottom part indicates the number of patients at risk. The two-sided log-rank test measured the differences between the high- and low-risk groups with a *p* value < 0.05.

Kaplan-Meier estimators found significant survival differences between high- and low-risk groups in all the cohorts we have tested. In specifically, the high-risk patients suffered unfavorable outcomes in TCGA-LUAD (*p* value < 0.0001, Figure 5(a)), GSE72094 (*p* value = 0.0011, Figure 5(b)), and GSE68465 (*p* value = 0.0099, Figure 5(c)). Consistently, in the five-year overall survival-based Kaplan-Meier estimators, the high-risk groups exhibited a poorer prognosis than the low-risk groups (Figure S2).

**Figure 6:**
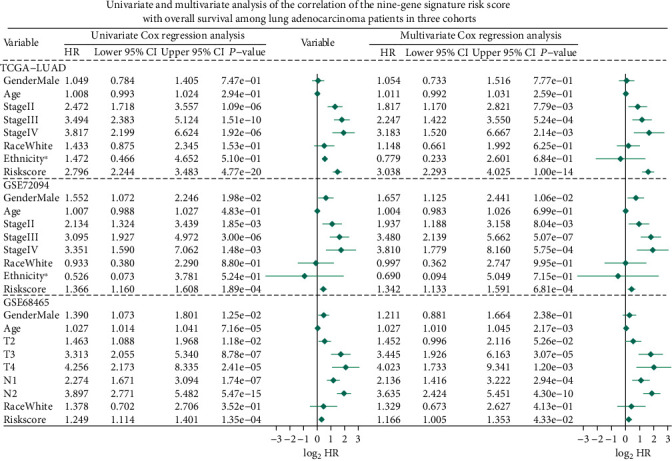
Univariate and multivariate Cox proportional hazard models built for testing the predicting ability of the nine-gene signature in three studied cohorts. ∗Hispanic or Latino vs. non-Hispanic or Latino. HR: hazard ratio; CI: confidence interval.

In order to test the prognostic ability and independence of gene signature, we established univariate and multivariate Cox proportional hazard models (Figure 6) in all cohorts in this section and incorporated available factors into these models, which included risk score, gender, age, race, tumor stage, ethnicity, T classification, or N classification. The Cox models established based on the TCGA-LUAD cohort data showed that the gene signature was a powerful prognostic factor, whether in univariate (*p* value = 4.77E-20) or multivariate (*p* value = 1.00E-14) analysis. Similarly, we found that gene signature showed strong and stable prognostic ability in all established Cox models in the two validation cohorts (*p* value ≤ 4.33E-02). The above results have exhibited that whether it was tested in the training cohort or the validation cohorts and whether it underwent through univariate or multivariate Cox analysis, the gene signature we found showed stable, independent, and powerful prognostic ability.

**Figure 7:**
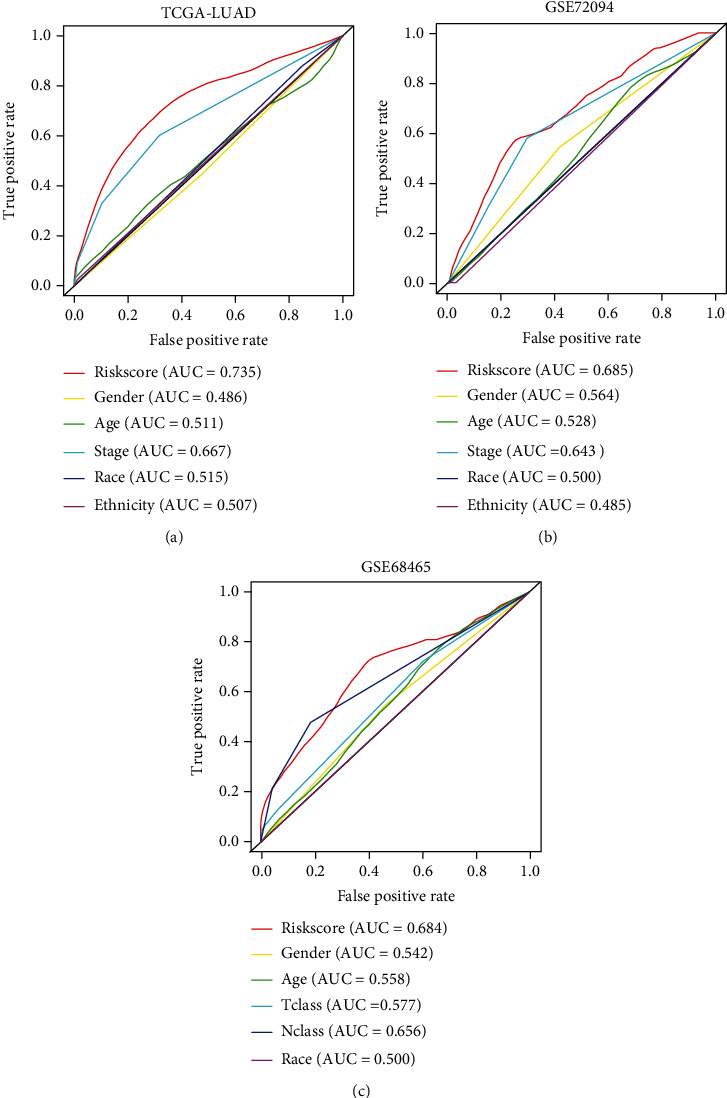
ROC curves constructed for examining the predictive ability of the nine-gene signature in the training (a) and validation (b and c) cohorts. ROC: receiver operating characteristic; AUC: area under the ROC curve; Tclass: T classification; Nclass: N classification.

The ROC curves were established to evaluate the capacity of the nine-gene signature in the LUAD outcome predicting.  Figure 7(a) shows the ROC curve we built using the training cohort data. Through comparison, we found that the AUC of the gene signature reached 0.735, which is the highest value among all clinical characteristics.  Figure 7(b) confirmed the superiority of the gene signature in the validation cohort of GSE72094. In this model, the risk score AUC reaches 0.685, which topped among all factors. Finally, Figure 7(c) showed that the gene signature AUC reached 0.684, better than the second-ranked N classification (AUC = 0.656).

### 3.4. GSEA

**Figure 8:**
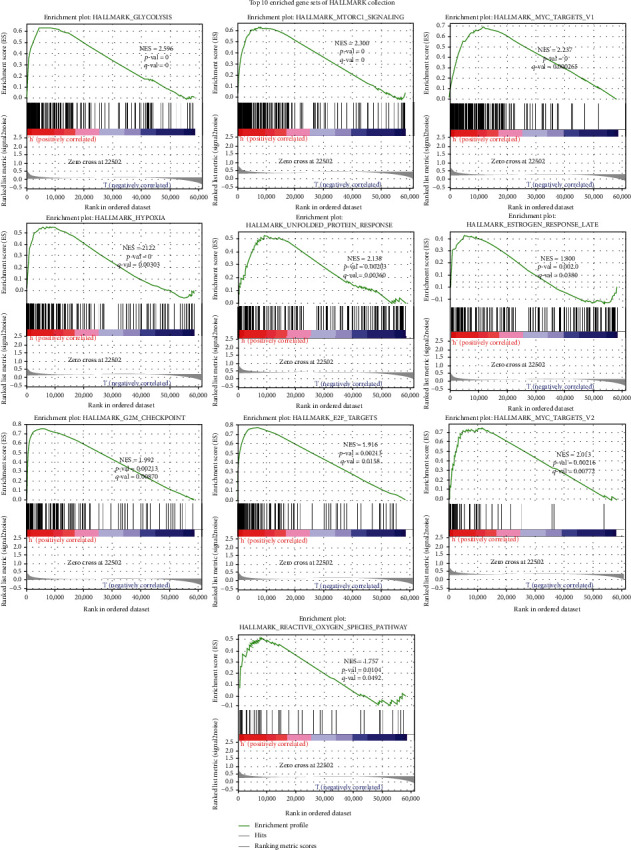
Top enriched item of GSEA performed with the nine-gene signature in LUAD using HALLMARK collection. ∣NES | >1, NOM *p* value < 0.05, and FDR *q* value <0.25 are set as the significance threshold. GSEA: gene set enrichment analysis.

In order to learn more about the mechanism and related pathways of the gene signature, we conducted the GSEA enrichment analysis between the high-risk group and the low-risk group based on the risk score of each case in the TCGA cohort. As shown (Figure 8 and Table S4), the enriched gene sets were all detected in the high-risk group and primarily involved in mechanism associated with glycolysis, mTORC1, MYC, hypoxia, unfolded protein response, estrogen, G2/M checkpoint, E2F, and reactive oxygen species.

### 3.5. 22 TICs Analysis

The GSEA analysis suggested that the difference between the two groups was related to the immune response, so we conducted 22 TIC analysis to further learn the interactions between the signature and the immune microenvironment. We run the CIBERSORT algorithm to calculate the relative content of 22 TICs in each patient in the TCGA cohort. As shown in Figure S3, we used the R software to output the 22 TIC visualization panorama and calculate the correlation of each two TICs.

**Figure 9:**
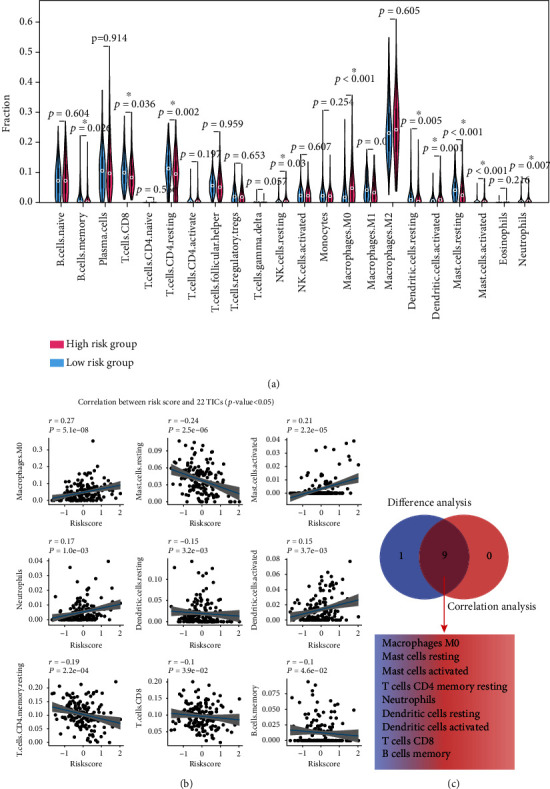
Integrating analysis for the relationship between TICs and the nine-gene signature. (a) The Wilcoxon rank-sum test was used to find TICs with significant distribution differences among patients with high- and low-risk scores. (b) The Spearman coefficient was applied to detect the correlation between each TIC and the nine-gene signature. Only correlations with *p* value < 0.05 were plotted. (c) The Venn diagram that integrating the results from (a) and (b). TIC: tumor-infiltrating immune cell; ∗*p* value < 0.05; *p* value < 0.05 was considered statistically significant.

After combining the results from the Wilcoxon rank sum test (Figure 9(a)) and Spearman's coefficient (Figure 9(b) and Table S5), we found nine TICs (Figure 9(c)), including macrophage M0, mast cell resting, mast cell activated, T cell CD4 memory resting, neutrophils, dendritic cell resting, dendritic cell activated, T cell CD8, and B cell memory, that were determined to be associated with the nine-gene signature. Among them, macrophage M0, mast cell activated, neutrophils, and dendritic cell activated positively correlated with risk score while the remaining negatively.

**Figure 10:**
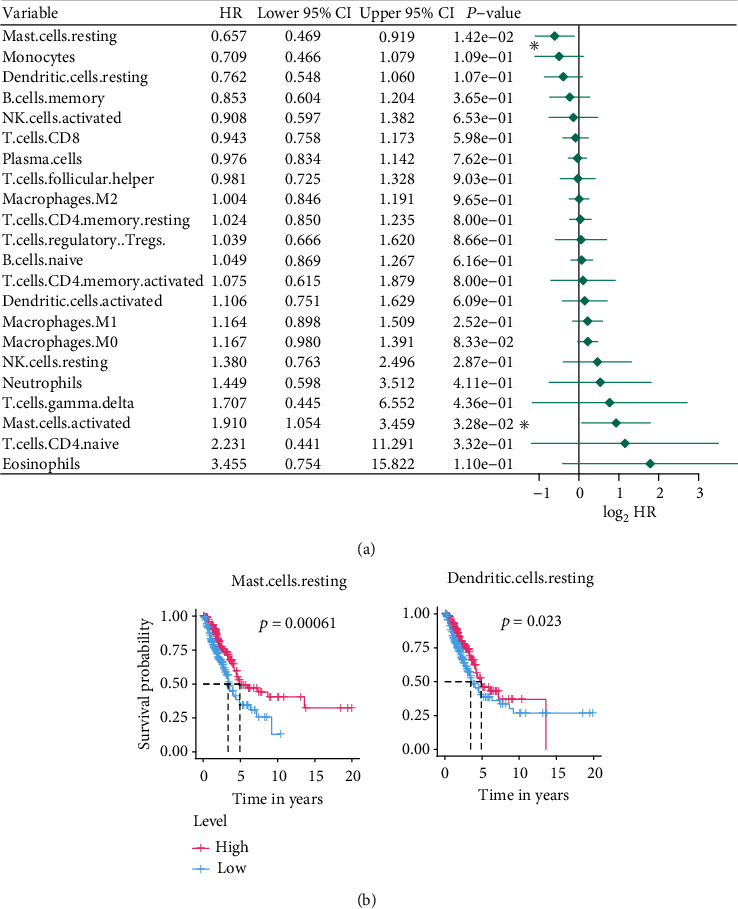
Univariate Cox proportional hazard model (a) and Kaplan-Meier estimator (b) built for evaluating the 22 TICs' prognostic capacities. (a) The asterisks shown specify a *p* value < 0.05. (b) We only showed the Kaplan-Meier estimators with *p* value < 0.05. TIC: tumor-infiltrating immune cell; *p* value < 0.05 was considered statistically significant; LUAD: lung adenocarcinoma.

Also, to confirm each TIC's prognostic ability, the Kaplan-Meier estimator and univariate Cox proportional hazard model were further built. The results of the univariate Cox proportional hazard model shown in Figure 10 confirmed that mast cell resting and mast cell activated can significantly affect the patient's prognosis. The Kaplan-Meier estimator (Table S6) emphasized that mast cell resting and dendritic cell resting can clearly distinguish the survival difference from LUAD patients. In the light of the results of Cox analysis and Kaplan-Meier estimator, mast cell resting has the potential prognostic ability in LUAD.

In view the correlation result of this section, we noticed that mast cell resting was closely related to the gene signature. Furthermore, survival analyses, including Cox and Kaplan-Meier analyses, also confirmed that mast cell resting could predict the LUAD prognosis. Accordingly, it is not difficult to infer that the significant infiltration from mast cell resting may play a vital role in the gene signature's prognostic power in LUAD patients.

## 4. Discussion

In this research, we found an immune-related nine-gene signature for predicting the LUAD prognosis by mining public datasets from the TCGA and GEO. Univariate Cox analysis was adopted to screen the immune-related potential prognosis genes using the data of the TCGA-LUAD dataset. Then, the RSFVH and LASSO models were built on these prognosis genes, and a nine-gene signature was constructed which was associated with LUAD prognosis. Univariate and multivariate Cox proportional hazard model, ROC curves, and Kaplan-Meier estimator were further used to test the prognosis ability of the gene signature in the validation cohorts. The validation results showed that the signature we found strongly predicted LUAD outcomes. The function annotation analysis detailed vital mechanism associated with the signature. TIC results displayed that mast cell resting may act as a backbone for the signature's prognosis ability. Compared with previous studies, our work is innovative. This study incorporated three cohorts, immune-related genes, RSFVH, LASSO, Cox model, Kaplan-Meier estimator, and ROC curve for processing. Our findings will help LUAD's in-depth research.

The signature we have found exhibited stable prognostic ability in all three cohorts. The signature consists of nine genes (Table 2), including BTK, CCR6, S100A10, SEMA3C, GPI, SCG2, TNFRSF11A, CCL20, and DKK1. In our research, BTK and CCR6 were showing favorably influence on LUAD prognosis, while the remaining genes showed adverse effects on the outcome. It has been studied that the expression of BTK is correlated with clinic characteristics (tumor staging and metastasis) negatively and related to the survival of LUAD patients positively, and BTK may be responsible for maintaining the immunodominant state of the tumor microenvironment [23]. CCR6 has been shown to be related to cancerous adrenal that developed lung metastases. However, there is no direct evidence on whether CCR6 in tumors is a prognostic marker for LUAD patients' survival [24, 25]. In many cancers, S100A10 has been demonstrated to play a vital role in promoting tumorigenesis. The overexpression of S100A10 is related to the poor prognosis of lung cancer. Recent studies have determined that S100A10 is one of the three gene expression characteristics that independently predict LUAD survivals [26–28]. The expression of SEMA3C is related to tumor progression, and it has been reported that SEMA3C is directly associated with the poor prognosis of lung cancer, breast cancer, gastric cancer, and ovarian cancer [29, 30]. In many cancers including lung cancer, increased SEMA3C expression is related to unfavorable prognosis and tumor progression [29, 30]. In the past decade, more and more studies have been conducted showing that different GPI-anchored proteins are profoundly involved in many cancers. However, it is not clear how GPI plays a role in the progression and outcome of LUAD [31]. It has been known that TNFRSF11A is related to glioma and breast cancer in existing studies, but its relationship with lung cancer is still unclear [32, 33]. Wang et al. [34] found that IL-1*β* can stimulate lung cancer cells to produce CCL20 by activating the MAPK and PI3K signaling pathways, and the autocrine of CCL20 can promote the migration and proliferation of lung cancer cells by initiating the ERK and PI3K signaling pathways. CCL20 has the potential to become a new therapeutic target for lung cancer [34]. Kimura et al. presented that the cytoskeleton-associated protein 4 of the DKK1 receptor mediates DKK1 signaling to promote cancer cell proliferation through the PI3K/AKT pathway and is correlated with the poor outcomes of lung cancer patients [35]. J. Zhang et al. concluded that DKK1 boosts the invasion and migration of non-small-cell lung cancer through the *β*-catenin signaling pathway [36]. It is worth noting that, according to previous reports, no evidence has been found that SCG2 is related to tumors or LUAD, so it is very likely that SCG2 is a potential new target, which is worthy of further research.

The GSEA results displayed that the gene sets about glycolysis, mTORC1, and MYC were top enriched. Glycolysis is a cytoplasmic pathway which breaks down glucose into two three-carbon compounds and generates energy [37]. Since the German scientist Otto Warburg put forward the “Warburg hypothesis” called the “Warburg effect,” people have known that there is a link between aerobic glycolysis and tumorigenesis for decades [38, 39]. Glycolysis has been proven to induce tumor cell proliferation and metastasis, by stimulating DNA mutation and peroxide production [40, 41]. Lung cancer cells that consume a lot of glucose can interfere or block the nutrient supply of neighboring normal cells [40, 41]. PKM2 was confirmed to be highly expressed and secreted in lung cancer cells and clinical samples [42]. mTOR, a pathway, is seen as dysregulated in many diseases including lung cancer [43, 44]. Studies have shown that inhibiting mTOR signaling can destroy angiogenesis, induce apoptosis and autophagy, and also block tumor cell progression [43, 44]. The activation of mTORC1 can regulate DNA damage, enhance nucleotide synthesis, help protein synthesis, accelerate body metabolism, and promote cell survival [45, 46]. Therefore, targeting mTOR is an attractive and promising strategy for developing therapeutic agents for lung cancer [44]. MYC family oncogenes are dysregulated in more than 50% of human cancers, and this dysregulation is usually associated with poor prognosis and poor patient survival [47]. Recent studies have shown that the expression and function of MYC potentially help develop new cancer treatments [48]. The MYC oncogene is usually amplified in cells grown from lung tumors [49]. Studies have shown that drug-like molecules can inhibit the activation of MYC, thereby causing tumors in the body to be suppressed [48]. The transfection of MYC enhanced the in vitro proliferation rate of human small-cell lung cancer cells [49].

In addition, based on the CIBERSORT algorithm and survival analysis, we uncovered that mast cell resting owns clear correlations with the gene signature and strong prognostic abilities as well, indicating that the infiltration of these cells plays a key role in the gene signature's predictive power. Mast cells are a type of white blood cells, which are found in the connective tissues throughout the body, especially subcutaneous, near blood vessels and lymphatic vessels, nerves, lungs, and intestines [50]. Mast cells contain granules rich in histamine and heparin and are part of the immune and neuroimmune system [50]. Mast cells in peripheral blood also play a role in tumor invasion, proving their role in regulating tumor biology [51]. Crosstalk between mast cells and other tumor-infiltrating cells seems to be a potential target for anticancer therapy [51]. The increase in mast cells in the tumor environment is associated with poor prognosis, increased metastasis, and reduced survival rates for several human cancers [51]. Welsh et al. reported that in non-small-cell lung adenocarcinoma, the number of mast cells in the tumor stroma has nothing to do with tumor progression, while the increase in the number of mast cells in the islets of tumor cells is associated with a favorable prognosis [52]. Reducing mast cells number is a therapeutic approach in macrocytosis and other diseases in which mast cells' number is increased [53, 54]. Mast cells might act as a new target for the adjuvant treatment of tumors through the selective inhibition of angiogenesis, tissue remodeling, and tumor-promoting molecules, permitting the secretion of cytotoxic cytokines and preventing mast cell-mediated immune suppression [53–56]. According to the findings of our work, mast cell resting has a promising potential to target the nine-gene signature and LUAD therapy. However, more efforts are needed to study these immune cells.

In the end, we must clarify the limitations of this research. The signature we derived was from retrospective data. We believe that more prospective data can make our results more effective and rigorous. In addition, although it has been validated in two independent cohorts, its proof was derived from the analysis results of public databases. There is still no wet laboratory data to explain and support the prognostic ability of these 9 genes and their role in immune infiltration. Therefore, ongoing research is needed to reveal more evidence to for the nine-gene signature's promising future.

## 5. Conclusion

This study has discovered a novel and powerful immune-related nine-gene signature that can predict the prognosis of LUAD. We validated the signature's stableness and applicability via examining it in other two GEO cohorts. More importantly, the key role of mast cell resting was identified, which may help the signature in prognostic ability. Our work potentially advances a new LUAD treatment discovery.

## Figures and Tables

**Table 1:** Clinical characteristics of patients involved in the study.

Characteristics	Training cohort (TCGA-LUAD, 500 cases)	Validation cohort (GSE72094, 442 cases)	Validation cohort (GSE68465, 443 cases)
Age			
<65	219 (43.8%)	115 (26.02%)	214 (48.31%)
≥65	271 (54.2%)	306 (69.23%)	229 (51.69%)
Unknown	10 (2%)	21 (4.75%)	0
Gender			
Female	270 (54%)	240 (54.3%)	220 (49.66%)
Male	230 (46%)	202 (45.7%)	223 (50.34%)
T classification			
T1	167 (33.4%)	NA	150 (33.86%)
T2	267 (53.4%)	NA	251 (56.66%)
T3	45 (9%)	NA	28 (6.32%)
T4	18 (3.6%)	NA	12 (2.71%)
Unknown	3 (0.6%)	NA	2 (0.45%)
N classification			
N0	324 (64.8%)	NA	299 (67.49%)
N1	94 (18.8%)	NA	88 (19.86%)
N2	69 (13.8%)	NA	53 (11.96%)
N3	2 (0.4%)	NA	0
Unknown	11 (2.2%)	NA	3 (0.68%)
M classification			
M0	332 (66.4%)	NA	NA
M1	24 (4.8%)	NA	NA
Unknown	144 (28.8%)	NA	NA
Tumor stage			
Stage I	268 (53.6%)	265 (59.95%)	NA
Stage II	119 (23.8%)	69 (15.61%)	NA
Stage III	80 (16%)	63 (14.25%)	NA
Stage IV	25 (5%)	17 (3.85%)	NA
Unknown	8 (1.6%)	28 (6.33%)	NA
Race			
White	386 (77.2%)	399 (90.27%)	295 (66.59%)
Black or African American	52 (10.4%)	13 (2.94%)	12 (2.71%)
American Indian or Alaska native	1 (0.2%)	0	1 (0.23%)
Asian	7 (1.4%)	3 (0.68%)	6 (1.35%)
Unknown	54 (10.8%)	27 (6.11%)	129 (29.12%)
Ethnicity			
Hispanic or Latino	7 (1.4%)	10 (2.26%)	NA
Not Hispanic or Latino	381 (76.2%)	402 (90.95%)	NA
Unknown	112 (22.4%)	30 (6.79%)	NA
Vital status			
Alive	318 (63.6%)	298 (67.42%)	207 (46.73%)
Dead	182 (36.4%)	122 (27.6%)	236 (53.27%)
Unknown	0	22 (4.98%)	0

**Table 2:** Immune-related prognostic genes with risk coefficient obtained from LASSO Cox regression model.

Gene symbol	Description	Risk coefficient	Category
BTK	Bruton tyrosine kinase	-0.219628874	BCR signaling pathway
CCR6	C-C motif chemokine receptor 6	-0.176444716	Antimicrobials, Chemokine_Receptors, and Cytokine_Receptors
S100A10	S100 calcium binding protein A10	0.109696741	Antimicrobials
SEMA3C	Semaphorin 3C	0.11695307	Chemokines and cytokines
GPI	Glucose-6-phosphate isomerase	0.132449603	Cytokines
SCG2	Secretogranin II	0.188369623	Cytokines
TNFRSF11A	TNF receptor superfamily member 11a	0.229854137	Cytokine_Receptors and TNF_Family_Members_Receptors
CCL20	C-C motif chemokine ligand 20	0.272150603	Antimicrobials, chemokines, and cytokines
DKK1	Dickkopf WNT signaling pathway inhibitor 1	0.339588505	Cytokines

## Data Availability

This study used the following publicly available datasets: TCGA (https://portal.gdc.cancer.gov/) and GEO (https://www.ncbi.nlm.nih.gov/geo/).

## Supplementary Materials

Supplementary Materials Figure S1: the overall distributions of the risk score (upper), survival status (middle), and gene expression (bottom) of the nine-gene signature, based on the data of the training (A) and validation (B and C) cohorts and five-year period. Figure S2: Kaplan-Meier estimator built for evaluating the prognosis capacity of the nine-gene signature using the data of the training (A) and validation (B and C) cohorts and five-year period. The bottom part signifies the number of patients at risk. The two-sided log-rank test measured the differences between the high- and low-risk patients with a *p* value < 0.05.Figure S3: overall profiles and correlations of the 22 TICs in LUAD patients, construed using the data from the training cohort. (A) The profile distribution of 22 TICs in high- and low-risk LUADs, displayed in the form of bar plots. (B) The inner correlations between 22 TICs, shown in the form of heat map. TIC: tumor-infiltrating immune cell; LUAD: lung adenocarcinoma; *p* value < 0.05 was considered statistically significant. Table S1: 1793 unique immune-related genes downloaded from the ImmPort. Table S2: 267 prognostic immune-related genes identified by univariate Cox analysis in the training cohort. Table S3: top 1000 risk models screened by Kaplan-Meier analysis. Table S4: Enriched gene sets of HALLMARK collection (∣NES | >1, NOM *p* value < 0.05, and FDR *q* value < 0.25). Table S5: the Spearman's rank correlation coefficient tests the correlation between each TIC and the gene signature. Table S6: the Kaplan-Meier estimator detects the prognostic ability of each TIC.
