# Supplementary material for: A Novel Immune-Related Gene Signature Predicts Prognosis of Lung Adenocarcinoma
Source: Biomed Res Int. 2022 Apr 9;2022:4995874. doi: 10.1155/2022/4995874 (PMC9013292; doi:10.1155/2022/4995874)
Supplement: Supplementary Materials — Figure S1: the overall distributions of the risk score (upper), survival status (middle), and gene expression (bottom) of the nine-gene signature, based on the data of the training (A) and validation (B and C) cohorts and five-year period. Figure S2: Kaplan-Meier estimator built for evaluating the prognosis capacity of the nine-gene signature using the data of the training (A) and validation (B and C) cohorts and five-year period. The bottom part signifies the number of patients at risk. The two-sided log-rank test measured the differences between the high- and low-risk patients with a p value < 0.05.Figure S3: overall profiles and correlations of the 22 TICs in LUAD patients, construed using the data from the training cohort. (A) The profile distribution of 22 TICs in high- and low-risk LUADs, displayed in the form of bar plots. (B) The inner correlations between 22 TICs, shown in the form of heat map. TIC: tumor-infiltrating immune cell; LUAD: lung adenocarcinoma; p value < 0.05 was considered statistically significant. Table S1: 1793 unique immune-related genes downloaded from the ImmPort. Table S2: 267 prognostic immune-related genes identified by univariate Cox analysis in the training cohort. Table S3: top 1000 risk models screened by Kaplan-Meier analysis. Table S4: Enriched gene sets of HALLMARK collection (∣NES | >1, NOM p value < 0.05, and FDR q value < 0.25). Table S5: the Spearman's rank correlation coefficient tests the correlation between each TIC and the gene signature. Table S6: the Kaplan-Meier estimator detects the prognostic ability of each TIC. [file 4995874.f1.pdf]

Figure S1

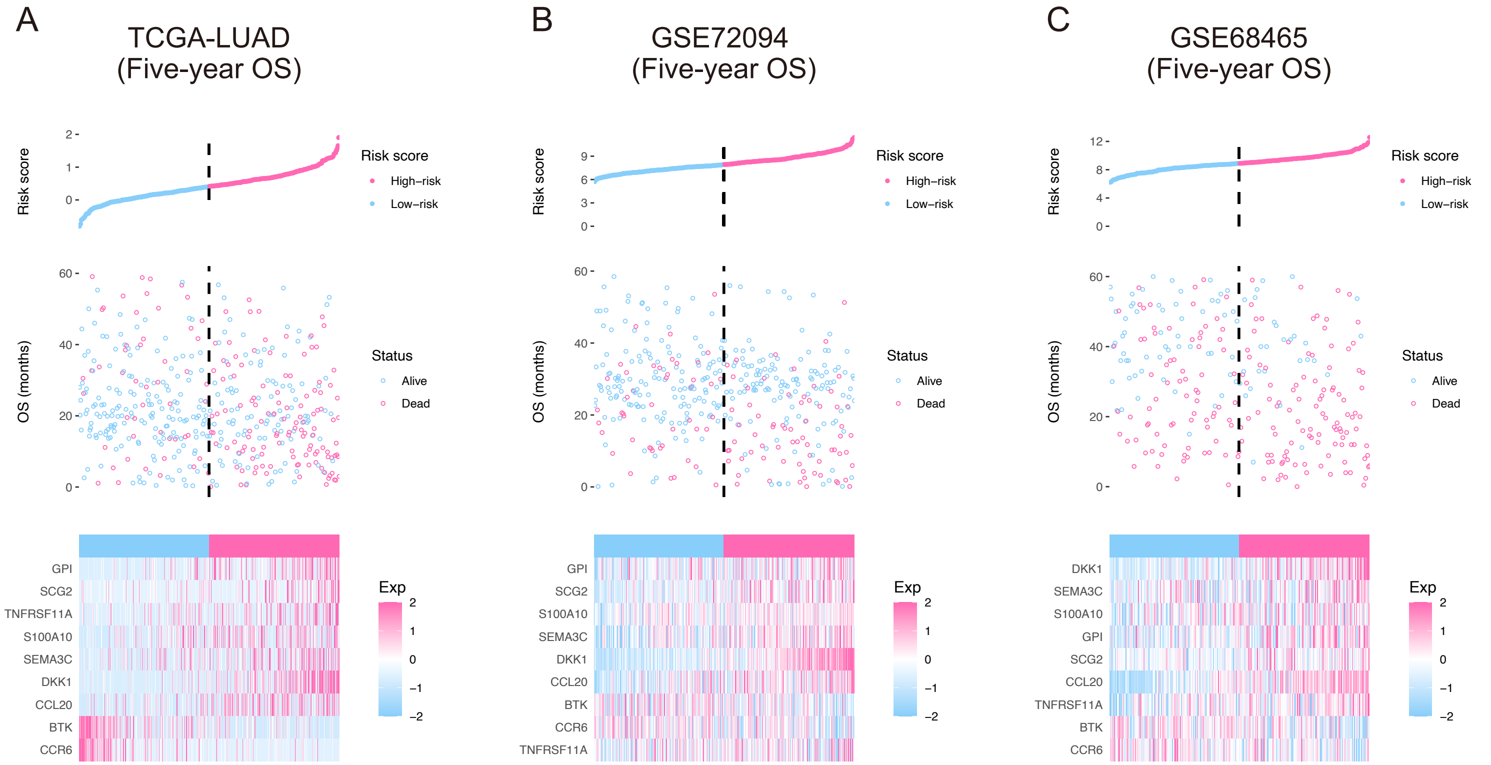
A
TCGA-LUAD
(Five-year OS)
B
GSE72094
(Five-year OS)
C
GSE68465
(Five-year OS)
Risk score
High−risk
Low−risk
OS (months)
Status
Alive
Dead
Exp
GPI
SCG2
TNFRSF11A
S100A10
SEMA3C
DKK1
CCL20
BTK
CCR6

Figure S2

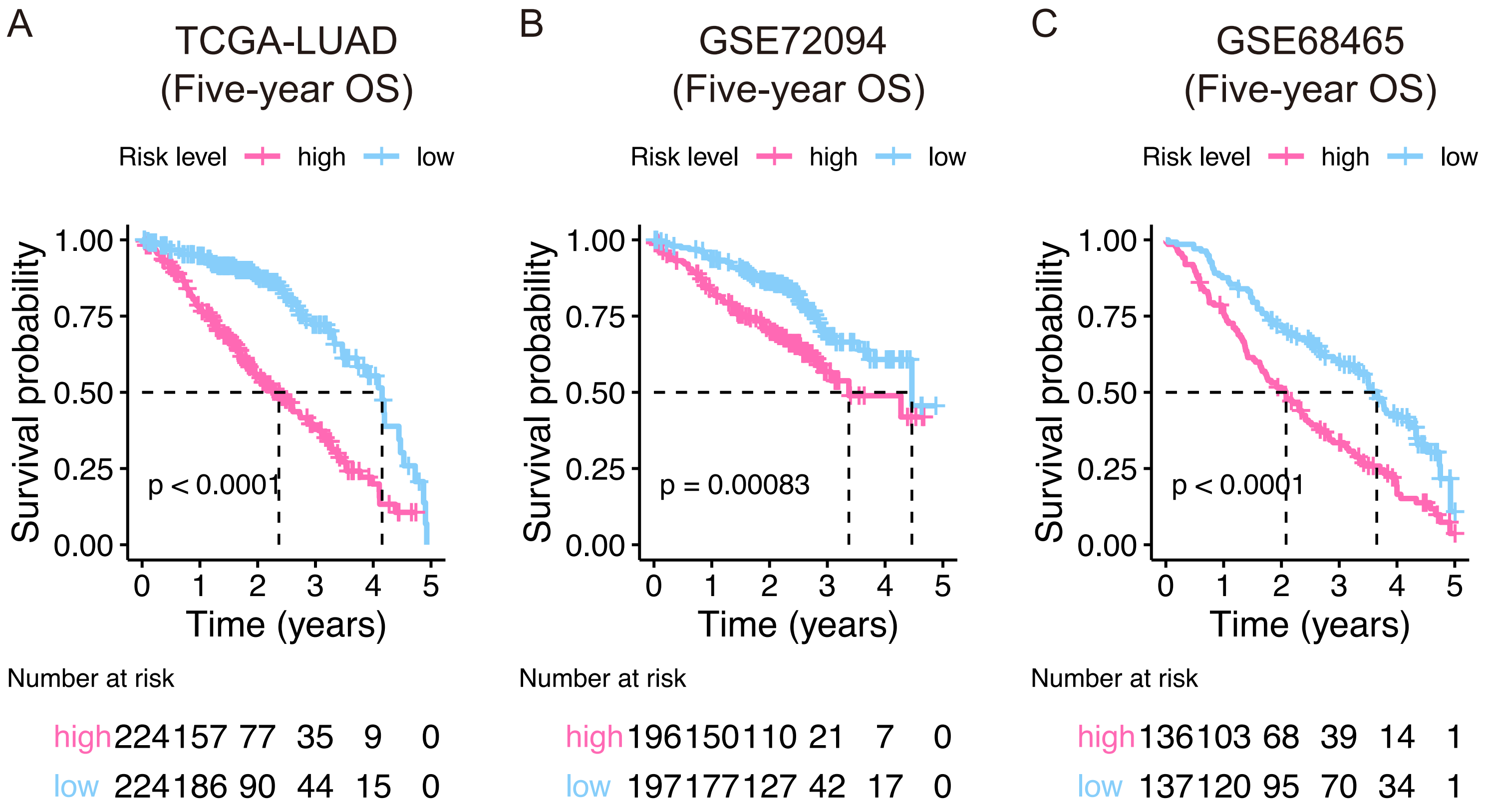
A
TCGA-LUAD
(Five-year OS)
Risk level high low
Survival probability
1.00
0.75
0.50
0.25
0.00
p < 0.0001
0 1 2 3 4 5
Time (years)
Number at risk
high 224 157 77 35 9 0
low 224 186 90 44 15 0
B
GSE72094
(Five-year OS)
Risk level high low
Survival probability
p = 0.00083
Time (years)
Number at risk
high 196 150 110 21 7 0
low 197 177 127 42 17 0
C
GSE68465
(Five-year OS)
Risk level high low
Survival probability
p < 0.0001
Time (years)
Number at risk
high 136 103 68 39 14 1
low 137 120 95 70 34 1

Figure S3

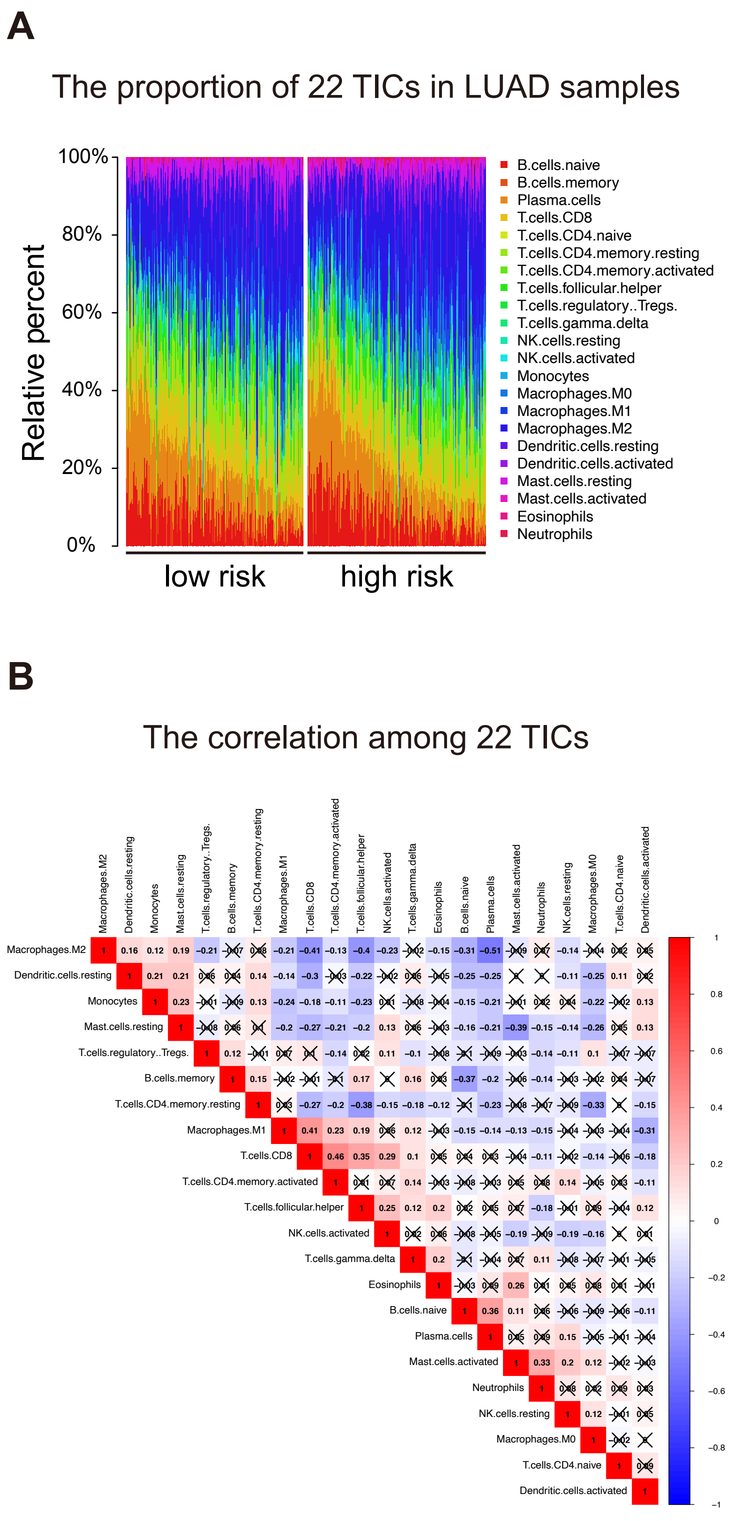
A
The proportion of 22 TICs in LUAD samples
Relative percent
100%
80%
60%
40%
20%
0%
low risk
high risk
B.cells.naive
B.cells.memory
Plasma.cells
T.cells.CD8
T.cells.CD4.naive
T.cells.CD4.memory.resting
T.cells.CD4.memory.activated
T.cells.follicular.helper
T.cells.regulatory..Tregs.
T.cells.gamma.delta
NK.cells.resting
NK.cells.activated
Monocytes
Macrophages.M0
Macrophages.M1
Macrophages.M2
Dendritic.cells.resting
Dendritic.cells.activated
Mast.cells.resting
Mast.cells.activated
Eosinophils
Neutrophils
B
The correlation among 22 TICs
Macrophages.M2
Dendritic.cells.resting
Monocytes
Mast.cells.resting
T.cells.regulatory..Tregs.
B.cells.memory
T.cells.CD4.memory.resting
Macrophages.M1
T.cells.CD8
T.cells.CD4.memory.activated
T.cells.follicular.helper
NK.cells.activated
T.cells.gamma.delta
Eosinophils
B.cells.naive
Plasma.cells
Mast.cells.activated
Neutrophils
NK.cells.resting
Macrophages.M0
T.cells.CD4.naive
Dendritic.cells.activated
1
0.8
0.6
0.4
0.2
0
−0.2
−0.4
−0.6
−0.8
−1

Table S1. 1793 unique immune-related genes downloaded from the ImmPort.

Symbol	Name	Chromosome	Category
A2M	alpha-2-macroglobulin	12	Antimicrobials
ABCC4	ATP binding cassette subfamily C member 4	13	Antimicrobials
ACKR1	atypical chemokine receptor 1 (Duffy blood group)	1	Chemokine_Receptors
ACKR2	atypical chemokine receptor 2	3	Antimicrobials
ACKR3	atypical chemokine receptor 3	2	Chemokine_Receptors
ACKR4	atypical chemokine receptor 4	3	Antimicrobials
ACO1	aconitase 1	9	Antimicrobials
ACTA1	actin alpha 1, skeletal muscle	1	Antimicrobials
ACTG1	actin gamma 1	17	Antimicrobials
ACVR1B	activin A receptor type 1B	12	Cytokine_Receptors
ACVR1C	activin A receptor type 1C	2	Cytokine_Receptors
ACVR2A	activin A receptor type 2A	2	Cytokine_Receptors
ACVR2B	activin A receptor type 2B	3	Cytokine_Receptors
ACVRL1	activin A receptor like type 1	12	Cytokine_Receptors
ADA2	adenosine deaminase 2	22	Cytokines
ADAR	adenosine deaminase RNA specific	1	Antimicrobials
ADCYAP1R1	ADCYAP receptor type I	7	Cytokine_Receptors
ADIPOQ	adiponectin, C1Q and collagen domain containing	3	Antimicrobials
ADIPOR1	adiponectin receptor 1	1	Cytokine_Receptors
ADIPOR2	adiponectin receptor 2	12	Cytokine_Receptors
ADM	adrenomedullin	11	Cytokines
ADM2	adrenomedullin 2	22	Cytokines
ADRB1	adrenoceptor beta 1	10	Cytokine_Receptors
ADRB2	adrenoceptor beta 2	5	Cytokine_Receptors
ADRM1	adhesion regulating molecule 1	20	Antigen_Processing_and_Presentation
AEN	apoptosis enhancing nuclease	15	Antimicrobials
AGER	advanced glycosylation end-product specific receptor	6	Antimicrobials
AGRP	agouti related neuropeptide	16	Cytokines
AGT	angiotensinogen	1	Cytokines

AGTR1	angiotensin II receptor type 1	3	Cytokine_Receptors
AGTR2	angiotensin II receptor type 2	X	Cytokine_Receptors
AHNAK	AHNAK nucleoprotein	11	Antimicrobials
AIMP1	aminoacyl tRNA synthetase complex interacting multifunctional protein 1	4	Cytokines
AKT1	AKT serine/threonine kinase 1	14	BCRSignalingPathway
AKT2	AKT serine/threonine kinase 2	19	BCRSignalingPathway
AKT3	AKT serine/threonine kinase 3	1	BCRSignalingPathway
ALB	albumin	4	Antimicrobials
AMBN	ameloblastin	4	Cytokines
AMELX	amelogenin X-linked	X	Cytokines
AMH	anti-Mullerian hormone	19	Cytokines
AMHR2	anti-Mullerian hormone receptor type 2	12	Cytokine_Receptors
ANGPT1	angiopoietin 1	8	Cytokine_Receptors
ANGPT4	angiopoietin 4	20	Cytokine_Receptors
ANGPTL1	angiopoietin like 1	1	Cytokine_Receptors
ANGPTL2	angiopoietin like 2	9	Cytokine_Receptors
ANGPTL3	angiopoietin like 3	1	Cytokine_Receptors
ANGPTL4	angiopoietin like 4	19	Cytokine_Receptors
ANGPTL5	angiopoietin like 5	11	Cytokines
ANGPTL6	angiopoietin like 6	19	Cytokine_Receptors
ANGPTL7	angiopoietin like 7	1	Cytokines
ANOS1	anosmin 1	X	Antimicrobials
ANXA6	annexin A6	5	Antimicrobials
AP3B1	adaptor related protein complex 3 subunit beta 1	5	Antigen_Processing_and_Presentation
APLN	apelin	X	Cytokines
APLNR	apelin receptor	11	Cytokine_Receptors
APOBEC3A	apolipoprotein B mRNA editing enzyme catalytic subunit 3A	22	Antimicrobials
APOBEC3C	apolipoprotein B mRNA editing enzyme catalytic subunit 3C	22	Antimicrobials
APOBEC3F	apolipoprotein B mRNA editing enzyme catalytic subunit 3F	22	Antimicrobials
APOBEC3G	apolipoprotein B mRNA editing enzyme catalytic subunit 3G	22	Antimicrobials

APOBEC3H	apolipoprotein B mRNA editing enzyme catalytic subunit 3H	22	Antimicrobials
APOD	apolipoprotein D	3	Antimicrobials
APOH	apolipoprotein H	17	Antimicrobials
APOM	apolipoprotein M	6	Antimicrobials
AQP9	aquaporin 9	15	Antimicrobials
AR	androgen receptor	X	Cytokine_Receptors
ARAF	A-Raf proto-oncogene, serine/threonine kinase	X	NaturalKiller_Cell_Cytotoxicity
AREG	amphiregulin	4	Cytokines
ARG2	arginase 2	14	Antimicrobials
ARRB1	arrestin beta 1	11	Antimicrobials
ARTN	artemin	1	Cytokines
AVP	arginine vasopressin	20	Cytokines
AVPR1A	arginine vasopressin receptor 1A	12	Cytokine_Receptors
AVPR1B	arginine vasopressin receptor 1B	1	Cytokine_Receptors
AVPR2	arginine vasopressin receptor 2	X	Cytokine_Receptors
AZGP1	alpha-2-glycoprotein 1, zinc-binding	7	Antigen_Processing_and_Presentation
AZU1	azurocidin 1	19	Antimicrobials
B2M	beta-2-microglobulin	15	Antigen_Processing_and_Presentation
BACH2	BTB domain and CNC homolog 2	6	Antimicrobials
BCL10	BCL10 immune signaling adaptor	1	BCRSignalingPathway
BCL3	BCL3 transcription coactivator	19	Antimicrobials
BDNF	brain derived neurotrophic factor	11	Cytokines
BECN1	beclin 1	17	Antimicrobials
BID	BH3 interacting domain death agonist	22	NaturalKiller_Cell_Cytotoxicity
BIRC5	baculoviral IAP repeat containing 5	17	Antimicrobials
BLNK	B cell linker	10	BCRSignalingPathway
BMP1	bone morphogenetic protein 1	8	Cytokines
BMP10	bone morphogenetic protein 10	2	Cytokines
BMP15	bone morphogenetic protein 15	X	Cytokines
BMP2	bone morphogenetic protein 2	20	Cytokines

BMP3	bone morphogenetic protein 3	4	Cytokines
BMP4	bone morphogenetic protein 4	14	Cytokines
BMP5	bone morphogenetic protein 5	6	Cytokines
BMP6	bone morphogenetic protein 6	6	Cytokines
BMP7	bone morphogenetic protein 7	20	Cytokines
BMP8A	bone morphogenetic protein 8a	1	Cytokines
BMP8B	bone morphogenetic protein 8b	1	Cytokines
BMPR1A	bone morphogenetic protein receptor type 1A	10	Cytokine_Receptors
BMPR1B	bone morphogenetic protein receptor type 1B	4	Cytokine_Receptors
BMPR2	bone morphogenetic protein receptor type 2	2	Cytokine_Receptors
BPHL	biphenyl hydrolase like	6	Antimicrobials
BPI	bactericidal permeability increasing protein	20	Antimicrobials
BPIFA1	BPI fold containing family A member 1	20	Antimicrobials
BPIFA2	BPI fold containing family A member 2	20	Antimicrobials
BPIFA3	BPI fold containing family A member 3	20	Antimicrobials
BPIFB1	BPI fold containing family B member 1	20	Antimicrobials
BPIFB2	BPI fold containing family B member 2	20	Antimicrobials
BPIFB3	BPI fold containing family B member 3	20	Antimicrobials
BPIFB4	BPI fold containing family B member 4	20	Antimicrobials
BPIFB6	BPI fold containing family B member 6	20	Antimicrobials
BPIFC	BPI fold containing family C	22	Antimicrobials
BRAF	B-Raf proto-oncogene, serine/threonine kinase	7	NaturalKiller_Cell_Cytotoxicity
BRD8	bromodomain containing 8	5	Cytokine_Receptors
BST2	bone marrow stromal cell antigen 2	19	Antimicrobials
BTC	betacellulin	4	Cytokines
BTK	Bruton tyrosine kinase	X	BCRSignalingPathway
C3	complement C3	19	Chemokines
C3AR1	complement C3a receptor 1	12	Cytokine_Receptors
C5	complement C5	9	Chemokines
C5AR1	complement C5a receptor 1	19	Chemokine_Receptors
C5AR2	complement component 5a receptor 2	19	Chemokine_Receptors
C8G	complement C8 gamma chain	9	Antimicrobials

CACYBP	calcyclin binding protein	1	Antimicrobials
CALCA	calcitonin related polypeptide alpha	11	Cytokines
CALCB	calcitonin related polypeptide beta	11	Cytokines
CALCR	calcitonin receptor	7	Cytokine_Receptors
CALCRL	calcitonin receptor like receptor	2	Cytokine_Receptors
CALR	calreticulin	19	Antigen_Processing_and_Presentation
CAMP	cathelicidin antimicrobial peptide	3	Antimicrobials
CANX	calnexin	5	Antigen_Processing_and_Presentation
CARD11	caspase recruitment domain family member 11	7	BCRSignalingPathway
CASP3	caspase 3	4	NaturalKiller_Cell_Cytotoxicity
CAT	catalase	11	Cytokines
CBL	Cbl proto-oncogene	11	TCRsignalingPathway
CBLB	Cbl proto-oncogene B	3	TCRsignalingPathway
CBLC	Cbl proto-oncogene C	19	TCRsignalingPathway
CCK	cholecystokinin	3	Cytokines
CCL1	C-C motif chemokine ligand 1	17	Antimicrobials
CCL11	C-C motif chemokine ligand 11	17	Antimicrobials
CCL13	C-C motif chemokine ligand 13	17	Antimicrobials
CCL14	C-C motif chemokine ligand 14	17	Antimicrobials
CCL15	C-C motif chemokine ligand 15	17	Antimicrobials
CCL15-CCL14	CCL15-CCL14 readthrough (NMD candidate)	17	Antimicrobials
CCL16	C-C motif chemokine ligand 16	17	Antimicrobials
CCL17	C-C motif chemokine ligand 17	16	Antimicrobials
CCL18	C-C motif chemokine ligand 18	17	Antimicrobials
CCL19	C-C motif chemokine ligand 19	9	Antimicrobials
CCL2	C-C motif chemokine ligand 2	17	Antimicrobials
CCL20	C-C motif chemokine ligand 20	2	Antimicrobials
CCL21	C-C motif chemokine ligand 21	9	Antimicrobials
CCL22	C-C motif chemokine ligand 22	16	Antimicrobials

CCL23	C-C motif chemokine ligand 23	17	Antimicrobials
CCL24	C-C motif chemokine ligand 24	7	Antimicrobials
CCL25	C-C motif chemokine ligand 25	19	Antimicrobials
CCL26	C-C motif chemokine ligand 26	7	Antimicrobials
CCL27	C-C motif chemokine ligand 27	9	Antimicrobials
CCL28	C-C motif chemokine ligand 28	5	Antimicrobials
CCL3	C-C motif chemokine ligand 3	17	Antimicrobials
CCL3L1	C-C motif chemokine ligand 3 like 1	17	Antimicrobials
CCL3L3	C-C motif chemokine ligand 3 like 3	17	Antimicrobials
CCL3P1	C-C motif chemokine ligand 3 pseudogene 1	17	Chemokines
CCL4	C-C motif chemokine ligand 4	17	Antimicrobials
CCL4L1	C-C motif chemokine ligand 4 like 1	17	Antimicrobials
CCL4L2	C-C motif chemokine ligand 4 like 2	17	Antimicrobials
CCL5	C-C motif chemokine ligand 5	17	Antimicrobials
CCL7	C-C motif chemokine ligand 7	17	Antimicrobials
CCL8	C-C motif chemokine ligand 8	17	Antimicrobials
CCN1	cellular communication network factor 1	1	Chemokines
CCN2	cellular communication network factor 2	6	Cytokines
CCN3	cellular communication network factor 3	8	Cytokines
CCR1	C-C motif chemokine receptor 1	3	Antimicrobials
CCR10	C-C motif chemokine receptor 10	17	Antimicrobials
CCR3	C-C motif chemokine receptor 3	3	Antimicrobials
CCR4	C-C motif chemokine receptor 4	3	Antimicrobials
CCR5	C-C motif chemokine receptor 5	3	Antimicrobials
CCR6	C-C motif chemokine receptor 6	6	Antimicrobials
CCR7	C-C motif chemokine receptor 7	17	Antimicrobials
CCR8	C-C motif chemokine receptor 8	3	Antimicrobials
CCR9	C-C motif chemokine receptor 9	3	Chemokine_Receptors
CCRL2	C-C motif chemokine receptor like 2	3	Chemokine_Receptors
CD14	CD14 molecule	5	Antimicrobials
CD19	CD19 molecule	16	BCRSignalingPathway
CD1A	CD1a molecule	1	Antigen_Processing_and_Presenta

			tion
CD1B	CD1b molecule	1	Antigen_Processing_and_Presentation
CD1C	CD1c molecule	1	Antigen_Processing_and_Presentation
CD1D	CD1d molecule	1	Antigen_Processing_and_Presentation
CD1E	CD1e molecule	1	Antigen_Processing_and_Presentation
CD209	CD209 molecule	19	Antigen_Processing_and_Presentation
CD22	CD22 molecule	19	BCRSignalingPathway
CD244	CD244 molecule	1	NaturalKiller_Cell_Cytotoxicity
CD247	CD247 molecule	1	NaturalKiller_Cell_Cytotoxicity
CD28	CD28 molecule	2	TCRsignalingPathway
CD320	CD320 molecule	19	Cytokines
CD3D	CD3d molecule	11	TCRsignalingPathway
CD3E	CD3e molecule	11	TCRsignalingPathway
CD3G	CD3g molecule	11	TCRsignalingPathway
CD4	CD4 molecule	12	Antigen_Processing_and_Presentation
CD40	CD40 molecule	20	Antimicrobials
CD40LG	CD40 ligand	X	Antimicrobials
CD48	CD48 molecule	1	NaturalKiller_Cell_Cytotoxicity
CD70	CD70 molecule	19	Cytokines
CD72	CD72 molecule	9	BCRSignalingPathway
CD74	CD74 molecule	5	Antigen_Processing_and_Presentation
CD79A	CD79a molecule	19	BCRSignalingPathway
CD79B	CD79b molecule	17	BCRSignalingPathway
CD81	CD81 molecule	11	Antimicrobials
CD86	CD86 molecule	3	Antimicrobials

CD8A	CD8a molecule	2	Antigen_Processing_and_Presentation
CD8B	CD8b molecule	2	Antigen_Processing_and_Presentation
CDC42	cell division cycle 42	1	TCRsignalingPathway
CDH1	cadherin 1	16	Antimicrobials
CDK4	cyclin dependent kinase 4	12	TCRsignalingPathway
CDNF	cerebral dopamine neurotrophic factor	10	Cytokines
CELA1	chymotrypsin like elastase 1	12	Antimicrobials
CER1	cerberus 1, DAN family BMP antagonist	9	Cytokines
CETP	cholesteryl ester transfer protein	16	Antimicrobials
CGA	glycoprotein hormones, alpha polypeptide	6	Cytokines
CGB1	chorionic gonadotropin subunit beta 1	19	Cytokines
CGB2	chorionic gonadotropin subunit beta 2	19	Cytokines
CGB3	chorionic gonadotropin subunit beta 3	19	Cytokines
CGB5	chorionic gonadotropin subunit beta 5	19	Cytokines
CGB7	chorionic gonadotropin subunit beta 7	19	Cytokines
CGB8	chorionic gonadotropin subunit beta 8	19	Cytokines
CHGA	chromogranin A	14	Cytokines
CHGB	chromogranin B	20	Cytokines
CHIT1	chitinase 1	1	Antimicrobials
CHP1	calcineurin like EF-hand protein 1	15	BCRSignalingPathway
CHP2	calcineurin like EF-hand protein 2	16	BCRSignalingPathway
CHUK	component of inhibitor of nuclear factor kappa B kinase complex	10	BCRSignalingPathway
CIITA	class II major histocompatibility complex transactivator	16	Antigen_Processing_and_Presentation
CKLF	chemokine like factor	16	Chemokines
CLCF1	cardiotrophin like cytokine factor 1	11	Cytokines
CLDN4	claudin 4	7	Antimicrobials
CLEC11A	C-type lectin domain containing 11A	19	Cytokines
CLEC4M	C-type lectin domain family 4 member M	19	Antigen_Processing_and_Presentation

CMA1	chymase 1	14	Chemokines
CMKLR1	chemerin chemokine-like receptor 1	12	Chemokine_Receptors
CMTM1	CKLF like MARVEL transmembrane domain containing 1	16	Cytokines
CMTM2	CKLF like MARVEL transmembrane domain containing 2	16	Cytokines
CMTM3	CKLF like MARVEL transmembrane domain containing 3	16	Cytokines
CMTM4	CKLF like MARVEL transmembrane domain containing 4	16	Cytokines
CMTM5	CKLF like MARVEL transmembrane domain containing 5	14	Cytokines
CMTM6	CKLF like MARVEL transmembrane domain containing 6	3	Cytokines
CMTM7	CKLF like MARVEL transmembrane domain containing 7	3	Cytokines
CMTM8	CKLF like MARVEL transmembrane domain containing 8	3	Cytokines
CNTF	ciliary neurotrophic factor	11	Cytokines
CNTFR	ciliary neurotrophic factor receptor	9	Cytokine_Receptors
COLEC10	collectin subfamily member 10	8	Antimicrobials
COLEC12	collectin subfamily member 12	18	Antimicrobials
CORT	cortistatin	1	Cytokines
CR2	complement C3d receptor 2	1	BCRSignalingPathway
CRABP1	cellular retinoic acid binding protein 1	15	Antimicrobials
CRABP2	cellular retinoic acid binding protein 2	1	Antimicrobials
CREB1	cAMP responsive element binding protein 1	2	Antigen_Processing_and_Presentation
CRH	corticotropin releasing hormone	8	Cytokines
CRHR1	corticotropin releasing hormone receptor 1	17	Cytokine_Receptors
CRHR2	corticotropin releasing hormone receptor 2	7	Cytokine_Receptors
CRIM1	cysteine rich transmembrane BMP regulator 1	2	Cytokine_Receptors
CRLF1	cytokine receptor like factor 1	19	Cytokine_Receptors
CRLF2	cytokine receptor like factor 2	X|Y	Cytokine_Receptors
CRLF3	cytokine receptor like factor 3	17	Cytokine_Receptors
CRP	C-reactive protein	1	Antimicrobials
CSF1	colony stimulating factor 1	1	Cytokines
CSF1R	colony stimulating factor 1 receptor	5	Cytokine_Receptors
CSF2	colony stimulating factor 2	5	Cytokines
CSF2RA	colony stimulating factor 2 receptor subunit alpha	X|Y	Cytokine_Receptors

CSF2RB	colony stimulating factor 2 receptor subunit beta	22	Cytokine_Receptors
CSF3	colony stimulating factor 3	17	Cytokines
CSF3R	colony stimulating factor 3 receptor	1	Cytokine_Receptors
CSH1	chorionic somatomammotropin hormone 1	17	Cytokines
CSH2	chorionic somatomammotropin hormone 2	17	Cytokines
CSHL1	chorionic somatomammotropin hormone like 1	17	Cytokines
CSK	C-terminal Src kinase	15	Antimicrobials
CSPG5	chondroitin sulfate proteoglycan 5	3	Cytokines
CSRP1	cysteine and glycine rich protein 1	1	Antimicrobials
CST4	cystatin S	20	Antimicrobials
CTF1	cardiotrophin 1	16	Cytokines
CTLA4	cytotoxic T-lymphocyte associated protein 4	2	TCRsignalingPathway
CTSB	cathepsin B	8	Antigen_Processing_and_Presentation
CTSE	cathepsin E	1	Antigen_Processing_and_Presentation
CTSG	cathepsin G	14	Antimicrobials
CTSL	cathepsin L	9	Antigen_Processing_and_Presentation
CTSS	cathepsin S	1	Antigen_Processing_and_Presentation
CX3CL1	C-X3-C motif chemokine ligand 1	16	Chemokines
CX3CR1	C-X3-C motif chemokine receptor 1	3	Chemokine_Receptors
CXCL1	C-X-C motif chemokine ligand 1	4	Antimicrobials
CXCL10	C-X-C motif chemokine ligand 10	4	Antimicrobials
CXCL11	C-X-C motif chemokine ligand 11	4	Antimicrobials
CXCL12	C-X-C motif chemokine ligand 12	10	Antimicrobials
CXCL13	C-X-C motif chemokine ligand 13	4	Antimicrobials
CXCL14	C-X-C motif chemokine ligand 14	5	Antimicrobials
CXCL16	C-X-C motif chemokine ligand 16	17	Antimicrobials
CXCL17	C-X-C motif chemokine ligand 17	19	Chemokines
CXCL2	C-X-C motif chemokine ligand 2	4	Antimicrobials

CXCL3	C-X-C motif chemokine ligand 3	4	Antimicrobials
CXCL5	C-X-C motif chemokine ligand 5	4	Antimicrobials
CXCL6	C-X-C motif chemokine ligand 6	4	Antimicrobials
CXCL8	C-X-C motif chemokine ligand 8	4	Antimicrobials
CXCL9	C-X-C motif chemokine ligand 9	4	Antimicrobials
CXCR1	C-X-C motif chemokine receptor 1	2	Antimicrobials
CXCR2	C-X-C motif chemokine receptor 2	2	Chemokine_Receptors
CXCR3	C-X-C motif chemokine receptor 3	X	Chemokine_Receptors
CXCR4	C-X-C motif chemokine receptor 4	2	Antimicrobials
CXCR5	C-X-C motif chemokine receptor 5	11	Chemokine_Receptors
CXCR6	C-X-C motif chemokine receptor 6	3	Antimicrobials
CYBB	cytochrome b-245 beta chain	X	Antimicrobials
CYLD	CYLD lysine 63 deubiquitinase	16	Antimicrobials
CYSLTR1	cysteinyl leukotriene receptor 1	X	Chemokine_Receptors
CYSLTR2	cysteinyl leukotriene receptor 2	13	Chemokine_Receptors
DAXX	death domain associated protein	6	Antimicrobials
DCD	dermcidin	12	Antimicrobials
DCK	deoxycytidine kinase	4	Antimicrobials
DDX17	DEAD-box helicase 17	22	Antimicrobials
DDX58	DExD/H-box helicase 58	9	Antimicrobials
DEFA1	defensin alpha 1	8	Antimicrobials
DEFA1A3	defensin alpha 1 and alpha 3, variable copy number locus	8	Antimicrobials
DEFA1B	defensin alpha 1B	8	Antimicrobials
DEFA3	defensin alpha 3	8	Antimicrobials
DEFA4	defensin alpha 4	8	Antimicrobials
DEFA5	defensin alpha 5	8	Antimicrobials
DEFA6	defensin alpha 6	8	Antimicrobials
DEFA7P	defensin alpha 7, pseudogene	8	Antimicrobials
DEFB1	defensin beta 1	8	Antimicrobials
DEFB103A	defensin beta 103A	8	Antimicrobials
DEFB103B	defensin beta 103B	8	Antimicrobials
DEFB104A	defensin beta 104A	8	Antimicrobials

DEFB104B	defensin beta 104B	8	Antimicrobials
DEFB105A	defensin beta 105A	8	Antimicrobials
DEFB105B	defensin beta 105B	8	Antimicrobials
DEFB106A	defensin beta 106A	8	Antimicrobials
DEFB106B	defensin beta 106B	8	Antimicrobials
DEFB107A	defensin beta 107A	8	Antimicrobials
DEFB107B	defensin beta 107B	8	Antimicrobials
DEFB108B	defensin beta 108B	11	Antimicrobials
DEFB108F	defensin beta 108F (pseudogene)	4	Antimicrobials
DEFB110	defensin beta 110	6	Antimicrobials
DEFB112	defensin beta 112	6	Antimicrobials
DEFB113	defensin beta 113	6	Antimicrobials
DEFB114	defensin beta 114	6	Antimicrobials
DEFB115	defensin beta 115	20	Antimicrobials
DEFB116	defensin beta 116	20	Antimicrobials
DEFB117	defensin beta 117 (pseudogene)	20	Antimicrobials
DEFB118	defensin beta 118	20	Antimicrobials
DEFB119	defensin beta 119	20	Antimicrobials
DEFB121	defensin beta 121	20	Antimicrobials
DEFB123	defensin beta 123	20	Antimicrobials
DEFB124	defensin beta 124	20	Antimicrobials
DEFB125	defensin beta 125	20	Antimicrobials
DEFB126	defensin beta 126	20	Antimicrobials
DEFB127	defensin beta 127	20	Antimicrobials
DEFB128	defensin beta 128	20	Antimicrobials
DEFB129	defensin beta 129	20	Antimicrobials
DEFB130A	defensin beta 130A	8	Antimicrobials
DEFB130B	defensin beta 130B	8	Antimicrobials
DEFB131A	defensin beta 131A	4	Antimicrobials
DEFB131B	defensin beta 131B	11	Antimicrobials
DEFB131C	defensin beta 131C (pseudogene)	8	Antimicrobials
DEFB132	defensin beta 132	20	Antimicrobials

DEFB133	defensin beta 133	6	Antimicrobials
DEFB134	defensin beta 134	8	Antimicrobials
DEFB135	defensin beta 135	8	Antimicrobials
DEFB136	defensin beta 136	8	Antimicrobials
DEFB4A	defensin beta 4A	8	Antimicrobials
DEFB4B	defensin beta 4B	8	Antimicrobials
DES	desmin	2	Antimicrobials
DHX58	DExH-box helicase 58	17	Antimicrobials
DKK1	dickkopf WNT signaling pathway inhibitor 1	10	Cytokines
DLL4	delta like canonical Notch ligand 4	15	Antimicrobials
DMBT1	deleted in malignant brain tumors 1	10	Antimicrobials
DUOX1	dual oxidase 1	15	Antimicrobials
DUOX2	dual oxidase 2	15	Antimicrobials
EBI3	Epstein-Barr virus induced 3	19	Cytokines
ECD	ecdysoneless cell cycle regulator	10	Antimicrobials
ECPAS	Ecm29 proteasome adaptor and scaffold	9	Antigen_Processing_and_Presentation
EDN1	endothelin 1	6	Chemokines
EDN2	endothelin 2	1	Chemokines
EDN3	endothelin 3	20	Chemokines
EDNRA	endothelin receptor type A	4	Chemokine_Receptors
EDNRB	endothelin receptor type B	13	Chemokine_Receptors
EED	embryonic ectoderm development	11	Antimicrobials
EGF	epidermal growth factor	4	Cytokines
EGFR	epidermal growth factor receptor	7	Cytokine_Receptors
EIF2AK2	eukaryotic translation initiation factor 2 alpha kinase 2	2	Antimicrobials
ELANE	elastase, neutrophil expressed	19	Antimicrobials
ELAVL1	ELAV like RNA binding protein 1	19	Antimicrobials
ELN	elastin	7	Antimicrobials
ENDOU	endonuclease, poly(U) specific	12	Cytokines
ENG	endoglin	9	Cytokine_Receptors
EPGN	epithelial mitogen	4	Cytokines

EPO	erythropoietin	7	Cytokines
EPOR	erythropoietin receptor	19	Cytokine_Receptors
EPPIN	epididymal peptidase inhibitor	20	Antimicrobials
ERAP1	endoplasmic reticulum aminopeptidase 1	5	Antigen_Processing_and_Presentation
ERAP2	endoplasmic reticulum aminopeptidase 2	5	Antigen_Processing_and_Presentation
EREG	epiregulin	4	Cytokines
ESM1	endothelial cell specific molecule 1	5	Cytokines
ESR1	estrogen receptor 1	6	Cytokine_Receptors
ESR2	estrogen receptor 2	14	Cytokine_Receptors
ESRRA	estrogen related receptor alpha	11	Cytokine_Receptors
ESRRB	estrogen related receptor beta	14	Cytokine_Receptors
ESRRG	estrogen related receptor gamma	1	Cytokine_Receptors
F2R	coagulation factor II thrombin receptor	5	Antimicrobials
F2RL1	F2R like trypsin receptor 1	5	Antimicrobials
FABP12	fatty acid binding protein 12	8	Antimicrobials
FABP2	fatty acid binding protein 2	4	Antimicrobials
FABP3	fatty acid binding protein 3	1	Antimicrobials
FABP4	fatty acid binding protein 4	8	Antimicrobials
FABP5	fatty acid binding protein 5	8	Antimicrobials
FABP6	fatty acid binding protein 6	5	Antimicrobials
FABP7	fatty acid binding protein 7	6	Antimicrobials
FABP9	fatty acid binding protein 9	8	Antimicrobials
FAM3B	FAM3 metabolism regulating signaling molecule B	21	Cytokines
FAM3C	FAM3 metabolism regulating signaling molecule C	7	Cytokines
FAM3D	FAM3 metabolism regulating signaling molecule D	3	Cytokines
FAS	Fas cell surface death receptor	10	NaturalKiller_Cell_Cytotoxicity
FASLG	Fas ligand	1	Antimicrobials
FCER1G	Fc fragment of IgE receptor Ig	1	Antigen_Processing_and_Presentation
FCGR2B	Fc fragment of IgG receptor IIb	1	BCRSignalingPathway

FCGR3A	Fc fragment of IgG receptor IIIa	1	NaturalKiller_Cell_Cytotoxicity
FCGR3B	Fc fragment of IgG receptor IIIb	1	NaturalKiller_Cell_Cytotoxicity
FCGRT	Fc fragment of IgG receptor and transporter	19	Antigen_Processing_and_Presentation
FCN2	ficolin 2	9	Antimicrobials
FGA	fibrinogen alpha chain	4	Antimicrobials
FGF1	fibroblast growth factor 1	5	Cytokines
FGF10	fibroblast growth factor 10	5	Chemokines
FGF11	fibroblast growth factor 11	17	Cytokines
FGF12	fibroblast growth factor 12	3	Cytokines
FGF13	fibroblast growth factor 13	X	Cytokines
FGF14	fibroblast growth factor 14	13	Cytokines
FGF16	fibroblast growth factor 16	X	Cytokines
FGF17	fibroblast growth factor 17	8	Cytokines
FGF18	fibroblast growth factor 18	5	Cytokines
FGF19	fibroblast growth factor 19	11	Cytokines
FGF2	fibroblast growth factor 2	4	Antimicrobials
FGF20	fibroblast growth factor 20	8	Cytokines
FGF21	fibroblast growth factor 21	19	Cytokines
FGF22	fibroblast growth factor 22	19	Cytokines
FGF23	fibroblast growth factor 23	12	Cytokines
FGF3	fibroblast growth factor 3	11	Cytokines
FGF4	fibroblast growth factor 4	11	Cytokines
FGF5	fibroblast growth factor 5	4	Cytokines
FGF6	fibroblast growth factor 6	12	Cytokines
FGF7	fibroblast growth factor 7	15	Cytokines
FGF7P3	fibroblast growth factor 7 pseudogene 3	9	Cytokines
FGF7P6	fibroblast growth factor 7 pseudogene 6	9	Cytokines
FGF8	fibroblast growth factor 8	10	Cytokines
FGF9	fibroblast growth factor 9	13	Cytokines
FGFR1	fibroblast growth factor receptor 1	8	Cytokine_Receptors
FGFR2	fibroblast growth factor receptor 2	10	Cytokine_Receptors

FGFR3	fibroblast growth factor receptor 3	4	Cytokine_Receptors
FGFR4	fibroblast growth factor receptor 4	5	Cytokine_Receptors
FGFRL1	fibroblast growth factor receptor like 1	4	Cytokine_Receptors
FGR	FGR proto-oncogene, Src family tyrosine kinase	1	Antimicrobials
FIGNL2	fidgetin like 2	12	Cytokines
FLT1	fms related receptor tyrosine kinase 1	13	Cytokine_Receptors
FLT3	fms related receptor tyrosine kinase 3	13	Cytokine_Receptors
FLT3LG	fms related receptor tyrosine kinase 3 ligand	19	Cytokines
FLT4	fms related receptor tyrosine kinase 4	5	Cytokine_Receptors
FOS	Fos proto-oncogene, AP-1 transcription factor subunit	14	BCRSignalingPathway
FPR1	formyl peptide receptor 1	19	Chemokine_Receptors
FPR2	formyl peptide receptor 2	19	Chemokine_Receptors
FSHB	follicle stimulating hormone subunit beta	11	Cytokines
FSHR	follicle stimulating hormone receptor	2	Cytokine_Receptors
FURIN	furin, paired basic amino acid cleaving enzyme	15	Antimicrobials
FYN	FYN proto-oncogene, Src family tyrosine kinase	6	NaturalKiller_Cell_Cytotoxicity
GAL	galanin and GMAP prepropeptide	11	Cytokines
GALP	galanin like peptide	19	Cytokines
GALR2	galanin receptor 2	17	Cytokine_Receptors
GALR3	galanin receptor 3	22	Cytokine_Receptors
GAST	gastrin	17	Cytokines
GBP2	guanylate binding protein 2	1	Antimicrobials
GCG	glucagon	2	Cytokines
GCGR	glucagon receptor	17	Cytokine_Receptors
GDF1	growth differentiation factor 1	19	Cytokines
GDF10	growth differentiation factor 10	10	Cytokines
GDF11	growth differentiation factor 11	12	Cytokines
GDF15	growth differentiation factor 15	19	Antimicrobials
GDF2	growth differentiation factor 2	10	Cytokines
GDF3	growth differentiation factor 3	12	Cytokines
GDF5	growth differentiation factor 5	20	Cytokines
GDF6	growth differentiation factor 6	8	Cytokines

GDF7	growth differentiation factor 7	2	Cytokines
GDF9	growth differentiation factor 9	5	Cytokines
GDNF	glial cell derived neurotrophic factor	5	Cytokines
GFAP	glial fibrillary acidic protein	17	Antimicrobials
GH1	growth hormone 1	17	Cytokines
GH2	growth hormone 2	17	Cytokines
GHR	growth hormone receptor	5	Cytokine_Receptors
GHRH	growth hormone releasing hormone	20	Cytokines
GHRHR	growth hormone releasing hormone receptor	7	Cytokine_Receptors
GHRL	ghrelin and obestatin prepropeptide	3	Cytokines
GHSR	growth hormone secretagogue receptor	3	Cytokine_Receptors
GIP	gastric inhibitory polypeptide	17	Cytokines
GIPR	gastric inhibitory polypeptide receptor	19	Cytokine_Receptors
GKN1	gastrokine 1	2	Cytokines
GLP1R	glucagon like peptide 1 receptor	6	Cytokine_Receptors
GLP2R	glucagon like peptide 2 receptor	17	Cytokine_Receptors
GMFB	glia maturation factor beta	14	Cytokines
GMFG	glia maturation factor gamma	19	Cytokines
GNAI1	G protein subunit alpha i1	7	Antimicrobials
GNLY	granulysin	2	Antimicrobials
GNRH1	gonadotropin releasing hormone 1	8	Cytokines
GNRH2	gonadotropin releasing hormone 2	20	Cytokines
GNRHR	gonadotropin releasing hormone receptor	4	Cytokine_Receptors
GPER1	G protein-coupled estrogen receptor 1	7	Cytokine_Receptors
GPHA2	glycoprotein hormone subunit alpha 2	11	Cytokines
GPHB5	glycoprotein hormone subunit beta 5	14	Cytokines
GPI	glucose-6-phosphate isomerase	19	Cytokines
GPR17	G protein-coupled receptor 17	2	Chemokine_Receptors
GPR32	G protein-coupled receptor 32	19	Chemokine_Receptors
GPR33	G protein-coupled receptor 33	14	Chemokine_Receptors
GRAP2	GRB2 related adaptor protein 2	22	TCRsignalingPathway
GRB2	growth factor receptor bound protein 2	17	NaturalKiller_Cell_Cytotoxicity

GREM1	gremlin 1, DAN family BMP antagonist	15	Cytokines
GREM2	gremlin 2, DAN family BMP antagonist	1	Cytokines
GRK2	G protein-coupled receptor kinase 2	11	Antimicrobials
GRN	granulin precursor	17	Antimicrobials
GRP	gastrin releasing peptide	18	Cytokines
GSK3B	glycogen synthase kinase 3 beta	3	BCRSignalingPathway
GUCA2A	guanylate cyclase activator 2A	1	Cytokines
GZMB	granzyme B	14	NaturalKiller_Cell_Cytotoxicity
HAMP	hepcidin antimicrobial peptide	19	Antimicrobials
HBEGF	heparin binding EGF like growth factor	5	Cytokines
HCK	HCK proto-oncogene, Src family tyrosine kinase	20	Antimicrobials
HCST	hematopoietic cell signal transducer	19	NaturalKiller_Cell_Cytotoxicity
HDAC1	histone deacetylase 1	1	Antimicrobials
HDGF	heparin binding growth factor	1	Cytokines
HDGFL3	HDGF like 3	15	Cytokines
HFE	homeostatic iron regulator	6	Antigen_Processing_and_Presenta tion
HGF	hepatocyte growth factor	7	Antimicrobials
HJV	hemojuvelin BMP co-receptor	1	Antimicrobials
HLA-A	major histocompatibility complex, class I, A	6	Antigen_Processing_and_Presenta tion
HLA-B	major histocompatibility complex, class I, B	6	Antigen_Processing_and_Presenta tion
HLA-C	major histocompatibility complex, class I, C	6	Antigen_Processing_and_Presenta tion
HLA-DMA	major histocompatibility complex, class II, DM alpha	6	Antigen_Processing_and_Presenta tion
HLA-DMB	major histocompatibility complex, class II, DM beta	6	Antigen_Processing_and_Presenta tion
HLA-DOA	major histocompatibility complex, class II, DO alpha	6	Antigen_Processing_and_Presenta tion
HLA-DOB	major histocompatibility complex, class II, DO beta	6	Antigen_Processing_and_Presenta

			tion
HLA-DPA1	major histocompatibility complex, class II, DP alpha 1	6	Antigen_Processing_and_Presentation
HLA-DPB1	major histocompatibility complex, class II, DP beta 1	6	Antigen_Processing_and_Presentation
HLA-DQA1	major histocompatibility complex, class II, DQ alpha 1	6	Antigen_Processing_and_Presentation
HLA-DQA2	major histocompatibility complex, class II, DQ alpha 2	6	Antigen_Processing_and_Presentation
HLA-DQB1	major histocompatibility complex, class II, DQ beta 1	6	Antigen_Processing_and_Presentation
HLA-DRA	major histocompatibility complex, class II, DR alpha	6	Antigen_Processing_and_Presentation
HLA-DRB1	major histocompatibility complex, class II, DR beta 1	6	Antigen_Processing_and_Presentation
HLA-DRB3	major histocompatibility complex, class II, DR beta 3	6	Antigen_Processing_and_Presentation
HLA-DRB4	major histocompatibility complex, class II, DR beta 4	6	Antigen_Processing_and_Presentation
HLA-DRB5	major histocompatibility complex, class II, DR beta 5	6	Antigen_Processing_and_Presentation
HLA-E	major histocompatibility complex, class I, E	6	Antigen_Processing_and_Presentation
HLA-F	major histocompatibility complex, class I, F	6	Antigen_Processing_and_Presentation
HLA-G	major histocompatibility complex, class I, G	6	Antigen_Processing_and_Presentation
HLA-H	major histocompatibility complex, class I, H (pseudogene)	6	Antigen_Processing_and_Presentation
HMGB1	high mobility group box 1	13	Antimicrobials
HMOX1	heme oxygenase 1	22	Antimicrobials
HNF4A	hepatocyte nuclear factor 4 alpha	20	Cytokine_Receptors

HNF4G	hepatocyte nuclear factor 4 gamma	8	Cytokine_Receptors
HRAS	HRas proto-oncogene, GTPase	11	BCRSignalingPathway
HRG	histidine rich glycoprotein	3	Antimicrobials
HSP90AA1	heat shock protein 90 alpha family class A member 1	14	Antigen_Processing_and_Presentation
HSP90AB1	heat shock protein 90 alpha family class B member 1	6	Antigen_Processing_and_Presentation
HSPA1A	heat shock protein family A (Hsp70) member 1A	6	Antigen_Processing_and_Presentation
HSPA1B	heat shock protein family A (Hsp70) member 1B	6	Antigen_Processing_and_Presentation
HSPA1L	heat shock protein family A (Hsp70) member 1 like	6	Antigen_Processing_and_Presentation
HSPA2	heat shock protein family A (Hsp70) member 2	14	Antigen_Processing_and_Presentation
HSPA4	heat shock protein family A (Hsp70) member 4	5	Antigen_Processing_and_Presentation
HSPA5	heat shock protein family A (Hsp70) member 5	9	Antigen_Processing_and_Presentation
HSPA6	heat shock protein family A (Hsp70) member 6	1	Antigen_Processing_and_Presentation
HSPA8	heat shock protein family A (Hsp70) member 8	11	Antigen_Processing_and_Presentation
HTN1	histatin 1	4	Antimicrobials
HTN3	histatin 3	4	Antimicrobials
HTR1A	5-hydroxytryptamine receptor 1A	5	Antimicrobials
HTR3A	5-hydroxytryptamine receptor 3A	11	Cytokine_Receptors
HTR3B	5-hydroxytryptamine receptor 3B	11	Cytokine_Receptors
HTR3C	5-hydroxytryptamine receptor 3C	3	Cytokine_Receptors
HTR3D	5-hydroxytryptamine receptor 3D	3	Cytokine_Receptors
HTR3E	5-hydroxytryptamine receptor 3E	3	Cytokine_Receptors
IAPP	islet amyloid polypeptide	12	Cytokines

ICAM1	intercellular adhesion molecule 1	19	Antigen_Processing_and_Presentation
ICAM2	intercellular adhesion molecule 2	17	NaturalKiller_Cell_Cytotoxicity
ICOS	inducible T cell costimulator	2	TCRsignalingPathway
IDO1	indoleamine 2,3-dioxygenase 1	8	Antimicrobials
IFI30	IFI30 lysosomal thiol reductase	19	Antigen_Processing_and_Presentation
IFIH1	interferon induced with helicase C domain 1	2	Antimicrobials
IFITM1	interferon induced transmembrane protein 1	11	BCRSignalingPathway
IFN1@	-	9	Antimicrobials
IFNA1	interferon alpha 1	9	Antigen_Processing_and_Presentation
IFNA10	interferon alpha 10	9	Antigen_Processing_and_Presentation
IFNA13	interferon alpha 13	9	Antigen_Processing_and_Presentation
IFNA14	interferon alpha 14	9	Antigen_Processing_and_Presentation
IFNA16	interferon alpha 16	9	Antigen_Processing_and_Presentation
IFNA17	interferon alpha 17	9	Antigen_Processing_and_Presentation
IFNA2	interferon alpha 2	9	Antigen_Processing_and_Presentation
IFNA21	interferon alpha 21	9	Antigen_Processing_and_Presentation
IFNA4	interferon alpha 4	9	Antigen_Processing_and_Presentation
IFNA5	interferon alpha 5	9	Antigen_Processing_and_Presentation
IFNA6	interferon alpha 6	9	Antigen_Processing_and_Presentation

IFNA7	interferon alpha 7	9	Antigen_Processing_and_Presentation
IFNA8	interferon alpha 8	9	Antigen_Processing_and_Presentation
IFNAR1	interferon alpha and beta receptor subunit 1	21	Antimicrobials
IFNAR2	interferon alpha and beta receptor subunit 2	21	Antimicrobials
IFNB1	interferon beta 1	9	Antimicrobials
IFNE	interferon epsilon	9	Cytokines
IFNG	interferon gamma	12	Antigen_Processing_and_Presentation
IFNGR1	interferon gamma receptor 1	6	Antimicrobials
IFNGR2	interferon gamma receptor 2	21	Cytokine_Receptors
IFNK	interferon kappa	9	Cytokines
IFNL1	interferon lambda 1	19	Antimicrobials
IFNL2	interferon lambda 2	19	Antimicrobials
IFNL3	interferon lambda 3	19	Cytokines
IFNLR1	interferon lambda receptor 1	1	Antimicrobials
IFNW1	interferon omega 1	9	Cytokines
IGF1	insulin like growth factor 1	12	Cytokines
IGF1R	insulin like growth factor 1 receptor	15	Cytokine_Receptors
IGF2	insulin like growth factor 2	11	Cytokines
IGF2R	insulin like growth factor 2 receptor	6	Cytokine_Receptors
IGH	immunoglobulin heavy locus	14	BCRSignalingPathway
IGHA1	immunoglobulin heavy constant alpha 1	14	BCRSignalingPathway
IGHA2	immunoglobulin heavy constant alpha 2 (A2m marker)	14	BCRSignalingPathway
IGHD	immunoglobulin heavy constant delta	14	BCRSignalingPathway
IGHD1-1	immunoglobulin heavy diversity 1-1	14	BCRSignalingPathway
IGHD1-14	immunoglobulin heavy diversity 1-14 (non-functional)	14	BCRSignalingPathway
IGHD1-20	immunoglobulin heavy diversity 1-20	14	BCRSignalingPathway
IGHD1-26	immunoglobulin heavy diversity 1-26	14	BCRSignalingPathway
IGHD1-7	immunoglobulin heavy diversity 1-7	14	BCRSignalingPathway
IGHD2-15	immunoglobulin heavy diversity 2-15	14	BCRSignalingPathway

IGHD2-2	immunoglobulin heavy diversity 2-2	14	BCRSignalingPathway
IGHD2-21	immunoglobulin heavy diversity 2-21	14	BCRSignalingPathway
IGHD2-8	immunoglobulin heavy diversity 2-8	14	BCRSignalingPathway
IGHD3-10	immunoglobulin heavy diversity 3-10	14	BCRSignalingPathway
IGHD3-16	immunoglobulin heavy diversity 3-16	14	BCRSignalingPathway
IGHD3-22	immunoglobulin heavy diversity 3-22	14	BCRSignalingPathway
IGHD3-3	immunoglobulin heavy diversity 3-3	14	BCRSignalingPathway
IGHD3-9	immunoglobulin heavy diversity 3-9	14	BCRSignalingPathway
IGHD4-11	immunoglobulin heavy diversity 4-11 (non-functional)	14	BCRSignalingPathway
IGHD4-17	immunoglobulin heavy diversity 4-17	14	BCRSignalingPathway
IGHD4-23	immunoglobulin heavy diversity 4-23 (non-functional)	14	BCRSignalingPathway
IGHD4-4	immunoglobulin heavy diversity 4-4	14	BCRSignalingPathway
IGHD5-12	immunoglobulin heavy diversity 5-12	14	BCRSignalingPathway
IGHD5-18	immunoglobulin heavy diversity 5-18	14	BCRSignalingPathway
IGHD5-24	immunoglobulin heavy diversity 5-24 (non-functional)	14	BCRSignalingPathway
IGHD5-5	immunoglobulin heavy diversity 5-5	14	BCRSignalingPathway
IGHD6-13	immunoglobulin heavy diversity 6-13	14	BCRSignalingPathway
IGHD6-19	immunoglobulin heavy diversity 6-19	14	BCRSignalingPathway
IGHD6-25	immunoglobulin heavy diversity 6-25	14	BCRSignalingPathway
IGHD6-6	immunoglobulin heavy diversity 6-6	14	BCRSignalingPathway
IGHD7-27	immunoglobulin heavy diversity 7-27	14	BCRSignalingPathway
IGHE	immunoglobulin heavy constant epsilon	14	BCRSignalingPathway
IGHG1	immunoglobulin heavy constant gamma 1 (G1m marker)	14	BCRSignalingPathway
IGHG2	immunoglobulin heavy constant gamma 2 (G2m marker)	14	BCRSignalingPathway
IGHG3	immunoglobulin heavy constant gamma 3 (G3m marker)	14	BCRSignalingPathway
IGHG4	immunoglobulin heavy constant gamma 4 (G4m marker)	14	BCRSignalingPathway
IGHJ1	immunoglobulin heavy joining 1	14	BCRSignalingPathway
IGHJ2	immunoglobulin heavy joining 2	14	BCRSignalingPathway
IGHJ3	immunoglobulin heavy joining 3	14	BCRSignalingPathway
IGHJ4	immunoglobulin heavy joining 4	14	BCRSignalingPathway
IGHJ5	immunoglobulin heavy joining 5	14	BCRSignalingPathway
IGHJ6	immunoglobulin heavy joining 6	14	BCRSignalingPathway

IGHM	immunoglobulin heavy constant mu	14	BCRSignalingPathway
IGHV1-18	immunoglobulin heavy variable 1-18	14	BCRSignalingPathway
IGHV1-2	immunoglobulin heavy variable 1-2	14	BCRSignalingPathway
IGHV1-24	immunoglobulin heavy variable 1-24	14	BCRSignalingPathway
IGHV1-3	immunoglobulin heavy variable 1-3	14	BCRSignalingPathway
IGHV1-38-4	immunoglobulin heavy variable 1-38-4 (non-functional)	14	BCRSignalingPathway
IGHV1-45	immunoglobulin heavy variable 1-45	14	BCRSignalingPathway
IGHV1-46	immunoglobulin heavy variable 1-46	14	BCRSignalingPathway
IGHV1-58	immunoglobulin heavy variable 1-58	14	BCRSignalingPathway
IGHV1-69	immunoglobulin heavy variable 1-69	14	BCRSignalingPathway
IGHV1-69-2	immunoglobulin heavy variable 1-69-2	14	BCRSignalingPathway
IGHV1-8	immunoglobulin heavy variable 1-8	14	BCRSignalingPathway
IGHV2-26	immunoglobulin heavy variable 2-26	14	BCRSignalingPathway
IGHV2-5	immunoglobulin heavy variable 2-5	14	BCRSignalingPathway
IGHV2-70	immunoglobulin heavy variable 2-70	14	BCRSignalingPathway
IGHV3-11	immunoglobulin heavy variable 3-11	14	BCRSignalingPathway
IGHV3-13	immunoglobulin heavy variable 3-13	14	BCRSignalingPathway
IGHV3-15	immunoglobulin heavy variable 3-15	14	BCRSignalingPathway
IGHV3-16	immunoglobulin heavy variable 3-16 (non-functional)	14	BCRSignalingPathway
IGHV3-20	immunoglobulin heavy variable 3-20	14	BCRSignalingPathway
IGHV3-21	immunoglobulin heavy variable 3-21	14	BCRSignalingPathway
IGHV3-23	immunoglobulin heavy variable 3-23	14	BCRSignalingPathway
IGHV3-30	immunoglobulin heavy variable 3-30	14	BCRSignalingPathway
IGHV3-30-3	immunoglobulin heavy variable 3-30-3	14	BCRSignalingPathway
IGHV3-30-5	immunoglobulin heavy variable 3-30-5	14	BCRSignalingPathway
IGHV3-33	immunoglobulin heavy variable 3-33	14	BCRSignalingPathway
IGHV3-35	immunoglobulin heavy variable 3-35 (non-functional)	14	BCRSignalingPathway
IGHV3-38	immunoglobulin heavy variable 3-38 (non-functional)	14	BCRSignalingPathway
IGHV3-38-3	immunoglobulin heavy variable 3-38-3 (non-functional)	14	BCRSignalingPathway
IGHV3-43	immunoglobulin heavy variable 3-43	14	BCRSignalingPathway
IGHV3-48	immunoglobulin heavy variable 3-48	14	BCRSignalingPathway
IGHV3-49	immunoglobulin heavy variable 3-49	14	BCRSignalingPathway

IGHV3-53	immunoglobulin heavy variable 3-53	14	BCRSignalingPathway
IGHV3-64	immunoglobulin heavy variable 3-64	14	BCRSignalingPathway
IGHV3-66	immunoglobulin heavy variable 3-66	14	BCRSignalingPathway
IGHV3-69-1	immunoglobulin heavy variable 3-69-1 (pseudogene)	14	BCRSignalingPathway
IGHV3-7	immunoglobulin heavy variable 3-7	14	BCRSignalingPathway
IGHV3-72	immunoglobulin heavy variable 3-72	14	BCRSignalingPathway
IGHV3-73	immunoglobulin heavy variable 3-73	14	BCRSignalingPathway
IGHV3-74	immunoglobulin heavy variable 3-74	14	BCRSignalingPathway
IGHV3-9	immunoglobulin heavy variable 3-9	14	BCRSignalingPathway
IGHV4-28	immunoglobulin heavy variable 4-28	14	BCRSignalingPathway
IGHV4-30-1	immunoglobulin heavy variable 4-30-1	14	BCRSignalingPathway
IGHV4-30-2	immunoglobulin heavy variable 4-30-2	14	BCRSignalingPathway
IGHV4-30-4	immunoglobulin heavy variable 4-30-4	14	BCRSignalingPathway
IGHV4-31	immunoglobulin heavy variable 4-31	14	BCRSignalingPathway
IGHV4-34	immunoglobulin heavy variable 4-34	14	BCRSignalingPathway
IGHV4-38-2	immunoglobulin heavy variable 4-38-2	14	BCRSignalingPathway
IGHV4-39	immunoglobulin heavy variable 4-39	14	BCRSignalingPathway
IGHV4-4	immunoglobulin heavy variable 4-4	14	BCRSignalingPathway
IGHV4-59	immunoglobulin heavy variable 4-59	14	BCRSignalingPathway
IGHV4-61	immunoglobulin heavy variable 4-61	14	BCRSignalingPathway
IGHV5-10-1	immunoglobulin heavy variable 5-10-1	14	BCRSignalingPathway
IGHV5-51	immunoglobulin heavy variable 5-51	14	BCRSignalingPathway
IGHV6-1	immunoglobulin heavy variable 6-1	14	BCRSignalingPathway
IGHV7-4-1	immunoglobulin heavy variable 7-4-1	14	BCRSignalingPathway
IGHV7-81	immunoglobulin heavy variable 7-81 (non-functional)	14	BCRSignalingPathway
IGK	immunoglobulin kappa locus	2	BCRSignalingPathway
IGKC	immunoglobulin kappa constant	2	BCRSignalingPathway
IGKDEL	immunoglobulin kappa deleting element or like	2	BCRSignalingPathway
IGKJ	-	2	BCRSignalingPathway
IGKJ1	immunoglobulin kappa joining 1	2	BCRSignalingPathway
IGKJ2	immunoglobulin kappa joining 2	2	BCRSignalingPathway
IGKJ3	immunoglobulin kappa joining 3	2	BCRSignalingPathway

IGKJ4	immunoglobulin kappa joining 4	2	BCRSignalingPathway
IGKJ5	immunoglobulin kappa joining 5	2	BCRSignalingPathway
IGKV@	-	2	BCRSignalingPathway
IGKV1-12	immunoglobulin kappa variable 1-12	2	BCRSignalingPathway
IGKV1-13	immunoglobulin kappa variable 1-13	2	BCRSignalingPathway
IGKV1-16	immunoglobulin kappa variable 1-16	2	BCRSignalingPathway
IGKV1-17	immunoglobulin kappa variable 1-17	2	BCRSignalingPathway
IGKV1-27	immunoglobulin kappa variable 1-27	2	BCRSignalingPathway
IGKV1-33	immunoglobulin kappa variable 1-33	2	BCRSignalingPathway
IGKV1-37	immunoglobulin kappa variable 1-37 (non-functional)	2	BCRSignalingPathway
IGKV1-39	immunoglobulin kappa variable 1-39	2	BCRSignalingPathway
IGKV1-5	immunoglobulin kappa variable 1-5	2	BCRSignalingPathway
IGKV1-6	immunoglobulin kappa variable 1-6	2	BCRSignalingPathway
IGKV1-8	immunoglobulin kappa variable 1-8	2	BCRSignalingPathway
IGKV1-9	immunoglobulin kappa variable 1-9	2	BCRSignalingPathway
IGKV1D-12	immunoglobulin kappa variable 1D-12	2	BCRSignalingPathway
IGKV1D-13	immunoglobulin kappa variable 1D-13	2	BCRSignalingPathway
IGKV1D-16	immunoglobulin kappa variable 1D-16	2	BCRSignalingPathway
IGKV1D-17	immunoglobulin kappa variable 1D-17	2	BCRSignalingPathway
IGKV1D-33	immunoglobulin kappa variable 1D-33	2	BCRSignalingPathway
IGKV1D-37	immunoglobulin kappa variable 1D-37 (non-functional)	2	BCRSignalingPathway
IGKV1D-39	immunoglobulin kappa variable 1D-39	2	BCRSignalingPathway
IGKV1D-42	immunoglobulin kappa variable 1D-42 (non-functional)	2	BCRSignalingPathway
IGKV1D-43	immunoglobulin kappa variable 1D-43	2	BCRSignalingPathway
IGKV1D-8	immunoglobulin kappa variable 1D-8	2	BCRSignalingPathway
IGKV2-24	immunoglobulin kappa variable 2-24	2	BCRSignalingPathway
IGKV2-28	immunoglobulin kappa variable 2-28	2	BCRSignalingPathway
IGKV2-30	immunoglobulin kappa variable 2-30	2	BCRSignalingPathway
IGKV2-40	immunoglobulin kappa variable 2-40	2	BCRSignalingPathway
IGKV2D-24	immunoglobulin kappa variable 2D-24 (non-functional)	2	BCRSignalingPathway
IGKV2D-28	immunoglobulin kappa variable 2D-28	2	BCRSignalingPathway
IGKV2D-29	immunoglobulin kappa variable 2D-29	2	BCRSignalingPathway

IGKV2D-30	immunoglobulin kappa variable 2D-30	2	BCRSignalingPathway
IGKV2D-40	immunoglobulin kappa variable 2D-40	2	BCRSignalingPathway
IGKV3-11	immunoglobulin kappa variable 3-11	2	BCRSignalingPathway
IGKV3-15	immunoglobulin kappa variable 3-15	2	BCRSignalingPathway
IGKV3-20	immunoglobulin kappa variable 3-20	2	BCRSignalingPathway
IGKV3-7	immunoglobulin kappa variable 3-7 (non-functional)	2	BCRSignalingPathway
IGKV3D-11	immunoglobulin kappa variable 3D-11	2	BCRSignalingPathway
IGKV3D-15	immunoglobulin kappa variable 3D-15	2	BCRSignalingPathway
IGKV3D-20	immunoglobulin kappa variable 3D-20	2	BCRSignalingPathway
IGKV3D-7	immunoglobulin kappa variable 3D-7	2	BCRSignalingPathway
IGKV4-1	immunoglobulin kappa variable 4-1	2	BCRSignalingPathway
IGKV5-2	immunoglobulin kappa variable 5-2	2	BCRSignalingPathway
IGKV6-21	immunoglobulin kappa variable 6-21 (non-functional)	2	BCRSignalingPathway
IGKV6D-21	immunoglobulin kappa variable 6D-21 (non-functional)	2	BCRSignalingPathway
IGKV6D-41	immunoglobulin kappa variable 6D-41 (non-functional)	2	BCRSignalingPathway
IGL	immunoglobulin lambda locus	22	BCRSignalingPathway
IGLC1	immunoglobulin lambda constant 1	22	BCRSignalingPathway
IGLC2	immunoglobulin lambda constant 2	22	BCRSignalingPathway
IGLC3	immunoglobulin lambda constant 3 (Kern-Oz+ marker)	22	BCRSignalingPathway
IGLC6	immunoglobulin lambda constant 6	22	BCRSignalingPathway
IGLC7	immunoglobulin lambda constant 7	22	BCRSignalingPathway
IGLJ	-	22	BCRSignalingPathway
IGLJ1	immunoglobulin lambda joining 1	22	BCRSignalingPathway
IGLJ2	immunoglobulin lambda joining 2	22	BCRSignalingPathway
IGLJ3	immunoglobulin lambda joining 3	22	BCRSignalingPathway
IGLJ4	immunoglobulin lambda joining 4 (non-functional)	22	BCRSignalingPathway
IGLJ5	immunoglobulin lambda joining 5 (non-functional)	22	BCRSignalingPathway
IGLJ6	immunoglobulin lambda joining 6	22	BCRSignalingPathway
IGLJ7	immunoglobulin lambda joining 7	22	BCRSignalingPathway
IGLV@	-	22	BCRSignalingPathway
IGLV10-54	immunoglobulin lambda variable 10-54	22	BCRSignalingPathway
IGLV11-55	immunoglobulin lambda variable 11-55 (non-functional)	22	BCRSignalingPathway

IGLV1-36	immunoglobulin lambda variable 1-36	22	BCRSignalingPathway
IGLV1-40	immunoglobulin lambda variable 1-40	22	BCRSignalingPathway
IGLV1-44	immunoglobulin lambda variable 1-44	22	BCRSignalingPathway
IGLV1-47	immunoglobulin lambda variable 1-47	22	BCRSignalingPathway
IGLV1-50	immunoglobulin lambda variable 1-50 (non-functional)	22	BCRSignalingPathway
IGLV1-51	immunoglobulin lambda variable 1-51	22	BCRSignalingPathway
IGLV2-11	immunoglobulin lambda variable 2-11	22	BCRSignalingPathway
IGLV2-14	immunoglobulin lambda variable 2-14	22	BCRSignalingPathway
IGLV2-18	immunoglobulin lambda variable 2-18	22	BCRSignalingPathway
IGLV2-23	immunoglobulin lambda variable 2-23	22	BCRSignalingPathway
IGLV2-33	immunoglobulin lambda variable 2-33 (non-functional)	22	BCRSignalingPathway
IGLV2-8	immunoglobulin lambda variable 2-8	22	BCRSignalingPathway
IGLV3-1	immunoglobulin lambda variable 3-1	22	BCRSignalingPathway
IGLV3-10	immunoglobulin lambda variable 3-10	22	BCRSignalingPathway
IGLV3-12	immunoglobulin lambda variable 3-12	22	BCRSignalingPathway
IGLV3-16	immunoglobulin lambda variable 3-16	22	BCRSignalingPathway
IGLV3-19	immunoglobulin lambda variable 3-19	22	BCRSignalingPathway
IGLV3-21	immunoglobulin lambda variable 3-21	22	BCRSignalingPathway
IGLV3-22	immunoglobulin lambda variable 3-22	22	BCRSignalingPathway
IGLV3-25	immunoglobulin lambda variable 3-25	22	BCRSignalingPathway
IGLV3-27	immunoglobulin lambda variable 3-27	22	BCRSignalingPathway
IGLV3-32	immunoglobulin lambda variable 3-32 (non-functional)	22	BCRSignalingPathway
IGLV3-9	immunoglobulin lambda variable 3-9	22	BCRSignalingPathway
IGLV4-3	immunoglobulin lambda variable 4-3	22	BCRSignalingPathway
IGLV4-60	immunoglobulin lambda variable 4-60	22	BCRSignalingPathway
IGLV4-69	immunoglobulin lambda variable 4-69	22	BCRSignalingPathway
IGLV5-37	immunoglobulin lambda variable 5-37	22	BCRSignalingPathway
IGLV5-39	immunoglobulin lambda variable 5-39	22	BCRSignalingPathway
IGLV5-45	immunoglobulin lambda variable 5-45	22	BCRSignalingPathway
IGLV5-48	immunoglobulin lambda variable 5-48 (non-functional)	22	BCRSignalingPathway
IGLV5-52	immunoglobulin lambda variable 5-52	22	BCRSignalingPathway
IGLV6-57	immunoglobulin lambda variable 6-57	22	BCRSignalingPathway

IGLV7-43	immunoglobulin lambda variable 7-43	22	BCRSignalingPathway
IGLV7-46	immunoglobulin lambda variable 7-46	22	BCRSignalingPathway
IGLV8-61	immunoglobulin lambda variable 8-61	22	BCRSignalingPathway
IGLV9-49	immunoglobulin lambda variable 9-49	22	BCRSignalingPathway
IKBKB	inhibitor of nuclear factor kappa B kinase subunit beta	8	BCRSignalingPathway
IKBKE	inhibitor of nuclear factor kappa B kinase subunit epsilon	1	Antimicrobials
IKBKG	inhibitor of nuclear factor kappa B kinase regulatory subunit gamma	X	BCRSignalingPathway
IL10	interleukin 10	1	Antimicrobials
IL10RA	interleukin 10 receptor subunit alpha	11	Cytokine_Receptors
IL10RB	interleukin 10 receptor subunit beta	21	Cytokine_Receptors
IL11	interleukin 11	19	Cytokines
IL11RA	interleukin 11 receptor subunit alpha	9	Cytokine_Receptors
IL12A	interleukin 12A	3	Cytokines
IL12B	interleukin 12B	5	Antimicrobials
IL12RB1	interleukin 12 receptor subunit beta 1	19	Cytokine_Receptors
IL12RB2	interleukin 12 receptor subunit beta 2	1	Cytokine_Receptors
IL13	interleukin 13	5	Antimicrobials
IL13RA1	interleukin 13 receptor subunit alpha 1	X	Cytokine_Receptors
IL13RA2	interleukin 13 receptor subunit alpha 2	X	Cytokine_Receptors
IL15	interleukin 15	4	Antimicrobials
IL15RA	interleukin 15 receptor subunit alpha	10	Cytokine_Receptors
IL16	interleukin 16	15	Cytokines
IL17A	interleukin 17A	6	Antimicrobials
IL17B	interleukin 17B	5	Cytokines
IL17C	interleukin 17C	16	Cytokines
IL17D	interleukin 17D	13	Cytokines
IL17F	interleukin 17F	6	Cytokines
IL17RA	interleukin 17 receptor A	22	Cytokine_Receptors
IL17RB	interleukin 17 receptor B	3	Cytokine_Receptors
IL17RC	interleukin 17 receptor C	3	Cytokine_Receptors
IL17RD	interleukin 17 receptor D	3	Cytokine_Receptors
IL17RE	interleukin 17 receptor E	3	Cytokine_Receptors

IL18	interleukin 18	11	Antimicrobials
IL18R1	interleukin 18 receptor 1	2	Cytokine_Receptors
IL18RAP	interleukin 18 receptor accessory protein	2	Cytokine_Receptors
IL19	interleukin 19	1	Cytokines
IL1A	interleukin 1 alpha	2	Antimicrobials
IL1B	interleukin 1 beta	2	Antimicrobials
IL1F10	interleukin 1 family member 10	2	Cytokines
IL1R1	interleukin 1 receptor type 1	2	Cytokine_Receptors
IL1R2	interleukin 1 receptor type 2	2	Cytokine_Receptors
IL1RAP	interleukin 1 receptor accessory protein	3	Cytokine_Receptors
IL1RL1	interleukin 1 receptor like 1	2	Cytokine_Receptors
IL1RL2	interleukin 1 receptor like 2	2	Cytokine_Receptors
IL1RN	interleukin 1 receptor antagonist	2	Cytokines
IL2	interleukin 2	4	Antimicrobials
IL20	interleukin 20	1	Cytokines
IL20RA	interleukin 20 receptor subunit alpha	6	Cytokine_Receptors
IL20RB	interleukin 20 receptor subunit beta	3	Cytokine_Receptors
IL21	interleukin 21	4	Cytokines
IL21R	interleukin 21 receptor	16	Cytokine_Receptors
IL22	interleukin 22	12	Antimicrobials
IL22RA1	interleukin 22 receptor subunit alpha 1	1	Cytokine_Receptors
IL22RA2	interleukin 22 receptor subunit alpha 2	6	Cytokine_Receptors
IL23A	interleukin 23 subunit alpha	12	Cytokines
IL23R	interleukin 23 receptor	1	Cytokine_Receptors
IL24	interleukin 24	1	Cytokines
IL25	interleukin 25	14	Cytokines
IL26	interleukin 26	12	Cytokines
IL27	interleukin 27	16	Antimicrobials
IL27RA	interleukin 27 receptor subunit alpha	19	Cytokine_Receptors
IL2RA	interleukin 2 receptor subunit alpha	10	Cytokine_Receptors
IL2RB	interleukin 2 receptor subunit beta	22	Cytokine_Receptors
IL2RG	interleukin 2 receptor subunit gamma	X	Cytokine_Receptors

IL3	interleukin 3	5	Cytokines
IL31	interleukin 31	12	Cytokines
IL31RA	interleukin 31 receptor A	5	Cytokine_Receptors
IL32	interleukin 32	16	Cytokines
IL33	interleukin 33	9	Cytokines
IL34	interleukin 34	16	Cytokines
IL36A	interleukin 36 alpha	2	Cytokines
IL36B	interleukin 36 beta	2	Cytokines
IL36G	interleukin 36 gamma	2	Cytokines
IL36RN	interleukin 36 receptor antagonist	2	Cytokines
IL37	interleukin 37	2	Cytokines
IL3RA	interleukin 3 receptor subunit alpha	X|Y	Cytokine_Receptors
IL4	interleukin 4	5	Antimicrobials
IL4R	interleukin 4 receptor	16	Cytokine_Receptors
IL5	interleukin 5	5	Cytokines
IL5RA	interleukin 5 receptor subunit alpha	3	Cytokine_Receptors
IL6	interleukin 6	7	Antimicrobials
IL6R	interleukin 6 receptor	1	Cytokine_Receptors
IL6ST	interleukin 6 signal transducer	5	Cytokines
IL7	interleukin 7	8	Cytokines
IL7R	interleukin 7 receptor	5	Antimicrobials
IL9	interleukin 9	5	Cytokines
IL9R	interleukin 9 receptor	X|Y	Cytokine_Receptors
ILK	integrin linked kinase	11	Antimicrobials
INHA	inhibin subunit alpha	2	Cytokines
INHBA	inhibin subunit beta A	7	Cytokines
INHBB	inhibin subunit beta B	2	Cytokines
INHBC	inhibin subunit beta C	12	Cytokines
INHBE	inhibin subunit beta E	12	Cytokines
INPP5D	inositol polyphosphate-5-phosphatase D	2	BCRSignalingPathway
INS	insulin	11	Cytokines
INS-IGF2	INS-IGF2 readthrough	11	Cytokines

INSL3	insulin like 3	19	Cytokines
INSL4	insulin like 4	9	Cytokines
INSL5	insulin like 5	1	Cytokines
INSL6	insulin like 6	9	Cytokines
INSR	insulin receptor	19	Cytokine_Receptors
IREB2	iron responsive element binding protein 2	15	Antimicrobials
IRF1	interferon regulatory factor 1	5	Antimicrobials
IRF3	interferon regulatory factor 3	19	Antimicrobials
IRF5	interferon regulatory factor 5	7	Antimicrobials
IRF7	interferon regulatory factor 7	11	Antimicrobials
IRF9	interferon regulatory factor 9	14	Antimicrobials
ISG15	ISG15 ubiquitin like modifier	1	Antimicrobials
ISG20	interferon stimulated exonuclease gene 20	15	Antimicrobials
ISG20L2	interferon stimulated exonuclease gene 20 like 2	1	Antimicrobials
ITGAL	integrin subunit alpha L	16	NaturalKiller_Cell_Cytotoxicity
ITGAV	integrin subunit alpha V	2	Antimicrobials
ITGB2	integrin subunit beta 2	21	NaturalKiller_Cell_Cytotoxicity
ITK	IL2 inducible T cell kinase	5	TCRsignalingPathway
JAG1	jagged canonical Notch ligand 1	20	Cytokines
JAG2	jagged canonical Notch ligand 2	14	Cytokines
JAK1	Janus kinase 1	1	Antimicrobials
JAK2	Janus kinase 2	9	Antimicrobials
JUN	Jun proto-oncogene, AP-1 transcription factor subunit	1	Antimicrobials
JUND	JunD proto-oncogene, AP-1 transcription factor subunit	19	Antimicrobials
KCNH2	potassium voltage-gated channel subfamily H member 2	7	Antimicrobials
KDR	kinase insert domain receptor	4	Cytokine_Receptors
KIR2DL1	killer cell immunoglobulin like receptor, two Ig domains and long cytoplasmic tail 1	19	Antigen_Processing_and_Presentation
KIR2DL2	killer cell immunoglobulin like receptor, two Ig domains and long cytoplasmic tail 2	19	Antigen_Processing_and_Presentation
KIR2DL3	killer cell immunoglobulin like receptor, two Ig domains and long cytoplasmic tail 3	19	Antigen_Processing_and_Presentation

KIR2DL4	killer cell immunoglobulin like receptor, two Ig domains and long cytoplasmic tail 4	19	Antigen_Processing_and_Presentation
KIR2DL5A	killer cell immunoglobulin like receptor, two Ig domains and long cytoplasmic tail 5A	19	Antigen_Processing_and_Presentation
KIR2DS1	killer cell immunoglobulin like receptor, two Ig domains and short cytoplasmic tail 1	19	Antigen_Processing_and_Presentation
KIR2DS3	killer cell immunoglobulin like receptor, two Ig domains and short cytoplasmic tail 3	19	Antigen_Processing_and_Presentation
KIR2DS4	killer cell immunoglobulin like receptor, two Ig domains and short cytoplasmic tail 4	19	Antigen_Processing_and_Presentation
KIR2DS5	killer cell immunoglobulin like receptor, two Ig domains and short cytoplasmic tail 5	19	Antigen_Processing_and_Presentation
KIR3DL1	killer cell immunoglobulin like receptor, three Ig domains and long cytoplasmic tail 1	19	Antigen_Processing_and_Presentation
KIR3DL2	killer cell immunoglobulin like receptor, three Ig domains and long cytoplasmic tail 2	19	Antigen_Processing_and_Presentation
KIR3DL3	killer cell immunoglobulin like receptor, three Ig domains and long cytoplasmic tail 3	19	Antigen_Processing_and_Presentation
KITLG	KIT ligand	12	Cytokines
KL	klotho	13	Cytokines
KLKB1	kallikrein B1	4	Antimicrobials
KLRC1	killer cell lectin like receptor C1	12	Antigen_Processing_and_Presentation
KLRC2	killer cell lectin like receptor C2	12	Antigen_Processing_and_Presentation
KLRC3	killer cell lectin like receptor C3	12	Antigen_Processing_and_Presentation
KLRC4	killer cell lectin like receptor C4	12	Antigen_Processing_and_Presentation
KLRD1	killer cell lectin like receptor D1	12	Antigen_Processing_and_Presentation
KLRK1	killer cell lectin like receptor K1	12	Antimicrobials

KNG1	kininogen 1	3	Antimicrobials
KRAS	KRAS proto-oncogene, GTPase	12	BCRSignalingPathway
LACRT	lacritin	12	Cytokines
LALBA	lactalbumin alpha	12	Antimicrobials
LANCL1	LanC like 1	2	Antimicrobials
LAT	linker for activation of T cells	16	NaturalKiller_Cell_Cytotoxicity
LBP	lipopolysaccharide binding protein	20	Antimicrobials
LCK	LCK proto-oncogene, Src family tyrosine kinase	1	NaturalKiller_Cell_Cytotoxicity
LCN1	lipocalin 1	9	Antimicrobials
LCN10	lipocalin 10	9	Antimicrobials
LCN12	lipocalin 12	9	Antimicrobials
LCN15	lipocalin 15	9	Antimicrobials
LCN1P1	lipocalin 1 pseudogene 1	9	Antimicrobials
LCN2	lipocalin 2	9	Antimicrobials
LCN6	lipocalin 6	9	Antimicrobials
LCN8	lipocalin 8	9	Antimicrobials
LCN9	lipocalin 9	9	Antimicrobials
LCNL1	lipocalin like 1	9	Antimicrobials
LCP2	lymphocyte cytosolic protein 2	5	NaturalKiller_Cell_Cytotoxicity
LEAP2	liver enriched antimicrobial peptide 2	5	Antimicrobials
LECT2	leukocyte cell derived chemotaxin 2	5	Chemokines
LEFTY1	left-right determination factor 1	1	Cytokines
LEFTY2	left-right determination factor 2	1	Cytokines
LEP	leptin	7	Antimicrobials
LEPR	leptin receptor	1	Cytokine_Receptors
LGMN	legumain	14	Antigen_Processing_and_Presentation
LGR4	leucine rich repeat containing G protein-coupled receptor 4	11	Cytokine_Receptors
LGR5	leucine rich repeat containing G protein-coupled receptor 5	12	Cytokine_Receptors
LGR6	leucine rich repeat containing G protein-coupled receptor 6	1	Cytokine_Receptors
LHB	luteinizing hormone subunit beta	19	Cytokines
LHCGR	luteinizing hormone/choriogonadotropin receptor	2	Cytokine_Receptors

LIF	LIF interleukin 6 family cytokine	22	Cytokines
LIFR	LIF receptor subunit alpha	5	Cytokine_Receptors
LILRB3	leukocyte immunoglobulin like receptor B3	19	BCRSignalingPathway
LIMS1	LIM zinc finger domain containing 1	2	Antimicrobials
LMBR1	limb development membrane protein 1	7	Antimicrobials
LMBR1L	limb development membrane protein 1 like	12	Antimicrobials
LPA	lipoprotein(a)	6	Antimicrobials
LRP1	LDL receptor related protein 1	12	Antimicrobials
LRSAM1	leucine rich repeat and sterile alpha motif containing 1	9	Cytokines
LTA	lymphotoxin alpha	6	Antigen_Processing_and_Presentation
LTB	lymphotoxin beta	6	Cytokines
LTB4R	leukotriene B4 receptor	14	Antimicrobials
LTB4R2	leukotriene B4 receptor 2	14	Chemokine_Receptors
LTBP1	latent transforming growth factor beta binding protein 1	2	Antimicrobials
LTBP2	latent transforming growth factor beta binding protein 2	14	Cytokines
LTBP3	latent transforming growth factor beta binding protein 3	11	Cytokines
LTBP4	latent transforming growth factor beta binding protein 4	19	Cytokines
LTBR	lymphotoxin beta receptor	12	Cytokine_Receptors
LTF	lactotransferrin	3	Antimicrobials
LYN	LYN proto-oncogene, Src family tyrosine kinase	8	BCRSignalingPathway
LYZ	lysozyme	12	Antimicrobials
MALT1	MALT1 paracaspase	18	BCRSignalingPathway
MANF	mesencephalic astrocyte derived neurotrophic factor	3	Cytokines
MAP2K1	mitogen-activated protein kinase kinase 1	15	Antimicrobials
MAP2K2	mitogen-activated protein kinase kinase 2	19	Antimicrobials
MAP3K14	mitogen-activated protein kinase kinase kinase 14	17	TCRsignalingPathway
MAP3K8	mitogen-activated protein kinase kinase kinase 8	10	TCRsignalingPathway
MAPK1	mitogen-activated protein kinase 1	22	Antimicrobials
MAPK14	mitogen-activated protein kinase 14	6	Antimicrobials
MAPK3	mitogen-activated protein kinase 3	16	Antimicrobials
MAPK8	mitogen-activated protein kinase 8	10	Antimicrobials

MAPT	microtubule associated protein tau	17	Antimicrobials
MARCO	macrophage receptor with collagenous structure	2	Antimicrobials
MASP1	mannan binding lectin serine peptidase 1	3	Antimicrobials
MASP2	mannan binding lectin serine peptidase 2	1	Antimicrobials
MAVS	mitochondrial antiviral signaling protein	20	Antimicrobials
MBL2	mannose binding lectin 2	10	Antimicrobials
MBL3P	mannose-binding lectin family member 3, pseudogene	10	Antimicrobials
MC1R	melanocortin 1 receptor	16	Cytokine_Receptors
MC2R	melanocortin 2 receptor	18	Cytokine_Receptors
MC3R	melanocortin 3 receptor	20	Cytokine_Receptors
MC4R	melanocortin 4 receptor	18	Cytokine_Receptors
MCHR1	melanin concentrating hormone receptor 1	22	Cytokine_Receptors
MCHR2	melanin concentrating hormone receptor 2	6	Cytokine_Receptors
MDK	midkine	11	Cytokines
MET	MET proto-oncogene, receptor tyrosine kinase	7	Cytokine_Receptors
MIA	MIA SH3 domain containing	19	Cytokines
MICA	MHC class I polypeptide-related sequence A	6	Antigen_Processing_and_Presentation
MICB	MHC class I polypeptide-related sequence B	6	Antigen_Processing_and_Presentation
MIF	macrophage migration inhibitory factor	22	Antimicrobials
MLN	motilin	6	Cytokines
MLNR	motilin receptor	13	Cytokine_Receptors
MMP12	matrix metallopeptidase 12	11	Antimicrobials
MMP9	matrix metallopeptidase 9	20	Antimicrobials
MPL	MPL proto-oncogene, thrombopoietin receptor	1	Cytokine_Receptors
MPO	myeloperoxidase	17	Antimicrobials
MR1	major histocompatibility complex, class I-related	1	Antigen_Processing_and_Presentation
MSR1	macrophage scavenger receptor 1	8	Antimicrobials
MSTN	myostatin	2	Cytokines
MTNR1A	melatonin receptor 1A	4	Cytokine_Receptors

MTNR1B	melatonin receptor 1B	11	Cytokine_Receptors
MUC4	mucin 4, cell surface associated	3	Antimicrobials
MUC5AC	mucin 5AC, oligomeric mucus/gel-forming	11	Antimicrobials
MX1	MX dynamin like GTPase 1	21	Antimicrobials
MX2	MX dynamin like GTPase 2	21	Antimicrobials
MYDGF	myeloid derived growth factor	19	Cytokines
NAMPT	nicotinamide phosphoribosyltransferase	7	Cytokines
NCK1	NCK adaptor protein 1	3	TCRsignalingPathway
NCK2	NCK adaptor protein 2	2	TCRsignalingPathway
NCR1	natural cytotoxicity triggering receptor 1	19	NaturalKiller_Cell_Cytotoxicity
NCR2	natural cytotoxicity triggering receptor 2	6	NaturalKiller_Cell_Cytotoxicity
NCR3	natural cytotoxicity triggering receptor 3	6	NaturalKiller_Cell_Cytotoxicity
NDP	norrin cystine knot growth factor NDP	X	Cytokines
NDRG1	N-myc downstream regulated 1	8	Antimicrobials
NEDD4	NEDD4 E3 ubiquitin protein ligase	15	Antimicrobials
NENF	neudesin neurotrophic factor	1	Cytokines
NEO1	neogenin 1	15	Antimicrobials
NEWENTRY	-	-	Antimicrobials
NFAT5	nuclear factor of activated T cells 5	16	BCRSignalingPathway
NFATC1	nuclear factor of activated T cells 1	18	BCRSignalingPathway
NFATC2	nuclear factor of activated T cells 2	20	BCRSignalingPathway
NFATC3	nuclear factor of activated T cells 3	16	BCRSignalingPathway
NFATC4	nuclear factor of activated T cells 4	14	BCRSignalingPathway
NFKB1	nuclear factor kappa B subunit 1	4	Antimicrobials
NFKBIA	NFKB inhibitor alpha	14	BCRSignalingPathway
NFKBIB	NFKB inhibitor beta	19	BCRSignalingPathway
NFKBIE	NFKB inhibitor epsilon	6	BCRSignalingPathway
NFKBIZ	NFKB inhibitor zeta	3	Antimicrobials
NFYA	nuclear transcription factor Y subunit alpha	6	Antigen_Processing_and_Presentation
NFYB	nuclear transcription factor Y subunit beta	12	Antigen_Processing_and_Presentation

NFYC	nuclear transcription factor Y subunit gamma	1	Antigen_Processing_and_Presentation
NGF	nerve growth factor	1	Cytokines
NGFR	nerve growth factor receptor	17	Cytokine_Receptors
NLRX1	NLR family member X1	11	Antimicrobials
NMB	neuromedin B	15	Cytokines
NMBR	neuromedin B receptor	6	Cytokine_Receptors
NOD1	nucleotide binding oligomerization domain containing 1	7	Antimicrobials
NOD2	nucleotide binding oligomerization domain containing 2	16	Antimicrobials
NODAL	nodal growth differentiation factor	10	Cytokines
NOS1	nitric oxide synthase 1	12	Antimicrobials
NOS2	nitric oxide synthase 2	17	Antimicrobials
NOX1	NADPH oxidase 1	X	Antimicrobials
NOX3	NADPH oxidase 3	6	Antimicrobials
NOX4	NADPH oxidase 4	11	Antimicrobials
NOX5	NADPH oxidase 5	15	Antimicrobials
NPFF	neuropeptide FF-amide peptide precursor	12	Cytokines
NPPA	natriuretic peptide A	1	Cytokines
NPPB	natriuretic peptide B	1	Cytokines
NPPC	natriuretic peptide C	2	Cytokines
NPR1	natriuretic peptide receptor 1	1	Cytokine_Receptors
NPR3	natriuretic peptide receptor 3	5	Cytokine_Receptors
NPY	neuropeptide Y	7	Cytokines
NR0B1	nuclear receptor subfamily 0 group B member 1	X	Cytokine_Receptors
NR0B2	nuclear receptor subfamily 0 group B member 2	1	Cytokine_Receptors
NR1D1	nuclear receptor subfamily 1 group D member 1	17	Cytokine_Receptors
NR1D2	nuclear receptor subfamily 1 group D member 2	3	Cytokine_Receptors
NR1H2	nuclear receptor subfamily 1 group H member 2	19	Cytokine_Receptors
NR1H3	nuclear receptor subfamily 1 group H member 3	11	Cytokine_Receptors
NR1H4	nuclear receptor subfamily 1 group H member 4	12	Cytokine_Receptors
NR1I2	nuclear receptor subfamily 1 group I member 2	3	Cytokine_Receptors
NR1I3	nuclear receptor subfamily 1 group I member 3	1	Cytokine_Receptors

NR2C1	nuclear receptor subfamily 2 group C member 1	12	Cytokine_Receptors
NR2C2	nuclear receptor subfamily 2 group C member 2	3	Cytokine_Receptors
NR2E1	nuclear receptor subfamily 2 group E member 1	6	Cytokine_Receptors
NR2E3	nuclear receptor subfamily 2 group E member 3	15	Cytokine_Receptors
NR2F1	nuclear receptor subfamily 2 group F member 1	5	Cytokine_Receptors
NR2F2	nuclear receptor subfamily 2 group F member 2	15	Cytokine_Receptors
NR2F6	nuclear receptor subfamily 2 group F member 6	19	Cytokine_Receptors
NR3C1	nuclear receptor subfamily 3 group C member 1	5	Cytokine_Receptors
NR3C2	nuclear receptor subfamily 3 group C member 2	4	Cytokine_Receptors
NR4A1	nuclear receptor subfamily 4 group A member 1	12	Cytokine_Receptors
NR4A2	nuclear receptor subfamily 4 group A member 2	2	Cytokine_Receptors
NR4A3	nuclear receptor subfamily 4 group A member 3	9	Cytokine_Receptors
NR5A1	nuclear receptor subfamily 5 group A member 1	9	Cytokine_Receptors
NR5A2	nuclear receptor subfamily 5 group A member 2	1	Cytokine_Receptors
NR6A1	nuclear receptor subfamily 6 group A member 1	9	Cytokine_Receptors
NRAS	NRAS proto-oncogene, GTPase	1	BCRSignalingPathway
NRG1	neuregulin 1	8	Cytokines
NRG2	neuregulin 2	5	Cytokines
NRG3	neuregulin 3	10	Cytokines
NRG4	neuregulin 4	15	Cytokines
NRP1	neuropilin 1	10	Cytokine_Receptors
NRP2	neuropilin 2	2	Cytokine_Receptors
NRTN	neurturin	19	Cytokines
NTF3	neurotrophin 3	12	Cytokines
NTF4	neurotrophin 4	19	Cytokines
NTS	neurotensin	12	Cytokines
NUDT6	nudix hydrolase 6	4	Cytokines
OAS1	2'-5'-oligoadenylate synthetase 1	12	Antimicrobials
OASL	2'-5'-oligoadenylate synthetase like	12	Antimicrobials
OBP2A	odorant binding protein 2A	9	Antimicrobials
OBP2B	odorant binding protein 2B	9	Antimicrobials
OGFR	opioid growth factor receptor	20	Cytokine_Receptors

OGN	osteoglycin	9	Cytokines
OLR1	oxidized low density lipoprotein receptor 1	12	Antimicrobials
OPRD1	opioid receptor delta 1	1	Cytokine_Receptors
OPRK1	opioid receptor kappa 1	8	Cytokine_Receptors
OPRL1	opioid related nociceptin receptor 1	20	Cytokine_Receptors
OPRM1	opioid receptor mu 1	6	Cytokine_Receptors
ORM1	orosomucoid 1	9	Antimicrobials
ORM2	orosomucoid 2	9	Antimicrobials
OSGIN1	oxidative stress induced growth inhibitor 1	16	Cytokines
OSM	oncostatin M	22	Cytokines
OSMR	oncostatin M receptor	5	Cytokine_Receptors
OSTN	osteocrin	3	Cytokines
OXT	oxytocin/neurophysin I prepropeptide	20	Cytokines
OXTR	oxytocin receptor	3	Cytokine_Receptors
PAEP	progestagen associated endometrial protein	9	Antimicrobials
PAK1	p21 (RAC1) activated kinase 1	11	NaturalKiller_Cell_Cytotoxicity
PAK2	p21 (RAC1) activated kinase 2	3	TCRsignalingPathway
PAK3	p21 (RAC1) activated kinase 3	X	TCRsignalingPathway
PAK4	p21 (RAC1) activated kinase 4	19	TCRsignalingPathway
PAK5	p21 (RAC1) activated kinase 5	20	TCRsignalingPathway
PAK6	p21 (RAC1) activated kinase 6	15	TCRsignalingPathway
PCSK1	proprotein convertase subtilisin/kexin type 1	5	Antimicrobials
PCSK2	proprotein convertase subtilisin/kexin type 2	20	Antimicrobials
PDCD1	programmed cell death 1	2	Antimicrobials
PDF	peptide deformylase, mitochondrial	16	Antimicrobials
PDGFA	platelet derived growth factor subunit A	7	Cytokines
PDGFB	platelet derived growth factor subunit B	22	Cytokines
PDGFC	platelet derived growth factor C	4	Cytokines
PDGFD	platelet derived growth factor D	11	Cytokines
PDGFRA	platelet derived growth factor receptor alpha	4	Antimicrobials
PDGFRB	platelet derived growth factor receptor beta	5	Antimicrobials
PDGFRL	platelet derived growth factor receptor like	8	Cytokines

PDIA2	protein disulfide isomerase family A member 2	16	Antigen_Processing_and_Presentation
PDIA3	protein disulfide isomerase family A member 3	15	Antigen_Processing_and_Presentation
PDK1	pyruvate dehydrogenase kinase 1	2	TCRsignalingPathway
PDYN	prodynorphin	20	Antimicrobials
PENK	proenkephalin	8	Antimicrobials
PF4	platelet factor 4	4	Antimicrobials
PF4V1	platelet factor 4 variant 1	4	Antimicrobials
PGC	progastricsin	6	Antimicrobials
PGF	placental growth factor	14	Cytokines
PGLYRP1	peptidoglycan recognition protein 1	19	Antimicrobials
PGLYRP2	peptidoglycan recognition protein 2	19	Antimicrobials
PGLYRP3	peptidoglycan recognition protein 3	1	Antimicrobials
PGLYRP4	peptidoglycan recognition protein 4	1	Antimicrobials
PGR	progesterone receptor	11	Cytokine_Receptors
PGRMC2	progesterone receptor membrane component 2	4	Cytokine_Receptors
PI15	peptidase inhibitor 15	8	Antimicrobials
PI3	peptidase inhibitor 3	20	Antimicrobials
PIK3CA	phosphatidylinositol-4,5-bisphosphate 3-kinase catalytic subunit alpha	3	BCRSignalingPathway
PIK3CB	phosphatidylinositol-4,5-bisphosphate 3-kinase catalytic subunit beta	3	BCRSignalingPathway
PIK3CD	phosphatidylinositol-4,5-bisphosphate 3-kinase catalytic subunit delta	1	BCRSignalingPathway
PIK3CG	phosphatidylinositol-4,5-bisphosphate 3-kinase catalytic subunit gamma	7	Antimicrobials
PIK3R1	phosphoinositide-3-kinase regulatory subunit 1	5	BCRSignalingPathway
PIK3R2	phosphoinositide-3-kinase regulatory subunit 2	19	BCRSignalingPathway
PIK3R3	phosphoinositide-3-kinase regulatory subunit 3	1	BCRSignalingPathway
PIK3R5	phosphoinositide-3-kinase regulatory subunit 5	17	BCRSignalingPathway
PLA2G2A	phospholipase A2 group IIA	1	Antimicrobials
PLAAT4	phospholipase A and acyltransferase 4	11	Antimicrobials
PLAU	plasminogen activator, urokinase	10	Antimicrobials
PLAUR	plasminogen activator, urokinase receptor	19	Chemokine_Receptors

PLCG1	phospholipase C gamma 1	20	NaturalKiller_Cell_Cytotoxicity
PLCG2	phospholipase C gamma 2	16	BCRSignalingPathway
PLSCR1	phospholipid scramblase 1	3	Antimicrobials
PLTP	phospholipid transfer protein	20	Antimicrobials
PLXNA1	plexin A1	3	Chemokine_Receptors
PLXNA2	plexin A2	1	Chemokine_Receptors
PLXNA3	plexin A3	X	Chemokine_Receptors
PLXNA4	plexin A4	7	Chemokine_Receptors
PLXNB1	plexin B1	3	Chemokine_Receptors
PLXNB2	plexin B2	22	Chemokine_Receptors
PLXNB3	plexin B3	X	Chemokine_Receptors
PLXNC1	plexin C1	12	Chemokine_Receptors
PLXND1	plexin D1	3	Chemokine_Receptors
PMCH	pro-melanin concentrating hormone	12	Cytokines
PML	PML nuclear body scaffold	15	Antimicrobials
PMP2	peripheral myelin protein 2	8	Antimicrobials
PNOC	prepronociceptin	8	Cytokines
POMC	proopiomelanocortin	2	Cytokines
PPARA	peroxisome proliferator activated receptor alpha	22	Cytokine_Receptors
PPARD	peroxisome proliferator activated receptor delta	6	Cytokine_Receptors
PPARG	peroxisome proliferator activated receptor gamma	3	Antimicrobials
PPBP	pro-platelet basic protein	4	Antimicrobials
PPBPP1	pro-platelet basic protein pseudogene 1	4	Chemokines
PPBPP2	pro-platelet basic protein pseudogene 2	4	Cytokines
PPIA	peptidylprolyl isomerase A	7	Antimicrobials
PPP3CA	protein phosphatase 3 catalytic subunit alpha	4	BCRSignalingPathway
PPP3CB	protein phosphatase 3 catalytic subunit beta	10	BCRSignalingPathway
PPP3CC	protein phosphatase 3 catalytic subunit gamma	8	BCRSignalingPathway
PPP3R1	protein phosphatase 3 regulatory subunit B, alpha	2	BCRSignalingPathway
PPP3R2	protein phosphatase 3 regulatory subunit B, beta	9	BCRSignalingPathway
PPP4C	protein phosphatase 4 catalytic subunit	16	Antimicrobials
PPY	pancreatic polypeptide	17	Cytokines

PRDX1	peroxiredoxin 1	1	Antimicrobials
PRDX2	peroxiredoxin 2	19	Antimicrobials
PRF1	perforin 1	10	NaturalKiller_Cell_Cytotoxicity
PRKCA	protein kinase C alpha	17	NaturalKiller_Cell_Cytotoxicity
PRKCB	protein kinase C beta	16	BCRSignalingPathway
PRKCG	protein kinase C gamma	19	NaturalKiller_Cell_Cytotoxicity
PRKCQ	protein kinase C theta	10	TCRsignalingPathway
PRL	prolactin	6	Cytokines
PRLH	prolactin releasing hormone	2	Cytokines
PRLHR	prolactin releasing hormone receptor	10	Cytokine_Receptors
PRLR	prolactin receptor	5	Cytokine_Receptors
PROC	protein C, inactivator of coagulation factors Va and VIIIa	2	Antimicrobials
PROCR	protein C receptor	20	Antigen_Processing_and_Presentation
PROK1	prokineticin 1	1	Cytokines
PROK2	prokineticin 2	3	Chemokines
PRTN3	proteinase 3	19	Antimicrobials
PSMB8	proteasome 20S subunit beta 8	6	Antigen_Processing_and_Presentation
PSMC1	proteasome 26S subunit, ATPase 1	14	Antigen_Processing_and_Presentation
PSMC2	proteasome 26S subunit, ATPase 2	7	Antigen_Processing_and_Presentation
PSMC3	proteasome 26S subunit, ATPase 3	11	Antigen_Processing_and_Presentation
PSMC4	proteasome 26S subunit, ATPase 4	19	Antigen_Processing_and_Presentation
PSMC5	proteasome 26S subunit, ATPase 5	17	Antigen_Processing_and_Presentation
PSMC6	proteasome 26S subunit, ATPase 6	14	Antigen_Processing_and_Presentation
PSMD1	proteasome 26S subunit, non-ATPase 1	2	Antigen_Processing_and_Presenta

			tion
PSMD10	proteasome 26S subunit, non-ATPase 10	X	Antigen_Processing_and_Presentation
PSMD11	proteasome 26S subunit, non-ATPase 11	17	Antigen_Processing_and_Presentation
PSMD13	proteasome 26S subunit, non-ATPase 13	11	Antigen_Processing_and_Presentation
PSMD14	proteasome 26S subunit, non-ATPase 14	2	Antigen_Processing_and_Presentation
PSMD2	proteasome 26S subunit, non-ATPase 2	3	Antigen_Processing_and_Presentation
PSMD3	proteasome 26S subunit, non-ATPase 3	17	Antigen_Processing_and_Presentation
PSMD4	proteasome 26S subunit, non-ATPase 4	1	Antigen_Processing_and_Presentation
PSMD5	proteasome 26S subunit, non-ATPase 5	9	Antigen_Processing_and_Presentation
PSMD6	proteasome 26S subunit, non-ATPase 6	3	Antigen_Processing_and_Presentation
PSMD7	proteasome 26S subunit, non-ATPase 7	16	Antigen_Processing_and_Presentation
PSMD8	proteasome 26S subunit, non-ATPase 8	19	Antigen_Processing_and_Presentation
PSME1	proteasome activator subunit 1	14	Antigen_Processing_and_Presentation
PSME2	proteasome activator subunit 2	14	Antigen_Processing_and_Presentation
PSME3	proteasome activator subunit 3	17	Antigen_Processing_and_Presentation
PSPN	persephin	19	Cytokines
PTAFR	platelet activating factor receptor	1	Chemokine_Receptors
PTGDR	prostaglandin D2 receptor	14	Antimicrobials

PTGDR2	prostaglandin D2 receptor 2	11	Chemokine_Receptors
PTGDS	prostaglandin D2 synthase	9	Antimicrobials
PTGER1	prostaglandin E receptor 1	19	Cytokine_Receptors
PTGER2	prostaglandin E receptor 2	14	Cytokine_Receptors
PTGER3	prostaglandin E receptor 3	1	Cytokine_Receptors
PTGER4	prostaglandin E receptor 4	5	Cytokine_Receptors
PTGFR	prostaglandin F receptor	1	Cytokine_Receptors
PTGS2	prostaglandin-endoperoxide synthase 2	1	Antimicrobials
PTH	parathyroid hormone	11	Cytokines
PTH1R	parathyroid hormone 1 receptor	3	Cytokine_Receptors
PTH2	parathyroid hormone 2	19	Cytokines
PTH2R	parathyroid hormone 2 receptor	2	Cytokine_Receptors
PTHLH	parathyroid hormone like hormone	12	Cytokines
PTK2	protein tyrosine kinase 2	8	Antimicrobials
PTK2B	protein tyrosine kinase 2 beta	8	Antimicrobials
PTN	pleiotrophin	7	Cytokines
PTPN11	protein tyrosine phosphatase non-receptor type 11	12	NaturalKiller_Cell_Cytotoxicity
PTPN6	protein tyrosine phosphatase non-receptor type 6	12	BCRSignalingPathway
PTPRC	protein tyrosine phosphatase receptor type C	1	TCRsignalingPathway
PTX3	pentraxin 3	3	Antimicrobials
PYY	peptide YY	17	Cytokines
QRFP	pyroglutamylated RFamide peptide	9	Cytokines
R3HDML	R3H domain containing like	20	Antimicrobials
RABEP1	rabaptin, RAB GTPase binding effector protein 1	17	Cytokines
RABEP2	rabaptin, RAB GTPase binding effector protein 2	16	Cytokines
RAC1	Rac family small GTPase 1	7	BCRSignalingPathway
RAC2	Rac family small GTPase 2	22	BCRSignalingPathway
RAC3	Rac family small GTPase 3	17	BCRSignalingPathway
RAET1E	retinoic acid early transcript 1E	6	Antigen_Processing_and_Presentation
RAET1G	retinoic acid early transcript 1G	6	Antigen_Processing_and_Presentation

RAET1L	retinoic acid early transcript 1L	6	Antigen_Processing_and_Presentation
RAF1	Raf-1 proto-oncogene, serine/threonine kinase	3	NaturalKiller_Cell_Cytotoxicity
RARA	retinoic acid receptor alpha	17	Cytokine_Receptors
RARB	retinoic acid receptor beta	3	Cytokine_Receptors
RARG	retinoic acid receptor gamma	12	Cytokine_Receptors
RASGRP1	RAS guanyl releasing protein 1	15	TCRsignalingPathway
RASGRP3	RAS guanyl releasing protein 3	2	BCRSignalingPathway
RBP1	retinol binding protein 1	3	Antimicrobials
RBP2	retinol binding protein 2	3	Antimicrobials
RBP4	retinol binding protein 4	10	Antimicrobials
RBP5	retinol binding protein 5	12	Antimicrobials
RBP7	retinol binding protein 7	1	Antimicrobials
REG1A	regenerating family member 1 alpha	2	Cytokines
REG3G	regenerating family member 3 gamma	2	Antimicrobials
RELA	RELA proto-oncogene, NF-kB subunit	11	Antimicrobials
RELB	RELB proto-oncogene, NF-kB subunit	19	Antigen_Processing_and_Presentation
RETN	resistin	19	Cytokines
RETNLB	resistin like beta	3	Cytokines
RFX5	regulatory factor X5	1	Antigen_Processing_and_Presentation
RFXANK	regulatory factor X associated ankyrin containing protein	19	Antigen_Processing_and_Presentation
RFXAP	regulatory factor X associated protein	13	Antigen_Processing_and_Presentation
RHOA	ras homolog family member A	3	TCRsignalingPathway
RLN1	relaxin 1	9	Cytokines
RLN2	relaxin 2	9	Cytokines
RLN3	relaxin 3	19	Cytokines
RN7SL1	RNA component of signal recognition particle 7SL1	14	Antimicrobials
RNASE2	ribonuclease A family member 2	14	Antimicrobials

RNASE3	ribonuclease A family member 3	14	Antimicrobials
RNASE7	ribonuclease A family member 7	14	Antimicrobials
RNASEL	ribonuclease L	1	Antimicrobials
ROBO1	roundabout guidance receptor 1	3	Chemokine_Receptors
ROBO2	roundabout guidance receptor 2	3	Chemokine_Receptors
ROBO3	roundabout guidance receptor 3	11	Antimicrobials
RORA	RAR related orphan receptor A	15	Cytokine_Receptors
RORB	RAR related orphan receptor B	9	Cytokine_Receptors
RORC	RAR related orphan receptor C	1	Cytokine_Receptors
RSAD2	radical S-adenosyl methionine domain containing 2	2	Antimicrobials
RXFP1	relaxin family peptide receptor 1	4	Cytokine_Receptors
RXFP2	relaxin family peptide receptor 2	13	Cytokine_Receptors
RXFP3	relaxin family peptide receptor 3	5	Chemokine_Receptors
RXRA	retinoid X receptor alpha	9	Cytokine_Receptors
RXRB	retinoid X receptor beta	6	Cytokine_Receptors
RXRG	retinoid X receptor gamma	1	Cytokine_Receptors
S100A1	S100 calcium binding protein A1	1	Antimicrobials
S100A10	S100 calcium binding protein A10	1	Antimicrobials
S100A11	S100 calcium binding protein A11	1	Antimicrobials
S100A12	S100 calcium binding protein A12	1	Antimicrobials
S100A13	S100 calcium binding protein A13	1	Antimicrobials
S100A14	S100 calcium binding protein A14	1	Antimicrobials
S100A16	S100 calcium binding protein A16	1	Antimicrobials
S100A2	S100 calcium binding protein A2	1	Antimicrobials
S100A3	S100 calcium binding protein A3	1	Antimicrobials
S100A5	S100 calcium binding protein A5	1	Antimicrobials
S100A6	S100 calcium binding protein A6	1	Antimicrobials
S100A7	S100 calcium binding protein A7	1	Antimicrobials
S100A7A	S100 calcium binding protein A7A	1	Antimicrobials
S100A7L2	S100 calcium binding protein A7 like 2	1	Antimicrobials
S100A8	S100 calcium binding protein A8	1	Antimicrobials
S100A9	S100 calcium binding protein A9	1	Antimicrobials

S100B	S100 calcium binding protein B	21	Antimicrobials
S100G	S100 calcium binding protein G	X	Antimicrobials
S100P	S100 calcium binding protein P	4	Antimicrobials
S100Z	S100 calcium binding protein Z	5	Antimicrobials
S1PR1	sphingosine-1-phosphate receptor 1	1	Cytokine_Receptors
S1PR2	sphingosine-1-phosphate receptor 2	19	Cytokine_Receptors
SAA1	serum amyloid A1	11	Chemokines
SAA2	serum amyloid A2	11	Chemokines
SBDS	SBDS ribosome maturation factor	7	Chemokines
SCG2	secretogranin II	2	Cytokines
SCGB3A1	secretoglobin family 3A member 1	5	Cytokines
SCT	secretin	11	Cytokines
SCTR	secretin receptor	2	Cytokine_Receptors
SDC1	syndecan 1	2	Cytokine_Receptors
SDC2	syndecan 2	8	Cytokine_Receptors
SDC3	syndecan 3	1	Cytokine_Receptors
SDC4	syndecan 4	20	Cytokine_Receptors
SECTM1	secreted and transmembrane 1	17	Cytokines
SEM1	SEM1 26S proteasome complex subunit	7	Antigen_Processing_and_Presentation
SEMA3A	semaphorin 3A	7	Chemokines
SEMA3B	semaphorin 3B	3	Chemokines
SEMA3C	semaphorin 3C	7	Chemokines
SEMA3D	semaphorin 3D	7	Chemokines
SEMA3E	semaphorin 3E	7	Chemokines
SEMA3F	semaphorin 3F	3	Chemokines
SEMA3G	semaphorin 3G	3	Chemokines
SEMA4A	semaphorin 4A	1	Chemokines
SEMA4B	semaphorin 4B	15	Chemokines
SEMA4C	semaphorin 4C	2	Chemokines
SEMA4D	semaphorin 4D	9	Chemokines
SEMA4F	ssemaphorin 4F	2	Chemokines

SEMA4G	semaphorin 4G	10	Chemokines
SEMA5A	semaphorin 5A	5	Chemokines
SEMA5B	semaphorin 5B	3	Chemokines
SEMA6A	semaphorin 6A	5	Chemokines
SEMA6B	semaphorin 6B	19	Chemokines
SEMA6C	semaphorin 6C	1	Chemokines
SEMA6D	semaphorin 6D	15	Chemokines
SEMA7A	semaphorin 7A (John Milton Hagen blood group)	15	Chemokines
SEMG1	semenogelin 1	20	Antimicrobials
SEMG2	semenogelin 2	20	Antimicrobials
SEPTIN7	septin 7	7	Antimicrobials
SERPINA3	serpin family A member 3	14	Antimicrobials
SERPIND1	serpin family D member 1	22	Antimicrobials
SFTPA1	surfactant protein A1	10	Antimicrobials
SFTPA2	surfactant protein A2	10	Antimicrobials
SFTPD	surfactant protein D	10	Antimicrobials
SH2D1A	SH2 domain containing 1A	X	NaturalKiller_Cell_Cytotoxicity
SH2D1B	SH2 domain containing 1B	1	NaturalKiller_Cell_Cytotoxicity
SH3BP2	SH3 domain binding protein 2	4	NaturalKiller_Cell_Cytotoxicity
SHC1	SHC adaptor protein 1	1	NaturalKiller_Cell_Cytotoxicity
SHC2	SHC adaptor protein 2	19	NaturalKiller_Cell_Cytotoxicity
SHC3	SHC adaptor protein 3	9	NaturalKiller_Cell_Cytotoxicity
SHC4	SHC adaptor protein 4	15	NaturalKiller_Cell_Cytotoxicity
SKIV2L	Ski2 like RNA helicase	6	Antimicrobials
SLC10A2	solute carrier family 10 member 2	13	Antigen_Processing_and_Presentation
SLC11A1	solute carrier family 11 member 1	2	Antimicrobials
SLC22A17	solute carrier family 22 member 17	14	Antimicrobials
SLC29A3	solute carrier family 29 member 3	10	Antimicrobials
SLC40A1	solute carrier family 40 member 1	2	Antimicrobials
SLIT1	slit guidance ligand 1	10	Chemokines
SLIT2	slit guidance ligand 2	4	Chemokines

SLPI	secretory leukocyte peptidase inhibitor	20	Antimicrobials
SLURP1	secreted LY6/PLAUR domain containing 1	8	Cytokines
SOCS1	suppressor of cytokine signaling 1	16	Antimicrobials
SOCS3	suppressor of cytokine signaling 3	17	Antimicrobials
SOD1	superoxide dismutase 1	21	Antimicrobials
SORT1	sortilin 1	1	Cytokine_Receptors
SOS1	SOS Ras/Rac guanine nucleotide exchange factor 1	2	NaturalKiller_Cell_Cytotoxicity
SOS2	SOS Ras/Rho guanine nucleotide exchange factor 2	14	NaturalKiller_Cell_Cytotoxicity
SP1	Sp1 transcription factor	12	Antimicrobials
SPAG11A	sperm associated antigen 11A	8	Antimicrobials
SPAG11B	sperm associated antigen 11B	8	Antimicrobials
SPINK5	serine peptidase inhibitor Kazal type 5	5	Antimicrobials
SPP1	secreted phosphoprotein 1	4	Cytokines
SRC	SRC proto-oncogene, non-receptor tyrosine kinase	20	Antimicrobials
SST	somatostatin	3	Cytokines
SSTR1	somatostatin receptor 1	14	Cytokine_Receptors
SSTR2	somatostatin receptor 2	17	Cytokine_Receptors
SSTR5	somatostatin receptor 5	16	Cytokine_Receptors
ST2	-	11	Cytokine_Receptors
STAB2	stabilin 2	12	Antimicrobials
STAT1	signal transducer and activator of transcription 1	2	Antimicrobials
STAT3	signal transducer and activator of transcription 3	17	Antimicrobials
STC1	stanniocalcin 1	8	Cytokines
STC2	stanniocalcin 2	5	Cytokines
STING1	stimulator of interferon response cGAMP interactor 1	5	Antimicrobials
SYK	spleen associated tyrosine kinase	9	BCRSignalingPathway
SYTL1	synaptotagmin like 1	1	Antimicrobials
TAC1	tachykinin precursor 1	7	Cytokines
TACR1	tachykinin receptor 1	2	Cytokine_Receptors
TAFA1	TAFA chemokine like family member 1	3	Antimicrobials
TAFA2	TAFA chemokine like family member 2	12	Antimicrobials
TAFA3	TAFA chemokine like family member 3	1	Antimicrobials

TAFA4	TAFA chemokine like family member 4	3	Antimicrobials
TAFA5	TAFA chemokine like family member 5	22	Antimicrobials
TANK	TRAF family member associated NFKB activator	2	Antimicrobials
TAP1	transporter 1, ATP binding cassette subfamily B member	6	Antigen_Processing_and_Presentation
TAP2	transporter 2, ATP binding cassette subfamily B member	6	Antigen_Processing_and_Presentation
TAPBP	TAP binding protein	6	Antigen_Processing_and_Presentation
TAPBPL	TAP binding protein like	12	Antigen_Processing_and_Presentation
TBK1	TANK binding kinase 1	12	Antimicrobials
TCF7L2	transcription factor 7 like 2	10	Antimicrobials
TCHHL1	trichohyalin like 1	1	Antimicrobials
TDGF1	teratocarcinoma-derived growth factor 1	3	Cytokines
TDGF1P3	teratocarcinoma-derived growth factor 1 pseudogene 3	X	Cytokines
TEC	tec protein tyrosine kinase	4	TCRsignalingPathway
TEK	TEK receptor tyrosine kinase	9	Cytokine_Receptors
TFR2	transferrin receptor 2	7	Antimicrobials
TFRC	transferrin receptor	3	Antimicrobials
TG	thyroglobulin	8	Cytokines
TGFA	transforming growth factor alpha	2	Cytokines
TGFB1	transforming growth factor beta 1	19	Antimicrobials
TGFB2	transforming growth factor beta 2	1	Cytokines
TGFB3	transforming growth factor beta 3	14	Cytokines
TGFBR1	transforming growth factor beta receptor 1	9	Cytokine_Receptors
TGFBR2	transforming growth factor beta receptor 2	3	Cytokine_Receptors
TGFBR3	transforming growth factor beta receptor 3	1	Cytokine_Receptors
THBS1	thrombospondin 1	15	Antigen_Processing_and_Presentation
THPO	thrombopoietin	3	Cytokines
THRA	thyroid hormone receptor alpha	17	Cytokine_Receptors

THRB	thyroid hormone receptor beta	3	Cytokine_Receptors
TIE1	tyrosine kinase with immunoglobulin like and EGF like domains 1	1	Cytokine_Receptors
TINAGL1	tubulointerstitial nephritis antigen like 1	1	Antimicrobials
TK2	thymidine kinase 2	16	Antimicrobials
TKFC	triokinase and FMN cyclase	11	Antimicrobials
TLR1	toll like receptor 1	4	Antimicrobials
TLR2	toll like receptor 2	4	Antimicrobials
TLR3	toll like receptor 3	4	Antimicrobials
TLR4	toll like receptor 4	9	Antimicrobials
TLR7	toll like receptor 7	X	Antimicrobials
TLR8	toll like receptor 8	X	Antimicrobials
TMPRSS6	transmembrane serine protease 6	22	Antimicrobials
TMSB10	thymosin beta 10	2	Antimicrobials
TMSB15A	thymosin beta 15a	X	Antimicrobials
TMSB15B	thymosin beta 15B	X	Antimicrobials
TMSB4X	thymosin beta 4 X-linked	X	Antimicrobials
TMSB4XP8	TMSB4X pseudogene 8	4	Antimicrobials
TMSB4Y	thymosin beta 4 Y-linked	Y	Antimicrobials
TNC	tenascin C	9	Chemokines
TNF	tumor necrosis factor	6	Antimicrobials
TNFAIP3	TNF alpha induced protein 3	6	Antimicrobials
TNFRSF10A	TNF receptor superfamily member 10a	8	Antimicrobials
TNFRSF10B	TNF receptor superfamily member 10b	8	Antimicrobials
TNFRSF10C	TNF receptor superfamily member 10c	8	Cytokine_Receptors
TNFRSF10D	TNF receptor superfamily member 10d	8	Cytokine_Receptors
TNFRSF11A	TNF receptor superfamily member 11a	18	Cytokine_Receptors
TNFRSF11B	TNF receptor superfamily member 11b	8	Cytokines
TNFRSF12A	TNF receptor superfamily member 12A	16	Cytokine_Receptors
TNFRSF13B	TNF receptor superfamily member 13B	17	Cytokine_Receptors
TNFRSF13C	TNF receptor superfamily member 13C	22	Cytokine_Receptors
TNFRSF14	TNF receptor superfamily member 14	1	Cytokine_Receptors
TNFRSF17	TNF receptor superfamily member 17	16	Cytokine_Receptors

TNFRSF18	TNF receptor superfamily member 18	1	Cytokine_Receptors
TNFRSF19	TNF receptor superfamily member 19	13	Cytokine_Receptors
TNFRSF1A	TNF receptor superfamily member 1A	12	Cytokine_Receptors
TNFRSF1B	TNF receptor superfamily member 1B	1	Cytokine_Receptors
TNFRSF21	TNF receptor superfamily member 21	6	Cytokine_Receptors
TNFRSF25	TNF receptor superfamily member 25	1	Cytokine_Receptors
TNFRSF4	TNF receptor superfamily member 4	1	Cytokine_Receptors
TNFRSF6B	TNF receptor superfamily member 6b	20	Cytokine_Receptors
TNFRSF8	TNF receptor superfamily member 8	1	Cytokine_Receptors
TNFRSF9	TNF receptor superfamily member 9	1	Cytokine_Receptors
TNFSF10	TNF superfamily member 10	3	Antimicrobials
TNFSF11	TNF superfamily member 11	13	Antimicrobials
TNFSF12	TNF superfamily member 12	17	Cytokines
TNFSF13	TNF superfamily member 13	17	Cytokines
TNFSF13B	TNF superfamily member 13b	13	Cytokines
TNFSF14	TNF superfamily member 14	19	Cytokines
TNFSF15	TNF superfamily member 15	9	Cytokines
TNFSF18	TNF superfamily member 18	1	Cytokines
TNFSF4	TNF superfamily member 4	1	Antimicrobials
TNFSF8	TNF superfamily member 8	9	Cytokines
TNFSF9	TNF superfamily member 9	19	Cytokines
TOR2A	torsin family 2 member A	9	Cytokines
TPM2	tropomyosin 2	9	Antimicrobials
TPT1	tumor protein, translationally-controlled 1	13	Antimicrobials
TRAC	T cell receptor alpha constant	14	TCRsignalingPathway
TRAF3	TNF receptor associated factor 3	14	Antimicrobials
TRAJ1	T cell receptor alpha joining 1 (non-functional)	14	TCRsignalingPathway
TRAJ10	T cell receptor alpha joining 10	14	TCRsignalingPathway
TRAJ11	T cell receptor alpha joining 11	14	TCRsignalingPathway
TRAJ12	T cell receptor alpha joining 12	14	TCRsignalingPathway
TRAJ13	T cell receptor alpha joining 13	14	TCRsignalingPathway
TRAJ14	T cell receptor alpha joining 14	14	TCRsignalingPathway

TRAJ15	T cell receptor alpha joining 15	14	TCRsignalingPathway
TRAJ16	T cell receptor alpha joining 16	14	TCRsignalingPathway
TRAJ17	T cell receptor alpha joining 17	14	TCRsignalingPathway
TRAJ18	T cell receptor alpha joining 18	14	TCRsignalingPathway
TRAJ19	T cell receptor alpha joining 19 (non-functional)	14	TCRsignalingPathway
TRAJ2	T cell receptor alpha joining 2 (non-functional)	14	TCRsignalingPathway
TRAJ20	T cell receptor alpha joining 20	14	TCRsignalingPathway
TRAJ21	T cell receptor alpha joining 21	14	TCRsignalingPathway
TRAJ22	T cell receptor alpha joining 22	14	TCRsignalingPathway
TRAJ23	T cell receptor alpha joining 23	14	TCRsignalingPathway
TRAJ24	T cell receptor alpha joining 24	14	TCRsignalingPathway
TRAJ25	T cell receptor alpha joining 25 (non-functional)	14	TCRsignalingPathway
TRAJ26	T cell receptor alpha joining 26	14	TCRsignalingPathway
TRAJ27	T cell receptor alpha joining 27	14	TCRsignalingPathway
TRAJ28	T cell receptor alpha joining 28	14	TCRsignalingPathway
TRAJ29	T cell receptor alpha joining 29	14	TCRsignalingPathway
TRAJ3	T cell receptor alpha joining 3	14	TCRsignalingPathway
TRAJ30	T cell receptor alpha joining 30	14	TCRsignalingPathway
TRAJ31	T cell receptor alpha joining 31	14	TCRsignalingPathway
TRAJ32	T cell receptor alpha joining 32	14	TCRsignalingPathway
TRAJ33	T cell receptor alpha joining 33	14	TCRsignalingPathway
TRAJ34	T cell receptor alpha joining 34	14	TCRsignalingPathway
TRAJ35	T cell receptor alpha joining 35 (non-functional)	14	TCRsignalingPathway
TRAJ36	T cell receptor alpha joining 36	14	TCRsignalingPathway
TRAJ37	T cell receptor alpha joining 37	14	TCRsignalingPathway
TRAJ38	T cell receptor alpha joining 38	14	TCRsignalingPathway
TRAJ39	T cell receptor alpha joining 39	14	TCRsignalingPathway
TRAJ4	T cell receptor alpha joining 4	14	TCRsignalingPathway
TRAJ40	T cell receptor alpha joining 40	14	TCRsignalingPathway
TRAJ41	T cell receptor alpha joining 41	14	TCRsignalingPathway
TRAJ42	T cell receptor alpha joining 42	14	TCRsignalingPathway
TRAJ43	T cell receptor alpha joining 43	14	TCRsignalingPathway

TRAJ44	T cell receptor alpha joining 44	14	TCRsignalingPathway
TRAJ45	T cell receptor alpha joining 45	14	TCRsignalingPathway
TRAJ46	T cell receptor alpha joining 46	14	TCRsignalingPathway
TRAJ47	T cell receptor alpha joining 47	14	TCRsignalingPathway
TRAJ48	T cell receptor alpha joining 48	14	TCRsignalingPathway
TRAJ49	T cell receptor alpha joining 49	14	TCRsignalingPathway
TRAJ5	T cell receptor alpha joining 5	14	TCRsignalingPathway
TRAJ50	T cell receptor alpha joining 50	14	TCRsignalingPathway
TRAJ52	T cell receptor alpha joining 52	14	TCRsignalingPathway
TRAJ53	T cell receptor alpha joining 53	14	TCRsignalingPathway
TRAJ54	T cell receptor alpha joining 54	14	TCRsignalingPathway
TRAJ56	T cell receptor alpha joining 56	14	TCRsignalingPathway
TRAJ57	T cell receptor alpha joining 57	14	TCRsignalingPathway
TRAJ58	T cell receptor alpha joining 58 (non-functional)	14	TCRsignalingPathway
TRAJ59	T cell receptor alpha joining 59 (non-functional)	14	TCRsignalingPathway
TRAJ6	T cell receptor alpha joining 6	14	TCRsignalingPathway
TRAJ61	T cell receptor alpha joining 61 (non-functional)	14	TCRsignalingPathway
TRAJ7	T cell receptor alpha joining 7	14	TCRsignalingPathway
TRAJ8	T cell receptor alpha joining 8	14	TCRsignalingPathway
TRAJ9	T cell receptor alpha joining 9	14	TCRsignalingPathway
TRAV10	T cell receptor alpha variable 10	14	TCRsignalingPathway
TRAV1-1	T cell receptor alpha variable 1-1	14	TCRsignalingPathway
TRAV1-2	T cell receptor alpha variable 1-2	14	TCRsignalingPathway
TRAV12-1	T cell receptor alpha variable 12-1	14	TCRsignalingPathway
TRAV12-2	T cell receptor alpha variable 12-2	14	TCRsignalingPathway
TRAV12-3	T cell receptor alpha variable 12-3	14	TCRsignalingPathway
TRAV13-1	T cell receptor alpha variable 13-1	14	TCRsignalingPathway
TRAV13-2	T cell receptor alpha variable 13-2	14	TCRsignalingPathway
TRAV14DV4	T cell receptor alpha variable 14/delta variable 4	14	TCRsignalingPathway
TRAV16	T cell receptor alpha variable 16	14	TCRsignalingPathway
TRAV17	T cell receptor alpha variable 17	14	TCRsignalingPathway
TRAV18	T cell receptor alpha variable 18	14	TCRsignalingPathway

TRAV19	T cell receptor alpha variable 19	14	TCRsignalingPathway
TRAV2	T cell receptor alpha variable 2	14	TCRsignalingPathway
TRAV20	T cell receptor alpha variable 20	14	TCRsignalingPathway
TRAV21	T cell receptor alpha variable 21	14	TCRsignalingPathway
TRAV22	T cell receptor alpha variable 22	14	TCRsignalingPathway
TRAV23DV6	T cell receptor alpha variable 23/delta variable 6	14	TCRsignalingPathway
TRAV24	T cell receptor alpha variable 24	14	TCRsignalingPathway
TRAV25	T cell receptor alpha variable 25	14	TCRsignalingPathway
TRAV26-1	T cell receptor alpha variable 26-1	14	TCRsignalingPathway
TRAV26-2	T cell receptor alpha variable 26-2	14	TCRsignalingPathway
TRAV27	T cell receptor alpha variable 27	14	TCRsignalingPathway
TRAV29DV5	T cell receptor alpha variable 29/delta variable 5	14	TCRsignalingPathway
TRAV3	T cell receptor alpha variable 3	14	TCRsignalingPathway
TRAV30	T cell receptor alpha variable 30	14	TCRsignalingPathway
TRAV34	T cell receptor alpha variable 34	14	TCRsignalingPathway
TRAV35	T cell receptor alpha variable 35	14	TCRsignalingPathway
TRAV36DV7	T cell receptor alpha variable 36/delta variable 7	14	TCRsignalingPathway
TRAV38-1	T cell receptor alpha variable 38-1	14	TCRsignalingPathway
TRAV38-2DV8	T cell receptor alpha variable 38-2/delta variable 8	14	TCRsignalingPathway
TRAV39	T cell receptor alpha variable 39	14	TCRsignalingPathway
TRAV4	T cell receptor alpha variable 4	14	TCRsignalingPathway
TRAV40	T cell receptor alpha variable 40	14	TCRsignalingPathway
TRAV41	T cell receptor alpha variable 41	14	TCRsignalingPathway
TRAV5	T cell receptor alpha variable 5	14	TCRsignalingPathway
TRAV6	T cell receptor alpha variable 6	14	TCRsignalingPathway
TRAV7	T cell receptor alpha variable 7	14	TCRsignalingPathway
TRAV8-1	T cell receptor alpha variable 8-1	14	TCRsignalingPathway
TRAV8-2	T cell receptor alpha variable 8-2	14	TCRsignalingPathway
TRAV8-3	T cell receptor alpha variable 8-3	14	TCRsignalingPathway
TRAV8-4	T cell receptor alpha variable 8-4	14	TCRsignalingPathway
TRAV8-6	T cell receptor alpha variable 8-6	14	TCRsignalingPathway

TRAV8-7	T cell receptor alpha variable 8-7 (pseudogene)	14	TCRsignalingPathway
TRAV9-1	T cell receptor alpha variable 9-1	14	TCRsignalingPathway
TRAV9-2	T cell receptor alpha variable 9-2	14	TCRsignalingPathway
TRBC1	T cell receptor beta constant 1	7	TCRsignalingPathway
TRBC2	T cell receptor beta constant 2	7	TCRsignalingPathway
TRBD1	T cell receptor beta diversity 1	7	TCRsignalingPathway
TRBD2	T cell receptor beta diversity 2	7	TCRsignalingPathway
TRBJ1-1	T cell receptor beta joining 1-1	7	TCRsignalingPathway
TRBJ1-2	T cell receptor beta joining 1-2	7	TCRsignalingPathway
TRBJ1-3	T cell receptor beta joining 1-3	7	TCRsignalingPathway
TRBJ1-4	T cell receptor beta joining 1-4	7	TCRsignalingPathway
TRBJ1-5	T cell receptor beta joining 1-5	7	TCRsignalingPathway
TRBJ1-6	T cell receptor beta joining 1-6	7	TCRsignalingPathway
TRBJ2-1	T cell receptor beta joining 2-1	7	TCRsignalingPathway
TRBJ2-2	T cell receptor beta joining 2-2	7	TCRsignalingPathway
TRBJ2-3	T cell receptor beta joining 2-3	7	TCRsignalingPathway
TRBJ2-4	T cell receptor beta joining 2-4	7	TCRsignalingPathway
TRBJ2-5	T cell receptor beta joining 2-5	7	TCRsignalingPathway
TRBJ2-6	T cell receptor beta joining 2-6	7	TCRsignalingPathway
TRBJ2-7	T cell receptor beta joining 2-7	7	TCRsignalingPathway
TRBV10-1	T cell receptor beta variable 10-1	7	TCRsignalingPathway
TRBV10-2	T cell receptor beta variable 10-2	7	TCRsignalingPathway
TRBV10-3	T cell receptor beta variable 10-3	7	TCRsignalingPathway
TRBV11-1	T cell receptor beta variable 11-1	7	TCRsignalingPathway
TRBV11-2	T cell receptor beta variable 11-2	7	TCRsignalingPathway
TRBV11-3	T cell receptor beta variable 11-3	7	TCRsignalingPathway
TRBV12-3	T cell receptor beta variable 12-3	7	TCRsignalingPathway
TRBV12-4	T cell receptor beta variable 12-4	7	TCRsignalingPathway
TRBV12-5	T cell receptor beta variable 12-5	7	TCRsignalingPathway
TRBV13	T cell receptor beta variable 13	7	TCRsignalingPathway
TRBV14	T cell receptor beta variable 14	7	TCRsignalingPathway
TRBV15	T cell receptor beta variable 15	7	TCRsignalingPathway

TRBV16	T cell receptor beta variable 16	7	TCRsignalingPathway
TRBV17	T cell receptor beta variable 17 (non-functional)	7	TCRsignalingPathway
TRBV18	T cell receptor beta variable 18	7	TCRsignalingPathway
TRBV19	T cell receptor beta variable 19	7	TCRsignalingPathway
TRBV2	T cell receptor beta variable 2	7	TCRsignalingPathway
TRBV20-1	T cell receptor beta variable 20-1	7	TCRsignalingPathway
TRBV24-1	T cell receptor beta variable 24-1	7	TCRsignalingPathway
TRBV25-1	T cell receptor beta variable 25-1	7	TCRsignalingPathway
TRBV27	T cell receptor beta variable 27	7	TCRsignalingPathway
TRBV28	T cell receptor beta variable 28	7	TCRsignalingPathway
TRBV29-1	T cell receptor beta variable 29-1	7	TCRsignalingPathway
TRBV30	T cell receptor beta variable 30	7	TCRsignalingPathway
TRBV3-1	T cell receptor beta variable 3-1	7	TCRsignalingPathway
TRBV4-1	T cell receptor beta variable 4-1	7	TCRsignalingPathway
TRBV4-2	T cell receptor beta variable 4-2	7	TCRsignalingPathway
TRBV4-3	T cell receptor beta variable 4-3	7	TCRsignalingPathway
TRBV5-1	T cell receptor beta variable 5-1	7	TCRsignalingPathway
TRBV5-4	T cell receptor beta variable 5-4	7	TCRsignalingPathway
TRBV5-5	T cell receptor beta variable 5-5	7	TCRsignalingPathway
TRBV5-6	T cell receptor beta variable 5-6	7	TCRsignalingPathway
TRBV5-7	T cell receptor beta variable 5-7 (non-functional)	7	TCRsignalingPathway
TRBV5-8	T cell receptor beta variable 5-8	7	TCRsignalingPathway
TRBV6-1	T cell receptor beta variable 6-1	7	TCRsignalingPathway
TRBV6-2	T cell receptor beta variable 6-2	7	TCRsignalingPathway
TRBV6-3	T cell receptor beta variable 6-3	7	TCRsignalingPathway
TRBV6-4	T cell receptor beta variable 6-4	7	TCRsignalingPathway
TRBV6-5	T cell receptor beta variable 6-5	7	TCRsignalingPathway
TRBV6-6	T cell receptor beta variable 6-6	7	TCRsignalingPathway
TRBV6-7	T cell receptor beta variable 6-7 (non-functional)	7	TCRsignalingPathway
TRBV6-8	T cell receptor beta variable 6-8	7	TCRsignalingPathway
TRBV6-9	T cell receptor beta variable 6-9	7	TCRsignalingPathway
TRBV7-2	T cell receptor beta variable 7-2	7	TCRsignalingPathway

TRBV7-3	T cell receptor beta variable 7-3	7	TCRsignalingPathway
TRBV7-4	T cell receptor beta variable 7-4	7	TCRsignalingPathway
TRBV7-6	T cell receptor beta variable 7-6	7	TCRsignalingPathway
TRBV7-7	T cell receptor beta variable 7-7	7	TCRsignalingPathway
TRBV7-8	T cell receptor beta variable 7-8	7	TCRsignalingPathway
TRBV7-9	T cell receptor beta variable 7-9	7	TCRsignalingPathway
TRBV9	T cell receptor beta variable 9	7	TCRsignalingPathway
TRDC	T cell receptor delta constant	14	TCRsignalingPathway
TRDD1	T cell receptor delta diversity 1	14	TCRsignalingPathway
TRDD2	T cell receptor delta diversity 2	14	TCRsignalingPathway
TRDD3	T cell receptor delta diversity 3	14	TCRsignalingPathway
TRDJ1	T cell receptor delta joining 1	14	TCRsignalingPathway
TRDJ2	T cell receptor delta joining 2	14	TCRsignalingPathway
TRDJ3	T cell receptor delta joining 3	14	TCRsignalingPathway
TRDJ4	T cell receptor delta joining 4	14	TCRsignalingPathway
TRDV1	T cell receptor delta variable 1	14	TCRsignalingPathway
TRDV2	T cell receptor delta variable 2	14	TCRsignalingPathway
TRDV3	T cell receptor delta variable 3	14	TCRsignalingPathway
TRGC1	T cell receptor gamma constant 1	7	TCRsignalingPathway
TRGC2	T cell receptor gamma constant 2	7	TCRsignalingPathway
TRGJ1	T cell receptor gamma joining 1	7	TCRsignalingPathway
TRGJ2	T cell receptor gamma joining 2	7	TCRsignalingPathway
TRGJP	T cell receptor gamma joining P	7	TCRsignalingPathway
TRGJP1	T cell receptor gamma joining P1	7	TCRsignalingPathway
TRGJP2	T cell receptor gamma joining P2	7	TCRsignalingPathway
TRGV2	T cell receptor gamma variable 2	7	TCRsignalingPathway
TRGV3	T cell receptor gamma variable 3	7	TCRsignalingPathway
TRGV4	T cell receptor gamma variable 4	7	TCRsignalingPathway
TRGV5	T cell receptor gamma variable 5	7	TCRsignalingPathway
TRGV8	T cell receptor gamma variable 8	7	TCRsignalingPathway
TRGV9	T cell receptor gamma variable 9	7	TCRsignalingPathway
TRH	thyrotropin releasing hormone	3	Cytokines

TRHR	thyrotropin releasing hormone receptor	8	Cytokine_Receptors
TRIM22	tripartite motif containing 22	11	Antimicrobials
TRIM27	tripartite motif containing 27	6	Antimicrobials
TRIM5	tripartite motif containing 5	11	Antimicrobials
TRPC4AP	transient receptor potential cation channel subfamily C member 4 associated protein	20	Antigen_Processing_and_Presentation
TSHB	thyroid stimulating hormone subunit beta	1	Cytokines
TSHR	thyroid stimulating hormone receptor	14	Cytokine_Receptors
TSLP	thymic stromal lymphopoietin	5	Cytokines
TUBB3	tubulin beta 3 class III	16	Cytokine_Receptors
TXK	TXK tyrosine kinase	4	Antimicrobials
TXLNA	taxilin alpha	1	Cytokines
TYK2	tyrosine kinase 2	19	Antimicrobials
TYMP	thymidine phosphorylase	22	Chemokines
TYROBP	transmembrane immune signaling adaptor TYROBP	19	NaturalKiller_Cell_Cytotoxicity
UBR1	ubiquitin protein ligase E3 component n-recognin 1	15	Antigen_Processing_and_Presentation
UBXN1	UBX domain protein 1	11	Antigen_Processing_and_Presentation
UCN	urocortin	2	Cytokines
UCN2	urocortin 2	3	Cytokines
UCN3	urocortin 3	10	Cytokines
ULBP1	UL16 binding protein 1	6	Antigen_Processing_and_Presentation
ULBP2	UL16 binding protein 2	6	Antigen_Processing_and_Presentation
ULBP3	UL16 binding protein 3	6	Antigen_Processing_and_Presentation
UMODL1	uromodulin like 1	21	Antimicrobials
UNC93B1	unc-93 homolog B1, TLR signaling regulator	11	Antimicrobials
UTS2	urotensin 2	1	Cytokines
UTS2B	urotensin 2B	3	Cytokines

VAV1	vav guanine nucleotide exchange factor 1	19	BCRSignalingPathway
VAV2	vav guanine nucleotide exchange factor 2	9	BCRSignalingPathway
VAV3	vav guanine nucleotide exchange factor 3	1	BCRSignalingPathway
VCAM1	vascular cell adhesion molecule 1	1	Antimicrobials
VDR	vitamin D receptor	12	Antimicrobials
VEGFA	vascular endothelial growth factor A	6	Antimicrobials
VEGFB	vascular endothelial growth factor B	11	Cytokines
VEGFC	vascular endothelial growth factor C	4	Cytokines
VEGFD	vascular endothelial growth factor D	X	Cytokines
VGF	VGF nerve growth factor inducible	7	Cytokines
VIM	vimentin	10	Antimicrobials
VIP	vasoactive intestinal peptide	6	Cytokines
VIPR1	vasoactive intestinal peptide receptor 1	3	Cytokine_Receptors
VIPR2	vasoactive intestinal peptide receptor 2	7	Cytokine_Receptors
VTN	vitronectin	17	Antimicrobials
WFDC2	WAP four-disulfide core domain 2	20	Antimicrobials
WFIKKN1	WAP, follistatin/kazal, immunoglobulin, kunitz and netrin domain containing 1	16	Antimicrobials
WNT5A	Wnt family member 5A	3	Antimicrobials
XCL1	X-C motif chemokine ligand 1	1	Antimicrobials
XCL2	X-C motif chemokine ligand 2	1	Antimicrobials
XCR1	X-C motif chemokine receptor 1	3	Chemokine_Receptors
ZAP70	zeta chain of T cell receptor associated protein kinase 70	2	NaturalKiller_Cell_Cytotoxicity
ZC3HAV1	zinc finger CCCH-type containing, antiviral 1	7	Antimicrobials
ZC3HAV1L	zinc finger CCCH-type containing, antiviral 1 like	7	Antimicrobials
ZYX	zyxin	7	Antimicrobials

Table S2. 267 prognostic immune-related genes identified by univariate Cox analysis in the training cohort.

Gene	HR	z	95% CI lower	95% CI upper	P-value
DKK1	1.50375999	5.68162866	1.306345655	1.731007477	1.33E-08
WFDC2	0.75039111	-4.1920261	0.656113488	0.858215582	2.76E-05
BMP5	0.73864136	-4.1519991	0.640217152	0.852196872	3.30E-05
VEGFC	1.35356224	4.14076823	1.172857338	1.562108763	3.46E-05
SHC1	1.36033732	4.00792065	1.17028164	1.581258357	6.13E-05
RFXAP	0.74753535	-3.9691846	0.647487967	0.863041676	7.21E-05
INSL4	1.27720723	3.90388239	1.129567813	1.444143761	9.47E-05
ADM	1.34413163	3.82362647	1.15505706	1.564156354	0.0001315
ARRB1	0.76474248	-3.8210881	0.666448009	0.877534422	0.00013286
FCGRT	0.76627061	-3.8178455	0.66838631	0.878489943	0.00013462
PSMD2	1.32562169	3.80278957	1.146368748	1.532903673	0.00014308
SFTPD	0.77051019	-3.7604393	0.672615062	0.882653374	0.00016962
CD40LG	0.74851421	-3.719569	0.642557349	0.871943221	0.00019956
GPI	1.30284319	3.71134359	1.132967621	1.498189667	0.00020616
LIFR	0.76448181	-3.6946363	0.662971281	0.881535081	0.0002202
VIPR1	0.76105582	-3.6129482	0.656276651	0.882563721	0.00030274
HSPA4	1.32903002	3.5595401	1.136354186	1.554375216	0.0003715
ADIPOR2	1.29418109	3.55870213	1.122828685	1.491683198	0.00037269
IL16	0.77449895	-3.5342428	0.672164822	0.892413004	0.00040895
HLA-DMA	0.79675433	-3.5318539	0.702369266	0.903822957	0.00041266
IL1R2	1.26435702	3.50130822	1.108779127	1.441764764	0.00046298
CD1D	0.78661932	-3.4493368	0.68633559	0.90155597	0.00056197
FURIN	1.28350462	3.4443921	1.113566081	1.479377049	0.00057235
CD1C	0.7784147	-3.4257938	0.674482554	0.898361926	0.00061301
CAT	0.78488648	-3.4034983	0.682699414	0.90236899	0.00066529
BIRC5	1.3069802	3.38789844	1.119451196	1.525923808	0.0007043
ADIPOQ	1.17315859	3.37031612	1.069112024	1.287331029	0.00075082
IL22RA1	1.27268178	3.36449284	1.105900246	1.464615739	0.00076684
XCR1	0.75283886	-3.3581324	0.637880814	0.888514507	0.00078471
PTGDS	0.78417491	-3.3360399	0.679797031	0.904579254	0.00084981
PSMD11	1.29213458	3.32542065	1.110974949	1.502834774	0.00088285
SEMA4A	0.78289663	-3.3099886	0.67727176	0.904994375	0.000933
ANGPTL4	1.2949554	3.30001988	1.110667039	1.509821964	0.00096678
MAP3K8	0.7889905	-3.2661729	0.684394694	0.909571637	0.00109012
CD19	0.77959956	-3.264621	0.671359396	0.905290773	0.00109611
CCR6	0.75686043	-3.2643002	0.640285973	0.894659152	0.00109735
CD79A	0.79311258	-3.2552142	0.689801863	0.911896014	0.00113307
FGF12	1.24200716	3.24120855	1.089449507	1.415927751	0.00119024
HLA-DOB	0.79819204	-3.2151554	0.695715856	0.915762566	0.00130374
ADRB2	0.79022101	-3.1705419	0.683185815	0.914025493	0.00152155
CD79B	0.79453184	-3.1658921	0.689082902	0.916117419	0.00154608
NR3C2	0.7912905	-3.1645916	0.684496384	0.914746488	0.00155301
CIITA	0.8006482	-3.1634334	0.697617324	0.918895663	0.0015592
PGC	0.79804825	-3.1582114	0.693791736	0.917971453	0.0015874

IL11RA	0.7837426	-3.1567525	0.673702068	0.911756824	0.00159537
GRAP2	0.78185144	-3.1423044	0.670597807	0.911562286	0.00167624
CX3CR1	0.78794782	-3.1311331	0.678748692	0.914715222	0.00174133
CD1E	0.78648056	-3.1307534	0.676682472	0.914094409	0.00174359
JAG1	1.26508091	3.10029948	1.090336638	1.467830818	0.00193325
NR0B2	0.77330533	-3.0931228	0.657059012	0.910117853	0.00198062
BTK	0.81530055	-3.0874588	0.716177928	0.92814223	0.00201876
NOD1	0.79554667	-3.0809252	0.687817219	0.920149254	0.00206358
CTF1	0.80629536	-3.0796506	0.703047688	0.9247057	0.00207244
GDF10	0.78212591	-3.0680923	0.668497048	0.91506902	0.0021543
CCR4	0.79656746	-3.0674297	0.688823185	0.921164861	0.00215908
STC1	1.26822246	3.05572043	1.088942741	1.47701817	0.0022452
GDF15	0.79378152	-3.0542594	0.684443507	0.920585998	0.00225617
RAC1	1.24272121	3.04790901	1.080653606	1.429094383	0.0023044
CD1B	0.78620792	-3.0376916	0.673188351	0.918201999	0.00238398
CR2	0.79255866	-3.0359388	0.682098927	0.920906349	0.00239788
PTGDR2	0.76039875	-3.0330935	0.637046546	0.907635807	0.00242061
HGF	0.79567893	-3.0269025	0.686221237	0.922595979	0.00247074
NEDD4	1.25234524	3.01903252	1.082134746	1.449328377	0.00253583
PIK3CG	0.805589	-3.012181	0.699879932	0.927264255	0.00259378
KL	0.77172302	-3.0099598	0.651899893	0.913570359	0.00261282
CTSG	0.78974553	-2.9904593	0.676549676	0.921880571	0.00278558
PTPN6	0.79746439	-2.9865131	0.687396323	0.925156897	0.00282179
TNFRSF13B	0.77531069	-2.9752786	0.65564381	0.916818945	0.00292723
CD22	0.79814697	-2.964706	0.687622315	0.926436764	0.00302972
PRKCB	0.80455349	-2.9547432	0.696477127	0.929400688	0.00312929
PSMD1	1.2124098	2.95018506	1.066784489	1.377914238	0.00317584
PPIA	1.23119995	2.9427827	1.071932846	1.414130865	0.00325277
TLR2	0.81928315	-2.9413517	0.717382792	0.935657909	0.00326783
LECT2	1.1950117	2.94034138	1.061201013	1.345695068	0.00327851
NTS	1.2364515	2.92549712	1.072561469	1.425384342	0.00343906
MIF	1.26900789	2.91339261	1.081084005	1.489598429	0.00357525
CACYBP	1.23163773	2.89280929	1.069493335	1.418364617	0.00381813
NCR3	0.80240055	-2.8894238	0.691094657	0.931633075	0.00385949
HLA-DMB	0.82963663	-2.8891136	0.730906436	0.941703221	0.0038633
KITLG	0.81379026	-2.8869552	0.707553212	0.9359785	0.0038899
PLAUR	1.24367655	2.85313471	1.070651336	1.444663924	0.00432903
SHC3	0.80532718	-2.8525906	0.694013065	0.934495187	0.00433644
LGR4	1.26298213	2.84683913	1.075445108	1.483222014	0.00441557
SORT1	0.83314398	-2.8221192	0.733939192	0.945758037	0.00477074
HLA-DQA1	0.83032961	-2.8080152	0.729269873	0.945393864	0.00498479
RXRB	0.81805363	-2.8044423	0.710930008	0.941318736	0.00504037
PSMC5	1.25300327	2.8027817	1.070175418	1.467065278	0.00506639
PPP3CC	0.81719843	-2.7951057	0.709333484	0.941465887	0.00518827
CCR7	0.81644329	-2.7784871	0.707616628	0.942006752	0.00546127
TLR7	0.81702297	-2.7755242	0.708365859	0.942347125	0.00551128
ITK	0.80790328	-2.7621423	0.694421772	0.939929782	0.00574234

PSME3	1.21605221	2.7606434	1.058373606	1.397222088	0.00576876
IL2	0.78705279	-2.7594603	0.663954687	0.932973438	0.00578969
SERPIND1	0.80391173	-2.7588955	0.68844121	0.938749838	0.00579971
F2RL1	1.26115307	2.75340474	1.069148346	1.487639268	0.00589789
ITGAL	0.82121089	-2.7516359	0.713709706	0.944904249	0.00592984
AGER	0.82533642	-2.7343133	0.719236745	0.947087606	0.00625105
ZAP70	0.809751	-2.7318654	0.695983107	0.942115791	0.00629769
ADRB1	0.8049123	-2.7315152	0.688842038	0.940540457	0.00630438
PSMC4	1.21254274	2.73094228	1.055914368	1.392404488	0.00631535
PLSCR1	1.21384834	2.72967404	1.056166967	1.395070901	0.0063397
CXCR6	0.8104641	-2.7278433	0.696881147	0.942559655	0.00637499
CCL20	1.22940808	2.72014459	1.059416564	1.426676048	0.00652534
IL11	1.21086767	2.71777107	1.054800153	1.390026832	0.00657233
IL24	0.81596264	-2.7170172	0.704615533	0.944905421	0.00658732
HLA-DPB1	0.84114997	-2.7165282	0.742454631	0.952964987	0.00659706
HLA-DRA	0.83947507	-2.7045033	0.73949623	0.952970903	0.00684066
EREG	1.20531375	2.70372752	1.052712268	1.380036399	0.00685665
TNFSF8	0.82075946	-2.7035497	0.711255931	0.94712194	0.00686032
RFX5	0.81196924	-2.7021504	0.698112604	0.944395	0.00688926
IGHM	0.82900086	-2.6945675	0.72329227	0.950158682	0.007048
HNF4A	1.21816006	2.68229798	1.054580897	1.407112463	0.00731183
TNFRSF17	0.82134641	-2.6679896	0.710780568	0.949111383	0.00763066
IL1A	1.20371505	2.66775864	1.050422681	1.379377978	0.00763591
IL5RA	0.78848288	-2.6526788	0.661509348	0.939828379	0.00798558
GMFG	0.83911833	-2.6426169	0.736757825	0.955700166	0.00822681
FLT3	0.81157069	-2.6377634	0.694947818	0.947764657	0.00834548
THRA	0.82558758	-2.6344858	0.715876141	0.952112822	0.00842648
IKBKB	0.82014966	-2.6331532	0.707620198	0.950574155	0.00845962
OXTR	1.20504462	2.63250162	1.0488038	1.384560717	0.00847586
HSPA2	1.20412422	2.6231482	1.04808212	1.3833984	0.00871214
CHIT1	0.82144305	-2.6145712	0.70882996	0.95194719	0.00893395
PDGFB	1.20298234	2.61333666	1.047287682	1.381823283	0.00896629
BMP15	0.76828807	-2.610966	0.630363261	0.936391124	0.00902869
PAK4	1.21939097	2.60708188	1.050466031	1.415480638	0.00913175
CXCR4	0.83794391	-2.5976903	0.733297886	0.957523553	0.00938531
SEMA3C	1.21781124	2.58420195	1.048751604	1.414123434	0.00976046
CD74	0.84758159	-2.5826158	0.747614983	0.960915129	0.00980544
IAPP	1.15170568	2.58105498	1.034572394	1.282100674	0.00984989
PAK2	1.22485247	2.57968105	1.049930077	1.428917608	0.00988916
NMB	1.20510564	2.57773389	1.045726641	1.388775568	0.00994506
PTPRC	0.8381278	-2.5708341	0.732559939	0.95890884	0.01014539
TRIM22	0.83563259	-2.5700045	0.728688491	0.958272077	0.01016972
PSMC6	1.20162473	2.56554786	1.04430898	1.382638671	0.0103013
PLCG2	0.83179764	-2.559147	0.722373718	0.957796905	0.01049294
S100A10	1.25113286	2.55781831	1.053763906	1.485468837	0.01053311
STC2	1.21396507	2.55668854	1.046293457	1.40850656	0.01056738
RORA	0.82295806	-2.5515962	0.708559894	0.955826001	0.01072307

CD28	0.83117263	-2.5467576	0.720918642	0.958288352	0.01087289
HLA-DOA	0.84430671	-2.5365585	0.740811955	0.962260139	0.0111948
NRAS	1.19968544	2.53550463	1.042191933	1.380978977	0.01122854
KRAS	1.20556209	2.53028353	1.043037513	1.393411015	0.01139704
GAL	1.1885362	2.51740782	1.03898774	1.359610182	0.01182219
RORC	0.83063673	-2.5151996	0.718807218	0.959864297	0.0118965
CX3CL1	0.83809574	-2.5137475	0.730273988	0.961836904	0.01194559
RELA	1.21854904	2.51162679	1.044371159	1.421775913	0.01201761
TNFRSF10C	0.83636332	-2.4976328	0.726935861	0.962263166	0.01250256
CYLD	0.83921143	-2.4953028	0.731265873	0.963091329	0.01258497
HSP90AA1	1.21810985	2.49466201	1.043195646	1.422352187	0.01260772
IL33	0.83616043	-2.4846676	0.726088965	0.962918181	0.01296725
OGN	0.82673909	-2.4846676	0.711519467	0.960616756	0.01296725
PSMC1	1.19102128	2.47986869	1.037329037	1.3674848	0.01314308
INHA	1.19027278	2.47784145	1.037075517	1.366100421	0.01321799
FGFR2	0.83316433	-2.4598773	0.720395511	0.963585681	0.01389845
TAP2	1.20073637	2.45567837	1.037618405	1.38949716	0.01406189
CCL13	0.84312197	-2.4545955	0.735723824	0.966197693	0.01410432
CD48	0.83953827	-2.4483385	0.729848592	0.965713316	0.01435167
PIK3CD	0.84830831	-2.444899	0.743495764	0.967896556	0.01448927
ANGPTL7	0.78687553	-2.4440216	0.649275038	0.953637619	0.01452455
GMFB	1.19660124	2.43776743	1.035805291	1.382358778	0.01477828
DMBT1	0.84205467	-2.4288671	0.73298309	0.967356657	0.01514608
LCK	0.83875746	-2.4138953	0.727164918	0.967475281	0.015783
LTBR	1.2047186	2.41228569	1.035539479	1.401537002	0.01585285
AVPR2	0.80190167	-2.409549	0.670087887	0.959644689	0.01597225
TNFRSF1A	1.21177399	2.40836459	1.03641056	1.416809382	0.01602417
PIK3R1	0.84530708	-2.4057192	0.73714425	0.969340889	0.01614066
CD247	0.83676616	-2.4033117	0.723578608	0.967659347	0.01624733
GIP	1.15875811	2.38371185	1.026539995	1.308005886	0.01713902
SEMG2	1.12794249	2.37263309	1.021160965	1.245890027	0.0176618
SEMA3A	1.18818875	2.37227333	1.030422536	1.370110267	0.01767901
MR1	0.84941372	-2.3623822	0.741844132	0.97258121	0.01815791
CTLA4	0.84391118	-2.3497328	0.73251955	0.972241725	0.01878689
LTB	0.84769497	-2.3357469	0.737945468	0.973766755	0.01950444
TNFRSF14	0.85358908	-2.3279715	0.747077977	0.975285497	0.01991362
SH2D1A	0.84090409	-2.3201258	0.72639829	0.973460005	0.02033408
CD3D	0.84726282	-2.3198658	0.736552143	0.974614351	0.02034814
NR1H4	1.16771121	2.31759313	1.02421368	1.331313461	0.02047144
BLNK	0.84346712	-2.3011767	0.729623377	0.975073988	0.02138164
PSMD14	1.18416161	2.29802201	1.025178198	1.367799986	0.02156054
IL7R	0.85275187	-2.2924748	0.74418198	0.977161191	0.02187826
CCL17	0.84636854	-2.2916227	0.733842877	0.976148602	0.02192743
CSF2RB	0.85953728	-2.2758795	0.754491179	0.979208705	0.02285322
HLA-DPA1	0.862131	-2.2733755	0.758627377	0.979756191	0.02300356
FGF5	1.11615405	2.27081675	1.015156434	1.227199888	0.02315807
TK2	0.85185636	-2.2670328	0.741591246	0.978516493	0.02338822

SLC22A17	0.84092233	-2.2667593	0.723928562	0.976823412	0.02340493
BMP1	1.19040943	2.26386607	1.023673598	1.384303169	0.02358235
HLA-DQB1	0.86754606	-2.2560617	0.766803172	0.981524582	0.02406677
PTGDR	0.82058857	-2.2534351	0.690930881	0.974577368	0.02423173
IL10RA	0.85987498	-2.2404249	0.75349113	0.981278941	0.02506335
S100A11	1.19045319	2.22503576	1.020985843	1.388049404	0.02607884
LCN1	1.15112039	2.22020112	1.016632913	1.303398839	0.02640512
EPOR	0.84482057	-2.2171245	0.727819051	0.980630817	0.02661459
SEMA6D	0.83534929	-2.2128815	0.712305279	0.979647982	0.02690582
AP3B1	1.17041272	2.20614549	1.017714313	1.346022084	0.02737381
RELB	1.17301885	2.20594528	1.017953839	1.35170494	0.02738783
ICOS	0.8515687	-2.2043644	0.73820334	0.982343503	0.02749873
PRTN3	1.16230075	2.20400271	1.016792674	1.328631761	0.02752415
TEK	0.85519398	-2.1903696	0.743490486	0.983680038	0.02849744
TNFRSF11A	1.18897924	2.17820419	1.017494135	1.389365867	0.02939084
KCNH2	0.84308838	-2.177115	0.723002519	0.983119689	0.02947199
A2M	0.86297289	-2.1738798	0.755600717	0.985602829	0.02971416
IL6ST	0.85813604	-2.1738294	0.747565341	0.985061007	0.02971795
CXCR5	0.82896811	-2.1726154	0.699920977	0.981808164	0.02980928
FGF14	0.84003607	-2.161358	0.71721563	0.983889049	0.03066769
PLTP	0.85459622	-2.160466	0.741062526	0.985523712	0.03073661
MPL	0.8424839	-2.1500305	0.720615683	0.984962089	0.0315528
PTK2B	0.86011946	-2.1481723	0.749637083	0.986884848	0.03170007
ACVR2A	0.85414495	-2.1469827	0.739651745	0.986360958	0.03179466
GHRHR	1.14963067	2.14146939	1.011888752	1.306122512	0.0322362
PLCG1	0.86500852	-2.1387237	0.757364183	0.987952381	0.03245806
PIK3R3	0.8557934	-2.1349493	0.741790143	0.987317427	0.03276515
ANGPT1	0.85339839	-2.1329659	0.737713642	0.987224259	0.03292752
GH1	0.83167859	-2.1312487	0.702011254	0.985296555	0.03306866
MASP2	0.84564803	-2.1281114	0.72465653	0.986840739	0.03332785
S100P	1.18892122	2.1239368	1.013449216	1.39477504	0.03367543
CAMP	0.84973428	-2.123162	0.731142402	0.987561859	0.03374028
CYSLTR1	0.8522199	-2.1224881	0.735226527	0.987829909	0.03379677
ELN	0.8646506	-2.1049204	0.755145774	0.990034889	0.03529824
GHR	0.83381562	-2.1014338	0.70380714	0.987839493	0.0356029
KNG1	1.14394738	2.09526083	1.008721881	1.297300698	0.03614781
FGF18	0.84570084	-2.093688	0.72290661	0.989353123	0.03628778
CD1A	0.85448961	-2.0926597	0.737469474	0.990078253	0.03637954
CRHR2	0.83947306	-2.0885586	0.712348494	0.989284081	0.03674747
CD81	0.8713274	-2.0878297	0.76564285	0.991599975	0.0368132
NFATC1	0.86818458	-2.0872547	0.760270015	0.991416796	0.03686512
BMP3	0.85500276	-2.0862196	0.737993067	0.990564485	0.03695874
OPRM1	1.11709834	2.08589588	1.00670778	1.23959377	0.03698806
S1PR2	0.85700016	-2.0844635	0.741249962	0.990825389	0.03711803
TUBB3	1.1662879	2.08193466	1.009052664	1.348024265	0.03734844
PF4V1	0.83172041	-2.0786445	0.699074764	0.98953485	0.03765003
SEMA4G	1.16718855	2.07632055	1.008701272	1.350577367	0.03786431

ABCC4	0.86013322	-2.0725971	0.745911663	0.991845533	0.03820979
PTH1R	0.85521923	-2.0709154	0.737554194	0.991655857	0.03836671
PLXND1	0.87125628	-2.069228	0.764631862	0.992748981	0.03852469
MC1R	1.15952365	2.06426406	1.007506426	1.334477931	0.03899269
CD3G	0.85670753	-2.056223	0.739280946	0.992786032	0.03976103
PLXNA2	0.86311018	-2.0528804	0.749939475	0.993359076	0.04008418
DES	0.86138079	-2.0524475	0.74698259	0.99329875	0.0401262
TSHR	0.83335409	-2.0499781	0.700060389	0.992027342	0.04036657
OAS1	1.17308917	2.04356236	1.006551974	1.367180479	0.0409968
DUOX1	0.85901804	-2.0435316	0.742511978	0.993804839	0.04099984
GREM1	1.16599541	2.04123234	1.006133077	1.351257918	0.04122774
HDGF	1.15961138	2.04063325	1.005871186	1.336849668	0.04128729
DEFB1	1.16802197	2.03977301	1.006095292	1.356010052	0.04137294
IL10	0.86053922	-2.035521	0.744667844	0.994440344	0.04179848
NR2F2	1.16076212	2.03373531	1.00542223	1.340102358	0.04197829
CYSLTR2	0.85064196	-2.0201284	0.727086223	0.995193854	0.04337007
S100B	0.8665669	-2.0198626	0.754134236	0.995761964	0.04339764
IFNAR2	0.86972824	-2.018094	0.759474441	0.995987712	0.04358148
IFNA5	0.80071661	-2.0121725	0.644854971	0.994250063	0.04420175
LILRB3	0.86942567	-2.0110019	0.758590085	0.996455156	0.04432525
RXRG	0.83782757	-2.0086472	0.704971856	0.995720644	0.04457456
FYN	0.87178327	-2.0045023	0.76232671	0.996955857	0.04501628
SEMA7A	1.162006	2.00070786	1.003062399	1.34613554	0.04542388
SCG2	1.139698	1.98868309	1.001890172	1.296461003	0.04673619
CCL19	0.87069245	-1.9885898	0.759617925	0.998008758	0.0467465
IL17RB	0.85791445	-1.9884005	0.737632087	0.997810726	0.04676741
IGHD	0.86488176	-1.9883282	0.749571064	0.997931349	0.04677541
IGHG1	0.86553829	-1.9867202	0.750614883	0.998057126	0.04695341
GNAI1	1.16636007	1.97410259	1.001102759	1.358897286	0.04837008
VIPR2	1.14638983	1.9727691	1.00088717	1.313044754	0.04852188
MET	1.1714526	1.96715515	1.000578649	1.37150757	0.04916533
FABP2	1.12469291	1.96691024	1.000415079	1.264409308	0.04919356
CSF3R	0.86351961	-1.9613828	0.745745271	0.999893861	0.04983439
PTX3	1.14395037	1.96072049	1.000051891	1.308554554	0.04991164
UTS2	0.8351539	-1.9606693	0.697527232	0.999935197	0.04991761

Table S3. Top 1000 risk models screened by Kaplan–Meier analysis.

Number of genes	Log rank p-value	Gene combination
9 genes	0	DKK1 | CCR6 | S100A10 | SEMA3C | BTK | CCL20 | TNFRSF11A | SCG2 | GPI
10 genes	0	DKK1 | F2RL1 | CCR6 | SEMA3C | PSMC1 | CCL20 | ADIPOR2 | TNFRSF11A | SCG2 | GPI
10 genes	0	F2RL1 | RFXAP | FCGRT | CCR6 | PSMC1 | CCL20 | PSMD2 | ADIPOR2 | SCG2 | GPI
11 genes	0	DKK1 | F2RL1 | FCGRT | CCR6 | SEMA3C | PSMC1 | CCL20 | ADIPOR2 | TNFRSF11A | SCG2 | GPI
11 genes	0	DKK1 | CCR6 | S100A10 | SEMA3C | OXTR | BTK | CCL20 | PSMD2 | TNFRSF11A | SCG2 | GPI
11 genes	0	DKK1 | S100A10 | SHC1 | SEMA3C | OXTR | BTK | CCL20 | ADIPOR2 | TNFRSF11A | SCG2 | GPI
11 genes	0	F2RL1 | RFXAP | CCR6 | S100A10 | PSMC1 | CCL20 | FURIN | PSMD2 | ADIPOR2 | SCG2 | GPI
12 genes	0	INSL4 | F2RL1 | RFXAP | CCR6 | S100A10 | PSMC1 | CCL20 | FURIN | PSMD2 | ADIPOR2 | SCG2 | GPI
12 genes	0	F2RL1 | RFXAP | FCGRT | CCR6 | S100A10 | PSMC1 | CCL20 | FURIN | PSMD2 | ADIPOR2 | SCG2 | GPI
13 genes	0	DKK1 | CCR6 | S100A10 | SHC1 | OXTR | BTK | CCL20 | FURIN | PSMD2 | ADIPOR2 | TNFRSF11A | SCG2 | GPI
14 genes	0	DKK1 | INSL4 | CCR6 | S100A10 | SHC1 | OXTR | BTK | CCL20 | FURIN | PSMD2 | ADIPOR2 | TNFRSF11A | SCG2 | GPI
9 genes	1.11E-16	DKK1 | SHC1 | OXTR | BTK | CCL20 | PSMD2 | TNFRSF11A | SCG2 | GPI
9 genes	1.11E-16	INSL4 | F2RL1 | RFXAP | CCR6 | PSMC1 | CCL20 | ADIPOR2 | TNFRSF11A | GPI
10 genes	1.11E-16	DKK1 | INSL4 | CCR6 | S100A10 | SEMA3C | BTK | CCL20 | TNFRSF11A | SCG2 | GPI
10 genes	1.11E-16	DKK1 | F2RL1 | RFXAP | CCR6 | SEMA3C | BTK | PSMC1 | CCL20 | ADIPOR2 | GPI
10 genes	1.11E-16	DKK1 | F2RL1 | FCGRT | CCR6 | SEMA3C | CCL20 | ADIPOR2 | TNFRSF11A | SCG2 | GPI
10 genes	1.11E-16	DKK1 | SHC1 | OXTR | BTK | CCL20 | FURIN | PSMD2 | TNFRSF11A | SCG2 | GPI
10 genes	1.11E-16	CCR6 | S100A10 | SHC1 | SEMA3C | BTK | CCL20 | FURIN | PSMD2 | TNFRSF11A | SCG2
11 genes	1.11E-16	DKK1 | INSL4 | F2RL1 | FCGRT | CCR6 | SHC1 | PSMC1 | FURIN | ADIPOR2 | TNFRSF11A | SCG2
11 genes	1.11E-16	DKK1 | FCGRT | S100A10 | SHC1 | SEMA3C | OXTR | BTK | CCL20 | TNFRSF11A | SCG2 | GPI
11 genes	1.11E-16	DKK1 | CCR6 | S100A10 | SHC1 | OXTR | BTK | CCL20 | FURIN | PSMD2 | TNFRSF11A | SCG2
11 genes	1.11E-16	DKK1 | CCR6 | SEMA3C | OXTR | BTK | PSMC1 | CCL20 | PSMD2 | TNFRSF11A | SCG2 | GPI
11 genes	1.11E-16	DKK1 | S100A10 | SHC1 | SEMA3C | OXTR | BTK | CCL20 | PSMD2 | TNFRSF11A | SCG2 | GPI
11 genes	1.11E-16	DKK1 | S100A10 | SHC1 | OXTR | BTK | PSMC1 | CCL20 | FURIN | PSMD2 | ADIPOR2 | SCG2
11 genes	1.11E-16	F2RL1 | RFXAP | FCGRT | CCR6 | S100A10 | PSMC1 | CCL20 | FURIN | PSMD2 | ADIPOR2 | SCG2
11 genes	1.11E-16	F2RL1 | RFXAP | CCR6 | OXTR | BTK | PSMC1 | CCL20 | FURIN | PSMD2 | SCG2 | GPI
12 genes	1.11E-16	DKK1 | INSL4 | FCGRT | S100A10 | SHC1 | SEMA3C | OXTR | BTK | CCL20 | TNFRSF11A | SCG2 | GPI
12 genes	1.11E-16	DKK1 | INSL4 | S100A10 | SHC1 | SEMA3C | BTK | CCL20 | FURIN | PSMD2 | TNFRSF11A | SCG2 | GPI
12 genes	1.11E-16	DKK1 | INSL4 | SHC1 | OXTR | BTK | PSMC1 | CCL20 | PSMD2 | ADIPOR2 | TNFRSF11A | SCG2 | GPI

12 genes	1.11E-16	DKK1 | FCGRT | CCR6 | S100A10 | SHC1 | OXTR | CCL20 | FURIN | PSMD2 | TNFRSF11A | SCG2 | GPI
12 genes	1.11E-16	DKK1 | CCR6 | S100A10 | SHC1 | SEMA3C | OXTR | BTK | CCL20 | ADIPOR2 | TNFRSF11A | SCG2 | GPI
12 genes	1.11E-16	DKK1 | CCR6 | S100A10 | SHC1 | OXTR | BTK | PSMC1 | CCL20 | ADIPOR2 | TNFRSF11A | SCG2 | GPI
12 genes	1.11E-16	DKK1 | CCR6 | S100A10 | SEMA3C | OXTR | BTK | CCL20 | PSMD2 | ADIPOR2 | TNFRSF11A | SCG2 | GPI
12 genes	1.11E-16	DKK1 | CCR6 | SEMA3C | OXTR | BTK | CCL20 | FURIN | PSMD2 | ADIPOR2 | TNFRSF11A | SCG2 | GPI
13 genes	1.11E-16	DKK1 | INSL4 | CCR6 | S100A10 | SHC1 | SEMA3C | OXTR | BTK | CCL20 | ADIPOR2 | TNFRSF11A | SCG2 | GPI
13 genes	1.11E-16	DKK1 | INSL4 | CCR6 | SEMA3C | OXTR | BTK | CCL20 | FURIN | PSMD2 | ADIPOR2 | TNFRSF11A | SCG2 | GPI
13 genes	1.11E-16	DKK1 | INSL4 | SHC1 | SEMA3C | OXTR | BTK | CCL20 | FURIN | PSMD2 | ADIPOR2 | TNFRSF11A | SCG2 | GPI
13 genes	1.11E-16	DKK1 | CCR6 | S100A10 | SHC1 | SEMA3C | OXTR | BTK | CCL20 | FURIN | PSMD2 | TNFRSF11A | SCG2 | GPI
14 genes	1.11E-16	DKK1 | INSL4 | CCR6 | S100A10 | SHC1 | SEMA3C | OXTR | BTK | CCL20 | FURIN | PSMD2 | TNFRSF11A | SCG2 | GPI
14 genes	1.11E-16	DKK1 | CCR6 | S100A10 | SHC1 | SEMA3C | OXTR | BTK | CCL20 | FURIN | PSMD2 | ADIPOR2 | TNFRSF11A | SCG2 | GPI
15 genes	1.11E-16	DKK1 | INSL4 | CCR6 | S100A10 | SHC1 | SEMA3C | OXTR | BTK | CCL20 | FURIN | PSMD2 | ADIPOR2 | TNFRSF11A | SCG2 | GPI
8 genes	2.22E-16	VEGFC | F2RL1 | CCR6 | SHC1 | OXTR | CCL20 | FURIN | PSMD2
9 genes	2.22E-16	DKK1 | F2RL1 | CCR6 | SEMA3C | CCL20 | ADIPOR2 | TNFRSF11A | SCG2 | GPI
9 genes	2.22E-16	DKK1 | S100A10 | SHC1 | SEMA3C | BTK | CCL20 | TNFRSF11A | SCG2 | GPI
10 genes	2.22E-16	DKK1 | F2RL1 | FCGRT | CCR6 | SEMA3C | PSMC1 | CCL20 | TNFRSF11A | SCG2 | GPI
10 genes	2.22E-16	DKK1 | S100A10 | SHC1 | SEMA3C | OXTR | BTK | CCL20 | TNFRSF11A | SCG2 | GPI
10 genes	2.22E-16	DKK1 | S100A10 | SHC1 | SEMA3C | BTK | PSMC1 | CCL20 | TNFRSF11A | SCG2 | GPI
10 genes	2.22E-16	INSL4 | F2RL1 | RFXAP | FCGRT | CCR6 | S100A10 | PSMC1 | CCL20 | ADIPOR2 | SCG2
10 genes	2.22E-16	INSL4 | F2RL1 | RFXAP | CCR6 | PSMC1 | CCL20 | PSMD2 | ADIPOR2 | TNFRSF11A | GPI
10 genes	2.22E-16	F2RL1 | RFXAP | CCR6 | S100A10 | PSMC1 | CCL20 | FURIN | PSMD2 | SCG2 | GPI
11 genes	2.22E-16	DKK1 | INSL4 | S100A10 | SHC1 | SEMA3C | BTK | PSMC1 | CCL20 | TNFRSF11A | SCG2 | GPI
11 genes	2.22E-16	DKK1 | CCR6 | S100A10 | SHC1 | OXTR | BTK | PSMC1 | CCL20 | TNFRSF11A | SCG2 | GPI
11 genes	2.22E-16	DKK1 | CCR6 | S100A10 | SEMA3C | OXTR | BTK | PSMC1 | CCL20 | PSMD2 | TNFRSF11A | SCG2
11 genes	2.22E-16	DKK1 | CCR6 | S100A10 | SEMA3C | BTK | PSMC1 | CCL20 | ADIPOR2 | TNFRSF11A | SCG2 | GPI
11 genes	2.22E-16	DKK1 | S100A10 | SHC1 | SEMA3C | BTK | CCL20 | FURIN | PSMD2 | TNFRSF11A | SCG2 | GPI
12 genes	2.22E-16	DKK1 | INSL4 | S100A10 | SHC1 | SEMA3C | OXTR | BTK | CCL20 | PSMD2 | TNFRSF11A | SCG2 | GPI
12 genes	2.22E-16	DKK1 | INSL4 | S100A10 | SHC1 | SEMA3C | OXTR | BTK | CCL20 | ADIPOR2 | TNFRSF11A | SCG2 | GPI
12 genes	2.22E-16	DKK1 | INSL4 | S100A10 | SHC1 | OXTR | BTK | PSMC1 | CCL20 | FURIN | PSMD2 | ADIPOR2 | SCG2
12 genes	2.22E-16	DKK1 | CCR6 | S100A10 | SHC1 | OXTR | BTK | PSMC1 | CCL20 | PSMD2 | TNFRSF11A | SCG2 | GPI
12 genes	2.22E-16	DKK1 | CCR6 | S100A10 | SHC1 | OXTR | BTK | CCL20 | FURIN | PSMD2 | TNFRSF11A | SCG2 | GPI
12 genes	2.22E-16	DKK1 | CCR6 | S100A10 | SEMA3C | BTK | CCL20 | FURIN | PSMD2 | ADIPOR2 | TNFRSF11A | SCG2 | GPI

12 genes	2.22E-16	DKK1 | S100A10 | SHC1 | SEMA3C | OXTR | BTK | CCL20 | FURIN | PSMD2 | TNFRSF11A | SCG2 | GPI
12 genes	2.22E-16	DKK1 | S100A10 | SHC1 | SEMA3C | OXTR | BTK | CCL20 | FURIN | ADIPOR2 | TNFRSF11A | SCG2 | GPI
12 genes	2.22E-16	DKK1 | S100A10 | SHC1 | SEMA3C | OXTR | BTK | CCL20 | PSMD2 | ADIPOR2 | TNFRSF11A | SCG2 | GPI
12 genes	2.22E-16	DKK1 | S100A10 | SHC1 | SEMA3C | BTK | CCL20 | FURIN | PSMD2 | ADIPOR2 | TNFRSF11A | SCG2 | GPI
12 genes	2.22E-16	DKK1 | SHC1 | SEMA3C | BTK | PSMC1 | CCL20 | FURIN | PSMD2 | ADIPOR2 | TNFRSF11A | SCG2 | GPI
12 genes	2.22E-16	F2RL1 | RFXAP | FCGRT | CCR6 | S100A10 | OXTR | BTK | CCL20 | FURIN | PSMD2 | ADIPOR2 | SCG2
12 genes	2.22E-16	F2RL1 | RFXAP | CCR6 | S100A10 | BTK | PSMC1 | CCL20 | FURIN | PSMD2 | ADIPOR2 | SCG2 | GPI
13 genes	2.22E-16	DKK1 | INSL4 | S100A10 | SHC1 | SEMA3C | OXTR | BTK | CCL20 | FURIN | PSMD2 | TNFRSF11A | SCG2 | GPI
13 genes	2.22E-16	DKK1 | INSL4 | S100A10 | SHC1 | SEMA3C | OXTR | BTK | CCL20 | FURIN | ADIPOR2 | TNFRSF11A | SCG2 | GPI
13 genes	2.22E-16	DKK1 | INSL4 | S100A10 | SHC1 | SEMA3C | BTK | CCL20 | FURIN | PSMD2 | ADIPOR2 | TNFRSF11A | SCG2 | GPI
13 genes	2.22E-16	DKK1 | INSL4 | SHC1 | SEMA3C | BTK | PSMC1 | CCL20 | FURIN | PSMD2 | ADIPOR2 | TNFRSF11A | SCG2 | GPI
13 genes	2.22E-16	DKK1 | F2RL1 | RFXAP | FCGRT | CCR6 | SHC1 | OXTR | CCL20 | FURIN | PSMD2 | ADIPOR2 | TNFRSF11A | SCG2
13 genes	2.22E-16	DKK1 | S100A10 | SHC1 | SEMA3C | OXTR | BTK | CCL20 | FURIN | PSMD2 | ADIPOR2 | TNFRSF11A | SCG2 | GPI
13 genes	2.22E-16	F2RL1 | RFXAP | FCGRT | CCR6 | S100A10 | BTK | PSMC1 | CCL20 | FURIN | PSMD2 | ADIPOR2 | SCG2 | GPI
13 genes	2.22E-16	F2RL1 | RFXAP | CCR6 | S100A10 | SHC1 | BTK | PSMC1 | CCL20 | FURIN | PSMD2 | ADIPOR2 | SCG2 | GPI
14 genes	2.22E-16	DKK1 | INSL4 | CCR6 | S100A10 | SHC1 | SEMA3C | OXTR | BTK | PSMC1 | CCL20 | ADIPOR2 | TNFRSF11A | SCG2 | GPI
14 genes	2.22E-16	INSL4 | F2RL1 | RFXAP | FCGRT | CCR6 | S100A10 | SHC1 | PSMC1 | CCL20 | FURIN | PSMD2 | ADIPOR2 | SCG2 | GPI
14 genes	2.22E-16	INSL4 | F2RL1 | RFXAP | FCGRT | CCR6 | S100A10 | BTK | PSMC1 | CCL20 | FURIN | PSMD2 | ADIPOR2 | SCG2 | GPI
14 genes	2.22E-16	F2RL1 | RFXAP | FCGRT | CCR6 | S100A10 | SHC1 | BTK | PSMC1 | CCL20 | FURIN | PSMD2 | ADIPOR2 | SCG2 | GPI
9 genes	3.33E-16	DKK1 | F2RL1 | FCGRT | CCR6 | PSMC1 | CCL20 | TNFRSF11A | SCG2 | GPI
9 genes	3.33E-16	INSL4 | F2RL1 | RFXAP | CCR6 | PSMC1 | CCL20 | PSMD2 | TNFRSF11A | GPI
10 genes	3.33E-16	DKK1 | INSL4 | SHC1 | SEMA3C | BTK | CCL20 | PSMD2 | TNFRSF11A | SCG2 | GPI
10 genes	3.33E-16	DKK1 | S100A10 | SHC1 | SEMA3C | BTK | CCL20 | PSMD2 | TNFRSF11A | SCG2 | GPI
10 genes	3.33E-16	VEGFC | INSL4 | F2RL1 | FCGRT | CCR6 | SHC1 | OXTR | CCL20 | FURIN | PSMD2
10 genes	3.33E-16	INSL4 | F2RL1 | CCR6 | S100A10 | PSMC1 | CCL20 | FURIN | ADIPOR2 | TNFRSF11A | GPI
10 genes	3.33E-16	F2RL1 | RFXAP | FCGRT | S100A10 | PSMC1 | CCL20 | FURIN | PSMD2 | ADIPOR2 | SCG2
11 genes	3.33E-16	DKK1 | INSL4 | F2RL1 | RFXAP | CCR6 | SEMA3C | BTK | PSMC1 | CCL20 | ADIPOR2 | GPI
11 genes	3.33E-16	DKK1 | INSL4 | SHC1 | SEMA3C | BTK | CCL20 | PSMD2 | ADIPOR2 | TNFRSF11A | SCG2 | GPI
11 genes	3.33E-16	DKK1 | F2RL1 | RFXAP | FCGRT | CCR6 | SEMA3C | BTK | PSMC1 | CCL20 | ADIPOR2 | GPI
11 genes	3.33E-16	DKK1 | FCGRT | S100A10 | SHC1 | SEMA3C | BTK | CCL20 | ADIPOR2 | TNFRSF11A | SCG2 | GPI
11 genes	3.33E-16	DKK1 | CCR6 | S100A10 | SHC1 | OXTR | BTK | CCL20 | PSMD2 | TNFRSF11A | SCG2 | GPI
11 genes	3.33E-16	DKK1 | CCR6 | S100A10 | SHC1 | OXTR | CCL20 | FURIN | PSMD2 | TNFRSF11A | SCG2 | GPI

11 genes	3.33E-16	DKK1 | S100A10 | SHC1 | SEMA3C | OXTR | BTK | CCL20 | FURIN | TNFRSF11A | SCG2 | GPI
11 genes	3.33E-16	INSL4 | F2RL1 | RFXAP | FCGRT | S100A10 | SHC1 | CCL20 | FURIN | PSMD2 | TNFRSF11A | SCG2
12 genes	3.33E-16	DKK1 | INSL4 | FCGRT | S100A10 | SHC1 | SEMA3C | BTK | CCL20 | ADIPOR2 | TNFRSF11A | SCG2 | GPI
12 genes	3.33E-16	DKK1 | INSL4 | FCGRT | S100A10 | SHC1 | OXTR | BTK | CCL20 | ADIPOR2 | TNFRSF11A | SCG2 | GPI
12 genes	3.33E-16	DKK1 | INSL4 | S100A10 | SHC1 | SEMA3C | OXTR | BTK | CCL20 | FURIN | PSMD2 | ADIPOR2 | SCG2
12 genes	3.33E-16	DKK1 | INSL4 | S100A10 | SHC1 | SEMA3C | OXTR | BTK | CCL20 | FURIN | TNFRSF11A | SCG2 | GPI
12 genes	3.33E-16	DKK1 | FCGRT | S100A10 | SHC1 | SEMA3C | OXTR | BTK | CCL20 | FURIN | PSMD2 | ADIPOR2 | SCG2
12 genes	3.33E-16	DKK1 | CCR6 | S100A10 | SEMA3C | OXTR | BTK | PSMC1 | CCL20 | PSMD2 | ADIPOR2 | TNFRSF11A | SCG2
12 genes	3.33E-16	VEGFC | INSL4 | CCR6 | SHC1 | SEMA3C | OXTR | BTK | PSMC1 | CCL20 | TNFRSF11A | SCG2 | GPI
12 genes	3.33E-16	VEGFC | CCR6 | SHC1 | SEMA3C | OXTR | BTK | CCL20 | FURIN | PSMD2 | TNFRSF11A | SCG2 | GPI
13 genes	3.33E-16	DKK1 | INSL4 | FCGRT | S100A10 | SHC1 | SEMA3C | OXTR | BTK | CCL20 | FURIN | PSMD2 | ADIPOR2 | SCG2
13 genes	3.33E-16	DKK1 | CCR6 | S100A10 | SHC1 | SEMA3C | OXTR | CCL20 | FURIN | PSMD2 | ADIPOR2 | TNFRSF11A | SCG2 | GPI
13 genes	3.33E-16	VEGFC | INSL4 | CCR6 | SHC1 | SEMA3C | OXTR | BTK | PSMC1 | CCL20 | FURIN | TNFRSF11A | SCG2 | GPI
13 genes	3.33E-16	INSL4 | F2RL1 | RFXAP | FCGRT | CCR6 | S100A10 | PSMC1 | CCL20 | FURIN | PSMD2 | ADIPOR2 | SCG2 | GPI
13 genes	3.33E-16	INSL4 | F2RL1 | RFXAP | CCR6 | S100A10 | BTK | PSMC1 | CCL20 | FURIN | PSMD2 | ADIPOR2 | SCG2 | GPI
14 genes	3.33E-16	DKK1 | INSL4 | S100A10 | SHC1 | SEMA3C | OXTR | BTK | CCL20 | FURIN | PSMD2 | ADIPOR2 | TNFRSF11A | SCG2 | GPI
15 genes	3.33E-16	INSL4 | F2RL1 | RFXAP | FCGRT | CCR6 | S100A10 | SHC1 | BTK | PSMC1 | CCL20 | FURIN | PSMD2 | ADIPOR2 | SCG2 | GPI
8 genes	4.44E-16	INSL4 | F2RL1 | RFXAP | CCR6 | PSMC1 | CCL20 | TNFRSF11A | GPI
9 genes	4.44E-16	DKK1 | F2RL1 | FCGRT | CCR6 | PSMC1 | CCL20 | ADIPOR2 | TNFRSF11A | GPI
9 genes	4.44E-16	DKK1 | F2RL1 | CCR6 | OXTR | PSMC1 | CCL20 | TNFRSF11A | SCG2 | GPI
9 genes	4.44E-16	DKK1 | FCGRT | SHC1 | SEMA3C | BTK | CCL20 | TNFRSF11A | SCG2 | GPI
9 genes	4.44E-16	INSL4 | F2RL1 | FCGRT | CCR6 | S100A10 | PSMC1 | CCL20 | FURIN | PSMD2
10 genes	4.44E-16	DKK1 | SHC1 | SEMA3C | OXTR | BTK | CCL20 | FURIN | TNFRSF11A | SCG2 | GPI
10 genes	4.44E-16	DKK1 | SHC1 | SEMA3C | OXTR | BTK | CCL20 | ADIPOR2 | TNFRSF11A | SCG2 | GPI
10 genes	4.44E-16	INSL4 | F2RL1 | RFXAP | FCGRT | CCR6 | SHC1 | PSMC1 | FURIN | TNFRSF11A | SCG2
10 genes	4.44E-16	FCGRT | CCR6 | S100A10 | SHC1 | SEMA3C | CCL20 | ADIPOR2 | TNFRSF11A | SCG2 | GPI
11 genes	4.44E-16	DKK1 | INSL4 | SHC1 | SEMA3C | OXTR | BTK | CCL20 | PSMD2 | TNFRSF11A | SCG2 | GPI
11 genes	4.44E-16	DKK1 | INSL4 | SHC1 | SEMA3C | OXTR | BTK | CCL20 | ADIPOR2 | TNFRSF11A | SCG2 | GPI
11 genes	4.44E-16	DKK1 | INSL4 | SHC1 | SEMA3C | BTK | PSMC1 | CCL20 | PSMD2 | TNFRSF11A | SCG2 | GPI
11 genes	4.44E-16	DKK1 | FCGRT | S100A10 | SHC1 | OXTR | BTK | CCL20 | FURIN | PSMD2 | TNFRSF11A | SCG2
11 genes	4.44E-16	DKK1 | FCGRT | SHC1 | SEMA3C | OXTR | BTK | CCL20 | ADIPOR2 | TNFRSF11A | SCG2 | GPI
11 genes	4.44E-16	DKK1 | SHC1 | OXTR | BTK | PSMC1 | CCL20 | PSMD2 | ADIPOR2 | TNFRSF11A | SCG2 | GPI

11 genes	4.44E-16	INSL4 | F2RL1 | RFXAP | CCR6 | SEMA3C | OXTR | PSMC1 | CCL20 | PSMD2 | TNFRSF11A | GPI
11 genes	4.44E-16	INSL4 | F2RL1 | CCR6 | SHC1 | OXTR | PSMC1 | CCL20 | FURIN | PSMD2 | ADIPOR2 | SCG2
12 genes	4.44E-16	DKK1 | INSL4 | FCGRT | SHC1 | SEMA3C | OXTR | BTK | CCL20 | FURIN | TNFRSF11A | SCG2 | GPI
12 genes	4.44E-16	DKK1 | INSL4 | CCR6 | S100A10 | SHC1 | OXTR | CCL20 | FURIN | PSMD2 | TNFRSF11A | SCG2 | GPI
12 genes	4.44E-16	DKK1 | INSL4 | SHC1 | SEMA3C | OXTR | BTK | CCL20 | PSMD2 | ADIPOR2 | TNFRSF11A | SCG2 | GPI
12 genes	4.44E-16	DKK1 | INSL4 | SHC1 | SEMA3C | BTK | PSMC1 | CCL20 | PSMD2 | ADIPOR2 | TNFRSF11A | SCG2 | GPI
12 genes	4.44E-16	DKK1 | FCGRT | S100A10 | SHC1 | SEMA3C | OXTR | BTK | CCL20 | FURIN | TNFRSF11A | SCG2 | GPI
12 genes	4.44E-16	DKK1 | CCR6 | S100A10 | SHC1 | SEMA3C | OXTR | BTK | CCL20 | PSMD2 | TNFRSF11A | SCG2 | GPI
12 genes	4.44E-16	DKK1 | SHC1 | SEMA3C | OXTR | BTK | PSMC1 | CCL20 | FURIN | PSMD2 | TNFRSF11A | SCG2 | GPI
12 genes	4.44E-16	DKK1 | SHC1 | SEMA3C | OXTR | BTK | CCL20 | FURIN | PSMD2 | ADIPOR2 | TNFRSF11A | SCG2 | GPI
12 genes	4.44E-16	INSL4 | F2RL1 | RFXAP | CCR6 | S100A10 | SHC1 | SEMA3C | OXTR | CCL20 | FURIN | PSMD2 | TNFRSF11A
12 genes	4.44E-16	F2RL1 | RFXAP | FCGRT | CCR6 | S100A10 | BTK | PSMC1 | CCL20 | FURIN | PSMD2 | ADIPOR2 | SCG2
13 genes	4.44E-16	DKK1 | INSL4 | FCGRT | S100A10 | SHC1 | SEMA3C | OXTR | BTK | CCL20 | FURIN | TNFRSF11A | SCG2 | GPI
13 genes	4.44E-16	DKK1 | F2RL1 | RFXAP | CCR6 | S100A10 | SHC1 | OXTR | BTK | CCL20 | PSMD2 | ADIPOR2 | SCG2 | GPI
13 genes	4.44E-16	DKK1 | F2RL1 | RFXAP | CCR6 | SHC1 | SEMA3C | OXTR | CCL20 | FURIN | PSMD2 | ADIPOR2 | TNFRSF11A | SCG2
13 genes	4.44E-16	DKK1 | CCR6 | S100A10 | SHC1 | SEMA3C | OXTR | BTK | PSMC1 | CCL20 | ADIPOR2 | TNFRSF11A | SCG2 | GPI
14 genes	4.44E-16	DKK1 | INSL4 | F2RL1 | RFXAP | CCR6 | S100A10 | SHC1 | OXTR | BTK | CCL20 | PSMD2 | ADIPOR2 | SCG2 | GPI
14 genes	4.44E-16	DKK1 | INSL4 | FCGRT | CCR6 | S100A10 | SHC1 | SEMA3C | OXTR | BTK | CCL20 | PSMD2 | ADIPOR2 | TNFRSF11A | SCG2
14 genes	4.44E-16	DKK1 | INSL4 | FCGRT | CCR6 | S100A10 | SHC1 | OXTR | BTK | CCL20 | FURIN | PSMD2 | TNFRSF11A | SCG2 | GPI
10 genes	5.55E-16	DKK1 | INSL4 | FCGRT | SHC1 | SEMA3C | BTK | CCL20 | TNFRSF11A | SCG2 | GPI
10 genes	5.55E-16	DKK1 | INSL4 | S100A10 | SHC1 | OXTR | BTK | CCL20 | PSMD2 | TNFRSF11A | SCG2
10 genes	5.55E-16	DKK1 | INSL4 | SHC1 | SEMA3C | OXTR | BTK | CCL20 | TNFRSF11A | SCG2 | GPI
10 genes	5.55E-16	DKK1 | F2RL1 | CCR6 | S100A10 | SEMA3C | OXTR | PSMC1 | CCL20 | SCG2 | GPI
10 genes	5.55E-16	DKK1 | FCGRT | S100A10 | SHC1 | OXTR | BTK | CCL20 | PSMD2 | TNFRSF11A | SCG2
10 genes	5.55E-16	DKK1 | CCR6 | SHC1 | SEMA3C | OXTR | BTK | CCL20 | TNFRSF11A | SCG2 | GPI
10 genes	5.55E-16	DKK1 | SHC1 | SEMA3C | BTK | CCL20 | PSMD2 | ADIPOR2 | TNFRSF11A | SCG2 | GPI
10 genes	5.55E-16	VEGFC | FCGRT | SEMA3C | BTK | CCL20 | FURIN | PSMD2 | TNFRSF11A | SCG2 | GPI
10 genes	5.55E-16	F2RL1 | RFXAP | FCGRT | CCR6 | S100A10 | CCL20 | PSMD2 | ADIPOR2 | SCG2 | GPI
10 genes	5.55E-16	F2RL1 | CCR6 | BTK | CCL20 | FURIN | PSMD2 | ADIPOR2 | TNFRSF11A | SCG2 | GPI
11 genes	5.55E-16	DKK1 | INSL4 | F2RL1 | RFXAP | FCGRT | CCR6 | PSMC1 | CCL20 | FURIN | ADIPOR2 | SCG2
11 genes	5.55E-16	DKK1 | INSL4 | CCR6 | SHC1 | BTK | PSMC1 | FURIN | PSMD2 | ADIPOR2 | TNFRSF11A | SCG2
11 genes	5.55E-16	DKK1 | INSL4 | SHC1 | SEMA3C | OXTR | BTK | CCL20 | FURIN | TNFRSF11A | SCG2 | GPI

11 genes	5.55E-16	DKK1 | F2RL1 | FCGRT | CCR6 | S100A10 | SEMA3C | OXTR | PSMC1 | CCL20 | SCG2 | GPI
11 genes	5.55E-16	DKK1 | FCGRT | S100A10 | SHC1 | OXTR | BTK | CCL20 | FURIN | PSMD2 | ADIPOR2 | SCG2
11 genes	5.55E-16	DKK1 | SHC1 | SEMA3C | BTK | CCL20 | FURIN | PSMD2 | ADIPOR2 | TNFRSF11A | SCG2 | GPI
12 genes	5.55E-16	DKK1 | INSL4 | F2RL1 | RFXAP | FCGRT | CCR6 | SEMA3C | BTK | PSMC1 | CCL20 | ADIPOR2 | GPI
12 genes	5.55E-16	DKK1 | INSL4 | F2RL1 | RFXAP | CCR6 | SHC1 | PSMC1 | FURIN | PSMD2 | ADIPOR2 | TNFRSF11A | SCG2
12 genes	5.55E-16	DKK1 | INSL4 | FCGRT | S100A10 | SHC1 | OXTR | BTK | CCL20 | FURIN | PSMD2 | ADIPOR2 | SCG2
12 genes	5.55E-16	DKK1 | INSL4 | CCR6 | S100A10 | SHC1 | OXTR | BTK | PSMC1 | CCL20 | TNFRSF11A | SCG2 | GPI
12 genes	5.55E-16	DKK1 | INSL4 | SHC1 | SEMA3C | OXTR | BTK | CCL20 | FURIN | PSMD2 | TNFRSF11A | SCG2 | GPI
12 genes	5.55E-16	DKK1 | INSL4 | SHC1 | SEMA3C | BTK | CCL20 | FURIN | PSMD2 | ADIPOR2 | TNFRSF11A | SCG2 | GPI
12 genes	5.55E-16	DKK1 | FCGRT | S100A10 | SHC1 | SEMA3C | OXTR | BTK | CCL20 | PSMD2 | TNFRSF11A | SCG2 | GPI
12 genes	5.55E-16	DKK1 | CCR6 | S100A10 | SEMA3C | OXTR | BTK | CCL20 | FURIN | PSMD2 | TNFRSF11A | SCG2 | GPI
12 genes	5.55E-16	INSL4 | F2RL1 | RFXAP | FCGRT | CCR6 | BTK | CCL20 | FURIN | PSMD2 | TNFRSF11A | SCG2 | GPI
12 genes	5.55E-16	INSL4 | F2RL1 | RFXAP | CCR6 | S100A10 | BTK | PSMC1 | CCL20 | FURIN | PSMD2 | ADIPOR2 | SCG2
12 genes	5.55E-16	INSL4 | F2RL1 | RFXAP | CCR6 | SHC1 | OXTR | BTK | PSMC1 | FURIN | PSMD2 | TNFRSF11A | SCG2
12 genes	5.55E-16	F2RL1 | RFXAP | FCGRT | S100A10 | SHC1 | PSMC1 | CCL20 | FURIN | PSMD2 | ADIPOR2 | TNFRSF11A | SCG2
12 genes	5.55E-16	F2RL1 | RFXAP | CCR6 | S100A10 | OXTR | BTK | CCL20 | FURIN | PSMD2 | ADIPOR2 | SCG2 | GPI
13 genes	5.55E-16	DKK1 | INSL4 | CCR6 | S100A10 | SEMA3C | OXTR | BTK | CCL20 | FURIN | PSMD2 | TNFRSF11A | SCG2 | GPI
13 genes	5.55E-16	DKK1 | CCR6 | S100A10 | SHC1 | OXTR | BTK | PSMC1 | CCL20 | PSMD2 | ADIPOR2 | TNFRSF11A | SCG2 | GPI
13 genes	5.55E-16	DKK1 | CCR6 | S100A10 | SEMA3C | OXTR | BTK | CCL20 | FURIN | PSMD2 | ADIPOR2 | TNFRSF11A | SCG2 | GPI
13 genes	5.55E-16	DKK1 | SHC1 | SEMA3C | OXTR | BTK | PSMC1 | CCL20 | FURIN | PSMD2 | ADIPOR2 | TNFRSF11A | SCG2 | GPI
13 genes	5.55E-16	INSL4 | F2RL1 | RFXAP | CCR6 | S100A10 | SHC1 | PSMC1 | CCL20 | FURIN | PSMD2 | ADIPOR2 | SCG2 | GPI
14 genes	5.55E-16	DKK1 | INSL4 | CCR6 | S100A10 | SEMA3C | OXTR | BTK | CCL20 | FURIN | PSMD2 | ADIPOR2 | TNFRSF11A | SCG2 | GPI
14 genes	5.55E-16	VEGFC | F2RL1 | RFXAP | FCGRT | CCR6 | S100A10 | SHC1 | SEMA3C | OXTR | PSMC1 | FURIN | PSMD2 | ADIPOR2 | SCG2
15 genes	5.55E-16	DKK1 | INSL4 | F2RL1 | FCGRT | CCR6 | S100A10 | SHC1 | SEMA3C | BTK | CCL20 | FURIN | PSMD2 | ADIPOR2 | SCG2 | GPI
15 genes	5.55E-16	DKK1 | INSL4 | FCGRT | CCR6 | S100A10 | SHC1 | OXTR | BTK | CCL20 | FURIN | PSMD2 | ADIPOR2 | TNFRSF11A | SCG2 | GPI
8 genes	6.66E-16	DKK1 | F2RL1 | CCR6 | SEMA3C | CCL20 | TNFRSF11A | SCG2 | GPI
9 genes	6.66E-16	DKK1 | S100A10 | SHC1 | SEMA3C | BTK | CCL20 | PSMD2 | TNFRSF11A | SCG2
9 genes	6.66E-16	DKK1 | SHC1 | SEMA3C | BTK | CCL20 | PSMD2 | TNFRSF11A | SCG2 | GPI
9 genes	6.66E-16	F2RL1 | RFXAP | FCGRT | CCR6 | PSMC1 | CCL20 | PSMD2 | SCG2 | GPI
10 genes	6.66E-16	DKK1 | F2RL1 | CCR6 | S100A10 | PSMC1 | CCL20 | FURIN | PSMD2 | TNFRSF11A | GPI
10 genes	6.66E-16	DKK1 | F2RL1 | CCR6 | BTK | PSMC1 | CCL20 | PSMD2 | ADIPOR2 | TNFRSF11A | GPI
10 genes	6.66E-16	DKK1 | FCGRT | SHC1 | SEMA3C | BTK | CCL20 | FURIN | TNFRSF11A | SCG2 | GPI

10 genes	6.66E-16	DKK1 | S100A10 | SHC1 | OXTR | BTK | CCL20 | ADIPOR2 | TNFRSF11A | SCG2 | GPI
10 genes	6.66E-16	DKK1 | SHC1 | OXTR | BTK | CCL20 | PSMD2 | ADIPOR2 | TNFRSF11A | SCG2 | GPI
11 genes	6.66E-16	DKK1 | INSL4 | FCGRT | SHC1 | SEMA3C | BTK | CCL20 | FURIN | TNFRSF11A | SCG2 | GPI
11 genes	6.66E-16	DKK1 | F2RL1 | RFXAP | CCR6 | SEMA3C | PSMC1 | CCL20 | ADIPOR2 | TNFRSF11A | SCG2 | GPI
11 genes	6.66E-16	DKK1 | S100A10 | SHC1 | OXTR | BTK | CCL20 | FURIN | PSMD2 | TNFRSF11A | SCG2 | GPI
11 genes	6.66E-16	INSL4 | F2RL1 | RFXAP | FCGRT | CCR6 | BTK | CCL20 | PSMD2 | ADIPOR2 | TNFRSF11A | GPI
11 genes	6.66E-16	F2RL1 | RFXAP | FCGRT | CCR6 | S100A10 | PSMC1 | CCL20 | FURIN | PSMD2 | SCG2 | GPI
12 genes	6.66E-16	DKK1 | INSL4 | CCR6 | S100A10 | SEMA3C | BTK | CCL20 | FURIN | PSMD2 | TNFRSF11A | SCG2 | GPI
12 genes	6.66E-16	DKK1 | INSL4 | SHC1 | SEMA3C | OXTR | BTK | PSMC1 | CCL20 | PSMD2 | TNFRSF11A | SCG2 | GPI
12 genes	6.66E-16	F2RL1 | RFXAP | FCGRT | CCR6 | BTK | PSMC1 | CCL20 | FURIN | PSMD2 | TNFRSF11A | SCG2 | GPI
12 genes	6.66E-16	F2RL1 | RFXAP | CCR6 | S100A10 | SHC1 | PSMC1 | CCL20 | FURIN | PSMD2 | ADIPOR2 | SCG2 | GPI
13 genes	6.66E-16	DKK1 | INSL4 | CCR6 | S100A10 | SHC1 | OXTR | BTK | CCL20 | FURIN | PSMD2 | TNFRSF11A | SCG2 | GPI
13 genes	6.66E-16	DKK1 | INSL4 | CCR6 | S100A10 | SEMA3C | BTK | CCL20 | FURIN | PSMD2 | ADIPOR2 | TNFRSF11A | SCG2 | GPI
13 genes	6.66E-16	DKK1 | INSL4 | S100A10 | SHC1 | SEMA3C | OXTR | BTK | CCL20 | PSMD2 | ADIPOR2 | TNFRSF11A | SCG2 | GPI
13 genes	6.66E-16	DKK1 | INSL4 | SHC1 | SEMA3C | OXTR | BTK | PSMC1 | CCL20 | PSMD2 | ADIPOR2 | TNFRSF11A | SCG2 | GPI
13 genes	6.66E-16	DKK1 | FCGRT | S100A10 | SHC1 | SEMA3C | OXTR | BTK | CCL20 | FURIN | ADIPOR2 | TNFRSF11A | SCG2 | GPI
13 genes	6.66E-16	INSL4 | F2RL1 | RFXAP | CCR6 | S100A10 | SHC1 | BTK | CCL20 | FURIN | PSMD2 | ADIPOR2 | TNFRSF11A | SCG2
14 genes	6.66E-16	DKK1 | INSL4 | FCGRT | CCR6 | S100A10 | SEMA3C | OXTR | BTK | CCL20 | FURIN | PSMD2 | TNFRSF11A | SCG2 | GPI
14 genes	6.66E-16	DKK1 | INSL4 | FCGRT | S100A10 | SHC1 | SEMA3C | OXTR | BTK | CCL20 | FURIN | ADIPOR2 | TNFRSF11A | SCG2 | GPI
14 genes	6.66E-16	DKK1 | INSL4 | CCR6 | S100A10 | SHC1 | OXTR | BTK | PSMC1 | CCL20 | PSMD2 | ADIPOR2 | TNFRSF11A | SCG2 | GPI
14 genes	6.66E-16	DKK1 | F2RL1 | RFXAP | FCGRT | CCR6 | SHC1 | SEMA3C | OXTR | CCL20 | FURIN | PSMD2 | ADIPOR2 | TNFRSF11A | SCG2
14 genes	6.66E-16	DKK1 | FCGRT | CCR6 | S100A10 | SHC1 | OXTR | BTK | CCL20 | FURIN | PSMD2 | ADIPOR2 | TNFRSF11A | SCG2 | GPI
14 genes	6.66E-16	DKK1 | CCR6 | S100A10 | SHC1 | SEMA3C | OXTR | BTK | PSMC1 | CCL20 | FURIN | PSMD2 | ADIPOR2 | TNFRSF11A | SCG2
15 genes	6.66E-16	DKK1 | VEGFC | INSL4 | RFXAP | SHC1 | SEMA3C | OXTR | BTK | CCL20 | FURIN | PSMD2 | ADIPOR2 | TNFRSF11A | SCG2 | GPI
15 genes	6.66E-16	DKK1 | INSL4 | FCGRT | CCR6 | S100A10 | SEMA3C | OXTR | BTK | CCL20 | FURIN | PSMD2 | ADIPOR2 | TNFRSF11A | SCG2 | GPI
9 genes	7.77E-16	DKK1 | F2RL1 | RFXAP | CCR6 | SEMA3C | CCL20 | ADIPOR2 | TNFRSF11A | GPI
9 genes	7.77E-16	DKK1 | F2RL1 | CCR6 | SEMA3C | PSMC1 | CCL20 | TNFRSF11A | SCG2 | GPI
9 genes	7.77E-16	DKK1 | SHC1 | SEMA3C | OXTR | BTK | CCL20 | TNFRSF11A | SCG2 | GPI
9 genes	7.77E-16	INSL4 | F2RL1 | RFXAP | FCGRT | CCR6 | CCL20 | PSMD2 | SCG2 | GPI
9 genes	7.77E-16	F2RL1 | RFXAP | FCGRT | CCR6 | PSMC1 | CCL20 | PSMD2 | ADIPOR2 | SCG2
10 genes	7.77E-16	F2RL1 | RFXAP | FCGRT | CCR6 | BTK | CCL20 | FURIN | PSMD2 | SCG2 | GPI
11 genes	7.77E-16	DKK1 | INSL4 | FCGRT | CCR6 | SEMA3C | BTK | CCL20 | PSMD2 | ADIPOR2 | TNFRSF11A | SCG2

11 genes	7.77E-16	DKK1 | INSL4 | CCR6 | S100A10 | SEMA3C | OXTR | BTK | CCL20 | PSMD2 | TNFRSF11A | SCG2
11 genes	7.77E-16	DKK1 | INSL4 | S100A10 | SHC1 | SEMA3C | OXTR | BTK | CCL20 | TNFRSF11A | SCG2 | GPI
11 genes	7.77E-16	DKK1 | FCGRT | CCR6 | S100A10 | SEMA3C | OXTR | BTK | CCL20 | TNFRSF11A | SCG2 | GPI
11 genes	7.77E-16	DKK1 | S100A10 | SHC1 | SEMA3C | OXTR | BTK | PSMC1 | CCL20 | TNFRSF11A | SCG2 | GPI
11 genes	7.77E-16	DKK1 | SHC1 | SEMA3C | BTK | PSMC1 | CCL20 | FURIN | PSMD2 | TNFRSF11A | SCG2 | GPI
12 genes	7.77E-16	DKK1 | INSL4 | FCGRT | S100A10 | SHC1 | SEMA3C | BTK | CCL20 | PSMD2 | TNFRSF11A | SCG2 | GPI
12 genes	7.77E-16	DKK1 | INSL4 | S100A10 | SHC1 | OXTR | BTK | CCL20 | FURIN | PSMD2 | ADIPOR2 | SCG2 | GPI
12 genes	7.77E-16	DKK1 | FCGRT | CCR6 | S100A10 | SHC1 | OXTR | BTK | PSMC1 | CCL20 | TNFRSF11A | SCG2 | GPI
12 genes	7.77E-16	INSL4 | CCR6 | S100A10 | SHC1 | SEMA3C | BTK | CCL20 | FURIN | ADIPOR2 | TNFRSF11A | SCG2 | GPI
12 genes	7.77E-16	F2RL1 | RFXAP | FCGRT | CCR6 | S100A10 | BTK | PSMC1 | CCL20 | FURIN | PSMD2 | SCG2 | GPI
13 genes	7.77E-16	DKK1 | INSL4 | CCR6 | S100A10 | SHC1 | SEMA3C | BTK | PSMC1 | CCL20 | FURIN | ADIPOR2 | SCG2 | GPI
13 genes	7.77E-16	DKK1 | INSL4 | CCR6 | S100A10 | SHC1 | OXTR | BTK | PSMC1 | CCL20 | ADIPOR2 | TNFRSF11A | SCG2 | GPI
13 genes	7.77E-16	DKK1 | FCGRT | CCR6 | S100A10 | SHC1 | SEMA3C | BTK | CCL20 | FURIN | PSMD2 | TNFRSF11A | SCG2 | GPI
13 genes	7.77E-16	INSL4 | F2RL1 | RFXAP | CCR6 | S100A10 | SHC1 | BTK | PSMC1 | CCL20 | PSMD2 | ADIPOR2 | SCG2 | GPI
14 genes	7.77E-16	DKK1 | INSL4 | FCGRT | CCR6 | S100A10 | SHC1 | SEMA3C | BTK | CCL20 | FURIN | PSMD2 | TNFRSF11A | SCG2 | GPI
14 genes	7.77E-16	DKK1 | INSL4 | CCR6 | S100A10 | SHC1 | SEMA3C | OXTR | BTK | CCL20 | FURIN | PSMD2 | ADIPOR2 | TNFRSF11A | SCG2
14 genes	7.77E-16	DKK1 | INSL4 | CCR6 | S100A10 | SHC1 | SEMA3C | OXTR | BTK | CCL20 | FURIN | PSMD2 | ADIPOR2 | SCG2 | GPI
14 genes	7.77E-16	DKK1 | INSL4 | CCR6 | S100A10 | SHC1 | OXTR | BTK | PSMC1 | CCL20 | FURIN | PSMD2 | TNFRSF11A | SCG2 | GPI
14 genes	7.77E-16	DKK1 | CCR6 | S100A10 | SHC1 | SEMA3C | OXTR | BTK | PSMC1 | CCL20 | FURIN | ADIPOR2 | TNFRSF11A | SCG2 | GPI
14 genes	7.77E-16	INSL4 | F2RL1 | RFXAP | CCR6 | S100A10 | SHC1 | BTK | PSMC1 | CCL20 | FURIN | PSMD2 | ADIPOR2 | SCG2 | GPI
15 genes	7.77E-16	DKK1 | INSL4 | FCGRT | CCR6 | S100A10 | SHC1 | SEMA3C | OXTR | BTK | PSMC1 | CCL20 | PSMD2 | TNFRSF11A | SCG2 | GPI
15 genes	7.77E-16	DKK1 | INSL4 | CCR6 | S100A10 | SHC1 | SEMA3C | OXTR | BTK | PSMC1 | CCL20 | FURIN | ADIPOR2 | TNFRSF11A | SCG2 | GPI
9 genes	8.88E-16	DKK1 | FCGRT | S100A10 | BTK | CCL20 | PSMD2 | ADIPOR2 | SCG2 | GPI
9 genes	8.88E-16	INSL4 | F2RL1 | RFXAP | CCR6 | OXTR | BTK | PSMC1 | CCL20 | SCG2
9 genes	8.88E-16	F2RL1 | CCR6 | SHC1 | BTK | CCL20 | FURIN | PSMD2 | TNFRSF11A | SCG2
10 genes	8.88E-16	DKK1 | INSL4 | FCGRT | S100A10 | BTK | CCL20 | PSMD2 | ADIPOR2 | SCG2 | GPI
10 genes	8.88E-16	DKK1 | F2RL1 | CCR6 | SEMA3C | BTK | CCL20 | ADIPOR2 | TNFRSF11A | SCG2 | GPI
10 genes	8.88E-16	DKK1 | CCR6 | S100A10 | SHC1 | BTK | CCL20 | ADIPOR2 | TNFRSF11A | SCG2 | GPI
10 genes	8.88E-16	DKK1 | S100A10 | SHC1 | BTK | PSMC1 | CCL20 | ADIPOR2 | TNFRSF11A | SCG2 | GPI
10 genes	8.88E-16	INSL4 | F2RL1 | RFXAP | FCGRT | CCR6 | BTK | CCL20 | PSMD2 | TNFRSF11A | SCG2
11 genes	8.88E-16	DKK1 | INSL4 | F2RL1 | CCR6 | SEMA3C | BTK | CCL20 | ADIPOR2 | TNFRSF11A | SCG2 | GPI
11 genes	8.88E-16	DKK1 | INSL4 | F2RL1 | CCR6 | SEMA3C | PSMC1 | CCL20 | ADIPOR2 | TNFRSF11A | SCG2 | GPI

11 genes	8.88E-16	DKK1 | INSL4 | S100A10 | SHC1 | BTK | PSMC1 | CCL20 | ADIPOR2 | TNFRSF11A | SCG2 | GPI
11 genes	8.88E-16	DKK1 | F2RL1 | RFXAP | FCGRT | CCR6 | PSMC1 | CCL20 | ADIPOR2 | TNFRSF11A | SCG2 | GPI
11 genes	8.88E-16	DKK1 | FCGRT | SHC1 | SEMA3C | OXTR | BTK | CCL20 | FURIN | TNFRSF11A | SCG2 | GPI
11 genes	8.88E-16	DKK1 | CCR6 | S100A10 | SEMA3C | OXTR | BTK | CCL20 | ADIPOR2 | TNFRSF11A | SCG2 | GPI
11 genes	8.88E-16	INSL4 | F2RL1 | RFXAP | CCR6 | S100A10 | PSMC1 | CCL20 | FURIN | PSMD2 | ADIPOR2 | SCG2
11 genes	8.88E-16	F2RL1 | RFXAP | FCGRT | CCR6 | BTK | CCL20 | FURIN | PSMD2 | TNFRSF11A | SCG2 | GPI
11 genes	8.88E-16	F2RL1 | RFXAP | FCGRT | S100A10 | SHC1 | CCL20 | FURIN | PSMD2 | ADIPOR2 | TNFRSF11A | SCG2
11 genes	8.88E-16	FCGRT | CCR6 | S100A10 | SHC1 | SEMA3C | BTK | CCL20 | FURIN | PSMD2 | TNFRSF11A | SCG2
12 genes	8.88E-16	DKK1 | INSL4 | F2RL1 | FCGRT | CCR6 | SEMA3C | PSMC1 | CCL20 | ADIPOR2 | TNFRSF11A | SCG2 | GPI
12 genes	8.88E-16	DKK1 | FCGRT | CCR6 | S100A10 | SEMA3C | OXTR | BTK | CCL20 | PSMD2 | TNFRSF11A | SCG2 | GPI
12 genes	8.88E-16	DKK1 | CCR6 | S100A10 | SEMA3C | OXTR | BTK | PSMC1 | CCL20 | PSMD2 | TNFRSF11A | SCG2 | GPI
12 genes	8.88E-16	DKK1 | CCR6 | S100A10 | OXTR | BTK | CCL20 | FURIN | PSMD2 | ADIPOR2 | TNFRSF11A | SCG2 | GPI
12 genes	8.88E-16	VEGFC | F2RL1 | FCGRT | CCR6 | S100A10 | OXTR | CCL20 | FURIN | PSMD2 | ADIPOR2 | SCG2 | GPI
12 genes	8.88E-16	INSL4 | F2RL1 | RFXAP | FCGRT | CCR6 | S100A10 | OXTR | BTK | PSMC1 | CCL20 | FURIN | PSMD2
12 genes	8.88E-16	F2RL1 | RFXAP | FCGRT | CCR6 | BTK | CCL20 | FURIN | PSMD2 | ADIPOR2 | TNFRSF11A | SCG2 | GPI
13 genes	8.88E-16	DKK1 | INSL4 | CCR6 | S100A10 | SHC1 | OXTR | BTK | PSMC1 | CCL20 | FURIN | PSMD2 | TNFRSF11A | SCG2
13 genes	8.88E-16	DKK1 | INSL4 | CCR6 | S100A10 | OXTR | BTK | CCL20 | FURIN | PSMD2 | ADIPOR2 | TNFRSF11A | SCG2 | GPI
13 genes	8.88E-16	DKK1 | F2RL1 | FCGRT | CCR6 | S100A10 | SHC1 | OXTR | CCL20 | FURIN | PSMD2 | TNFRSF11A | SCG2 | GPI
13 genes	8.88E-16	DKK1 | CCR6 | S100A10 | SHC1 | SEMA3C | OXTR | BTK | PSMC1 | CCL20 | PSMD2 | TNFRSF11A | SCG2 | GPI
13 genes	8.88E-16	F2RL1 | RFXAP | FCGRT | CCR6 | SHC1 | BTK | PSMC1 | CCL20 | FURIN | PSMD2 | ADIPOR2 | SCG2 | GPI
14 genes	8.88E-16	DKK1 | INSL4 | FCGRT | CCR6 | SHC1 | SEMA3C | OXTR | BTK | CCL20 | FURIN | ADIPOR2 | TNFRSF11A | SCG2 | GPI
14 genes	8.88E-16	DKK1 | FCGRT | CCR6 | S100A10 | SEMA3C | OXTR | BTK | CCL20 | FURIN | PSMD2 | ADIPOR2 | TNFRSF11A | SCG2 | GPI
14 genes	8.88E-16	INSL4 | F2RL1 | RFXAP | FCGRT | CCR6 | S100A10 | SHC1 | BTK | PSMC1 | CCL20 | PSMD2 | ADIPOR2 | SCG2 | GPI
15 genes	8.88E-16	VEGFC | INSL4 | RFXAP | CCR6 | S100A10 | SHC1 | SEMA3C | OXTR | BTK | CCL20 | FURIN | PSMD2 | ADIPOR2 | TNFRSF11A | SCG2
9 genes	9.99E-16	DKK1 | F2RL1 | RFXAP | CCR6 | PSMC1 | CCL20 | ADIPOR2 | TNFRSF11A | SCG2
9 genes	9.99E-16	DKK1 | F2RL1 | CCR6 | SEMA3C | BTK | CCL20 | TNFRSF11A | SCG2 | GPI
9 genes	9.99E-16	INSL4 | F2RL1 | CCR6 | SEMA3C | PSMC1 | CCL20 | ADIPOR2 | SCG2 | GPI
10 genes	9.99E-16	DKK1 | CCR6 | S100A10 | SEMA3C | OXTR | BTK | CCL20 | ADIPOR2 | TNFRSF11A | SCG2
10 genes	9.99E-16	DKK1 | CCR6 | SEMA3C | OXTR | BTK | CCL20 | ADIPOR2 | TNFRSF11A | SCG2 | GPI
11 genes	9.99E-16	DKK1 | CCR6 | S100A10 | SHC1 | SEMA3C | BTK | CCL20 | ADIPOR2 | TNFRSF11A | SCG2 | GPI
11 genes	9.99E-16	DKK1 | CCR6 | S100A10 | SEMA3C | OXTR | BTK | CCL20 | PSMD2 | ADIPOR2 | TNFRSF11A | SCG2
11 genes	9.99E-16	VEGFC | FCGRT | CCR6 | OXTR | BTK | CCL20 | FURIN | PSMD2 | TNFRSF11A | SCG2 | GPI

11 genes	9.99E-16	INSL4 | F2RL1 | CCR6 | S100A10 | SHC1 | PSMC1 | CCL20 | FURIN | PSMD2 | TNFRSF11A | GPI
12 genes	9.99E-16	DKK1 | INSL4 | F2RL1 | CCR6 | SHC1 | SEMA3C | PSMC1 | FURIN | PSMD2 | ADIPOR2 | TNFRSF11A | SCG2
12 genes	9.99E-16	DKK1 | INSL4 | CCR6 | S100A10 | SHC1 | SEMA3C | BTK | CCL20 | ADIPOR2 | TNFRSF11A | SCG2 | GPI
12 genes	9.99E-16	DKK1 | FCGRT | CCR6 | S100A10 | SHC1 | OXTR | BTK | CCL20 | PSMD2 | TNFRSF11A | SCG2 | GPI
12 genes	9.99E-16	VEGFC | F2RL1 | FCGRT | CCR6 | BTK | CCL20 | FURIN | PSMD2 | ADIPOR2 | TNFRSF11A | SCG2 | GPI
13 genes	9.99E-16	DKK1 | INSL4 | F2RL1 | RFXAP | FCGRT | CCR6 | SEMA3C | PSMC1 | CCL20 | ADIPOR2 | TNFRSF11A | SCG2 | GPI
13 genes	9.99E-16	DKK1 | INSL4 | FCGRT | CCR6 | S100A10 | SHC1 | OXTR | BTK | CCL20 | FURIN | PSMD2 | TNFRSF11A | SCG2
13 genes	9.99E-16	DKK1 | INSL4 | CCR6 | S100A10 | SHC1 | SEMA3C | BTK | CCL20 | FURIN | PSMD2 | TNFRSF11A | SCG2 | GPI
13 genes	9.99E-16	DKK1 | INSL4 | CCR6 | S100A10 | SHC1 | OXTR | BTK | PSMC1 | CCL20 | FURIN | PSMD2 | SCG2 | GPI
13 genes	9.99E-16	DKK1 | INSL4 | CCR6 | S100A10 | SHC1 | OXTR | BTK | PSMC1 | CCL20 | PSMD2 | TNFRSF11A | SCG2 | GPI
13 genes	9.99E-16	DKK1 | FCGRT | CCR6 | SHC1 | SEMA3C | OXTR | BTK | CCL20 | FURIN | ADIPOR2 | TNFRSF11A | SCG2 | GPI
13 genes	9.99E-16	DKK1 | FCGRT | S100A10 | SHC1 | SEMA3C | OXTR | BTK | CCL20 | PSMD2 | ADIPOR2 | TNFRSF11A | SCG2 | GPI
13 genes	9.99E-16	INSL4 | F2RL1 | RFXAP | FCGRT | S100A10 | SHC1 | PSMC1 | CCL20 | FURIN | PSMD2 | ADIPOR2 | TNFRSF11A | SCG2
14 genes	9.99E-16	DKK1 | VEGFC | RFXAP | SHC1 | SEMA3C | OXTR | BTK | CCL20 | FURIN | PSMD2 | ADIPOR2 | TNFRSF11A | SCG2 | GPI
14 genes	9.99E-16	DKK1 | INSL4 | RFXAP | FCGRT | CCR6 | S100A10 | SHC1 | OXTR | BTK | CCL20 | FURIN | PSMD2 | SCG2 | GPI
14 genes	9.99E-16	DKK1 | INSL4 | SHC1 | SEMA3C | OXTR | BTK | PSMC1 | CCL20 | FURIN | PSMD2 | ADIPOR2 | TNFRSF11A | SCG2 | GPI
14 genes	9.99E-16	DKK1 | FCGRT | CCR6 | S100A10 | SHC1 | SEMA3C | BTK | CCL20 | FURIN | PSMD2 | ADIPOR2 | TNFRSF11A | SCG2 | GPI
15 genes	9.99E-16	DKK1 | INSL4 | FCGRT | CCR6 | S100A10 | SHC1 | SEMA3C | BTK | CCL20 | FURIN | PSMD2 | ADIPOR2 | TNFRSF11A | SCG2 | GPI
17 genes	9.99E-16	DKK1 | VEGFC | INSL4 | F2RL1 | RFXAP | FCGRT | CCR6 | S100A10 | SHC1 | OXTR | BTK | CCL20 | FURIN | PSMD2 | ADIPOR2 | SCG2 | GPI
9 genes	1.11E-15	DKK1 | S100A10 | SHC1 | OXTR | BTK | CCL20 | PSMD2 | TNFRSF11A | SCG2
9 genes	1.11E-15	F2RL1 | RFXAP | FCGRT | S100A10 | PSMC1 | CCL20 | FURIN | PSMD2 | SCG2
10 genes	1.11E-15	DKK1 | FCGRT | SHC1 | SEMA3C | OXTR | BTK | CCL20 | TNFRSF11A | SCG2 | GPI
10 genes	1.11E-15	DKK1 | CCR6 | S100A10 | SHC1 | OXTR | BTK | CCL20 | PSMD2 | TNFRSF11A | SCG2
10 genes	1.11E-15	DKK1 | CCR6 | S100A10 | SHC1 | OXTR | PSMC1 | CCL20 | ADIPOR2 | TNFRSF11A | SCG2
10 genes	1.11E-15	F2RL1 | RFXAP | FCGRT | CCR6 | S100A10 | SEMA3C | OXTR | CCL20 | FURIN | PSMD2
11 genes	1.11E-15	DKK1 | INSL4 | CCR6 | S100A10 | SEMA3C | BTK | PSMC1 | CCL20 | TNFRSF11A | SCG2 | GPI
11 genes	1.11E-15	DKK1 | INSL4 | CCR6 | SHC1 | SEMA3C | PSMC1 | FURIN | PSMD2 | ADIPOR2 | TNFRSF11A | SCG2
11 genes	1.11E-15	DKK1 | INSL4 | CCR6 | SEMA3C | OXTR | CCL20 | FURIN | PSMD2 | TNFRSF11A | SCG2 | GPI
11 genes	1.11E-15	DKK1 | INSL4 | S100A10 | SHC1 | SEMA3C | BTK | CCL20 | FURIN | TNFRSF11A | SCG2 | GPI
11 genes	1.11E-15	DKK1 | INSL4 | S100A10 | SHC1 | OXTR | BTK | CCL20 | ADIPOR2 | TNFRSF11A | SCG2 | GPI
11 genes	1.11E-15	DKK1 | FCGRT | CCR6 | S100A10 | SHC1 | OXTR | PSMC1 | CCL20 | ADIPOR2 | TNFRSF11A | SCG2
11 genes	1.11E-15	DKK1 | CCR6 | S100A10 | SHC1 | SEMA3C | OXTR | PSMC1 | CCL20 | ADIPOR2 | TNFRSF11A | SCG2

11 genes	1.11E-15	DKK1 | S100A10 | SHC1 | SEMA3C | BTK | CCL20 | FURIN | ADIPOR2 | TNFRSF11A | SCG2 | GPI
11 genes	1.11E-15	VEGFC | F2RL1 | RFXAP | FCGRT | CCR6 | PSMC1 | CCL20 | FURIN | PSMD2 | SCG2 | GPI
11 genes	1.11E-15	INSL4 | F2RL1 | RFXAP | FCGRT | CCR6 | S100A10 | CCL20 | FURIN | PSMD2 | ADIPOR2 | SCG2
11 genes	1.11E-15	INSL4 | F2RL1 | CCR6 | SHC1 | OXTR | PSMC1 | CCL20 | FURIN | PSMD2 | TNFRSF11A | SCG2
11 genes	1.11E-15	F2RL1 | RFXAP | FCGRT | CCR6 | S100A10 | SHC1 | CCL20 | FURIN | PSMD2 | TNFRSF11A | SCG2
11 genes	1.11E-15	F2RL1 | RFXAP | FCGRT | CCR6 | S100A10 | PSMC1 | CCL20 | FURIN | ADIPOR2 | SCG2 | GPI
11 genes	1.11E-15	F2RL1 | RFXAP | FCGRT | S100A10 | SHC1 | PSMC1 | CCL20 | FURIN | PSMD2 | TNFRSF11A | SCG2
11 genes	1.11E-15	F2RL1 | FCGRT | CCR6 | SEMA3C | OXTR | CCL20 | FURIN | ADIPOR2 | TNFRSF11A | SCG2 | GPI
12 genes	1.11E-15	DKK1 | INSL4 | FCGRT | CCR6 | SHC1 | SEMA3C | PSMC1 | FURIN | PSMD2 | ADIPOR2 | TNFRSF11A | SCG2
12 genes	1.11E-15	DKK1 | FCGRT | CCR6 | S100A10 | SHC1 | SEMA3C | OXTR | PSMC1 | CCL20 | ADIPOR2 | TNFRSF11A | SCG2
12 genes	1.11E-15	DKK1 | FCGRT | S100A10 | SHC1 | SEMA3C | OXTR | BTK | PSMC1 | CCL20 | TNFRSF11A | SCG2 | GPI
12 genes	1.11E-15	DKK1 | FCGRT | S100A10 | SHC1 | OXTR | BTK | CCL20 | FURIN | PSMD2 | ADIPOR2 | TNFRSF11A | SCG2
12 genes	1.11E-15	DKK1 | CCR6 | S100A10 | SHC1 | OXTR | BTK | PSMC1 | CCL20 | FURIN | PSMD2 | TNFRSF11A | SCG2
12 genes	1.11E-15	DKK1 | S100A10 | SHC1 | SEMA3C | OXTR | BTK | PSMC1 | CCL20 | FURIN | PSMD2 | ADIPOR2 | SCG2
12 genes	1.11E-15	FCGRT | CCR6 | S100A10 | SHC1 | SEMA3C | BTK | CCL20 | FURIN | ADIPOR2 | TNFRSF11A | SCG2 | GPI
13 genes	1.11E-15	DKK1 | INSL4 | CCR6 | S100A10 | SHC1 | SEMA3C | OXTR | BTK | PSMC1 | CCL20 | TNFRSF11A | SCG2 | GPI
13 genes	1.11E-15	DKK1 | INSL4 | CCR6 | S100A10 | SHC1 | OXTR | BTK | CCL20 | PSMD2 | ADIPOR2 | TNFRSF11A | SCG2 | GPI
13 genes	1.11E-15	DKK1 | INSL4 | CCR6 | SHC1 | SEMA3C | OXTR | BTK | CCL20 | FURIN | PSMD2 | ADIPOR2 | TNFRSF11A | SCG2
13 genes	1.11E-15	DKK1 | INSL4 | S100A10 | SHC1 | SEMA3C | OXTR | BTK | PSMC1 | CCL20 | FURIN | PSMD2 | ADIPOR2 | SCG2
13 genes	1.11E-15	DKK1 | FCGRT | CCR6 | S100A10 | SEMA3C | OXTR | BTK | CCL20 | PSMD2 | ADIPOR2 | TNFRSF11A | SCG2 | GPI
13 genes	1.11E-15	DKK1 | S100A10 | SHC1 | SEMA3C | OXTR | BTK | PSMC1 | CCL20 | PSMD2 | ADIPOR2 | TNFRSF11A | SCG2 | GPI
13 genes	1.11E-15	VEGFC | INSL4 | F2RL1 | RFXAP | CCR6 | S100A10 | SHC1 | OXTR | PSMC1 | FURIN | PSMD2 | ADIPOR2 | SCG2
14 genes	1.11E-15	DKK1 | FCGRT | CCR6 | S100A10 | SHC1 | SEMA3C | OXTR | CCL20 | FURIN | PSMD2 | ADIPOR2 | TNFRSF11A | SCG2 | GPI
14 genes	1.11E-15	DKK1 | CCR6 | S100A10 | SHC1 | SEMA3C | OXTR | BTK | PSMC1 | CCL20 | FURIN | PSMD2 | TNFRSF11A | SCG2 | GPI
9 genes	1.22E-15	VEGFC | INSL4 | F2RL1 | CCR6 | SHC1 | OXTR | CCL20 | FURIN | PSMD2
9 genes	1.22E-15	VEGFC | SEMA3C | BTK | CCL20 | FURIN | PSMD2 | TNFRSF11A | SCG2 | GPI
9 genes	1.22E-15	INSL4 | F2RL1 | RFXAP | FCGRT | CCR6 | OXTR | CCL20 | FURIN | PSMD2
9 genes	1.22E-15	F2RL1 | FCGRT | CCR6 | OXTR | PSMC1 | CCL20 | PSMD2 | ADIPOR2 | SCG2
10 genes	1.22E-15	DKK1 | F2RL1 | FCGRT | CCR6 | SHC1 | PSMC1 | FURIN | ADIPOR2 | TNFRSF11A | SCG2
10 genes	1.22E-15	DKK1 | CCR6 | SHC1 | SEMA3C | BTK | CCL20 | ADIPOR2 | TNFRSF11A | SCG2 | GPI
10 genes	1.22E-15	DKK1 | S100A10 | SHC1 | OXTR | BTK | CCL20 | FURIN | PSMD2 | TNFRSF11A | SCG2
11 genes	1.22E-15	DKK1 | INSL4 | FCGRT | SHC1 | SEMA3C | OXTR | BTK | CCL20 | TNFRSF11A | SCG2 | GPI

11 genes	1.22E-15	DKK1 | F2RL1 | CCR6 | SHC1 | OXTR | BTK | CCL20 | FURIN | TNFRSF11A | SCG2 | GPI
11 genes	1.22E-15	DKK1 | CCR6 | S100A10 | SHC1 | OXTR | BTK | CCL20 | ADIPOR2 | TNFRSF11A | SCG2 | GPI
11 genes	1.22E-15	DKK1 | S100A10 | SHC1 | SEMA3C | OXTR | BTK | CCL20 | FURIN | PSMD2 | ADIPOR2 | SCG2
11 genes	1.22E-15	INSL4 | F2RL1 | RFXAP | FCGRT | CCR6 | S100A10 | BTK | CCL20 | PSMD2 | ADIPOR2 | SCG2
11 genes	1.22E-15	INSL4 | F2RL1 | RFXAP | CCR6 | S100A10 | SHC1 | CCL20 | FURIN | PSMD2 | TNFRSF11A | SCG2
11 genes	1.22E-15	F2RL1 | CCR6 | S100A10 | SHC1 | BTK | PSMC1 | CCL20 | FURIN | ADIPOR2 | TNFRSF11A | GPI
12 genes	1.22E-15	DKK1 | INSL4 | FCGRT | SHC1 | SEMA3C | OXTR | BTK | CCL20 | ADIPOR2 | TNFRSF11A | SCG2 | GPI
12 genes	1.22E-15	DKK1 | F2RL1 | FCGRT | CCR6 | SHC1 | OXTR | BTK | CCL20 | FURIN | TNFRSF11A | SCG2 | GPI
12 genes	1.22E-15	DKK1 | CCR6 | S100A10 | SHC1 | OXTR | PSMC1 | CCL20 | FURIN | PSMD2 | TNFRSF11A | SCG2 | GPI
12 genes	1.22E-15	DKK1 | CCR6 | SHC1 | SEMA3C | OXTR | BTK | CCL20 | PSMD2 | ADIPOR2 | TNFRSF11A | SCG2 | GPI
12 genes	1.22E-15	DKK1 | S100A10 | SHC1 | SEMA3C | OXTR | BTK | PSMC1 | CCL20 | PSMD2 | TNFRSF11A | SCG2 | GPI
12 genes	1.22E-15	INSL4 | F2RL1 | RFXAP | FCGRT | CCR6 | PSMC1 | CCL20 | FURIN | PSMD2 | TNFRSF11A | SCG2 | GPI
12 genes	1.22E-15	F2RL1 | RFXAP | FCGRT | CCR6 | S100A10 | OXTR | CCL20 | FURIN | PSMD2 | ADIPOR2 | SCG2 | GPI
13 genes	1.22E-15	DKK1 | VEGFC | FCGRT | S100A10 | SHC1 | SEMA3C | OXTR | BTK | PSMC1 | CCL20 | PSMD2 | ADIPOR2 | SCG2
13 genes	1.22E-15	DKK1 | F2RL1 | FCGRT | CCR6 | SHC1 | SEMA3C | OXTR | PSMC1 | CCL20 | ADIPOR2 | TNFRSF11A | SCG2 | GPI
13 genes	1.22E-15	DKK1 | FCGRT | CCR6 | S100A10 | SHC1 | OXTR | PSMC1 | CCL20 | FURIN | PSMD2 | TNFRSF11A | SCG2 | GPI
13 genes	1.22E-15	DKK1 | CCR6 | S100A10 | SEMA3C | OXTR | BTK | PSMC1 | CCL20 | PSMD2 | ADIPOR2 | TNFRSF11A | SCG2 | GPI
13 genes	1.22E-15	DKK1 | S100A10 | SHC1 | SEMA3C | OXTR | BTK | PSMC1 | CCL20 | FURIN | PSMD2 | TNFRSF11A | SCG2 | GPI
13 genes	1.22E-15	INSL4 | F2RL1 | RFXAP | FCGRT | CCR6 | SHC1 | OXTR | BTK | CCL20 | FURIN | PSMD2 | TNFRSF11A | SCG2
13 genes	1.22E-15	F2RL1 | RFXAP | FCGRT | CCR6 | S100A10 | SHC1 | OXTR | BTK | CCL20 | FURIN | PSMD2 | TNFRSF11A | SCG2
14 genes	1.22E-15	DKK1 | INSL4 | FCGRT | CCR6 | S100A10 | SEMA3C | OXTR | BTK | CCL20 | FURIN | PSMD2 | ADIPOR2 | TNFRSF11A | SCG2
14 genes	1.22E-15	DKK1 | INSL4 | S100A10 | SHC1 | SEMA3C | OXTR | BTK | PSMC1 | CCL20 | FURIN | PSMD2 | TNFRSF11A | SCG2 | GPI
16 genes	1.22E-15	DKK1 | INSL4 | CCR6 | S100A10 | SHC1 | SEMA3C | OXTR | BTK | PSMC1 | CCL20 | FURIN | PSMD2 | ADIPOR2 | TNFRSF11A | SCG2 | GPI
9 genes	1.33E-15	DKK1 | INSL4 | F2RL1 | CCR6 | SEMA3C | CCL20 | TNFRSF11A | SCG2 | GPI
9 genes	1.33E-15	DKK1 | RFXAP | FCGRT | S100A10 | OXTR | BTK | CCL20 | ADIPOR2 | SCG2
10 genes	1.33E-15	DKK1 | INSL4 | F2RL1 | CCR6 | SEMA3C | PSMC1 | CCL20 | TNFRSF11A | SCG2 | GPI
10 genes	1.33E-15	DKK1 | INSL4 | CCR6 | SHC1 | OXTR | BTK | CCL20 | TNFRSF11A | SCG2 | GPI
10 genes	1.33E-15	DKK1 | INSL4 | SHC1 | OXTR | BTK | CCL20 | FURIN | TNFRSF11A | SCG2 | GPI
10 genes	1.33E-15	DKK1 | RFXAP | FCGRT | S100A10 | OXTR | BTK | CCL20 | FURIN | ADIPOR2 | SCG2
10 genes	1.33E-15	DKK1 | CCR6 | SEMA3C | OXTR | BTK | PSMC1 | CCL20 | TNFRSF11A | SCG2 | GPI
10 genes	1.33E-15	DKK1 | S100A10 | SHC1 | OXTR | BTK | CCL20 | FURIN | PSMD2 | ADIPOR2 | SCG2
11 genes	1.33E-15	DKK1 | INSL4 | F2RL1 | FCGRT | CCR6 | SEMA3C | PSMC1 | CCL20 | TNFRSF11A | SCG2 | GPI

11 genes	1.33E-15	DKK1 | INSL4 | CCR6 | SHC1 | SEMA3C | BTK | CCL20 | ADIPOR2 | TNFRSF11A | SCG2 | GPI
11 genes	1.33E-15	DKK1 | FCGRT | CCR6 | SEMA3C | OXTR | BTK | CCL20 | PSMD2 | TNFRSF11A | SCG2 | GPI
11 genes	1.33E-15	DKK1 | CCR6 | S100A10 | OXTR | BTK | CCL20 | FURIN | PSMD2 | TNFRSF11A | SCG2 | GPI
11 genes	1.33E-15	DKK1 | S100A10 | SHC1 | SEMA3C | BTK | CCL20 | PSMD2 | ADIPOR2 | TNFRSF11A | SCG2 | GPI
12 genes	1.33E-15	DKK1 | INSL4 | FCGRT | S100A10 | SHC1 | OXTR | BTK | CCL20 | PSMD2 | ADIPOR2 | TNFRSF11A | SCG2
12 genes	1.33E-15	DKK1 | INSL4 | CCR6 | S100A10 | SHC1 | SEMA3C | OXTR | BTK | CCL20 | TNFRSF11A | SCG2 | GPI
12 genes	1.33E-15	DKK1 | F2RL1 | FCGRT | CCR6 | SHC1 | SEMA3C | PSMC1 | FURIN | PSMD2 | ADIPOR2 | TNFRSF11A | SCG2
12 genes	1.33E-15	DKK1 | FCGRT | CCR6 | SEMA3C | OXTR | BTK | CCL20 | PSMD2 | ADIPOR2 | TNFRSF11A | SCG2 | GPI
12 genes	1.33E-15	INSL4 | F2RL1 | RFXAP | FCGRT | CCR6 | S100A10 | OXTR | BTK | CCL20 | FURIN | PSMD2 | SCG2
13 genes	1.33E-15	DKK1 | INSL4 | FCGRT | CCR6 | S100A10 | SHC1 | OXTR | BTK | CCL20 | PSMD2 | TNFRSF11A | SCG2 | GPI
13 genes	1.33E-15	INSL4 | F2RL1 | RFXAP | CCR6 | S100A10 | SHC1 | PSMC1 | CCL20 | PSMD2 | ADIPOR2 | TNFRSF11A | SCG2 | GPI
13 genes	1.33E-15	INSL4 | F2RL1 | RFXAP | CCR6 | S100A10 | OXTR | BTK | CCL20 | FURIN | PSMD2 | ADIPOR2 | SCG2 | GPI
14 genes	1.33E-15	DKK1 | S100A10 | SHC1 | SEMA3C | OXTR | BTK | PSMC1 | CCL20 | FURIN | PSMD2 | ADIPOR2 | TNFRSF11A | SCG2 | GPI
14 genes	1.33E-15	INSL4 | F2RL1 | RFXAP | CCR6 | S100A10 | SHC1 | SEMA3C | OXTR | BTK | CCL20 | FURIN | PSMD2 | ADIPOR2 | TNFRSF11A
15 genes	1.33E-15	DKK1 | INSL4 | FCGRT | CCR6 | S100A10 | SHC1 | SEMA3C | OXTR | BTK | CCL20 | FURIN | PSMD2 | TNFRSF11A | SCG2 | GPI
15 genes	1.33E-15	DKK1 | INSL4 | CCR6 | S100A10 | SHC1 | SEMA3C | OXTR | BTK | PSMC1 | CCL20 | FURIN | PSMD2 | TNFRSF11A | SCG2 | GPI
15 genes	1.33E-15	DKK1 | CCR6 | S100A10 | SHC1 | SEMA3C | OXTR | BTK | PSMC1 | CCL20 | FURIN | PSMD2 | ADIPOR2 | TNFRSF11A | SCG2 | GPI
9 genes	1.44E-15	INSL4 | F2RL1 | FCGRT | CCR6 | S100A10 | CCL20 | FURIN | PSMD2 | ADIPOR2
10 genes	1.44E-15	DKK1 | S100A10 | SHC1 | SEMA3C | BTK | CCL20 | ADIPOR2 | TNFRSF11A | SCG2 | GPI
10 genes	1.44E-15	INSL4 | F2RL1 | RFXAP | CCR6 | OXTR | BTK | PSMC1 | CCL20 | FURIN | SCG2
10 genes	1.44E-15	INSL4 | F2RL1 | FCGRT | CCR6 | S100A10 | BTK | CCL20 | FURIN | PSMD2 | ADIPOR2
10 genes	1.44E-15	INSL4 | F2RL1 | FCGRT | CCR6 | SEMA3C | PSMC1 | CCL20 | FURIN | PSMD2 | GPI
10 genes	1.44E-15	F2RL1 | FCGRT | CCR6 | SEMA3C | BTK | PSMC1 | CCL20 | FURIN | PSMD2 | GPI
11 genes	1.44E-15	DKK1 | INSL4 | F2RL1 | FCGRT | CCR6 | SEMA3C | PSMC1 | CCL20 | PSMD2 | TNFRSF11A | GPI
11 genes	1.44E-15	DKK1 | INSL4 | CCR6 | SEMA3C | OXTR | BTK | CCL20 | ADIPOR2 | TNFRSF11A | SCG2 | GPI
11 genes	1.44E-15	DKK1 | INSL4 | S100A10 | SHC1 | SEMA3C | BTK | CCL20 | ADIPOR2 | TNFRSF11A | SCG2 | GPI
11 genes	1.44E-15	DKK1 | INSL4 | SHC1 | OXTR | BTK | CCL20 | FURIN | PSMD2 | TNFRSF11A | SCG2 | GPI
11 genes	1.44E-15	DKK1 | INSL4 | SHC1 | OXTR | BTK | CCL20 | PSMD2 | ADIPOR2 | TNFRSF11A | SCG2 | GPI
11 genes	1.44E-15	DKK1 | RFXAP | FCGRT | S100A10 | OXTR | BTK | CCL20 | FURIN | PSMD2 | ADIPOR2 | SCG2
11 genes	1.44E-15	VEGFC | F2RL1 | RFXAP | FCGRT | CCR6 | PSMC1 | CCL20 | PSMD2 | ADIPOR2 | SCG2 | GPI
11 genes	1.44E-15	F2RL1 | RFXAP | FCGRT | S100A10 | PSMC1 | CCL20 | FURIN | PSMD2 | ADIPOR2 | SCG2 | GPI
11 genes	1.44E-15	F2RL1 | CCR6 | SHC1 | OXTR | BTK | PSMC1 | CCL20 | FURIN | PSMD2 | TNFRSF11A | SCG2

11 genes	1.44E-15	CCR6 | S100A10 | SHC1 | SEMA3C | BTK | CCL20 | FURIN | ADIPOR2 | TNFRSF11A | SCG2 | GPI
12 genes	1.44E-15	DKK1 | INSL4 | FCGRT | S100A10 | SHC1 | SEMA3C | BTK | CCL20 | FURIN | TNFRSF11A | SCG2 | GPI
12 genes	1.44E-15	DKK1 | INSL4 | CCR6 | S100A10 | SEMA3C | OXTR | BTK | CCL20 | ADIPOR2 | TNFRSF11A | SCG2 | GPI
12 genes	1.44E-15	DKK1 | F2RL1 | RFXAP | FCGRT | CCR6 | SHC1 | PSMC1 | FURIN | PSMD2 | ADIPOR2 | TNFRSF11A | SCG2
12 genes	1.44E-15	DKK1 | F2RL1 | RFXAP | CCR6 | S100A10 | SHC1 | OXTR | CCL20 | FURIN | PSMD2 | ADIPOR2 | SCG2
12 genes	1.44E-15	DKK1 | S100A10 | SHC1 | SEMA3C | OXTR | BTK | PSMC1 | CCL20 | ADIPOR2 | TNFRSF11A | SCG2 | GPI
13 genes	1.44E-15	DKK1 | INSL4 | F2RL1 | RFXAP | FCGRT | CCR6 | S100A10 | SEMA3C | BTK | PSMC1 | CCL20 | PSMD2 | GPI
13 genes	1.44E-15	DKK1 | INSL4 | F2RL1 | RFXAP | CCR6 | S100A10 | SHC1 | OXTR | CCL20 | FURIN | PSMD2 | ADIPOR2 | SCG2
13 genes	1.44E-15	DKK1 | INSL4 | FCGRT | S100A10 | SHC1 | SEMA3C | OXTR | BTK | CCL20 | ADIPOR2 | TNFRSF11A | SCG2 | GPI
13 genes	1.44E-15	DKK1 | F2RL1 | RFXAP | FCGRT | CCR6 | S100A10 | SHC1 | OXTR | CCL20 | FURIN | PSMD2 | ADIPOR2 | SCG2
13 genes	1.44E-15	DKK1 | FCGRT | CCR6 | S100A10 | SHC1 | OXTR | BTK | PSMC1 | CCL20 | PSMD2 | TNFRSF11A | SCG2 | GPI
14 genes	1.44E-15	DKK1 | INSL4 | F2RL1 | RFXAP | FCGRT | CCR6 | S100A10 | SHC1 | OXTR | CCL20 | FURIN | PSMD2 | ADIPOR2 | SCG2
14 genes	1.44E-15	DKK1 | INSL4 | CCR6 | S100A10 | SHC1 | SEMA3C | OXTR | BTK | PSMC1 | CCL20 | PSMD2 | TNFRSF11A | SCG2 | GPI
14 genes	1.44E-15	DKK1 | F2RL1 | RFXAP | FCGRT | CCR6 | S100A10 | SHC1 | OXTR | BTK | CCL20 | PSMD2 | ADIPOR2 | SCG2 | GPI
9 genes	1.55E-15	DKK1 | SHC1 | SEMA3C | BTK | CCL20 | FURIN | TNFRSF11A | SCG2 | GPI
9 genes	1.55E-15	VEGFC | FCGRT | CCR6 | SEMA3C | OXTR | CCL20 | FURIN | TNFRSF11A | GPI
10 genes	1.55E-15	DKK1 | INSL4 | SHC1 | SEMA3C | BTK | CCL20 | FURIN | TNFRSF11A | SCG2 | GPI
10 genes	1.55E-15	DKK1 | F2RL1 | CCR6 | SEMA3C | OXTR | BTK | CCL20 | TNFRSF11A | SCG2 | GPI
10 genes	1.55E-15	F2RL1 | CCR6 | S100A10 | SHC1 | PSMC1 | CCL20 | FURIN | PSMD2 | ADIPOR2 | SCG2
11 genes	1.55E-15	DKK1 | INSL4 | CCR6 | S100A10 | SEMA3C | OXTR | BTK | CCL20 | TNFRSF11A | SCG2 | GPI
11 genes	1.55E-15	DKK1 | INSL4 | CCR6 | SHC1 | SEMA3C | OXTR | BTK | CCL20 | TNFRSF11A | SCG2 | GPI
11 genes	1.55E-15	DKK1 | FCGRT | CCR6 | SEMA3C | BTK | CCL20 | FURIN | PSMD2 | TNFRSF11A | SCG2 | GPI
11 genes	1.55E-15	DKK1 | CCR6 | S100A10 | SHC1 | OXTR | PSMC1 | CCL20 | PSMD2 | TNFRSF11A | SCG2 | GPI
11 genes	1.55E-15	INSL4 | F2RL1 | RFXAP | FCGRT | CCR6 | S100A10 | OXTR | BTK | CCL20 | FURIN | PSMD2
12 genes	1.55E-15	DKK1 | INSL4 | F2RL1 | RFXAP | CCR6 | SEMA3C | PSMC1 | CCL20 | ADIPOR2 | TNFRSF11A | SCG2 | GPI
12 genes	1.55E-15	DKK1 | INSL4 | FCGRT | CCR6 | SEMA3C | OXTR | CCL20 | FURIN | PSMD2 | TNFRSF11A | SCG2 | GPI
12 genes	1.55E-15	DKK1 | INSL4 | CCR6 | S100A10 | SEMA3C | OXTR | BTK | CCL20 | PSMD2 | TNFRSF11A | SCG2 | GPI
12 genes	1.55E-15	DKK1 | F2RL1 | CCR6 | SHC1 | SEMA3C | OXTR | BTK | CCL20 | ADIPOR2 | TNFRSF11A | SCG2 | GPI
12 genes	1.55E-15	DKK1 | CCR6 | S100A10 | SHC1 | OXTR | BTK | CCL20 | PSMD2 | ADIPOR2 | TNFRSF11A | SCG2 | GPI
12 genes	1.55E-15	F2RL1 | CCR6 | SEMA3C | OXTR | BTK | PSMC1 | CCL20 | FURIN | PSMD2 | ADIPOR2 | TNFRSF11A | GPI
13 genes	1.55E-15	DKK1 | INSL4 | F2RL1 | CCR6 | SHC1 | SEMA3C | OXTR | BTK | CCL20 | ADIPOR2 | TNFRSF11A | SCG2 | GPI
13 genes	1.55E-15	DKK1 | INSL4 | CCR6 | S100A10 | SHC1 | SEMA3C | OXTR | BTK | CCL20 | PSMD2 | TNFRSF11A | SCG2 | GPI

13 genes	1.55E-15	DKK1 | F2RL1 | FCGRT | CCR6 | S100A10 | SHC1 | BTK | PSMC1 | CCL20 | FURIN | TNFRSF11A | SCG2 | GPI
13 genes	1.55E-15	DKK1 | RFXAP | CCR6 | SHC1 | SEMA3C | OXTR | BTK | CCL20 | FURIN | PSMD2 | TNFRSF11A | SCG2 | GPI
13 genes	1.55E-15	DKK1 | CCR6 | S100A10 | SHC1 | SEMA3C | OXTR | BTK | PSMC1 | CCL20 | FURIN | TNFRSF11A | SCG2 | GPI
13 genes	1.55E-15	VEGFC | INSL4 | F2RL1 | RFXAP | CCR6 | S100A10 | SEMA3C | OXTR | BTK | CCL20 | PSMD2 | SCG2 | GPI
14 genes	1.55E-15	DKK1 | INSL4 | F2RL1 | FCGRT | CCR6 | S100A10 | SHC1 | BTK | PSMC1 | CCL20 | FURIN | TNFRSF11A | SCG2 | GPI
14 genes	1.55E-15	DKK1 | INSL4 | RFXAP | FCGRT | CCR6 | S100A10 | SHC1 | OXTR | BTK | CCL20 | FURIN | PSMD2 | ADIPOR2 | SCG2
14 genes	1.55E-15	DKK1 | INSL4 | CCR6 | S100A10 | SHC1 | SEMA3C | OXTR | BTK | PSMC1 | CCL20 | FURIN | TNFRSF11A | SCG2 | GPI
14 genes	1.55E-15	DKK1 | INSL4 | CCR6 | S100A10 | SHC1 | SEMA3C | OXTR | BTK | PSMC1 | CCL20 | PSMD2 | ADIPOR2 | TNFRSF11A | SCG2
14 genes	1.55E-15	DKK1 | CCR6 | S100A10 | SHC1 | SEMA3C | OXTR | BTK | PSMC1 | CCL20 | PSMD2 | ADIPOR2 | TNFRSF11A | SCG2 | GPI
14 genes	1.55E-15	VEGFC | INSL4 | F2RL1 | RFXAP | FCGRT | CCR6 | S100A10 | SHC1 | OXTR | PSMC1 | FURIN | PSMD2 | ADIPOR2 | SCG2
15 genes	1.55E-15	DKK1 | INSL4 | CCR6 | S100A10 | SHC1 | SEMA3C | OXTR | BTK | PSMC1 | CCL20 | PSMD2 | ADIPOR2 | TNFRSF11A | SCG2 | GPI
15 genes	1.55E-15	DKK1 | F2RL1 | RFXAP | FCGRT | CCR6 | S100A10 | SHC1 | OXTR | BTK | CCL20 | FURIN | PSMD2 | ADIPOR2 | SCG2 | GPI
15 genes	1.55E-15	VEGFC | INSL4 | F2RL1 | RFXAP | CCR6 | S100A10 | SEMA3C | OXTR | BTK | CCL20 | FURIN | PSMD2 | ADIPOR2 | SCG2 | GPI
15 genes	1.55E-15	VEGFC | INSL4 | RFXAP | FCGRT | CCR6 | S100A10 | SHC1 | SEMA3C | OXTR | BTK | CCL20 | PSMD2 | ADIPOR2 | TNFRSF11A | SCG2
8 genes	1.67E-15	DKK1 | F2RL1 | CCR6 | PSMC1 | CCL20 | TNFRSF11A | SCG2 | GPI
9 genes	1.67E-15	DKK1 | F2RL1 | FCGRT | CCR6 | SEMA3C | CCL20 | TNFRSF11A | SCG2 | GPI
9 genes	1.67E-15	DKK1 | CCR6 | S100A10 | OXTR | BTK | CCL20 | PSMD2 | TNFRSF11A | SCG2
9 genes	1.67E-15	DKK1 | CCR6 | SEMA3C | OXTR | BTK | CCL20 | TNFRSF11A | SCG2 | GPI
10 genes	1.67E-15	DKK1 | INSL4 | F2RL1 | RFXAP | CCR6 | PSMC1 | CCL20 | ADIPOR2 | TNFRSF11A | SCG2
10 genes	1.67E-15	F2RL1 | FCGRT | CCR6 | SEMA3C | CCL20 | PSMD2 | ADIPOR2 | TNFRSF11A | SCG2 | GPI
11 genes	1.67E-15	DKK1 | INSL4 | F2RL1 | RFXAP | CCR6 | OXTR | PSMC1 | CCL20 | FURIN | PSMD2 | SCG2
11 genes	1.67E-15	DKK1 | INSL4 | FCGRT | CCR6 | S100A10 | SHC1 | BTK | CCL20 | TNFRSF11A | SCG2 | GPI
11 genes	1.67E-15	DKK1 | INSL4 | CCR6 | S100A10 | SHC1 | OXTR | BTK | CCL20 | PSMD2 | TNFRSF11A | SCG2
11 genes	1.67E-15	DKK1 | INSL4 | SHC1 | OXTR | BTK | PSMC1 | CCL20 | PSMD2 | TNFRSF11A | SCG2 | GPI
11 genes	1.67E-15	DKK1 | F2RL1 | RFXAP | FCGRT | CCR6 | SHC1 | CCL20 | PSMD2 | ADIPOR2 | TNFRSF11A | SCG2
11 genes	1.67E-15	DKK1 | F2RL1 | FCGRT | CCR6 | S100A10 | PSMC1 | CCL20 | FURIN | PSMD2 | TNFRSF11A | GPI
11 genes	1.67E-15	DKK1 | F2RL1 | FCGRT | CCR6 | OXTR | PSMC1 | CCL20 | ADIPOR2 | TNFRSF11A | SCG2 | GPI
11 genes	1.67E-15	DKK1 | CCR6 | SHC1 | SEMA3C | OXTR | BTK | CCL20 | FURIN | PSMD2 | TNFRSF11A | SCG2
12 genes	1.67E-15	DKK1 | INSL4 | F2RL1 | RFXAP | CCR6 | OXTR | PSMC1 | CCL20 | FURIN | PSMD2 | ADIPOR2 | SCG2
12 genes	1.67E-15	DKK1 | INSL4 | F2RL1 | FCGRT | CCR6 | S100A10 | PSMC1 | CCL20 | FURIN | PSMD2 | TNFRSF11A | GPI
12 genes	1.67E-15	DKK1 | INSL4 | FCGRT | CCR6 | S100A10 | SEMA3C | OXTR | BTK | CCL20 | TNFRSF11A | SCG2 | GPI
12 genes	1.67E-15	DKK1 | INSL4 | CCR6 | S100A10 | SHC1 | OXTR | BTK | CCL20 | ADIPOR2 | TNFRSF11A | SCG2 | GPI

12 genes	1.67E-15	DKK1 | F2RL1 | RFXAP | CCR6 | S100A10 | SEMA3C | BTK | PSMC1 | CCL20 | PSMD2 | ADIPOR2 | GPI
12 genes	1.67E-15	DKK1 | FCGRT | CCR6 | S100A10 | SEMA3C | OXTR | BTK | CCL20 | FURIN | PSMD2 | TNFRSF11A | SCG2
12 genes	1.67E-15	DKK1 | CCR6 | S100A10 | SHC1 | OXTR | CCL20 | FURIN | PSMD2 | ADIPOR2 | TNFRSF11A | SCG2 | GPI
12 genes	1.67E-15	DKK1 | CCR6 | SHC1 | SEMA3C | OXTR | BTK | CCL20 | FURIN | PSMD2 | ADIPOR2 | TNFRSF11A | SCG2
12 genes	1.67E-15	INSL4 | F2RL1 | CCR6 | S100A10 | SHC1 | BTK | PSMC1 | CCL20 | FURIN | ADIPOR2 | TNFRSF11A | GPI
13 genes	1.67E-15	DKK1 | INSL4 | F2RL1 | RFXAP | CCR6 | S100A10 | BTK | PSMC1 | CCL20 | FURIN | ADIPOR2 | TNFRSF11A | GPI
13 genes	1.67E-15	DKK1 | INSL4 | FCGRT | CCR6 | S100A10 | SEMA3C | OXTR | BTK | CCL20 | FURIN | PSMD2 | TNFRSF11A | SCG2
13 genes	1.67E-15	DKK1 | INSL4 | FCGRT | S100A10 | SHC1 | SEMA3C | BTK | CCL20 | FURIN | PSMD2 | ADIPOR2 | SCG2 | GPI
13 genes	1.67E-15	DKK1 | FCGRT | CCR6 | S100A10 | SHC1 | SEMA3C | BTK | PSMC1 | CCL20 | PSMD2 | TNFRSF11A | SCG2 | GPI
13 genes	1.67E-15	INSL4 | F2RL1 | RFXAP | CCR6 | SHC1 | BTK | PSMC1 | CCL20 | FURIN | PSMD2 | TNFRSF11A | SCG2 | GPI
14 genes	1.67E-15	DKK1 | VEGFC | INSL4 | FCGRT | S100A10 | SHC1 | SEMA3C | BTK | CCL20 | FURIN | PSMD2 | ADIPOR2 | SCG2 | GPI
14 genes	1.67E-15	DKK1 | FCGRT | CCR6 | S100A10 | SHC1 | OXTR | BTK | PSMC1 | CCL20 | PSMD2 | ADIPOR2 | TNFRSF11A | SCG2 | GPI
14 genes	1.67E-15	INSL4 | F2RL1 | RFXAP | FCGRT | CCR6 | S100A10 | SHC1 | BTK | PSMC1 | FURIN | ADIPOR2 | TNFRSF11A | SCG2 | GPI
8 genes	1.78E-15	VEGFC | F2RL1 | CCR6 | OXTR | BTK | CCL20 | FURIN | PSMD2
8 genes	1.78E-15	F2RL1 | CCR6 | S100A10 | PSMC1 | CCL20 | FURIN | PSMD2 | ADIPOR2
9 genes	1.78E-15	INSL4 | F2RL1 | CCR6 | SHC1 | BTK | CCL20 | FURIN | TNFRSF11A | GPI
10 genes	1.78E-15	DKK1 | F2RL1 | RFXAP | CCR6 | SHC1 | OXTR | CCL20 | FURIN | PSMD2 | ADIPOR2
10 genes	1.78E-15	INSL4 | F2RL1 | RFXAP | FCGRT | CCR6 | PSMC1 | CCL20 | ADIPOR2 | SCG2 | GPI
10 genes	1.78E-15	F2RL1 | CCR6 | SHC1 | OXTR | BTK | CCL20 | FURIN | PSMD2 | TNFRSF11A | SCG2
11 genes	1.78E-15	DKK1 | VEGFC | SHC1 | SEMA3C | OXTR | BTK | CCL20 | PSMD2 | TNFRSF11A | SCG2 | GPI
11 genes	1.78E-15	DKK1 | INSL4 | F2RL1 | RFXAP | CCR6 | SHC1 | OXTR | CCL20 | FURIN | PSMD2 | ADIPOR2
11 genes	1.78E-15	DKK1 | F2RL1 | RFXAP | CCR6 | SHC1 | SEMA3C | OXTR | CCL20 | FURIN | PSMD2 | ADIPOR2
11 genes	1.78E-15	DKK1 | F2RL1 | FCGRT | CCR6 | BTK | PSMC1 | CCL20 | PSMD2 | ADIPOR2 | TNFRSF11A | GPI
11 genes	1.78E-15	DKK1 | FCGRT | S100A10 | SHC1 | SEMA3C | BTK | CCL20 | FURIN | TNFRSF11A | SCG2 | GPI
11 genes	1.78E-15	DKK1 | S100A10 | SHC1 | OXTR | BTK | CCL20 | FURIN | PSMD2 | ADIPOR2 | TNFRSF11A | SCG2
11 genes	1.78E-15	INSL4 | F2RL1 | RFXAP | FCGRT | CCR6 | SHC1 | CCL20 | FURIN | PSMD2 | TNFRSF11A | GPI
11 genes	1.78E-15	INSL4 | F2RL1 | RFXAP | FCGRT | CCR6 | BTK | CCL20 | PSMD2 | ADIPOR2 | SCG2 | GPI
12 genes	1.78E-15	DKK1 | VEGFC | SHC1 | SEMA3C | OXTR | BTK | CCL20 | PSMD2 | ADIPOR2 | TNFRSF11A | SCG2 | GPI
12 genes	1.78E-15	VEGFC | INSL4 | F2RL1 | RFXAP | CCR6 | S100A10 | SHC1 | OXTR | BTK | CCL20 | PSMD2 | SCG2
13 genes	1.78E-15	DKK1 | INSL4 | F2RL1 | RFXAP | FCGRT | CCR6 | SHC1 | SEMA3C | OXTR | CCL20 | FURIN | PSMD2 | ADIPOR2
13 genes	1.78E-15	DKK1 | INSL4 | FCGRT | CCR6 | S100A10 | SHC1 | OXTR | BTK | PSMC1 | CCL20 | TNFRSF11A | SCG2 | GPI
13 genes	1.78E-15	DKK1 | INSL4 | FCGRT | CCR6 | S100A10 | SHC1 | OXTR | CCL20 | FURIN | PSMD2 | TNFRSF11A | SCG2 | GPI

13 genes	1.78E-15	DKK1 | INSL4 | FCGRT | S100A10 | SHC1 | SEMA3C | OXTR | BTK | CCL20 | PSMD2 | TNFRSF11A | SCG2 | GPI
13 genes	1.78E-15	DKK1 | INSL4 | CCR6 | SHC1 | SEMA3C | OXTR | BTK | CCL20 | PSMD2 | ADIPOR2 | TNFRSF11A | SCG2 | GPI
13 genes	1.78E-15	DKK1 | CCR6 | SHC1 | SEMA3C | OXTR | BTK | CCL20 | FURIN | PSMD2 | ADIPOR2 | TNFRSF11A | SCG2 | GPI
13 genes	1.78E-15	VEGFC | INSL4 | F2RL1 | RFXAP | FCGRT | CCR6 | S100A10 | SHC1 | OXTR | BTK | CCL20 | PSMD2 | SCG2
13 genes	1.78E-15	VEGFC | CCR6 | SHC1 | SEMA3C | OXTR | BTK | CCL20 | FURIN | PSMD2 | ADIPOR2 | TNFRSF11A | SCG2 | GPI
13 genes	1.78E-15	F2RL1 | RFXAP | FCGRT | CCR6 | S100A10 | SHC1 | BTK | PSMC1 | CCL20 | FURIN | PSMD2 | SCG2 | GPI
14 genes	1.78E-15	DKK1 | INSL4 | CCR6 | S100A10 | SHC1 | SEMA3C | OXTR | BTK | PSMC1 | CCL20 | FURIN | ADIPOR2 | SCG2 | GPI
14 genes	1.78E-15	DKK1 | FCGRT | CCR6 | S100A10 | SHC1 | SEMA3C | OXTR | BTK | CCL20 | FURIN | PSMD2 | TNFRSF11A | SCG2 | GPI
16 genes	1.78E-15	DKK1 | INSL4 | F2RL1 | RFXAP | FCGRT | CCR6 | S100A10 | SHC1 | OXTR | BTK | CCL20 | FURIN | PSMD2 | ADIPOR2 | SCG2 | GPI
9 genes	1.89E-15	F2RL1 | CCR6 | CCL20 | FURIN | PSMD2 | ADIPOR2 | TNFRSF11A | SCG2 | GPI
10 genes	1.89E-15	DKK1 | INSL4 | F2RL1 | FCGRT | CCR6 | SEMA3C | CCL20 | TNFRSF11A | SCG2 | GPI
10 genes	1.89E-15	DKK1 | CCR6 | S100A10 | BTK | CCL20 | PSMD2 | ADIPOR2 | TNFRSF11A | SCG2 | GPI
10 genes	1.89E-15	DKK1 | CCR6 | SHC1 | SEMA3C | BTK | PSMC1 | CCL20 | TNFRSF11A | SCG2 | GPI
10 genes	1.89E-15	F2RL1 | RFXAP | FCGRT | S100A10 | CCL20 | FURIN | PSMD2 | ADIPOR2 | SCG2 | GPI
11 genes	1.89E-15	DKK1 | INSL4 | FCGRT | S100A10 | SHC1 | OXTR | BTK | CCL20 | FURIN | PSMD2 | TNFRSF11A
11 genes	1.89E-15	DKK1 | INSL4 | SHC1 | SEMA3C | BTK | CCL20 | FURIN | PSMD2 | TNFRSF11A | SCG2 | GPI
11 genes	1.89E-15	DKK1 | S100A10 | SHC1 | SEMA3C | OXTR | BTK | PSMC1 | CCL20 | PSMD2 | TNFRSF11A | SCG2
11 genes	1.89E-15	DKK1 | SHC1 | SEMA3C | OXTR | BTK | CCL20 | FURIN | PSMD2 | TNFRSF11A | SCG2 | GPI
11 genes	1.89E-15	VEGFC | F2RL1 | RFXAP | FCGRT | CCR6 | OXTR | BTK | CCL20 | FURIN | PSMD2 | GPI
11 genes	1.89E-15	F2RL1 | RFXAP | FCGRT | CCR6 | S100A10 | OXTR | BTK | CCL20 | FURIN | PSMD2 | SCG2
11 genes	1.89E-15	F2RL1 | RFXAP | FCGRT | CCR6 | BTK | PSMC1 | CCL20 | FURIN | PSMD2 | SCG2 | GPI
12 genes	1.89E-15	DKK1 | INSL4 | F2RL1 | CCR6 | SEMA3C | OXTR | BTK | CCL20 | ADIPOR2 | TNFRSF11A | SCG2 | GPI
12 genes	1.89E-15	DKK1 | FCGRT | CCR6 | S100A10 | SHC1 | SEMA3C | BTK | CCL20 | PSMD2 | TNFRSF11A | SCG2 | GPI
12 genes	1.89E-15	DKK1 | FCGRT | CCR6 | SHC1 | SEMA3C | OXTR | BTK | CCL20 | ADIPOR2 | TNFRSF11A | SCG2 | GPI
12 genes	1.89E-15	DKK1 | FCGRT | SHC1 | SEMA3C | BTK | CCL20 | FURIN | PSMD2 | ADIPOR2 | TNFRSF11A | SCG2 | GPI
12 genes	1.89E-15	DKK1 | CCR6 | S100A10 | SHC1 | SEMA3C | OXTR | BTK | PSMC1 | CCL20 | TNFRSF11A | SCG2 | GPI
13 genes	1.89E-15	DKK1 | INSL4 | FCGRT | CCR6 | S100A10 | SEMA3C | OXTR | BTK | PSMC1 | CCL20 | TNFRSF11A | SCG2 | GPI
13 genes	1.89E-15	DKK1 | INSL4 | FCGRT | CCR6 | SHC1 | SEMA3C | OXTR | BTK | CCL20 | ADIPOR2 | TNFRSF11A | SCG2 | GPI
13 genes	1.89E-15	DKK1 | CCR6 | S100A10 | SHC1 | SEMA3C | OXTR | PSMC1 | CCL20 | FURIN | PSMD2 | TNFRSF11A | SCG2 | GPI
13 genes	1.89E-15	INSL4 | F2RL1 | RFXAP | CCR6 | SHC1 | OXTR | BTK | PSMC1 | FURIN | PSMD2 | ADIPOR2 | TNFRSF11A | SCG2
13 genes	1.89E-15	F2RL1 | RFXAP | FCGRT | CCR6 | S100A10 | SHC1 | PSMC1 | CCL20 | FURIN | PSMD2 | ADIPOR2 | SCG2 | GPI
14 genes	1.89E-15	DKK1 | F2RL1 | RFXAP | FCGRT | CCR6 | S100A10 | SHC1 | SEMA3C | OXTR | CCL20 | FURIN | PSMD2 | ADIPOR2 | SCG2

15 genes	1.89E-15	DKK1 | INSL4 | F2RL1 | RFXAP | FCGRT | CCR6 | S100A10 | SHC1 | SEMA3C | OXTR | CCL20 | FURIN | PSMD2 | ADIPOR2 | SCG2
15 genes	1.89E-15	DKK1 | INSL4 | F2RL1 | CCR6 | SHC1 | OXTR | BTK | PSMC1 | CCL20 | FURIN | PSMD2 | ADIPOR2 | TNFRSF11A | SCG2 | GPI
10 genes	2.00E-15	DKK1 | F2RL1 | CCR6 | SHC1 | SEMA3C | FURIN | PSMD2 | ADIPOR2 | TNFRSF11A | SCG2
10 genes	2.00E-15	INSL4 | F2RL1 | RFXAP | FCGRT | CCR6 | BTK | CCL20 | PSMD2 | ADIPOR2 | SCG2
11 genes	2.00E-15	DKK1 | F2RL1 | CCR6 | SHC1 | OXTR | PSMC1 | CCL20 | ADIPOR2 | TNFRSF11A | SCG2 | GPI
11 genes	2.00E-15	DKK1 | F2RL1 | CCR6 | SEMA3C | OXTR | PSMC1 | CCL20 | ADIPOR2 | TNFRSF11A | SCG2 | GPI
11 genes	2.00E-15	DKK1 | FCGRT | S100A10 | SHC1 | SEMA3C | OXTR | BTK | CCL20 | PSMD2 | TNFRSF11A | SCG2
11 genes	2.00E-15	DKK1 | SHC1 | SEMA3C | OXTR | BTK | CCL20 | FURIN | ADIPOR2 | TNFRSF11A | SCG2 | GPI
11 genes	2.00E-15	INSL4 | F2RL1 | RFXAP | FCGRT | CCR6 | SHC1 | BTK | PSMC1 | FURIN | TNFRSF11A | SCG2
11 genes	2.00E-15	F2RL1 | RFXAP | FCGRT | CCR6 | S100A10 | OXTR | CCL20 | FURIN | PSMD2 | ADIPOR2 | SCG2
12 genes	2.00E-15	DKK1 | INSL4 | F2RL1 | CCR6 | SEMA3C | PSMC1 | CCL20 | FURIN | ADIPOR2 | TNFRSF11A | SCG2 | GPI
12 genes	2.00E-15	DKK1 | INSL4 | CCR6 | S100A10 | SHC1 | OXTR | BTK | CCL20 | FURIN | PSMD2 | TNFRSF11A | SCG2
12 genes	2.00E-15	DKK1 | INSL4 | CCR6 | S100A10 | SEMA3C | OXTR | BTK | CCL20 | PSMD2 | ADIPOR2 | TNFRSF11A | SCG2
12 genes	2.00E-15	DKK1 | INSL4 | SHC1 | SEMA3C | OXTR | BTK | CCL20 | FURIN | ADIPOR2 | TNFRSF11A | SCG2 | GPI
12 genes	2.00E-15	DKK1 | F2RL1 | FCGRT | CCR6 | SHC1 | OXTR | PSMC1 | CCL20 | ADIPOR2 | TNFRSF11A | SCG2 | GPI
12 genes	2.00E-15	DKK1 | F2RL1 | FCGRT | CCR6 | OXTR | PSMC1 | CCL20 | PSMD2 | ADIPOR2 | TNFRSF11A | SCG2 | GPI
12 genes	2.00E-15	DKK1 | F2RL1 | CCR6 | S100A10 | SHC1 | BTK | PSMC1 | CCL20 | FURIN | TNFRSF11A | SCG2 | GPI
12 genes	2.00E-15	VEGFC | F2RL1 | RFXAP | CCR6 | S100A10 | OXTR | BTK | CCL20 | PSMD2 | ADIPOR2 | SCG2 | GPI
12 genes	2.00E-15	INSL4 | F2RL1 | RFXAP | FCGRT | CCR6 | SHC1 | BTK | CCL20 | FURIN | PSMD2 | TNFRSF11A | GPI
12 genes	2.00E-15	F2RL1 | RFXAP | FCGRT | S100A10 | SHC1 | OXTR | BTK | CCL20 | FURIN | PSMD2 | TNFRSF11A | SCG2
13 genes	2.00E-15	DKK1 | INSL4 | F2RL1 | FCGRT | CCR6 | SEMA3C | PSMC1 | CCL20 | FURIN | ADIPOR2 | TNFRSF11A | SCG2 | GPI
13 genes	2.00E-15	DKK1 | INSL4 | F2RL1 | CCR6 | S100A10 | SHC1 | BTK | PSMC1 | CCL20 | FURIN | TNFRSF11A | SCG2 | GPI
13 genes	2.00E-15	DKK1 | INSL4 | FCGRT | CCR6 | S100A10 | SEMA3C | OXTR | BTK | PSMC1 | CCL20 | ADIPOR2 | TNFRSF11A | SCG2
14 genes	2.00E-15	DKK1 | RFXAP | S100A10 | SHC1 | SEMA3C | OXTR | BTK | CCL20 | FURIN | PSMD2 | ADIPOR2 | TNFRSF11A | SCG2 | GPI
15 genes	2.00E-15	DKK1 | VEGFC | F2RL1 | RFXAP | FCGRT | CCR6 | SHC1 | OXTR | BTK | CCL20 | FURIN | PSMD2 | ADIPOR2 | SCG2 | GPI
15 genes	2.00E-15	DKK1 | INSL4 | RFXAP | S100A10 | SHC1 | SEMA3C | OXTR | BTK | CCL20 | FURIN | PSMD2 | ADIPOR2 | TNFRSF11A | SCG2 | GPI
16 genes	2.00E-15	DKK1 | VEGFC | INSL4 | F2RL1 | RFXAP | FCGRT | CCR6 | SHC1 | OXTR | BTK | CCL20 | FURIN | PSMD2 | ADIPOR2 | SCG2 | GPI
16 genes	2.00E-15	DKK1 | FCGRT | CCR6 | S100A10 | SHC1 | SEMA3C | OXTR | BTK | PSMC1 | CCL20 | FURIN | PSMD2 | ADIPOR2 | TNFRSF11A | SCG2 | GPI
9 genes	2.11E-15	INSL4 | F2RL1 | CCR6 | SHC1 | CCL20 | FURIN | PSMD2 | TNFRSF11A | SCG2
10 genes	2.11E-15	DKK1 | F2RL1 | CCR6 | SEMA3C | OXTR | PSMC1 | CCL20 | TNFRSF11A | SCG2 | GPI
10 genes	2.11E-15	DKK1 | F2RL1 | CCR6 | SEMA3C | OXTR | CCL20 | ADIPOR2 | TNFRSF11A | SCG2 | GPI
10 genes	2.11E-15	DKK1 | SHC1 | SEMA3C | BTK | CCL20 | FURIN | PSMD2 | TNFRSF11A | SCG2 | GPI

10 genes	2.11E-15	INSL4 | F2RL1 | RFXAP | CCR6 | S100A10 | SEMA3C | PSMC1 | FURIN | ADIPOR2 | SCG2
10 genes	2.11E-15	F2RL1 | RFXAP | CCR6 | S100A10 | SEMA3C | PSMC1 | CCL20 | FURIN | PSMD2 | GPI
11 genes	2.11E-15	DKK1 | INSL4 | F2RL1 | CCR6 | SHC1 | SEMA3C | PSMC1 | FURIN | ADIPOR2 | TNFRSF11A | SCG2
11 genes	2.11E-15	DKK1 | F2RL1 | FCGRT | CCR6 | SHC1 | SEMA3C | FURIN | PSMD2 | ADIPOR2 | TNFRSF11A | SCG2
11 genes	2.11E-15	DKK1 | CCR6 | S100A10 | SEMA3C | OXTR | BTK | CCL20 | FURIN | PSMD2 | TNFRSF11A | SCG2
11 genes	2.11E-15	DKK1 | S100A10 | SHC1 | OXTR | BTK | PSMC1 | CCL20 | FURIN | PSMD2 | SCG2 | GPI
11 genes	2.11E-15	F2RL1 | RFXAP | FCGRT | CCR6 | S100A10 | OXTR | CCL20 | FURIN | PSMD2 | SCG2 | GPI
11 genes	2.11E-15	F2RL1 | RFXAP | FCGRT | CCR6 | OXTR | BTK | CCL20 | FURIN | PSMD2 | ADIPOR2 | GPI
12 genes	2.11E-15	DKK1 | INSL4 | FCGRT | CCR6 | SHC1 | BTK | PSMC1 | FURIN | PSMD2 | ADIPOR2 | TNFRSF11A | SCG2
12 genes	2.11E-15	DKK1 | INSL4 | FCGRT | S100A10 | SHC1 | OXTR | BTK | PSMC1 | CCL20 | FURIN | PSMD2 | SCG2
12 genes	2.11E-15	DKK1 | F2RL1 | FCGRT | CCR6 | SEMA3C | OXTR | PSMC1 | CCL20 | ADIPOR2 | TNFRSF11A | SCG2 | GPI
12 genes	2.11E-15	DKK1 | F2RL1 | CCR6 | SHC1 | SEMA3C | OXTR | PSMC1 | CCL20 | ADIPOR2 | TNFRSF11A | SCG2 | GPI
12 genes	2.11E-15	DKK1 | CCR6 | S100A10 | SEMA3C | OXTR | BTK | CCL20 | FURIN | PSMD2 | ADIPOR2 | TNFRSF11A | SCG2
13 genes	2.11E-15	DKK1 | INSL4 | CCR6 | S100A10 | SHC1 | OXTR | BTK | CCL20 | FURIN | PSMD2 | ADIPOR2 | TNFRSF11A | SCG2
13 genes	2.11E-15	DKK1 | F2RL1 | RFXAP | CCR6 | SHC1 | PSMC1 | CCL20 | FURIN | PSMD2 | ADIPOR2 | TNFRSF11A | SCG2 | GPI
13 genes	2.11E-15	DKK1 | FCGRT | CCR6 | S100A10 | SEMA3C | OXTR | BTK | CCL20 | FURIN | PSMD2 | ADIPOR2 | TNFRSF11A | SCG2
13 genes	2.11E-15	INSL4 | F2RL1 | RFXAP | FCGRT | CCR6 | S100A10 | OXTR | BTK | CCL20 | FURIN | PSMD2 | ADIPOR2 | GPI
14 genes	2.11E-15	DKK1 | INSL4 | FCGRT | CCR6 | S100A10 | SHC1 | OXTR | BTK | PSMC1 | CCL20 | PSMD2 | TNFRSF11A | SCG2 | GPI
14 genes	2.11E-15	VEGFC | F2RL1 | RFXAP | CCR6 | S100A10 | SEMA3C | OXTR | BTK | CCL20 | FURIN | PSMD2 | ADIPOR2 | SCG2 | GPI
14 genes	2.11E-15	INSL4 | F2RL1 | RFXAP | FCGRT | CCR6 | S100A10 | SHC1 | BTK | CCL20 | FURIN | PSMD2 | ADIPOR2 | TNFRSF11A | SCG2
9 genes	2.22E-15	F2RL1 | RFXAP | CCR6 | S100A10 | OXTR | CCL20 | FURIN | PSMD2 | TNFRSF11A
10 genes	2.22E-15	DKK1 | F2RL1 | FCGRT | CCR6 | SEMA3C | BTK | PSMC1 | CCL20 | FURIN | GPI
10 genes	2.22E-15	DKK1 | FCGRT | CCR6 | SHC1 | SEMA3C | FURIN | PSMD2 | ADIPOR2 | TNFRSF11A | SCG2
10 genes	2.22E-15	VEGFC | FCGRT | SEMA3C | OXTR | BTK | CCL20 | FURIN | TNFRSF11A | SCG2 | GPI
10 genes	2.22E-15	INSL4 | F2RL1 | CCR6 | SHC1 | BTK | PSMC1 | CCL20 | FURIN | TNFRSF11A | GPI
11 genes	2.22E-15	DKK1 | INSL4 | F2RL1 | RFXAP | CCR6 | PSMC1 | CCL20 | ADIPOR2 | TNFRSF11A | SCG2 | GPI
11 genes	2.22E-15	DKK1 | INSL4 | CCR6 | SEMA3C | OXTR | BTK | CCL20 | FURIN | TNFRSF11A | SCG2 | GPI
11 genes	2.22E-15	DKK1 | F2RL1 | CCR6 | S100A10 | SHC1 | BTK | PSMC1 | CCL20 | TNFRSF11A | SCG2 | GPI
11 genes	2.22E-15	DKK1 | FCGRT | CCR6 | SHC1 | SEMA3C | PSMC1 | FURIN | PSMD2 | ADIPOR2 | TNFRSF11A | SCG2
11 genes	2.22E-15	DKK1 | FCGRT | SHC1 | SEMA3C | BTK | CCL20 | FURIN | ADIPOR2 | TNFRSF11A | SCG2 | GPI
11 genes	2.22E-15	F2RL1 | CCR6 | OXTR | BTK | CCL20 | FURIN | PSMD2 | ADIPOR2 | TNFRSF11A | SCG2 | GPI
12 genes	2.22E-15	DKK1 | INSL4 | FCGRT | SHC1 | SEMA3C | BTK | CCL20 | FURIN | ADIPOR2 | TNFRSF11A | SCG2 | GPI

12 genes	2.22E-15	DKK1 | CCR6 | S100A10 | SHC1 | SEMA3C | OXTR | BTK | CCL20 | PSMD2 | ADIPOR2 | TNFRSF11A | SCG2
12 genes	2.22E-15	DKK1 | CCR6 | SHC1 | SEMA3C | OXTR | BTK | CCL20 | FURIN | PSMD2 | TNFRSF11A | SCG2 | GPI
12 genes	2.22E-15	F2RL1 | RFXAP | FCGRT | CCR6 | S100A10 | PSMC1 | CCL20 | PSMD2 | ADIPOR2 | TNFRSF11A | SCG2 | GPI
12 genes	2.22E-15	F2RL1 | RFXAP | FCGRT | S100A10 | SHC1 | BTK | CCL20 | FURIN | PSMD2 | ADIPOR2 | TNFRSF11A | SCG2
13 genes	2.22E-15	DKK1 | INSL4 | F2RL1 | RFXAP | CCR6 | S100A10 | SEMA3C | BTK | PSMC1 | CCL20 | PSMD2 | ADIPOR2 | GPI
13 genes	2.22E-15	DKK1 | INSL4 | FCGRT | SHC1 | SEMA3C | BTK | CCL20 | FURIN | PSMD2 | ADIPOR2 | TNFRSF11A | SCG2 | GPI
13 genes	2.22E-15	F2RL1 | RFXAP | FCGRT | CCR6 | S100A10 | OXTR | BTK | PSMC1 | CCL20 | FURIN | PSMD2 | SCG2 | GPI
14 genes	2.22E-15	DKK1 | INSL4 | FCGRT | SHC1 | SEMA3C | BTK | PSMC1 | CCL20 | FURIN | PSMD2 | ADIPOR2 | TNFRSF11A | SCG2 | GPI
16 genes	2.22E-15	DKK1 | F2RL1 | RFXAP | FCGRT | CCR6 | S100A10 | SHC1 | SEMA3C | OXTR | BTK | CCL20 | FURIN | PSMD2 | ADIPOR2 | TNFRSF11A | SCG2
8 genes	2.33E-15	F2RL1 | RFXAP | FCGRT | CCR6 | CCL20 | PSMD2 | ADIPOR2 | SCG2
9 genes	2.33E-15	VEGFC | F2RL1 | FCGRT | CCR6 | OXTR | BTK | CCL20 | FURIN | PSMD2
9 genes	2.33E-15	F2RL1 | RFXAP | CCR6 | S100A10 | CCL20 | FURIN | ADIPOR2 | TNFRSF11A | GPI
10 genes	2.33E-15	INSL4 | F2RL1 | RFXAP | FCGRT | CCR6 | CCL20 | PSMD2 | ADIPOR2 | SCG2 | GPI
10 genes	2.33E-15	INSL4 | F2RL1 | FCGRT | CCR6 | S100A10 | BTK | PSMC1 | CCL20 | FURIN | PSMD2
11 genes	2.33E-15	DKK1 | F2RL1 | CCR6 | S100A10 | SEMA3C | CCL20 | FURIN | PSMD2 | ADIPOR2 | TNFRSF11A | GPI
11 genes	2.33E-15	DKK1 | RFXAP | FCGRT | S100A10 | SEMA3C | OXTR | BTK | CCL20 | FURIN | ADIPOR2 | SCG2
11 genes	2.33E-15	DKK1 | CCR6 | S100A10 | SHC1 | SEMA3C | OXTR | BTK | CCL20 | TNFRSF11A | SCG2 | GPI
11 genes	2.33E-15	DKK1 | SHC1 | OXTR | BTK | CCL20 | FURIN | PSMD2 | ADIPOR2 | TNFRSF11A | SCG2 | GPI
11 genes	2.33E-15	F2RL1 | RFXAP | CCR6 | S100A10 | SHC1 | PSMC1 | CCL20 | FURIN | PSMD2 | SCG2 | GPI
11 genes	2.33E-15	F2RL1 | CCR6 | SEMA3C | OXTR | BTK | PSMC1 | CCL20 | FURIN | PSMD2 | ADIPOR2 | GPI
12 genes	2.33E-15	DKK1 | INSL4 | S100A10 | SHC1 | SEMA3C | OXTR | BTK | PSMC1 | CCL20 | TNFRSF11A | SCG2 | GPI
13 genes	2.33E-15	DKK1 | CCR6 | SHC1 | SEMA3C | OXTR | BTK | PSMC1 | CCL20 | FURIN | PSMD2 | ADIPOR2 | TNFRSF11A | SCG2
13 genes	2.33E-15	VEGFC | F2RL1 | RFXAP | FCGRT | CCR6 | S100A10 | OXTR | BTK | CCL20 | PSMD2 | ADIPOR2 | SCG2 | GPI
14 genes	2.33E-15	DKK1 | INSL4 | FCGRT | CCR6 | S100A10 | SHC1 | OXTR | PSMC1 | CCL20 | FURIN | ADIPOR2 | TNFRSF11A | SCG2 | GPI
14 genes	2.33E-15	DKK1 | F2RL1 | CCR6 | SHC1 | OXTR | BTK | PSMC1 | CCL20 | FURIN | PSMD2 | ADIPOR2 | TNFRSF11A | SCG2 | GPI
14 genes	2.33E-15	VEGFC | INSL4 | F2RL1 | RFXAP | FCGRT | CCR6 | S100A10 | OXTR | BTK | CCL20 | PSMD2 | ADIPOR2 | SCG2 | GPI
16 genes	2.33E-15	DKK1 | INSL4 | F2RL1 | FCGRT | CCR6 | SHC1 | OXTR | BTK | PSMC1 | CCL20 | FURIN | PSMD2 | ADIPOR2 | TNFRSF11A | SCG2 | GPI
8 genes	2.44E-15	F2RL1 | RFXAP | S100A10 | CCL20 | FURIN | PSMD2 | ADIPOR2 | GPI
9 genes	2.44E-15	DKK1 | F2RL1 | RFXAP | CCR6 | SEMA3C | PSMC1 | CCL20 | FURIN | GPI
9 genes	2.44E-15	F2RL1 | RFXAP | FCGRT | CCR6 | CCL20 | FURIN | PSMD2 | TNFRSF11A | SCG2
10 genes	2.44E-15	DKK1 | INSL4 | F2RL1 | CCR6 | PSMC1 | CCL20 | ADIPOR2 | TNFRSF11A | SCG2 | GPI
10 genes	2.44E-15	DKK1 | F2RL1 | RFXAP | FCGRT | CCR6 | SEMA3C | PSMC1 | CCL20 | FURIN | GPI

10 genes	2.44E-15	DKK1 | F2RL1 | CCR6 | OXTR | PSMC1 | CCL20 | ADIPOR2 | TNFRSF11A | SCG2 | GPI
10 genes	2.44E-15	DKK1 | FCGRT | CCR6 | SHC1 | PSMC1 | FURIN | PSMD2 | ADIPOR2 | TNFRSF11A | SCG2
10 genes	2.44E-15	VEGFC | F2RL1 | RFXAP | CCR6 | BTK | PSMC1 | CCL20 | PSMD2 | ADIPOR2 | SCG2
10 genes	2.44E-15	INSL4 | F2RL1 | RFXAP | CCR6 | PSMC1 | CCL20 | FURIN | ADIPOR2 | TNFRSF11A | GPI
10 genes	2.44E-15	F2RL1 | RFXAP | FCGRT | CCR6 | S100A10 | PSMC1 | CCL20 | PSMD2 | SCG2 | GPI
10 genes	2.44E-15	F2RL1 | RFXAP | FCGRT | CCR6 | CCL20 | FURIN | PSMD2 | ADIPOR2 | TNFRSF11A | SCG2
10 genes	2.44E-15	F2RL1 | RFXAP | CCR6 | PSMC1 | CCL20 | FURIN | PSMD2 | ADIPOR2 | SCG2 | GPI
11 genes	2.44E-15	DKK1 | INSL4 | F2RL1 | FCGRT | CCR6 | PSMC1 | CCL20 | ADIPOR2 | TNFRSF11A | SCG2 | GPI
11 genes	2.44E-15	DKK1 | INSL4 | F2RL1 | CCR6 | S100A10 | PSMC1 | CCL20 | FURIN | PSMD2 | TNFRSF11A | GPI
11 genes	2.44E-15	DKK1 | INSL4 | FCGRT | SHC1 | OXTR | BTK | CCL20 | PSMD2 | TNFRSF11A | SCG2 | GPI
11 genes	2.44E-15	DKK1 | INSL4 | CCR6 | S100A10 | SHC1 | OXTR | PSMC1 | CCL20 | FURIN | ADIPOR2 | SCG2
11 genes	2.44E-15	DKK1 | INSL4 | SHC1 | SEMA3C | OXTR | BTK | PSMC1 | CCL20 | TNFRSF11A | SCG2 | GPI
11 genes	2.44E-15	DKK1 | F2RL1 | RFXAP | CCR6 | S100A10 | BTK | PSMC1 | CCL20 | PSMD2 | ADIPOR2 | GPI
11 genes	2.44E-15	DKK1 | FCGRT | CCR6 | S100A10 | SEMA3C | BTK | CCL20 | ADIPOR2 | TNFRSF11A | SCG2 | GPI
11 genes	2.44E-15	DKK1 | S100A10 | SHC1 | SEMA3C | OXTR | BTK | CCL20 | FURIN | PSMD2 | TNFRSF11A | SCG2
11 genes	2.44E-15	VEGFC | F2RL1 | RFXAP | FCGRT | CCR6 | BTK | PSMC1 | CCL20 | PSMD2 | ADIPOR2 | SCG2
11 genes	2.44E-15	INSL4 | F2RL1 | RFXAP | FCGRT | CCR6 | SEMA3C | OXTR | PSMC1 | CCL20 | TNFRSF11A | GPI
11 genes	2.44E-15	F2RL1 | RFXAP | FCGRT | CCR6 | S100A10 | SHC1 | OXTR | CCL20 | FURIN | PSMD2 | SCG2
11 genes	2.44E-15	F2RL1 | RFXAP | CCR6 | S100A10 | OXTR | BTK | CCL20 | FURIN | PSMD2 | ADIPOR2 | SCG2
12 genes	2.44E-15	DKK1 | INSL4 | CCR6 | S100A10 | SHC1 | SEMA3C | OXTR | PSMC1 | CCL20 | FURIN | ADIPOR2 | SCG2
12 genes	2.44E-15	DKK1 | INSL4 | S100A10 | SHC1 | SEMA3C | BTK | CCL20 | FURIN | ADIPOR2 | TNFRSF11A | SCG2 | GPI
12 genes	2.44E-15	DKK1 | INSL4 | S100A10 | SEMA3C | OXTR | BTK | PSMC1 | CCL20 | FURIN | PSMD2 | ADIPOR2 | GPI
12 genes	2.44E-15	DKK1 | F2RL1 | RFXAP | FCGRT | CCR6 | SEMA3C | PSMC1 | CCL20 | ADIPOR2 | TNFRSF11A | SCG2 | GPI
12 genes	2.44E-15	DKK1 | F2RL1 | FCGRT | CCR6 | S100A10 | SEMA3C | BTK | CCL20 | FURIN | PSMD2 | SCG2 | GPI
12 genes	2.44E-15	F2RL1 | RFXAP | FCGRT | CCR6 | S100A10 | SHC1 | BTK | CCL20 | FURIN | PSMD2 | TNFRSF11A | SCG2
12 genes	2.44E-15	F2RL1 | RFXAP | FCGRT | CCR6 | S100A10 | OXTR | PSMC1 | CCL20 | FURIN | PSMD2 | SCG2 | GPI
12 genes	2.44E-15	F2RL1 | RFXAP | CCR6 | S100A10 | SHC1 | BTK | PSMC1 | CCL20 | FURIN | PSMD2 | SCG2 | GPI
13 genes	2.44E-15	DKK1 | INSL4 | F2RL1 | FCGRT | CCR6 | S100A10 | SEMA3C | BTK | CCL20 | FURIN | PSMD2 | SCG2 | GPI
13 genes	2.44E-15	DKK1 | INSL4 | CCR6 | S100A10 | SHC1 | SEMA3C | OXTR | CCL20 | FURIN | PSMD2 | TNFRSF11A | SCG2 | GPI
13 genes	2.44E-15	DKK1 | F2RL1 | RFXAP | FCGRT | CCR6 | S100A10 | OXTR | BTK | CCL20 | FURIN | ADIPOR2 | SCG2 | GPI
13 genes	2.44E-15	DKK1 | RFXAP | CCR6 | S100A10 | SHC1 | SEMA3C | OXTR | BTK | CCL20 | FURIN | PSMD2 | TNFRSF11A | SCG2
13 genes	2.44E-15	DKK1 | CCR6 | S100A10 | SHC1 | OXTR | BTK | PSMC1 | CCL20 | FURIN | PSMD2 | TNFRSF11A | SCG2 | GPI

13 genes	2.44E-15	DKK1 | CCR6 | S100A10 | SHC1 | OXTR | PSMC1 | CCL20 | FURIN | PSMD2 | ADIPOR2 | TNFRSF11A | SCG2 | GPI
13 genes	2.44E-15	VEGFC | INSL4 | F2RL1 | RFXAP | CCR6 | S100A10 | OXTR | CCL20 | FURIN | PSMD2 | ADIPOR2 | SCG2 | GPI
13 genes	2.44E-15	F2RL1 | RFXAP | FCGRT | CCR6 | S100A10 | SHC1 | BTK | CCL20 | FURIN | PSMD2 | ADIPOR2 | TNFRSF11A | SCG2
14 genes	2.44E-15	DKK1 | FCGRT | S100A10 | SHC1 | SEMA3C | OXTR | BTK | CCL20 | FURIN | PSMD2 | ADIPOR2 | TNFRSF11A | SCG2 | GPI
14 genes	2.44E-15	VEGFC | INSL4 | F2RL1 | RFXAP | FCGRT | CCR6 | S100A10 | SHC1 | OXTR | BTK | CCL20 | PSMD2 | ADIPOR2 | SCG2
15 genes	2.44E-15	DKK1 | F2RL1 | FCGRT | CCR6 | S100A10 | SHC1 | SEMA3C | BTK | PSMC1 | CCL20 | FURIN | PSMD2 | ADIPOR2 | SCG2 | GPI
15 genes	2.44E-15	DKK1 | FCGRT | CCR6 | S100A10 | SHC1 | SEMA3C | OXTR | PSMC1 | CCL20 | FURIN | PSMD2 | ADIPOR2 | TNFRSF11A | SCG2 | GPI
9 genes	2.55E-15	INSL4 | F2RL1 | RFXAP | S100A10 | BTK | CCL20 | FURIN | PSMD2 | ADIPOR2
9 genes	2.55E-15	CCR6 | S100A10 | SHC1 | SEMA3C | CCL20 | ADIPOR2 | TNFRSF11A | SCG2 | GPI
10 genes	2.55E-15	DKK1 | F2RL1 | RFXAP | CCR6 | PSMC1 | CCL20 | FURIN | PSMD2 | TNFRSF11A | GPI
10 genes	2.55E-15	DKK1 | FCGRT | S100A10 | SHC1 | SEMA3C | BTK | CCL20 | PSMD2 | ADIPOR2 | SCG2
10 genes	2.55E-15	DKK1 | SHC1 | OXTR | BTK | PSMC1 | CCL20 | PSMD2 | TNFRSF11A | SCG2 | GPI
10 genes	2.55E-15	INSL4 | F2RL1 | FCGRT | CCR6 | S100A10 | OXTR | BTK | CCL20 | FURIN | PSMD2
10 genes	2.55E-15	F2RL1 | RFXAP | FCGRT | CCR6 | S100A10 | PSMC1 | CCL20 | FURIN | PSMD2 | SCG2
10 genes	2.55E-15	F2RL1 | FCGRT | CCR6 | SHC1 | OXTR | PSMC1 | CCL20 | FURIN | PSMD2 | ADIPOR2
11 genes	2.55E-15	DKK1 | INSL4 | F2RL1 | RFXAP | FCGRT | CCR6 | PSMC1 | CCL20 | TNFRSF11A | SCG2 | GPI
11 genes	2.55E-15	DKK1 | INSL4 | FCGRT | S100A10 | SHC1 | SEMA3C | BTK | CCL20 | PSMD2 | ADIPOR2 | SCG2
11 genes	2.55E-15	DKK1 | INSL4 | S100A10 | SHC1 | OXTR | BTK | CCL20 | FURIN | PSMD2 | ADIPOR2 | SCG2
11 genes	2.55E-15	DKK1 | INSL4 | S100A10 | SEMA3C | OXTR | BTK | PSMC1 | CCL20 | FURIN | PSMD2 | GPI
11 genes	2.55E-15	DKK1 | F2RL1 | RFXAP | CCR6 | PSMC1 | CCL20 | FURIN | PSMD2 | ADIPOR2 | TNFRSF11A | GPI
11 genes	2.55E-15	VEGFC | CCR6 | OXTR | BTK | CCL20 | FURIN | PSMD2 | ADIPOR2 | TNFRSF11A | SCG2 | GPI
11 genes	2.55E-15	F2RL1 | RFXAP | FCGRT | CCR6 | S100A10 | BTK | CCL20 | FURIN | PSMD2 | ADIPOR2 | SCG2
11 genes	2.55E-15	F2RL1 | RFXAP | CCR6 | S100A10 | SEMA3C | PSMC1 | CCL20 | FURIN | PSMD2 | ADIPOR2 | GPI
11 genes	2.55E-15	F2RL1 | RFXAP | CCR6 | S100A10 | BTK | PSMC1 | CCL20 | FURIN | PSMD2 | ADIPOR2 | SCG2
11 genes	2.55E-15	F2RL1 | FCGRT | CCR6 | SHC1 | OXTR | BTK | PSMC1 | CCL20 | FURIN | PSMD2 | ADIPOR2
12 genes	2.55E-15	DKK1 | INSL4 | F2RL1 | RFXAP | FCGRT | CCR6 | SEMA3C | PSMC1 | CCL20 | TNFRSF11A | SCG2 | GPI
12 genes	2.55E-15	DKK1 | FCGRT | S100A10 | SHC1 | SEMA3C | OXTR | BTK | CCL20 | ADIPOR2 | TNFRSF11A | SCG2 | GPI
12 genes	2.55E-15	VEGFC | F2RL1 | RFXAP | CCR6 | S100A10 | SHC1 | OXTR | BTK | CCL20 | PSMD2 | ADIPOR2 | SCG2
12 genes	2.55E-15	VEGFC | F2RL1 | RFXAP | CCR6 | S100A10 | SEMA3C | OXTR | BTK | CCL20 | PSMD2 | SCG2 | GPI
12 genes	2.55E-15	VEGFC | FCGRT | CCR6 | OXTR | BTK | CCL20 | FURIN | PSMD2 | ADIPOR2 | TNFRSF11A | SCG2 | GPI
12 genes	2.55E-15	F2RL1 | RFXAP | FCGRT | CCR6 | BTK | PSMC1 | CCL20 | FURIN | PSMD2 | ADIPOR2 | SCG2 | GPI
13 genes	2.55E-15	DKK1 | INSL4 | S100A10 | SHC1 | OXTR | BTK | PSMC1 | CCL20 | FURIN | PSMD2 | ADIPOR2 | SCG2 | GPI

13 genes	2.55E-15	DKK1 | FCGRT | S100A10 | SHC1 | SEMA3C | OXTR | BTK | PSMC1 | CCL20 | FURIN | PSMD2 | TNFRSF11A | SCG2
13 genes	2.55E-15	DKK1 | FCGRT | S100A10 | SHC1 | SEMA3C | BTK | PSMC1 | CCL20 | PSMD2 | ADIPOR2 | TNFRSF11A | SCG2 | GPI
13 genes	2.55E-15	VEGFC | INSL4 | F2RL1 | RFXAP | FCGRT | CCR6 | S100A10 | OXTR | CCL20 | FURIN | PSMD2 | SCG2 | GPI
13 genes	2.55E-15	INSL4 | FCGRT | CCR6 | S100A10 | SHC1 | SEMA3C | BTK | CCL20 | FURIN | PSMD2 | ADIPOR2 | TNFRSF11A | SCG2
13 genes	2.55E-15	F2RL1 | RFXAP | FCGRT | CCR6 | S100A10 | SHC1 | SEMA3C | PSMC1 | CCL20 | FURIN | PSMD2 | SCG2 | GPI
14 genes	2.55E-15	DKK1 | INSL4 | F2RL1 | RFXAP | FCGRT | CCR6 | S100A10 | SHC1 | SEMA3C | OXTR | CCL20 | FURIN | PSMD2 | SCG2
14 genes	2.55E-15	DKK1 | F2RL1 | RFXAP | FCGRT | CCR6 | SHC1 | PSMC1 | CCL20 | FURIN | PSMD2 | ADIPOR2 | TNFRSF11A | SCG2 | GPI
14 genes	2.55E-15	DKK1 | F2RL1 | RFXAP | CCR6 | S100A10 | SHC1 | OXTR | BTK | CCL20 | FURIN | PSMD2 | ADIPOR2 | SCG2 | GPI
10 genes	2.66E-15	DKK1 | INSL4 | S100A10 | SHC1 | SEMA3C | BTK | CCL20 | TNFRSF11A | SCG2 | GPI
11 genes	2.66E-15	DKK1 | INSL4 | CCR6 | SEMA3C | OXTR | BTK | PSMC1 | CCL20 | TNFRSF11A | SCG2 | GPI
11 genes	2.66E-15	INSL4 | F2RL1 | RFXAP | FCGRT | CCR6 | BTK | CCL20 | FURIN | PSMD2 | ADIPOR2 | TNFRSF11A
11 genes	2.66E-15	INSL4 | F2RL1 | RFXAP | CCR6 | PSMC1 | CCL20 | FURIN | PSMD2 | ADIPOR2 | TNFRSF11A | GPI
11 genes	2.66E-15	F2RL1 | RFXAP | S100A10 | SHC1 | BTK | CCL20 | FURIN | PSMD2 | ADIPOR2 | TNFRSF11A | SCG2
11 genes	2.66E-15	F2RL1 | FCGRT | CCR6 | S100A10 | SHC1 | PSMC1 | CCL20 | FURIN | ADIPOR2 | TNFRSF11A | GPI
12 genes	2.66E-15	DKK1 | INSL4 | F2RL1 | RFXAP | FCGRT | CCR6 | PSMC1 | CCL20 | ADIPOR2 | TNFRSF11A | SCG2 | GPI
12 genes	2.66E-15	DKK1 | INSL4 | F2RL1 | CCR6 | SEMA3C | OXTR | BTK | PSMC1 | CCL20 | TNFRSF11A | SCG2 | GPI
12 genes	2.66E-15	DKK1 | INSL4 | FCGRT | SHC1 | SEMA3C | BTK | CCL20 | FURIN | PSMD2 | TNFRSF11A | SCG2 | GPI
12 genes	2.66E-15	DKK1 | INSL4 | CCR6 | SEMA3C | OXTR | BTK | PSMC1 | CCL20 | ADIPOR2 | TNFRSF11A | SCG2 | GPI
12 genes	2.66E-15	DKK1 | CCR6 | S100A10 | SHC1 | SEMA3C | OXTR | CCL20 | FURIN | PSMD2 | TNFRSF11A | SCG2 | GPI
12 genes	2.66E-15	F2RL1 | RFXAP | FCGRT | CCR6 | S100A10 | SEMA3C | PSMC1 | CCL20 | FURIN | PSMD2 | SCG2 | GPI
13 genes	2.66E-15	DKK1 | INSL4 | F2RL1 | RFXAP | FCGRT | CCR6 | SHC1 | PSMC1 | FURIN | PSMD2 | ADIPOR2 | TNFRSF11A | SCG2
13 genes	2.66E-15	DKK1 | INSL4 | F2RL1 | CCR6 | SEMA3C | OXTR | BTK | PSMC1 | CCL20 | ADIPOR2 | TNFRSF11A | SCG2 | GPI
13 genes	2.66E-15	DKK1 | INSL4 | CCR6 | S100A10 | SHC1 | SEMA3C | OXTR | BTK | PSMC1 | CCL20 | ADIPOR2 | TNFRSF11A | GPI
14 genes	2.66E-15	DKK1 | CCR6 | S100A10 | SHC1 | OXTR | BTK | PSMC1 | CCL20 | FURIN | PSMD2 | ADIPOR2 | TNFRSF11A | SCG2 | GPI
14 genes	2.66E-15	INSL4 | F2RL1 | RFXAP | FCGRT | CCR6 | SHC1 | OXTR | BTK | CCL20 | FURIN | PSMD2 | ADIPOR2 | TNFRSF11A | SCG2
15 genes	2.66E-15	DKK1 | INSL4 | CCR6 | S100A10 | SHC1 | OXTR | BTK | PSMC1 | CCL20 | FURIN | PSMD2 | ADIPOR2 | TNFRSF11A | SCG2 | GPI
9 genes	2.78E-15	DKK1 | F2RL1 | FCGRT | CCR6 | PSMC1 | CCL20 | FURIN | TNFRSF11A | GPI
9 genes	2.78E-15	F2RL1 | RFXAP | FCGRT | CCR6 | CCL20 | PSMD2 | ADIPOR2 | SCG2 | GPI
10 genes	2.78E-15	F2RL1 | RFXAP | CCR6 | SHC1 | PSMC1 | CCL20 | FURIN | PSMD2 | SCG2 | GPI
10 genes	2.78E-15	F2RL1 | RFXAP | CCR6 | OXTR | CCL20 | FURIN | ADIPOR2 | TNFRSF11A | SCG2 | GPI
11 genes	2.78E-15	DKK1 | CCR6 | S100A10 | SEMA3C | BTK | CCL20 | FURIN | PSMD2 | TNFRSF11A | SCG2 | GPI
11 genes	2.78E-15	F2RL1 | RFXAP | FCGRT | CCR6 | PSMC1 | CCL20 | FURIN | PSMD2 | ADIPOR2 | SCG2 | GPI

11 genes	2.78E-15	F2RL1 | RFXAP | FCGRT | S100A10 | SHC1 | SEMA3C | CCL20 | FURIN | PSMD2 | TNFRSF11A | SCG2
12 genes	2.78E-15	DKK1 | INSL4 | F2RL1 | RFXAP | CCR6 | S100A10 | PSMC1 | CCL20 | FURIN | PSMD2 | ADIPOR2 | GPI
12 genes	2.78E-15	DKK1 | INSL4 | CCR6 | SEMA3C | OXTR | BTK | CCL20 | FURIN | PSMD2 | TNFRSF11A | SCG2 | GPI
12 genes	2.78E-15	DKK1 | INSL4 | S100A10 | SHC1 | OXTR | BTK | CCL20 | FURIN | ADIPOR2 | TNFRSF11A | SCG2 | GPI
13 genes	2.78E-15	DKK1 | INSL4 | F2RL1 | RFXAP | FCGRT | CCR6 | S100A10 | PSMC1 | CCL20 | FURIN | PSMD2 | ADIPOR2 | GPI
13 genes	2.78E-15	DKK1 | INSL4 | CCR6 | S100A10 | SEMA3C | OXTR | BTK | CCL20 | FURIN | ADIPOR2 | TNFRSF11A | SCG2 | GPI
13 genes	2.78E-15	DKK1 | FCGRT | S100A10 | SHC1 | SEMA3C | OXTR | BTK | CCL20 | FURIN | PSMD2 | TNFRSF11A | SCG2 | GPI
13 genes	2.78E-15	INSL4 | F2RL1 | RFXAP | FCGRT | CCR6 | SHC1 | OXTR | BTK | CCL20 | PSMD2 | ADIPOR2 | TNFRSF11A | SCG2
14 genes	2.78E-15	DKK1 | INSL4 | CCR6 | S100A10 | SHC1 | SEMA3C | BTK | PSMC1 | CCL20 | FURIN | PSMD2 | TNFRSF11A | SCG2 | GPI
8 genes	2.89E-15	DKK1 | F2RL1 | CCR6 | PSMC1 | CCL20 | ADIPOR2 | TNFRSF11A | GPI
8 genes	2.89E-15	INSL4 | F2RL1 | RFXAP | CCR6 | SHC1 | OXTR | CCL20 | ADIPOR2
9 genes	2.89E-15	DKK1 | RFXAP | S100A10 | OXTR | BTK | CCL20 | FURIN | ADIPOR2 | SCG2
9 genes	2.89E-15	VEGFC | F2RL1 | CCR6 | SHC1 | OXTR | BTK | CCL20 | FURIN | PSMD2
10 genes	2.89E-15	DKK1 | S100A10 | SHC1 | OXTR | BTK | CCL20 | PSMD2 | TNFRSF11A | SCG2 | GPI
11 genes	2.89E-15	DKK1 | INSL4 | F2RL1 | RFXAP | FCGRT | CCR6 | SEMA3C | CCL20 | ADIPOR2 | TNFRSF11A | GPI
11 genes	2.89E-15	DKK1 | INSL4 | F2RL1 | CCR6 | S100A10 | SHC1 | SEMA3C | OXTR | CCL20 | FURIN | PSMD2
11 genes	2.89E-15	DKK1 | INSL4 | SEMA3C | OXTR | BTK | CCL20 | FURIN | PSMD2 | TNFRSF11A | SCG2 | GPI
11 genes	2.89E-15	DKK1 | CCR6 | S100A10 | SHC1 | SEMA3C | BTK | CCL20 | PSMD2 | TNFRSF11A | SCG2 | GPI
11 genes	2.89E-15	DKK1 | CCR6 | S100A10 | SEMA3C | OXTR | BTK | PSMC1 | CCL20 | TNFRSF11A | SCG2 | GPI
11 genes	2.89E-15	DKK1 | S100A10 | SHC1 | OXTR | BTK | CCL20 | FURIN | ADIPOR2 | TNFRSF11A | SCG2 | GPI
11 genes	2.89E-15	INSL4 | F2RL1 | RFXAP | FCGRT | CCR6 | S100A10 | PSMC1 | CCL20 | FURIN | PSMD2 | SCG2
12 genes	2.89E-15	DKK1 | INSL4 | CCR6 | SHC1 | SEMA3C | OXTR | BTK | CCL20 | FURIN | PSMD2 | TNFRSF11A | SCG2
12 genes	2.89E-15	DKK1 | F2RL1 | FCGRT | CCR6 | S100A10 | SHC1 | BTK | PSMC1 | CCL20 | TNFRSF11A | SCG2 | GPI
12 genes	2.89E-15	DKK1 | CCR6 | S100A10 | SHC1 | SEMA3C | BTK | PSMC1 | CCL20 | PSMD2 | TNFRSF11A | SCG2 | GPI
12 genes	2.89E-15	INSL4 | F2RL1 | RFXAP | CCR6 | SHC1 | PSMC1 | CCL20 | FURIN | PSMD2 | TNFRSF11A | SCG2 | GPI
13 genes	2.89E-15	DKK1 | VEGFC | RFXAP | SHC1 | SEMA3C | OXTR | BTK | CCL20 | FURIN | PSMD2 | TNFRSF11A | SCG2 | GPI
13 genes	2.89E-15	DKK1 | INSL4 | FCGRT | S100A10 | SHC1 | SEMA3C | BTK | CCL20 | PSMD2 | ADIPOR2 | TNFRSF11A | SCG2 | GPI
13 genes	2.89E-15	DKK1 | INSL4 | CCR6 | S100A10 | SHC1 | SEMA3C | OXTR | BTK | CCL20 | FURIN | PSMD2 | TNFRSF11A | SCG2
13 genes	2.89E-15	VEGFC | INSL4 | F2RL1 | RFXAP | CCR6 | S100A10 | OXTR | BTK | CCL20 | PSMD2 | ADIPOR2 | SCG2 | GPI
13 genes	2.89E-15	VEGFC | INSL4 | F2RL1 | FCGRT | CCR6 | S100A10 | OXTR | CCL20 | FURIN | PSMD2 | ADIPOR2 | SCG2 | GPI
14 genes	2.89E-15	DKK1 | INSL4 | FCGRT | CCR6 | S100A10 | SHC1 | SEMA3C | BTK | CCL20 | PSMD2 | ADIPOR2 | TNFRSF11A | SCG2 | GPI
15 genes	2.89E-15	DKK1 | INSL4 | CCR6 | S100A10 | SHC1 | SEMA3C | OXTR | BTK | PSMC1 | CCL20 | FURIN | PSMD2 | ADIPOR2 | TNFRSF11A | SCG2

15 genes	2.89E-15	DKK1 | FCGRT | CCR6 | S100A10 | SHC1 | SEMA3C | OXTR | BTK | PSMC1 | CCL20 | FURIN | PSMD2 | TNFRSF11A | SCG2 | GPI
15 genes	2.89E-15	VEGFC | INSL4 | RFXAP | FCGRT | CCR6 | S100A10 | SHC1 | SEMA3C | OXTR | BTK | CCL20 | FURIN | PSMD2 | TNFRSF11A | SCG2
16 genes	2.89E-15	VEGFC | INSL4 | RFXAP | FCGRT | CCR6 | S100A10 | SHC1 | SEMA3C | OXTR | BTK | CCL20 | FURIN | PSMD2 | ADIPOR2 | TNFRSF11A | SCG2
10 genes	3.00E-15	F2RL1 | RFXAP | FCGRT | CCR6 | S100A10 | BTK | CCL20 | PSMD2 | ADIPOR2 | SCG2
10 genes	3.00E-15	F2RL1 | CCR6 | BTK | PSMC1 | CCL20 | FURIN | PSMD2 | ADIPOR2 | SCG2 | GPI
11 genes	3.00E-15	DKK1 | INSL4 | FCGRT | S100A10 | BTK | CCL20 | FURIN | PSMD2 | ADIPOR2 | SCG2 | GPI
11 genes	3.00E-15	DKK1 | F2RL1 | RFXAP | CCR6 | SHC1 | PSMC1 | FURIN | PSMD2 | ADIPOR2 | TNFRSF11A | SCG2
11 genes	3.00E-15	DKK1 | F2RL1 | CCR6 | S100A10 | BTK | PSMC1 | CCL20 | ADIPOR2 | TNFRSF11A | SCG2 | GPI
11 genes	3.00E-15	DKK1 | F2RL1 | CCR6 | OXTR | PSMC1 | CCL20 | PSMD2 | ADIPOR2 | TNFRSF11A | SCG2 | GPI
11 genes	3.00E-15	DKK1 | FCGRT | CCR6 | S100A10 | SEMA3C | OXTR | BTK | CCL20 | PSMD2 | TNFRSF11A | SCG2
11 genes	3.00E-15	VEGFC | F2RL1 | RFXAP | CCR6 | OXTR | BTK | CCL20 | FURIN | PSMD2 | ADIPOR2 | GPI
12 genes	3.00E-15	DKK1 | VEGFC | RFXAP | SHC1 | SEMA3C | OXTR | BTK | CCL20 | PSMD2 | TNFRSF11A | SCG2 | GPI
12 genes	3.00E-15	DKK1 | INSL4 | F2RL1 | RFXAP | FCGRT | CCR6 | SEMA3C | PSMC1 | CCL20 | ADIPOR2 | TNFRSF11A | SCG2
12 genes	3.00E-15	DKK1 | INSL4 | FCGRT | CCR6 | S100A10 | SEMA3C | BTK | CCL20 | FURIN | PSMD2 | TNFRSF11A | SCG2
12 genes	3.00E-15	DKK1 | CCR6 | S100A10 | SHC1 | SEMA3C | BTK | PSMC1 | CCL20 | ADIPOR2 | TNFRSF11A | SCG2 | GPI
12 genes	3.00E-15	DKK1 | CCR6 | SHC1 | SEMA3C | OXTR | BTK | PSMC1 | CCL20 | FURIN | PSMD2 | TNFRSF11A | SCG2
12 genes	3.00E-15	DKK1 | CCR6 | SEMA3C | OXTR | BTK | PSMC1 | CCL20 | PSMD2 | ADIPOR2 | TNFRSF11A | SCG2 | GPI
12 genes	3.00E-15	DKK1 | S100A10 | SHC1 | SEMA3C | OXTR | BTK | PSMC1 | CCL20 | FURIN | PSMD2 | TNFRSF11A | SCG2
12 genes	3.00E-15	VEGFC | F2RL1 | RFXAP | FCGRT | CCR6 | OXTR | BTK | CCL20 | FURIN | PSMD2 | ADIPOR2 | GPI
12 genes	3.00E-15	F2RL1 | RFXAP | CCR6 | SHC1 | BTK | PSMC1 | CCL20 | FURIN | PSMD2 | ADIPOR2 | SCG2 | GPI
13 genes	3.00E-15	DKK1 | VEGFC | RFXAP | SHC1 | SEMA3C | OXTR | BTK | CCL20 | PSMD2 | ADIPOR2 | TNFRSF11A | SCG2 | GPI
14 genes	3.00E-15	DKK1 | INSL4 | CCR6 | S100A10 | SHC1 | SEMA3C | OXTR | CCL20 | FURIN | PSMD2 | ADIPOR2 | TNFRSF11A | SCG2 | GPI
15 genes	3.00E-15	DKK1 | FCGRT | CCR6 | S100A10 | SHC1 | SEMA3C | OXTR | BTK | PSMC1 | CCL20 | PSMD2 | ADIPOR2 | TNFRSF11A | SCG2 | GPI
10 genes	3.11E-15	DKK1 | INSL4 | F2RL1 | RFXAP | CCR6 | PSMC1 | CCL20 | TNFRSF11A | SCG2 | GPI
10 genes	3.11E-15	DKK1 | INSL4 | SHC1 | OXTR | BTK | CCL20 | PSMD2 | TNFRSF11A | SCG2 | GPI
10 genes	3.11E-15	DKK1 | F2RL1 | FCGRT | CCR6 | OXTR | PSMC1 | CCL20 | TNFRSF11A | SCG2 | GPI
11 genes	3.11E-15	DKK1 | CCR6 | S100A10 | SEMA3C | BTK | CCL20 | PSMD2 | ADIPOR2 | TNFRSF11A | SCG2 | GPI
11 genes	3.11E-15	F2RL1 | RFXAP | FCGRT | CCR6 | S100A10 | SHC1 | SEMA3C | OXTR | CCL20 | PSMD2 | SCG2
11 genes	3.11E-15	F2RL1 | FCGRT | CCR6 | SEMA3C | OXTR | BTK | CCL20 | FURIN | PSMD2 | ADIPOR2 | GPI
12 genes	3.11E-15	DKK1 | INSL4 | RFXAP | FCGRT | S100A10 | SEMA3C | OXTR | BTK | CCL20 | FURIN | ADIPOR2 | SCG2
12 genes	3.11E-15	DKK1 | INSL4 | CCR6 | SHC1 | SEMA3C | OXTR | BTK | CCL20 | ADIPOR2 | TNFRSF11A | SCG2 | GPI
12 genes	3.11E-15	DKK1 | F2RL1 | RFXAP | CCR6 | SHC1 | OXTR | CCL20 | FURIN | PSMD2 | ADIPOR2 | TNFRSF11A | SCG2

12 genes	3.11E-15	DKK1 | CCR6 | S100A10 | SHC1 | OXTR | BTK | PSMC1 | CCL20 | FURIN | PSMD2 | SCG2 | GPI
12 genes	3.11E-15	INSL4 | F2RL1 | RFXAP | CCR6 | SHC1 | OXTR | PSMC1 | FURIN | PSMD2 | ADIPOR2 | TNFRSF11A | SCG2
12 genes	3.11E-15	F2RL1 | RFXAP | FCGRT | CCR6 | SHC1 | PSMC1 | CCL20 | FURIN | PSMD2 | ADIPOR2 | SCG2 | GPI
13 genes	3.11E-15	DKK1 | VEGFC | CCR6 | S100A10 | SHC1 | OXTR | BTK | PSMC1 | CCL20 | PSMD2 | TNFRSF11A | SCG2 | GPI
13 genes	3.11E-15	DKK1 | FCGRT | CCR6 | S100A10 | SHC1 | OXTR | BTK | CCL20 | FURIN | PSMD2 | TNFRSF11A | SCG2 | GPI
13 genes	3.11E-15	DKK1 | FCGRT | CCR6 | S100A10 | SEMA3C | OXTR | BTK | CCL20 | FURIN | PSMD2 | TNFRSF11A | SCG2 | GPI
14 genes	3.11E-15	DKK1 | VEGFC | INSL4 | RFXAP | SHC1 | SEMA3C | OXTR | BTK | CCL20 | FURIN | PSMD2 | TNFRSF11A | SCG2 | GPI
14 genes	3.11E-15	DKK1 | INSL4 | CCR6 | S100A10 | SHC1 | SEMA3C | BTK | CCL20 | FURIN | PSMD2 | ADIPOR2 | TNFRSF11A | SCG2 | GPI
15 genes	3.11E-15	DKK1 | F2RL1 | RFXAP | FCGRT | CCR6 | S100A10 | SHC1 | SEMA3C | OXTR | BTK | CCL20 | FURIN | PSMD2 | TNFRSF11A | SCG2
16 genes	3.11E-15	DKK1 | INSL4 | FCGRT | CCR6 | S100A10 | SHC1 | SEMA3C | OXTR | BTK | PSMC1 | CCL20 | FURIN | PSMD2 | TNFRSF11A | SCG2 | GPI
9 genes	3.22E-15	DKK1 | F2RL1 | RFXAP | CCR6 | BTK | CCL20 | FURIN | ADIPOR2 | GPI
9 genes	3.22E-15	DKK1 | F2RL1 | CCR6 | PSMC1 | CCL20 | FURIN | PSMD2 | TNFRSF11A | GPI
9 genes	3.22E-15	F2RL1 | CCR6 | S100A10 | PSMC1 | CCL20 | FURIN | ADIPOR2 | TNFRSF11A | GPI
10 genes	3.22E-15	DKK1 | F2RL1 | CCR6 | SEMA3C | PSMC1 | CCL20 | FURIN | TNFRSF11A | SCG2 | GPI
10 genes	3.22E-15	DKK1 | RFXAP | FCGRT | S100A10 | SEMA3C | OXTR | BTK | CCL20 | ADIPOR2 | SCG2
10 genes	3.22E-15	INSL4 | F2RL1 | CCR6 | S100A10 | OXTR | BTK | CCL20 | FURIN | PSMD2 | GPI
10 genes	3.22E-15	F2RL1 | RFXAP | FCGRT | CCR6 | PSMC1 | CCL20 | FURIN | PSMD2 | SCG2 | GPI
10 genes	3.22E-15	F2RL1 | FCGRT | CCR6 | S100A10 | CCL20 | FURIN | PSMD2 | ADIPOR2 | TNFRSF11A | SCG2
11 genes	3.22E-15	DKK1 | INSL4 | F2RL1 | RFXAP | CCR6 | S100A10 | SEMA3C | CCL20 | FURIN | PSMD2 | ADIPOR2
11 genes	3.22E-15	DKK1 | INSL4 | F2RL1 | RFXAP | CCR6 | SEMA3C | PSMC1 | CCL20 | TNFRSF11A | SCG2 | GPI
11 genes	3.22E-15	DKK1 | CCR6 | S100A10 | SHC1 | OXTR | BTK | PSMC1 | CCL20 | PSMD2 | TNFRSF11A | SCG2
11 genes	3.22E-15	F2RL1 | FCGRT | CCR6 | SEMA3C | PSMC1 | CCL20 | FURIN | PSMD2 | ADIPOR2 | SCG2 | GPI
12 genes	3.22E-15	DKK1 | FCGRT | CCR6 | SEMA3C | BTK | PSMC1 | CCL20 | FURIN | PSMD2 | TNFRSF11A | SCG2 | GPI
12 genes	3.22E-15	DKK1 | CCR6 | S100A10 | SHC1 | OXTR | BTK | CCL20 | FURIN | PSMD2 | ADIPOR2 | TNFRSF11A | SCG2
13 genes	3.22E-15	DKK1 | INSL4 | FCGRT | CCR6 | S100A10 | SEMA3C | BTK | CCL20 | FURIN | PSMD2 | ADIPOR2 | TNFRSF11A | SCG2
13 genes	3.22E-15	DKK1 | CCR6 | S100A10 | SHC1 | SEMA3C | BTK | PSMC1 | CCL20 | FURIN | PSMD2 | TNFRSF11A | SCG2 | GPI
13 genes	3.22E-15	VEGFC | INSL4 | F2RL1 | RFXAP | CCR6 | S100A10 | SHC1 | OXTR | BTK | CCL20 | PSMD2 | ADIPOR2 | SCG2
13 genes	3.22E-15	VEGFC | INSL4 | FCGRT | CCR6 | SHC1 | SEMA3C | OXTR | BTK | PSMC1 | CCL20 | TNFRSF11A | SCG2 | GPI
14 genes	3.22E-15	DKK1 | FCGRT | CCR6 | S100A10 | SHC1 | SEMA3C | BTK | PSMC1 | CCL20 | PSMD2 | ADIPOR2 | TNFRSF11A | SCG2 | GPI
9 genes	3.33E-15	DKK1 | F2RL1 | CCR6 | OXTR | PSMC1 | CCL20 | ADIPOR2 | TNFRSF11A | GPI
9 genes	3.33E-15	DKK1 | F2RL1 | CCR6 | PSMC1 | CCL20 | ADIPOR2 | TNFRSF11A | SCG2 | GPI
9 genes	3.33E-15	DKK1 | CCR6 | S100A10 | BTK | CCL20 | PSMD2 | TNFRSF11A | SCG2 | GPI

9 genes	3.33E-15	VEGFC | F2RL1 | FCGRT | CCR6 | SHC1 | OXTR | CCL20 | FURIN | PSMD2
9 genes	3.33E-15	INSL4 | F2RL1 | RFXAP | FCGRT | CCR6 | CCL20 | PSMD2 | ADIPOR2 | SCG2
10 genes	3.33E-15	DKK1 | CCR6 | SEMA3C | BTK | CCL20 | FURIN | PSMD2 | TNFRSF11A | SCG2 | GPI
10 genes	3.33E-15	F2RL1 | RFXAP | FCGRT | S100A10 | PSMC1 | CCL20 | FURIN | PSMD2 | SCG2 | GPI
11 genes	3.33E-15	DKK1 | FCGRT | CCR6 | S100A10 | SEMA3C | BTK | PSMC1 | CCL20 | TNFRSF11A | SCG2 | GPI
12 genes	3.33E-15	DKK1 | FCGRT | CCR6 | S100A10 | SHC1 | OXTR | BTK | CCL20 | FURIN | PSMD2 | TNFRSF11A | SCG2
12 genes	3.33E-15	DKK1 | CCR6 | S100A10 | SHC1 | SEMA3C | BTK | CCL20 | FURIN | PSMD2 | TNFRSF11A | SCG2 | GPI
12 genes	3.33E-15	INSL4 | F2RL1 | RFXAP | FCGRT | CCR6 | SHC1 | OXTR | BTK | CCL20 | PSMD2 | TNFRSF11A | SCG2
13 genes	3.33E-15	DKK1 | INSL4 | SHC1 | SEMA3C | OXTR | BTK | PSMC1 | CCL20 | FURIN | ADIPOR2 | TNFRSF11A | SCG2 | GPI
13 genes	3.33E-15	DKK1 | F2RL1 | RFXAP | CCR6 | S100A10 | SHC1 | SEMA3C | OXTR | CCL20 | FURIN | PSMD2 | ADIPOR2 | SCG2
13 genes	3.33E-15	DKK1 | FCGRT | CCR6 | S100A10 | SHC1 | SEMA3C | BTK | CCL20 | PSMD2 | ADIPOR2 | TNFRSF11A | SCG2 | GPI
13 genes	3.33E-15	DKK1 | CCR6 | S100A10 | SHC1 | SEMA3C | BTK | CCL20 | FURIN | PSMD2 | ADIPOR2 | TNFRSF11A | SCG2 | GPI
13 genes	3.33E-15	F2RL1 | RFXAP | FCGRT | CCR6 | S100A10 | OXTR | BTK | CCL20 | FURIN | PSMD2 | ADIPOR2 | SCG2 | GPI
14 genes	3.33E-15	DKK1 | INSL4 | F2RL1 | RFXAP | CCR6 | S100A10 | SHC1 | SEMA3C | OXTR | CCL20 | FURIN | PSMD2 | ADIPOR2 | SCG2
14 genes	3.33E-15	DKK1 | FCGRT | CCR6 | S100A10 | SHC1 | SEMA3C | OXTR | BTK | PSMC1 | CCL20 | PSMD2 | TNFRSF11A | SCG2 | GPI
14 genes	3.33E-15	INSL4 | F2RL1 | RFXAP | FCGRT | CCR6 | S100A10 | SEMA3C | OXTR | CCL20 | FURIN | PSMD2 | ADIPOR2 | SCG2 | GPI
14 genes	3.33E-15	INSL4 | FCGRT | CCR6 | S100A10 | SHC1 | SEMA3C | BTK | PSMC1 | CCL20 | PSMD2 | ADIPOR2 | TNFRSF11A | SCG2 | GPI
15 genes	3.33E-15	VEGFC | F2RL1 | RFXAP | FCGRT | CCR6 | S100A10 | SEMA3C | OXTR | BTK | CCL20 | FURIN | PSMD2 | ADIPOR2 | SCG2 | GPI
10 genes	3.44E-15	DKK1 | INSL4 | F2RL1 | CCR6 | S100A10 | SHC1 | OXTR | CCL20 | FURIN | PSMD2
10 genes	3.44E-15	DKK1 | F2RL1 | RFXAP | CCR6 | S100A10 | SEMA3C | PSMC1 | CCL20 | FURIN | ADIPOR2
11 genes	3.44E-15	DKK1 | INSL4 | F2RL1 | RFXAP | FCGRT | CCR6 | BTK | CCL20 | FURIN | ADIPOR2 | GPI
11 genes	3.44E-15	DKK1 | INSL4 | F2RL1 | FCGRT | CCR6 | S100A10 | SHC1 | OXTR | CCL20 | FURIN | PSMD2
11 genes	3.44E-15	DKK1 | INSL4 | CCR6 | S100A10 | SHC1 | SEMA3C | BTK | CCL20 | TNFRSF11A | SCG2 | GPI
11 genes	3.44E-15	DKK1 | F2RL1 | FCGRT | CCR6 | S100A10 | SHC1 | SEMA3C | OXTR | CCL20 | FURIN | PSMD2
11 genes	3.44E-15	INSL4 | F2RL1 | RFXAP | FCGRT | CCR6 | SHC1 | BTK | CCL20 | PSMD2 | TNFRSF11A | SCG2
11 genes	3.44E-15	F2RL1 | CCR6 | SEMA3C | OXTR | BTK | PSMC1 | CCL20 | FURIN | PSMD2 | TNFRSF11A | GPI
12 genes	3.44E-15	DKK1 | INSL4 | CCR6 | S100A10 | SHC1 | OXTR | BTK | PSMC1 | CCL20 | PSMD2 | TNFRSF11A | SCG2
12 genes	3.44E-15	DKK1 | INSL4 | S100A10 | SHC1 | SEMA3C | BTK | CCL20 | PSMD2 | ADIPOR2 | TNFRSF11A | SCG2 | GPI
12 genes	3.44E-15	DKK1 | FCGRT | S100A10 | SHC1 | OXTR | BTK | PSMC1 | CCL20 | FURIN | PSMD2 | ADIPOR2 | SCG2
13 genes	3.44E-15	DKK1 | INSL4 | FCGRT | S100A10 | SHC1 | OXTR | BTK | PSMC1 | CCL20 | FURIN | PSMD2 | ADIPOR2 | SCG2
13 genes	3.44E-15	DKK1 | F2RL1 | FCGRT | CCR6 | SHC1 | SEMA3C | OXTR | CCL20 | FURIN | PSMD2 | ADIPOR2 | TNFRSF11A | SCG2
13 genes	3.44E-15	DKK1 | FCGRT | CCR6 | S100A10 | SHC1 | SEMA3C | OXTR | BTK | CCL20 | ADIPOR2 | TNFRSF11A | SCG2 | GPI

13 genes	3.44E-15	DKK1 | FCGRT | S100A10 | SHC1 | SEMA3C | OXTR | BTK | CCL20 | FURIN | PSMD2 | ADIPOR2 | TNFRSF11A | SCG2
13 genes	3.44E-15	F2RL1 | RFXAP | FCGRT | S100A10 | SHC1 | SEMA3C | PSMC1 | CCL20 | FURIN | PSMD2 | ADIPOR2 | TNFRSF11A | SCG2
14 genes	3.44E-15	VEGFC | INSL4 | RFXAP | FCGRT | CCR6 | S100A10 | SHC1 | SEMA3C | OXTR | BTK | CCL20 | PSMD2 | TNFRSF11A | SCG2
14 genes	3.44E-15	VEGFC | INSL4 | CCR6 | SHC1 | SEMA3C | OXTR | BTK | PSMC1 | CCL20 | FURIN | ADIPOR2 | TNFRSF11A | SCG2 | GPI
9 genes	3.55E-15	INSL4 | F2RL1 | CCR6 | SHC1 | OXTR | CCL20 | FURIN | PSMD2 | TNFRSF11A
9 genes	3.55E-15	F2RL1 | RFXAP | FCGRT | CCR6 | CCL20 | FURIN | PSMD2 | ADIPOR2 | SCG2
10 genes	3.55E-15	DKK1 | F2RL1 | CCR6 | S100A10 | BTK | PSMC1 | CCL20 | TNFRSF11A | SCG2 | GPI
11 genes	3.55E-15	DKK1 | INSL4 | CCR6 | SHC1 | OXTR | BTK | CCL20 | PSMD2 | TNFRSF11A | SCG2 | GPI
11 genes	3.55E-15	DKK1 | INSL4 | CCR6 | SEMA3C | BTK | CCL20 | FURIN | PSMD2 | TNFRSF11A | SCG2 | GPI
11 genes	3.55E-15	DKK1 | INSL4 | S100A10 | SHC1 | OXTR | BTK | PSMC1 | CCL20 | FURIN | PSMD2 | SCG2
12 genes	3.55E-15	DKK1 | INSL4 | F2RL1 | RFXAP | CCR6 | S100A10 | BTK | PSMC1 | CCL20 | PSMD2 | ADIPOR2 | GPI
12 genes	3.55E-15	DKK1 | INSL4 | CCR6 | S100A10 | SHC1 | OXTR | BTK | CCL20 | PSMD2 | TNFRSF11A | SCG2 | GPI
13 genes	3.55E-15	DKK1 | INSL4 | FCGRT | CCR6 | S100A10 | SHC1 | OXTR | BTK | CCL20 | PSMD2 | ADIPOR2 | TNFRSF11A | SCG2
13 genes	3.55E-15	DKK1 | FCGRT | S100A10 | SHC1 | SEMA3C | OXTR | BTK | PSMC1 | CCL20 | PSMD2 | TNFRSF11A | SCG2 | GPI
13 genes	3.55E-15	VEGFC | INSL4 | F2RL1 | RFXAP | CCR6 | S100A10 | OXTR | BTK | CCL20 | FURIN | PSMD2 | ADIPOR2 | SCG2
7 genes	3.66E-15	F2RL1 | RFXAP | FCGRT | CCR6 | S100A10 | CCL20 | FURIN
9 genes	3.66E-15	INSL4 | F2RL1 | CCR6 | SEMA3C | PSMC1 | CCL20 | FURIN | PSMD2 | GPI
10 genes	3.66E-15	INSL4 | F2RL1 | RFXAP | FCGRT | CCR6 | S100A10 | OXTR | CCL20 | FURIN | PSMD2
10 genes	3.66E-15	CCR6 | S100A10 | SHC1 | SEMA3C | CCL20 | FURIN | ADIPOR2 | TNFRSF11A | SCG2 | GPI
11 genes	3.66E-15	DKK1 | INSL4 | S100A10 | SHC1 | SEMA3C | BTK | CCL20 | PSMD2 | TNFRSF11A | SCG2 | GPI
11 genes	3.66E-15	DKK1 | CCR6 | SHC1 | SEMA3C | OXTR | BTK | CCL20 | ADIPOR2 | TNFRSF11A | SCG2 | GPI
11 genes	3.66E-15	VEGFC | F2RL1 | RFXAP | CCR6 | S100A10 | OXTR | CCL20 | FURIN | PSMD2 | SCG2 | GPI
11 genes	3.66E-15	INSL4 | F2RL1 | RFXAP | FCGRT | CCR6 | S100A10 | BTK | CCL20 | FURIN | PSMD2 | ADIPOR2
11 genes	3.66E-15	FCGRT | CCR6 | SHC1 | SEMA3C | PSMC1 | CCL20 | FURIN | ADIPOR2 | TNFRSF11A | SCG2 | GPI
12 genes	3.66E-15	DKK1 | FCGRT | CCR6 | S100A10 | SEMA3C | BTK | CCL20 | FURIN | PSMD2 | ADIPOR2 | TNFRSF11A | SCG2
12 genes	3.66E-15	DKK1 | FCGRT | CCR6 | SHC1 | OXTR | BTK | CCL20 | FURIN | PSMD2 | TNFRSF11A | SCG2 | GPI
12 genes	3.66E-15	DKK1 | S100A10 | SHC1 | OXTR | BTK | CCL20 | FURIN | PSMD2 | ADIPOR2 | TNFRSF11A | SCG2 | GPI
12 genes	3.66E-15	VEGFC | INSL4 | F2RL1 | RFXAP | FCGRT | CCR6 | BTK | PSMC1 | CCL20 | FURIN | PSMD2 | ADIPOR2
12 genes	3.66E-15	F2RL1 | RFXAP | CCR6 | S100A10 | SEMA3C | PSMC1 | CCL20 | FURIN | PSMD2 | ADIPOR2 | SCG2 | GPI
12 genes	3.66E-15	F2RL1 | CCR6 | SHC1 | OXTR | BTK | PSMC1 | CCL20 | FURIN | PSMD2 | ADIPOR2 | TNFRSF11A | SCG2
13 genes	3.66E-15	DKK1 | INSL4 | FCGRT | CCR6 | SEMA3C | OXTR | CCL20 | FURIN | PSMD2 | ADIPOR2 | TNFRSF11A | SCG2 | GPI
13 genes	3.66E-15	VEGFC | CCR6 | SHC1 | SEMA3C | OXTR | BTK | PSMC1 | CCL20 | FURIN | PSMD2 | TNFRSF11A | SCG2 | GPI

14 genes	3.66E-15	DKK1 | FCGRT | CCR6 | S100A10 | SHC1 | OXTR | PSMC1 | CCL20 | FURIN | PSMD2 | ADIPOR2 | TNFRSF11A | SCG2 | GPI
14 genes	3.66E-15	VEGFC | CCR6 | SHC1 | SEMA3C | OXTR | BTK | PSMC1 | CCL20 | FURIN | PSMD2 | ADIPOR2 | TNFRSF11A | SCG2 | GPI
15 genes	3.66E-15	DKK1 | F2RL1 | RFXAP | FCGRT | CCR6 | SHC1 | SEMA3C | OXTR | PSMC1 | CCL20 | FURIN | PSMD2 | ADIPOR2 | SCG2 | GPI
16 genes	3.66E-15	DKK1 | INSL4 | FCGRT | CCR6 | S100A10 | SHC1 | SEMA3C | OXTR | BTK | PSMC1 | CCL20 | PSMD2 | ADIPOR2 | TNFRSF11A | SCG2 | GPI
9 genes	3.77E-15	DKK1 | F2RL1 | RFXAP | CCR6 | PSMC1 | CCL20 | FURIN | ADIPOR2 | SCG2
10 genes	3.77E-15	DKK1 | INSL4 | F2RL1 | RFXAP | CCR6 | PSMC1 | CCL20 | FURIN | ADIPOR2 | SCG2
10 genes	3.77E-15	DKK1 | INSL4 | F2RL1 | CCR6 | SEMA3C | CCL20 | ADIPOR2 | TNFRSF11A | SCG2 | GPI
10 genes	3.77E-15	DKK1 | INSL4 | CCR6 | SHC1 | OXTR | BTK | PSMC1 | CCL20 | FURIN | PSMD2
10 genes	3.77E-15	DKK1 | INSL4 | S100A10 | SHC1 | OXTR | BTK | CCL20 | PSMD2 | ADIPOR2 | SCG2
10 genes	3.77E-15	DKK1 | RFXAP | CCR6 | S100A10 | SEMA3C | BTK | CCL20 | TNFRSF11A | SCG2 | GPI
10 genes	3.77E-15	DKK1 | FCGRT | S100A10 | SHC1 | OXTR | BTK | CCL20 | PSMD2 | ADIPOR2 | SCG2
10 genes	3.77E-15	DKK1 | FCGRT | S100A10 | SEMA3C | OXTR | BTK | CCL20 | FURIN | PSMD2 | GPI
10 genes	3.77E-15	INSL4 | F2RL1 | CCR6 | SHC1 | OXTR | PSMC1 | CCL20 | PSMD2 | ADIPOR2 | SCG2
10 genes	3.77E-15	F2RL1 | RFXAP | CCR6 | BTK | PSMC1 | CCL20 | FURIN | PSMD2 | SCG2 | GPI
10 genes	3.77E-15	CCR6 | S100A10 | SHC1 | SEMA3C | OXTR | BTK | PSMC1 | FURIN | TNFRSF11A | SCG2
11 genes	3.77E-15	DKK1 | INSL4 | F2RL1 | CCR6 | SEMA3C | PSMC1 | CCL20 | FURIN | TNFRSF11A | SCG2 | GPI
11 genes	3.77E-15	DKK1 | INSL4 | S100A10 | SHC1 | OXTR | BTK | CCL20 | PSMD2 | ADIPOR2 | TNFRSF11A | SCG2
11 genes	3.77E-15	DKK1 | F2RL1 | FCGRT | CCR6 | SEMA3C | OXTR | PSMC1 | CCL20 | TNFRSF11A | SCG2 | GPI
11 genes	3.77E-15	DKK1 | FCGRT | S100A10 | SHC1 | OXTR | BTK | PSMC1 | CCL20 | TNFRSF11A | SCG2 | GPI
11 genes	3.77E-15	DKK1 | CCR6 | SEMA3C | OXTR | BTK | CCL20 | FURIN | PSMD2 | TNFRSF11A | SCG2 | GPI
11 genes	3.77E-15	DKK1 | S100A10 | SHC1 | SEMA3C | BTK | PSMC1 | CCL20 | ADIPOR2 | TNFRSF11A | SCG2 | GPI
11 genes	3.77E-15	F2RL1 | RFXAP | FCGRT | CCR6 | S100A10 | OXTR | BTK | PSMC1 | CCL20 | FURIN | PSMD2
12 genes	3.77E-15	DKK1 | INSL4 | F2RL1 | RFXAP | FCGRT | CCR6 | OXTR | PSMC1 | CCL20 | FURIN | PSMD2 | SCG2
12 genes	3.77E-15	DKK1 | INSL4 | F2RL1 | FCGRT | CCR6 | SEMA3C | PSMC1 | CCL20 | FURIN | TNFRSF11A | SCG2 | GPI
12 genes	3.77E-15	DKK1 | INSL4 | FCGRT | CCR6 | S100A10 | SEMA3C | BTK | CCL20 | FURIN | PSMD2 | SCG2 | GPI
12 genes	3.77E-15	DKK1 | INSL4 | CCR6 | SEMA3C | OXTR | CCL20 | FURIN | PSMD2 | ADIPOR2 | TNFRSF11A | SCG2 | GPI
12 genes	3.77E-15	DKK1 | INSL4 | S100A10 | SHC1 | SEMA3C | BTK | PSMC1 | CCL20 | ADIPOR2 | TNFRSF11A | SCG2 | GPI
12 genes	3.77E-15	DKK1 | F2RL1 | RFXAP | FCGRT | CCR6 | SEMA3C | BTK | PSMC1 | CCL20 | FURIN | ADIPOR2 | GPI
12 genes	3.77E-15	DKK1 | CCR6 | S100A10 | SEMA3C | OXTR | BTK | CCL20 | FURIN | ADIPOR2 | TNFRSF11A | SCG2 | GPI
12 genes	3.77E-15	DKK1 | CCR6 | SHC1 | SEMA3C | OXTR | BTK | CCL20 | FURIN | ADIPOR2 | TNFRSF11A | SCG2 | GPI
12 genes	3.77E-15	DKK1 | S100A10 | SHC1 | SEMA3C | OXTR | BTK | CCL20 | FURIN | PSMD2 | ADIPOR2 | TNFRSF11A | SCG2
13 genes	3.77E-15	DKK1 | INSL4 | F2RL1 | RFXAP | FCGRT | CCR6 | OXTR | PSMC1 | CCL20 | FURIN | PSMD2 | ADIPOR2 | SCG2

13 genes	3.77E-15	DKK1 | INSL4 | CCR6 | SHC1 | SEMA3C | OXTR | BTK | CCL20 | FURIN | ADIPOR2 | TNFRSF11A | SCG2 | GPI
13 genes	3.77E-15	DKK1 | F2RL1 | RFXAP | CCR6 | SHC1 | BTK | PSMC1 | CCL20 | FURIN | ADIPOR2 | TNFRSF11A | SCG2 | GPI
13 genes	3.77E-15	DKK1 | S100A10 | SHC1 | SEMA3C | OXTR | BTK | PSMC1 | CCL20 | FURIN | PSMD2 | ADIPOR2 | TNFRSF11A | SCG2
14 genes	3.77E-15	DKK1 | VEGFC | F2RL1 | RFXAP | FCGRT | CCR6 | SHC1 | OXTR | BTK | CCL20 | PSMD2 | ADIPOR2 | SCG2 | GPI
15 genes	3.77E-15	DKK1 | INSL4 | RFXAP | CCR6 | S100A10 | SHC1 | SEMA3C | OXTR | BTK | CCL20 | FURIN | PSMD2 | TNFRSF11A | SCG2 | GPI
15 genes	3.77E-15	DKK1 | INSL4 | FCGRT | CCR6 | S100A10 | SHC1 | SEMA3C | OXTR | BTK | PSMC1 | CCL20 | FURIN | PSMD2 | SCG2 | GPI
16 genes	3.77E-15	DKK1 | INSL4 | RFXAP | CCR6 | S100A10 | SHC1 | SEMA3C | OXTR | BTK | CCL20 | FURIN | PSMD2 | ADIPOR2 | TNFRSF11A | SCG2 | GPI
8 genes	3.89E-15	INSL4 | F2RL1 | CCR6 | CCL20 | FURIN | TNFRSF11A | SCG2 | GPI
10 genes	3.89E-15	VEGFC | F2RL1 | RFXAP | CCR6 | SHC1 | OXTR | BTK | CCL20 | FURIN | PSMD2
11 genes	3.89E-15	DKK1 | INSL4 | F2RL1 | FCGRT | CCR6 | SEMA3C | CCL20 | ADIPOR2 | TNFRSF11A | SCG2 | GPI
11 genes	3.89E-15	VEGFC | F2RL1 | FCGRT | CCR6 | SHC1 | OXTR | BTK | CCL20 | FURIN | PSMD2 | ADIPOR2
11 genes	3.89E-15	INSL4 | CCR6 | S100A10 | SHC1 | SEMA3C | BTK | CCL20 | ADIPOR2 | TNFRSF11A | SCG2 | GPI
11 genes	3.89E-15	F2RL1 | RFXAP | CCR6 | BTK | PSMC1 | CCL20 | FURIN | PSMD2 | ADIPOR2 | SCG2 | GPI
12 genes	3.89E-15	DKK1 | INSL4 | CCR6 | S100A10 | SHC1 | BTK | CCL20 | FURIN | PSMD2 | TNFRSF11A | SCG2 | GPI
12 genes	3.89E-15	DKK1 | F2RL1 | CCR6 | S100A10 | SHC1 | OXTR | CCL20 | FURIN | PSMD2 | TNFRSF11A | SCG2 | GPI
12 genes	3.89E-15	DKK1 | FCGRT | CCR6 | S100A10 | SHC1 | OXTR | BTK | CCL20 | PSMD2 | ADIPOR2 | TNFRSF11A | SCG2
12 genes	3.89E-15	INSL4 | F2RL1 | RFXAP | FCGRT | CCR6 | S100A10 | PSMC1 | CCL20 | FURIN | PSMD2 | ADIPOR2 | SCG2
12 genes	3.89E-15	INSL4 | F2RL1 | CCR6 | SHC1 | OXTR | BTK | PSMC1 | CCL20 | FURIN | PSMD2 | ADIPOR2 | SCG2
12 genes	3.89E-15	INSL4 | FCGRT | CCR6 | S100A10 | SHC1 | SEMA3C | BTK | CCL20 | FURIN | PSMD2 | TNFRSF11A | SCG2
13 genes	3.89E-15	INSL4 | F2RL1 | RFXAP | CCR6 | S100A10 | SHC1 | SEMA3C | BTK | PSMC1 | CCL20 | PSMD2 | ADIPOR2 | SCG2
13 genes	3.89E-15	INSL4 | FCGRT | CCR6 | S100A10 | SHC1 | SEMA3C | BTK | CCL20 | FURIN | ADIPOR2 | TNFRSF11A | SCG2 | GPI
14 genes	3.89E-15	DKK1 | INSL4 | CCR6 | S100A10 | SHC1 | OXTR | BTK | PSMC1 | CCL20 | FURIN | PSMD2 | ADIPOR2 | SCG2 | GPI
14 genes	3.89E-15	INSL4 | F2RL1 | RFXAP | FCGRT | CCR6 | S100A10 | SHC1 | SEMA3C | BTK | PSMC1 | CCL20 | PSMD2 | ADIPOR2 | SCG2
14 genes	3.89E-15	INSL4 | F2RL1 | RFXAP | FCGRT | CCR6 | SEMA3C | OXTR | BTK | PSMC1 | CCL20 | FURIN | PSMD2 | ADIPOR2 | SCG2
15 genes	3.89E-15	DKK1 | INSL4 | RFXAP | FCGRT | CCR6 | S100A10 | SHC1 | OXTR | BTK | CCL20 | FURIN | PSMD2 | ADIPOR2 | SCG2 | GPI
10 genes	4.00E-15	DKK1 | INSL4 | S100A10 | SHC1 | BTK | CCL20 | ADIPOR2 | TNFRSF11A | SCG2 | GPI
10 genes	4.00E-15	DKK1 | F2RL1 | RFXAP | CCR6 | SEMA3C | PSMC1 | CCL20 | ADIPOR2 | TNFRSF11A | SCG2
10 genes	4.00E-15	DKK1 | F2RL1 | FCGRT | CCR6 | S100A10 | SHC1 | OXTR | CCL20 | FURIN | PSMD2
10 genes	4.00E-15	DKK1 | F2RL1 | CCR6 | S100A10 | SHC1 | SEMA3C | OXTR | CCL20 | FURIN | PSMD2
10 genes	4.00E-15	DKK1 | CCR6 | S100A10 | SHC1 | SEMA3C | BTK | CCL20 | TNFRSF11A | SCG2 | GPI
10 genes	4.00E-15	INSL4 | F2RL1 | CCR6 | S100A10 | SHC1 | OXTR | CCL20 | FURIN | PSMD2 | TNFRSF11A
10 genes	4.00E-15	INSL4 | F2RL1 | CCR6 | SHC1 | OXTR | BTK | CCL20 | FURIN | PSMD2 | ADIPOR2

11 genes	4.00E-15	DKK1 | INSL4 | F2RL1 | CCR6 | SHC1 | SEMA3C | FURIN | PSMD2 | ADIPOR2 | TNFRSF11A | SCG2
11 genes	4.00E-15	DKK1 | CCR6 | SHC1 | SEMA3C | OXTR | PSMC1 | FURIN | PSMD2 | ADIPOR2 | TNFRSF11A | SCG2
11 genes	4.00E-15	VEGFC | F2RL1 | FCGRT | CCR6 | SHC1 | OXTR | CCL20 | FURIN | PSMD2 | SCG2 | GPI
11 genes	4.00E-15	INSL4 | F2RL1 | RFXAP | FCGRT | CCR6 | BTK | CCL20 | PSMD2 | ADIPOR2 | TNFRSF11A | SCG2
12 genes	4.00E-15	DKK1 | INSL4 | F2RL1 | FCGRT | CCR6 | S100A10 | SHC1 | SEMA3C | OXTR | CCL20 | FURIN | PSMD2
12 genes	4.00E-15	DKK1 | FCGRT | CCR6 | S100A10 | SHC1 | SEMA3C | OXTR | PSMC1 | CCL20 | TNFRSF11A | SCG2 | GPI
12 genes	4.00E-15	DKK1 | CCR6 | S100A10 | SHC1 | SEMA3C | OXTR | BTK | CCL20 | FURIN | TNFRSF11A | SCG2 | GPI
12 genes	4.00E-15	DKK1 | S100A10 | SHC1 | SEMA3C | OXTR | BTK | PSMC1 | CCL20 | FURIN | TNFRSF11A | SCG2 | GPI
12 genes	4.00E-15	INSL4 | F2RL1 | RFXAP | CCR6 | S100A10 | SHC1 | PSMC1 | CCL20 | FURIN | PSMD2 | ADIPOR2 | SCG2
13 genes	4.00E-15	DKK1 | INSL4 | CCR6 | S100A10 | SHC1 | SEMA3C | OXTR | BTK | CCL20 | FURIN | TNFRSF11A | SCG2 | GPI
13 genes	4.00E-15	DKK1 | INSL4 | S100A10 | SHC1 | SEMA3C | OXTR | BTK | PSMC1 | CCL20 | FURIN | TNFRSF11A | SCG2 | GPI
13 genes	4.00E-15	DKK1 | RFXAP | FCGRT | CCR6 | S100A10 | SHC1 | OXTR | BTK | CCL20 | FURIN | PSMD2 | SCG2 | GPI
13 genes	4.00E-15	DKK1 | CCR6 | S100A10 | SHC1 | SEMA3C | OXTR | BTK | CCL20 | FURIN | PSMD2 | ADIPOR2 | TNFRSF11A | SCG2
13 genes	4.00E-15	VEGFC | CCR6 | SHC1 | SEMA3C | OXTR | BTK | PSMC1 | CCL20 | FURIN | ADIPOR2 | TNFRSF11A | SCG2 | GPI
14 genes	4.00E-15	DKK1 | RFXAP | CCR6 | S100A10 | SHC1 | SEMA3C | OXTR | BTK | CCL20 | FURIN | PSMD2 | TNFRSF11A | SCG2 | GPI
14 genes	4.00E-15	VEGFC | INSL4 | CCR6 | SHC1 | SEMA3C | OXTR | BTK | CCL20 | FURIN | PSMD2 | ADIPOR2 | TNFRSF11A | SCG2 | GPI
15 genes	4.00E-15	DKK1 | INSL4 | FCGRT | CCR6 | S100A10 | SHC1 | SEMA3C | BTK | PSMC1 | CCL20 | PSMD2 | ADIPOR2 | TNFRSF11A | SCG2 | GPI
15 genes	4.00E-15	DKK1 | INSL4 | CCR6 | S100A10 | SHC1 | SEMA3C | OXTR | BTK | PSMC1 | CCL20 | FURIN | PSMD2 | ADIPOR2 | SCG2 | GPI
15 genes	4.00E-15	DKK1 | RFXAP | CCR6 | S100A10 | SHC1 | SEMA3C | OXTR | BTK | CCL20 | FURIN | PSMD2 | ADIPOR2 | TNFRSF11A | SCG2 | GPI
8 genes	4.11E-15	INSL4 | F2RL1 | CCR6 | SHC1 | CCL20 | PSMD2 | TNFRSF11A | SCG2
9 genes	4.11E-15	DKK1 | CCR6 | SHC1 | SEMA3C | FURIN | PSMD2 | ADIPOR2 | TNFRSF11A | SCG2
10 genes	4.11E-15	DKK1 | F2RL1 | RFXAP | CCR6 | PSMC1 | CCL20 | FURIN | ADIPOR2 | TNFRSF11A | SCG2
10 genes	4.11E-15	DKK1 | F2RL1 | FCGRT | CCR6 | PSMC1 | CCL20 | ADIPOR2 | TNFRSF11A | SCG2 | GPI
10 genes	4.11E-15	DKK1 | F2RL1 | CCR6 | SEMA3C | OXTR | PSMC1 | CCL20 | ADIPOR2 | TNFRSF11A | GPI
10 genes	4.11E-15	DKK1 | S100A10 | SHC1 | OXTR | BTK | PSMC1 | CCL20 | TNFRSF11A | SCG2 | GPI
11 genes	4.11E-15	DKK1 | F2RL1 | CCR6 | SHC1 | SEMA3C | PSMC1 | FURIN | PSMD2 | ADIPOR2 | TNFRSF11A | SCG2
11 genes	4.11E-15	INSL4 | F2RL1 | RFXAP | FCGRT | S100A10 | OXTR | BTK | CCL20 | FURIN | PSMD2 | ADIPOR2
11 genes	4.11E-15	INSL4 | F2RL1 | RFXAP | CCR6 | SHC1 | SEMA3C | OXTR | BTK | CCL20 | PSMD2 | TNFRSF11A
11 genes	4.11E-15	INSL4 | F2RL1 | CCR6 | S100A10 | SHC1 | PSMC1 | CCL20 | FURIN | PSMD2 | ADIPOR2 | SCG2
12 genes	4.11E-15	DKK1 | INSL4 | S100A10 | SHC1 | OXTR | BTK | CCL20 | PSMD2 | ADIPOR2 | TNFRSF11A | SCG2 | GPI
12 genes	4.11E-15	F2RL1 | RFXAP | S100A10 | SHC1 | SEMA3C | OXTR | PSMC1 | CCL20 | FURIN | PSMD2 | TNFRSF11A | SCG2
13 genes	4.11E-15	DKK1 | INSL4 | FCGRT | SHC1 | SEMA3C | OXTR | BTK | CCL20 | FURIN | ADIPOR2 | TNFRSF11A | SCG2 | GPI

13 genes	4.11E-15	DKK1 | INSL4 | S100A10 | SHC1 | SEMA3C | OXTR | BTK | CCL20 | FURIN | PSMD2 | ADIPOR2 | SCG2 | GPI
13 genes	4.11E-15	VEGFC | INSL4 | CCR6 | SHC1 | SEMA3C | OXTR | BTK | CCL20 | FURIN | PSMD2 | TNFRSF11A | SCG2 | GPI
13 genes	4.11E-15	INSL4 | F2RL1 | FCGRT | CCR6 | SHC1 | OXTR | PSMC1 | CCL20 | FURIN | PSMD2 | TNFRSF11A | SCG2 | GPI
13 genes	4.11E-15	F2RL1 | RFXAP | FCGRT | CCR6 | S100A10 | SHC1 | OXTR | BTK | CCL20 | FURIN | PSMD2 | ADIPOR2 | SCG2
14 genes	4.11E-15	DKK1 | F2RL1 | CCR6 | S100A10 | SHC1 | SEMA3C | OXTR | BTK | PSMC1 | CCL20 | ADIPOR2 | TNFRSF11A | SCG2 | GPI
14 genes	4.11E-15	INSL4 | F2RL1 | RFXAP | FCGRT | CCR6 | S100A10 | SHC1 | BTK | PSMC1 | CCL20 | PSMD2 | TNFRSF11A | SCG2 | GPI
15 genes	4.11E-15	VEGFC | F2RL1 | RFXAP | CCR6 | S100A10 | SHC1 | SEMA3C | OXTR | BTK | CCL20 | FURIN | PSMD2 | ADIPOR2 | SCG2 | GPI
9 genes	4.22E-15	INSL4 | F2RL1 | CCR6 | PSMC1 | CCL20 | FURIN | PSMD2 | ADIPOR2 | GPI
9 genes	4.22E-15	F2RL1 | FCGRT | CCR6 | SEMA3C | CCL20 | PSMD2 | TNFRSF11A | SCG2 | GPI
9 genes	4.22E-15	FCGRT | CCR6 | SHC1 | SEMA3C | OXTR | CCL20 | PSMD2 | TNFRSF11A | GPI
10 genes	4.22E-15	DKK1 | INSL4 | CCR6 | SHC1 | SEMA3C | BTK | CCL20 | TNFRSF11A | SCG2 | GPI

Table S4. Enriched gene sets of HALLMARK collection (| NES |> 1, NOM p-val <0.05, and FDR q-val <0.25).

NAME	ES	NES	NOM p-val	FDR q-val
HALLMARK_GLYCOLYSIS	0.6369638	2.5964317	0	0
HALLMARK_MTORC1_SIGNALING	0.61170495	2.2989163	0	0
HALLMARK_MYC_TARGETS_V1	0.6872714	2.2366955	0	2.65E-04
HALLMARK_HYPOXIA	0.5492785	2.1221604	0	0.003025117
HALLMARK_UNFOLDED_PROTEIN_RESPONSE	0.5240902	2.127752	0.00203666	0.003595226
HALLMARK_ESTROGEN_RESPONSE_LATE	0.4220594	1.7995743	0.00203666	0.0378938
HALLMARK_G2M_CHECKPOINT	0.7621759	1.9917821	0.00212766	0.008661938
HALLMARK_E2F_TARGETS	0.7668138	1.915678	0.00212766	0.015834505
HALLMARK_MYC_TARGETS_V2	0.72222024	2.0134616	0.002155172	0.007724643
HALLMARK_REACTIVE_OXYGEN_SPECIES_PATHWAY	0.5151387	1.7574512	0.010416667	0.04922938
HALLMARK_ANGIOGENESIS	0.60289335	1.7296876	0.018796992	0.048142523
HALLMARK_UV_RESPONSE_UP	0.35204056	1.6443462	0.018828452	0.07107789
HALLMARK_MITOTIC_SPINDLE	0.5572468	1.7544369	0.021825397	0.04508069
HALLMARK_EPITHELIAL_MESENCHYMAL_TRANSITION	0.64020175	1.7259194	0.038910504	0.04582746
HALLMARK_DNA_REPAIR	0.4068548	1.691844	0.04025424	0.056149222

Table S5. The Spearman's rank correlation coefficient tests the correlation between each TIC and the gene signature.

TIC	r	p-value
Macrophages.M0	0.271167529	5.12E-08
Mast.cells.resting	-0.2356071	2.47E-06
Mast.cells.activated	0.212880273	2.19E-05
T.cells.CD4.memory.resting	-0.18605559	0.00021595
Neutrophils	0.165496852	0.00102095
Dendritic.cells.resting	-0.14858161	0.00322988
Dendritic.cells.activated	0.146474709	0.00369904
T.cells.CD8	-0.10455058	0.03882968
B.cells.memory	-0.10095621	0.04604419
T.cells.CD4.naive	0.0961929	0.05737925
Monocytes	-0.09062125	0.07347538
NK.cells.resting	0.085517598	0.09128043
T.cells.CD4.memory.activated	0.080626251	0.11143663
T.cells.follicular.helper	0.080271019	0.11302702
Eosinophils	0.078872502	0.11946196
Macrophages.M1	-0.05855988	0.24799586
T.cells.gamma.delta	-0.05508037	0.27726596
T.cells.regulatory..Tregs.	-0.05074412	0.31690788
B.cells.naive	0.023174008	0.64779281
NK.cells.activated	0.010012421	0.84355062
Plasma.cells	-0.00356959	0.94390852
Macrophages.M2	-0.00062594	0.9901534

Table S6. The Kaplan-Meier estimator detects the prognostic ability of each TIC.

TIC	P-value
Mast.cells.resting	0.00061317
Dendritic.cells.resting	0.02270994
B.cells.memory	0.06549356
Mast.cells.activated	0.09517642
Macrophages.M0	0.12146742
Monocytes	0.14255326
Macrophages.M1	0.17839695
NK.cells.resting	0.21829716
T.cells.CD4.memory.activated	0.24854026
Neutrophils	0.26505376
Plasma.cells	0.43978489
Macrophages.M2	0.5779231
T.cells.follicular.helper	0.61044847
Dendritic.cells.activated	0.6242769
T.cells.regulatory..Tregs.	0.63727822
T.cells.CD4.naive	0.69714507
B.cells.naive	0.87054954
T.cells.CD8	0.92482278
Eosinophils	0.93401179
T.cells.CD4.memory.resting	0.96665969
NK.cells.activated	0.99268104
T.cells.gamma.delta	0.99649767
